# Oligonucleotide usage in coronavirus genomes mimics that in exon regions in host genomes

**DOI:** 10.1186/s12985-023-01995-3

**Published:** 2023-03-01

**Authors:** Yuki Iwasaki, Takashi Abe, Toshimichi Ikemura

**Affiliations:** 1 Department of Bioscience, Nagahama Institute of Bio-Science and Technology, Tamura-Cho 1266, Nagahama-Shi, Shiga-Ken 526-0829 Japan; 2 Department of Information Engineering, Graduate School of Science and Technology, Niigata University, Niigata-Ken, 950-2181 Japan

**Keywords:** SARS-CoV-2, Oligonucleotide frequency, Genome, Exon region, Zoonoses, Pandemic, Zinc-finger antiviral protein

## Abstract

**Background:**

Viruses use various host factors for their growth, and efficient growth requires efficient use of these factors. Our previous study revealed that the occurrence frequency of oligonucleotides in the influenza virus genome is distinctly different among derived hosts, and the frequency tends to adapt to the host cells in which they grow. We aimed to study the adaptation mechanisms of a zoonotic virus to host cells.

**Methods:**

Herein, we compared the frequency of oligonucleotides in the genome of alpha- and betacoronavirus with those in the genomes of humans and bats, which are typical hosts of the viruses.

**Results:**

By comparing the oligonucleotide frequency in coronaviruses and their host genomes, we found a statistically tested positive correlation between the frequency of coronaviruses and that of the exon regions of the host from which the virus is derived. To examine the characteristics of early-stage changes in the viral genome, which are assumed to accompany the host change from non-humans to humans, we compared the oligonucleotide frequency between severe acute respiratory syndrome coronavirus 2 (SARS-CoV-2) at the beginning of the pandemic and the prevalent variants thereafter, and found changes towards the frequency of the host exon regions.

**Conclusions:**

In alpha- and betacoronaviruses, the genome oligonucleotide frequency is thought to change in response to the cellular environment in which the virus is replicating, and actually the frequency has approached the frequency in exon regions in the host.

**Supplementary Information:**

The online version contains supplementary material available at 10.1186/s12985-023-01995-3.

## Background

Viruses have always posed threats to public health, as highlighted by the current severe acute respiratory syndrome coronavirus 2 (SARS-CoV-2) pandemic [1, 2], the ebolavirus outbreak in West Africa in 2014 [3–6], and the emerging and re-emerging nature of influenza viruses [7]. To address the worldwide threats caused by zoonotic RNA viruses, which suddenly cause serious outbreaks by invasion from non-human hosts, we must understand the molecular evolutionary changes in their genome sequences from various aspects. Viral growth depends on many host factors (e.g. nucleotide pools, proteins, and RNAs), but human cells may not provide ideal growth conditions for viruses invading non-human hosts. Our previous time-series analyses of short and long oligonucleotide compositions in the above-mentioned virus genomes showed directional changes in their composition after the invasion from non-human hosts, that is, a time-series monotonic increase or decrease trend [8–10]. In the case of human influenza A viruses, common directional changes have been observed for three subtypes (H1N1, H3N2, and H1N1pdm09), which have invaded the human population independently over long intervals, such as several decades [9, 11]. Interestingly, the destination of these reproducible directional changes appears to be the composition of influenza B [11], which can currently infect humans but not birds.

The category, nature, and content of factors available in host cells should differ among hosts; therefore, when a virus invades a new host population and causes an epidemic, its genome is altered so that it can efficiently utilize host factors and evade the host’s antiviral mechanisms [12–14]. A wide variety of oligonucleotides are known to be functional motifs (e.g. those for binding to host proteins and RNAs) [15–17], and thus, the bias of host-dependent oligonucleotide composition may provide insight into the molecular mechanism of host adaptation of the virus.

In this study, we analyzed coronaviruses, including SARS-CoV-2. Most viruses belonging to Coronaviridae use bats as their natural reservoir hosts, and some can infect a wide range of mammals and birds [18]. In humans, four types (Alpha 229E, Alpha NL63, Beta HKU1, and Beta OC43) are prevalent and known to cause common colds [18]. In the past two decades, viruses, such as SARS, MERS, and SARS-CoV-2, have posed a serious threat to humans [1, 19, 20]. When these coronaviruses have invaded human populations from non-human hosts, their genome sequences are likely to change to efficiently utilize human cell factors and avoid human antiviral functions. We previously examined time-series changes in oligonucleotide frequencies in SARS-CoV-2 genomes, mainly during the first year of the pandemic, and found that a variety of oligonucleotides changed their frequency in a directional manner [21–23]. We believe that this study could facilitate the elucidation of the host adaptation mechanisms of the virus.

## Methods

### Genome sequences of coronaviruses prevalent in humans or bats

The complete sequences of two types of human coronaviruses (Human-CoV), alphacoronaviruses (27 229E and 55 NL63 strains) and betacoronaviruses (18 HKU1 and 138 OC43 strains), were obtained from the NCBI virus database (https://www.ncbi.nlm.nih.gov/labs/virus/). The complete genome sequences of two types of bat coronavirus (Bat-CoV), alphacoronaviruses (87 strains) and betacoronaviruses (79 strains, including 34 SARS-CoV), isolated from three bat species (Chiroptera, Vespertilionidae, and Rhinolophidae), were obtained from the NCBI virus database. The poly(A) tail of each sequence was removed prior to the analysis. The strains that were used are listed in (see Additional file 2: Table S1).

### Human and bat genome sequences and annotation information

Human genome sequence (GRCh38) and annotation data, including exon–intron information, were obtained from the Ensembl Genome Browser (https://asia.ensembl.org/Homo_sapiens/Info/Index). Six bat genome sequences and annotation data, including exon–intron information, were obtained from Bat1k (https://bds.mpi-cbg.de/hillerlab/Bat1KPilotProject/) [24].

### SARS-CoV-2 genome sequences

The complete genome sequences of five variants (alpha, beta, delta, gamma, and omicron) of SARS-CoV-2 were obtained from the GISAID database (https://www.gisaid.org/); sequences that were complete, with high coverage, and from humans were downloaded on November 29, 2021. For each variant, the number of isolates per month was counted, and only the genome data from the month with the highest number of isolates were used for the present analysis after removing the poly(A) tail. These strains are listed in (see Additional file 3: Data S1, S2, S3, S4, S5, and S6 ).

### Statistical analyses

To compare the frequency of oligonucleotides between human- and bat-CoVs, we calculated the frequency of each viral genome; the program to calculate frequency of oligonucleotides can be obtained from a GitHub repository (https://github.com/yukakokatsura/BLSOM). To test whether there was a significant difference in oligonucleotide frequency between human- and bat-CoV, a *t*-test was performed using the *t*-test function in R. The *p* values obtained from the tests were corrected using the BH method [25]. For comparison of the oligonucleotide frequency in exon (or intron) regions between humans and bats, the regions were extracted from each gene, the number of each oligonucleotide was summed up for each gene, and the statistical analysis of the frequency was conducted as described above. For comparison of the oligonucleotide frequency between the genomes of humans and bats, the genome sequence of each species was fragmented into 100-kb pieces, the frequency was calculated for each fragment, and statistical analysis was conducted as described above. Similar results were obtained when the fragment size was set to 1 Mb.

### Degree of adaptation

We characterized the degree of adaptation of each oligonucleotide to the human cellular environment of SARS-CoV-2 using the following two-step method:

In the first step, we calculated the midpoint between human-CoV and bat-CoV frequencies to be used as a baseline for examining whether the human or bat cellular environment is more suitable for each oligonucleotide.

The baseline was calculated using the following formula:

$${\text{Xi}}\, = \,\left( {{\text{Hi}}\, + \,{\text{Bi}}} \right)/{2}$$

where Xi is the baseline of oligonucleotide i, and Hi and Bi are the frequencies of oligonucleotide i of human-CoV and bat-CoV, respectively.

In the second step, for each oligonucleotide, the log_2_ fold change in frequency of the baseline was calculated using the following formula:

$${\text{FCi}}\, = \,{\text{log}}_{{2}} \,\left( {{\text{Ci}}/{\text{Xi}}} \right)$$

where FCi is the log_2_ fold change of oligonucleotide i, and Ci is the frequencies of oligonucleotide i of SARS-CoV-2.

## Results

### Comparison of human and bat coronaviruses

The genome sequence of coronaviruses that cause human common colds, which have been prevalent in humans for a long time, may be well-adapted for growth and transmission in the human population. Thus, their comparison with the genomes of coronaviruses isolated from bats will provide clues regarding the molecular mechanisms involved in adaptation to human cells. Here, the occurrence frequency of short oligonucleotides (di- to tetra-nucleotides) in the genome sequences of 238 human-CoV (27 strains of Alpha 229E, 55 strains of Alpha-NL63, 18 strains of Beta-HKU1, and 138 strains of Beta-OC43) and 166 bat-CoV (87 strains of Alpha-CoV and 79 strains of Beta-CoV) strains were compared (see Additional file 2: Table S2). This comparison showed that the frequency of 87 oligonucleotides (6 di-, 18 tri-, and 63 tetra-nucleotides) was significantly higher (*t*-test; FDR ≤ 0.05) in human-CoV, while the frequency of 224 oligonucleotides (10 di-, 41 tri-, and 173 tetra-nucleotides) was significantly higher in bat-CoV (Table 1). This host-dependent preference may be related to molecular mechanisms supporting coronaviruses to efficiently replicate in human or bat cells and escape the antiviral mechanisms of each host.

**Table 1:** The list of oligonucleotides with significant differences in frequency between human-CoV and bat-CoV

	Preferred in human-CoV	Preferred in Bat-CoV
Di	AA, AU, GU, UA, UG, UU	AC, AG, CA, CC, CG, CU, GA, GC, GG, UC
Tri	AAA, AAG, AAU, AGU, AUA, AUG, AUU, GAU, GUA, GUU, UAA, UAG, UAU, UGA, UGU, UUA, UUG, UUU	AAC, ACA, ACC, ACG, ACU, AGC, AGG, CAA, CAC, CAG, CAU, CCA, CCC, CCG, CCU, CGA, CGC, CGG, CGU, CUA, CUC, CUG, CUU, GAA, GAC, GAG, GCA, GCC, GCG, GCU, GGC, GGG, GUC, GUG, UAC, UCA, UCC, UCG, UCU, UGC, UUC
Tetra	AAAA, AAAG, AAAU, AAGC, AAGU, AAUA, AAUC, AAUG, AAUU, AGAU, AGUA, AGUU, AUAA, AUAC, AUAG, AUAU, AUCU, AUGA, AUGG, AUGU, AUUA, AUUC, AUUG, AUUU, CUAA, GAAU, GAUA, GAUC, GAUG, GAUU, GGAU, GGUA, GGUU, GUAA, GUAU, GUGU, GUUA, GUUG, GUUU, UAAA, UAAG, UAAU, UACU, UAGA, UAGU, UAUA, UAUC, UAUG, UAUU, UGAU, UGGA, UGGU, UGUA, UGUU, UUAA, UUAG, UUAU, UUGA, UUGG, UUGU, UUUA, UUUG, UUUU	AAAC, AACA, AACC, AACG, AACU, AAGG, ACAA, ACAC, ACAG, ACAU, ACCA, ACCC, ACCG, ACCU, ACGA, ACGC, ACGG, ACGU, ACUA, ACUC, ACUG, ACUU, AGAC, AGAG, AGCA, AGCC, AGCG, AGCU, AGGC, AGGG, AGGU, AGUC, AGUG, AUCC, AUCG, CAAA, CAAC, CAAG, CAAU, CACA, CACC, CACG, CACU, CAGA, CAGC, CAGG, CAGU, CAUC, CAUG, CCAA, CCAC, CCAG, CCAU, CCCA, CCCC, CCCG, CCCU, CCGA, CCGC, CCGG, CCGU, CCUA, CCUC, CCUG, CCUU, CGAA, CGAC, CGAG, CGAU, CGCA, CGCC, CGCG, CGCU, CGGA, CGGC, CGGG, CGGU, CGUA, CGUC, CGUG, CGUU, CUAC, CUAG, CUAU, CUCA, CUCC, CUCG, CUCU, CUGA, CUGC, CUGG, CUGU, CUUA, CUUC, CUUG, CUUU, GAAC, GAAG, GACA, GACC, GACG, GACU, GAGA, GAGC, GAGG, GCAA, GCAC, GCAG, GCCA, GCCC, GCCG, GCCU, GCGA, GCGC, GCGG, GCGU, GCUC, GCUG, GCUU, GGAA, GGAC, GGAG, GGCA, GGCC, GGCG, GGCU, GGGA, GGGC, GGGU, GGUC, GGUG, GUAC, GUCA, GUCC, GUCG, GUCU, GUGA, GUGC, GUGG, UAAC, UACA, UACC, UACG, UCAA, UCAC, UCAG, UCAU, UCCA, UCCC, UCCG, UCCU, UCGA, UCGC, UCGG, UCGU, UCUA, UCUC, UCUG, UGAC, UGAG, UGCA, UGCC, UGCG, UGCU, UGGC, UGGG, UGUC, UUAC, UUCA, UUCC, UUCG, UUGC, UUUC

It is clear from Table 1 that human-CoV has a lower frequency of C-containing oligonucleotides and a higher frequency of U-containing oligonucleotides. Several studies, including ours, have found that a large portion of mutations in SARS-CoV-2 are C to U mutations, which are thought to be the result of RNA editing by APOBEC3G [21, 26, 27]. The effect of APOBEC3G is thought to be related to an antiviral mechanism in humans. However, if a certain C-to-U mutation is favorable to the virus, rather than unfavorable or neutral, this advantageous mutation will rapidly expand its frequency in the viral population. This type of complexity was assumed for the effect of APOBEC3G.

### Host genome differences

When considering the molecular mechanisms of the host-dependent preference for oligonucleotide usage in coronaviruses, it would be interesting to compare it with the usage frequency of host genomes and mRNAs. Greenbaum et al*.* [28] reported that single-stranded RNA viruses, including influenza viruses, mimic their dinucleotide frequency (especially CG frequency) to reflect the frequency in their host genomes. In the present study, we first calculated the oligonucleotide frequency in human and bat genomes and compared it to that of coronaviruses isolated from these hosts (see Additional file 2: Table S3); for the host genomes, exon and intron regions were analyzed separately to obtain information on mRNAs. For the bat, we intitailly analyzed *Rhinolophus ferrumequinum*, a member of the Rhinolophidae family, from which various coronaviruses, including SARS, have been isolated [29, 30]. Comparing oligonucleotide frequencies in the human and bat exon regions, 147 oligonucleotides (7 di-, 27 tri-, and 113 tetra-nucleotides) were more frequent in humans, whereas 172 oligonucleotides (9 di-, 35 tri-, and 128 tetra-nucleotides) were more frequent in bats (Table 2). In the case of intron regions, 127 oligonucleotides (5 di-, 26 tri-, and 96 tetra-nucleotides) were more frequent in humans, but 186 oligonucleotides (7 di-, 34 tri-, and 145 tetra-nucleotides) were more frequent in bats (see Additional file 2: Table S4), showing a difference between the two genomic regions.

**Table 2:** Oligonucleotides with significant differences in frequency between human exon and bat exon

	Preferred in human exon	Preferred in Bat exon
Di	AA, AU, CU, GU, UA, UC, UU	AC, AG, CA, CC, CG, GA, GC, GG, UG
Tri	AAA, AAU, ACU, AGG, AGU, AUA, AUU, CAU, CCU, CUA, CUC, CUU, GGG, GGU, GUA, GUU, UAA, UAG, UAU, UCA, UCU, UGA, UGU, UUA, UUC, UUG, UUU	AAC, AAG, ACA, ACC, ACG, AGA, AGC, AUC, AUG, CAA, CAC, CAG, CCA, CCC, CCG, CGA, CGC, CGG, CGU, CUG, GAA, GAC, GAG, GCA, GCC, GCG, GCU, GGA, GGC, GUC, GUG, UAC, UCG, UGC, UGG
Tetra	AAAA, AAAC, AAAU, AACU, AAGU, AAUA, AAUC, AAUG, AAUU, ACAU, ACUA, ACUC, ACUU, AGGC, AGGG, AGGU, AGUA, AGUC, AGUG, AGUU, AUAA, AUAC, AUAG, AUAU, AUCU, AUGU, AUUA, AUUC, AUUG, AUUU, CAAA, CAAU, CACU, CAGG, CAUA, CAUG, CAUU, CCUA, CCUC, CCUU, CUAA, CUAG, CUAU, CUCA, CUCC, CUCU, CUGU, CUUA, CUUG, CUUU, GAAU, GAGU, GAUA, GAUU, GCAU, GCCU, GCUA, GCUU, GGGA, GGGU, GGUA, GGUU, GUAA, GUAG, GUAU, GUCU, GUGA, GUGU, GUUA, GUUG, GUUU, UAAA, UAAC, UAAG, UAAU, UACA, UACU, UAGA, UAGC, UAGG, UAGU, UAUA, UAUC, UAUG, UAUU, UCAU, UCCC, UCCU, UCUA, UCUC, UCUG, UCUU, UGAG, UGAU, UGGG, UGUA, UGUG, UGUU, UUAA, UUAC, UUAG, UUAU, UUCA, UUCC, UUCU, UUGA, UUGC, UUGG, UUGU, UUUA, UUUC, UUUG, UUUU	AAAG, AACC, AACG, AAGA, AAGC, AAGG, ACAA, ACAC, ACAG, ACCA, ACCC, ACCG, ACCU, ACGA, ACGC, ACGG, ACGU, AGAA, AGAC, AGAG, AGCA, AGCC, AGCG, AGCU, AGGA, AUCA, AUCC, AUCG, AUGA, AUGC, AUGG, CAAC, CAAG, CACC, CACG, CAGA, CAGC, CAUC, CCAA, CCAC, CCAG, CCAU, CCCC, CCCG, CCGA, CCGC, CCGG, CCGU, CCUG, CGAA, CGAC, CGAG, CGAU, CGCA, CGCC, CGCG, CGCU, CGGA, CGGC, CGGG, CGGU, CGUA, CGUC, CGUG, CGUU, CUAC, CUCG, CUGC, CUGG, CUUC, GAAA, GAAC, GAAG, GACA, GACC, GACG, GAGA, GAGC, GAGG, GAUC, GAUG, GCAA, GCAC, GCAG, GCCA, GCCC, GCCG, GCGA, GCGC, GCGG, GCGU, GCUC, GCUG, GGAA, GGAC, GGAG, GGCA, GGCC, GGCG, GGGC, GGUC, GGUG, GUAC, GUCA, GUCC, GUCG, GUGC, GUGG, GUUC, UACC, UACG, UCAA, UCCA, UCCG, UCGA, UCGC, UCGG, UCGU, UGAA, UGAC, UGCA, UGCC, UGCG, UGCU, UGGA, UGGC, UGGU, UUCG

### Host genome differences and host-dependent differences in viral genomes

**Fig. 1:**
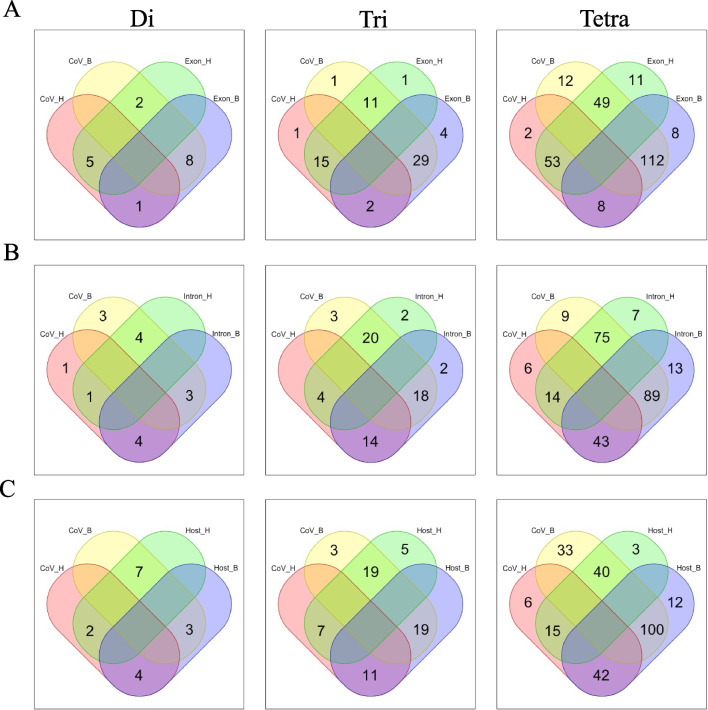
Oligonucleotide frequencies in coronavirus genomes correlate with those of their host exon region. Venn diagrams of di-, tri-, and tetra-nucleotides that were specifically high in frequency in coronaviruses and host genomes. **A** Host exons, **B** Host introns, and **C** 100 kb fragments of human and bat genomes. In the comparison of oligonucleotide frequency between human- and bat-CoV, the oligonucleotide that was more frequent in human-CoV is denoted by CoV_H, and the oligonucleotide that was more frequent in bat-CoV is denoted by CoV_B. In the comparison of oligonucleotide frequency between human and bat exons, the oligonucleotide that was more frequent in human exons is denoted by Exon_H, and the oligonucleotide that was more frequent in bat exons is denoted by Exon_B. In the comparison of oligonucleotide frequency between human- and bat-introns, the oligonucleotide that was more frequent in human intron is denoted by Intron_H, and the oligonucleotide that was more frequent in bat intron is denoted by Intron_B. In the comparison of oligonucleotide frequency between 100 kb fragments of human and bat genomes, the oligonucleotide that was more frequent in the human genome is denoted by Host_H, and the oligonucleotide that was more frequent in the bat genome is denoted by Host_B

We then compared host genome differences with host-dependent preferences in viral genomes. Of the 147 oligonucleotides that were more frequent in human exon regions, 73 (5 di-, 15 tri-, and 53 tetra-nucleotides) were more frequent in human-CoV. Of the 172 oligonucleotides that were more frequent in the bat exon regions, 149 (8 di-, 29 tri-, and 112 tetra-nucleotides) were more frequent in bat-CoV (Fig. 1A, B), suggesting a higher degree of host adaptation within the bat virus. Notably, in the case of intron regions, the preference differed from that of the exon regions. Of the 127 oligonucleotides that were more frequent in human intron regions, only 19 (1 di-, 4 tri-, and 14 tetra-nucleotides) were more frequent in human-CoV, and of the 186 oligonucleotides that were more frequent in bat intron regions, only 110 (3 di-, 18 tri-, and 89 tetra-nucleotides) were more frequent in bat-CoV (Fig. 1B). The finding that the viral genome is more similar to exonic regions than to intronic regions may be related to the fact that viral RNAs are located only in the cytoplasm but not in the cell nucleus.

Next, we analyzed the frequency of oligonucleotides in whole genomes of humans and bats, which included intergenic regions (see Additional file 2: Table S5), and the results were closer to those of the intron region than the exon region (Fig. 1C). Collectively, the oligonucleotide frequencies in the coronavirus genomes were more closely related to the frequency in exon regions; therefore, we focused on exon regions in the following analyses.

### Characteristics of exon regions of other bats

**Fig. 2:**
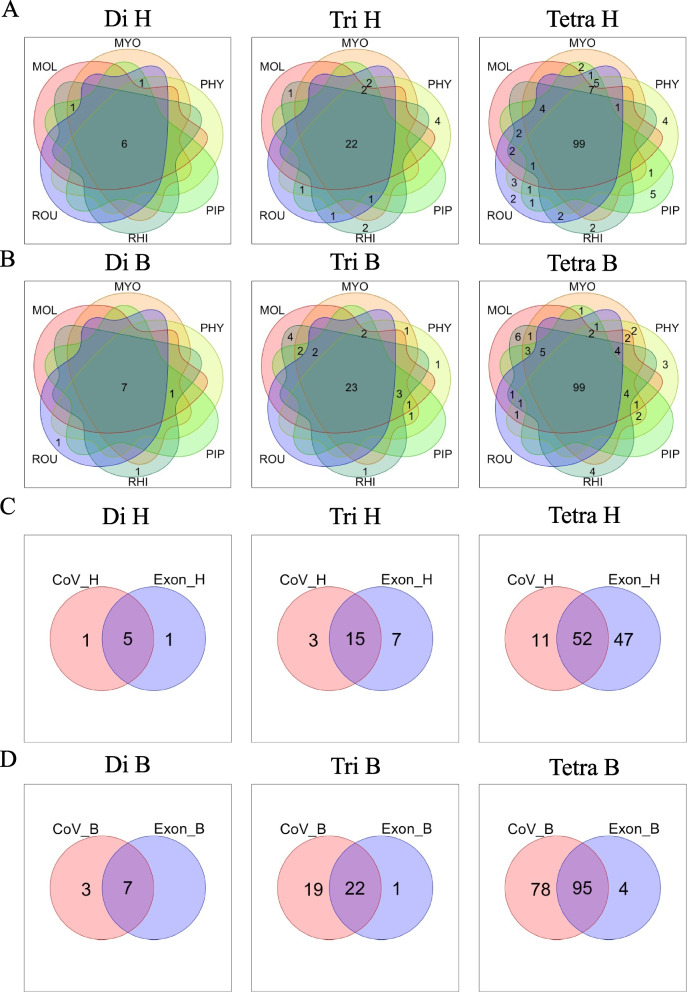
Correlation of oligonucleotide frequencies between virus genome and exon region of host animals. **A**, **B** Venn diagram showing di-, tri-, and tetra-nucleotides that were specifically high in frequency in host animals. The oligonucleotides that are (**A**) more or (**B**) less frequent in the human exon region than the exon region in each bat. The notation in the Venn diagram is as follows. MOL: *Molossus molossus*, MYO: *Myotis myotis*, PHY: *Phyllostomus discolor*, PIP: *Pipistrellus kuhlii*, RHI: *Rhinolophus ferrumequinum*, ROU: *Rousettus aegyptiacus*. **C**, **D** Venn diagram showing di-, tri-, and tetra-nucleotides that were specifically high in frequency in human-CoV and the human genome and in Bat-CoV and the bat genome, respectively

In the above analysis, we used the genome data of *R. ferrumequinum*; however, coronaviruses have been isolated from various bat species. To obtain a much broader perspective on the host-dependent adaptation of coronaviruses, we analyzed five additional species. *R. ferrumequinum* is a member of the Microchiroptera family, which includes *Phyllostomus discolor*, *Myotis myotis*, *Pipistrellus kuhlii*, and *Molossus molossus*, and sequences and annotation data of the latter four were also obtained from the Bat1K Project [24]. *Rousettus aegyptiacus*, a member of Megachiroptera, was also analyzed. It should be noted that various coronaviruses have been isolated from bats or their relatives [31]. As conducted for *R. ferrumequinum*, we calculated the oligonucleotide frequency in the exon regions of each bat and compared it with the frequency in human exon regions (see Additional file 2: Table S6). In each comparison, there was a significant difference (*t*-test; FDR < 0.05) in the frequency of approximately 300 oligonucleotides (Fig. 2A, B). Importantly, a large portion is common among six bats; 127 oligonucleotides (6 di-, 22 tri-, and 99 tetra-nucleotides) and 129 oligonucleotides (7 di-, 23 tri-, and 99 tetra-nucleotides) were commonly found at higher and lower frequencies, respectively, than in human exon regions. The selection of common features among the six bats slightly reduced the number of focal oligonucleotides compared to the results obtained for *R. ferrumequinum* alone. These oligonucleotides are considered to characterize exon regions in a wide phylogenetic range of bats, differentially from human exons. Next, as shown in Table 1 and Fig. 1, we compared the results of host exon differences with host-dependent preferences in viral genomes.

As shown in Fig. 2C, D, from the 127 oligonucleotides that were used more frequently in human exon regions, 72 oligonucleotides (5 di-, 15 tri-, and 52 tetra-nucleotides) were more frequently used in human-CoV. From the 129 oligonucleotides that were used more frequently in exon regions of bats, 124 (7 di-, 22 tri-, and 95 tetra-nucleotides) were used more frequently in bat-CoV, suggesting a higher degree of host adaptation on the bat virus side. These 72 and 124 oligonucleotides, which show common features for viral genomes and host exon regions, are thought to be landmarks for the adaptation of coronaviruses to their respective hosts. We defined each oligonucleotide set as “Human- or Bat-type oligonucleotide” (Table 3). Bat-CoV appears to have a greater degree of adaptation to its host than human-CoV, which may be related to the fact that bats have been natural reservoir hosts of bat-CoV for a long period of evolution.

**Table 3:** The list of human- and bat-type oligonucleotides

	Human-type	Bat-type
Di	AA, AU, GU, UA, UU	AC, CA, CC, CG, GA, GC, GG
Tri	AAA, AAU, AGU, AUA, AUU, GUA, GUU, UAA, UAG, UAU, UGA, UGU, UUA, UUG, UUU	ACC, ACG, AGC, CAC, CAG, CCA, CCC, CCG, CGA, CGC, CGG, CGU, GAC, GAG, GCA, GCC, GCG, GCU, GGC, GUC, UCG, UGC
Tetra	AAAA, AAAU, AAGU, AAUA, AAUC, AAUG, AAUU, AGUA, AGUU, AUAA, AUAC, AUAG, AUAU, AUCU, AUGU, AUUA, AUUC, AUUG, AUUU, CUAA, GAAU, GAUA, GAUU, GGUA, GGUU, GUAA, GUAU, GUUA, GUUG, GUUU, UAAA, UAAG, UAAU, UACU, UAGA, UAGU, UAUA, UAUC, UAUG, UAUU, UGAU, UGUA, UGUU, UUAA, UUAG, UUAU, UUGA, UUGG, UUGU, UUUA, UUUG, UUUU	AACG, AAGG, ACCA, ACCC, ACCG, ACCU, ACGA, ACGC, ACGG, ACGU, AGCA, AGCC, AGCG, AUCG, CAAC, CAAG, CACC, CACG, CAGA, CAGC, CAUC, CCAA, CCAC, CCAG, CCCC, CCCG, CCGA, CCGC, CCGG, CCGU, CCUG, CGAA, CGAC, CGAG, CGAU, CGCA, CGCC, CGCG, CGCU, CGGA, CGGC, CGGG, CGGU, CGUC, CGUG, CGUU, CUAC, CUCG, CUGC, CUGG, CUUC, GAAC, GAAG, GACA, GACC, GACG, GAGA, GAGC, GAGG, GCAC, GCAG, GCCA, GCCC, GCCG, GCGA, GCGC, GCGG, GCGU, GCUC, GCUG, GGAC, GGAG, GGCA, GGCC, GGCG, GGGC, GGUC, GUAC, GUCC, GUCG, GUGC, GUGG, UACC, UACG, UCCA, UCCG, UCGA, UCGC, UCGG, UCGU, UGCC, UGCG, UGCU, UGGC, UUCG

Underlined oligonucleotides indicate those in SARS-CoV-2 with a frequency closer to human-CoV than to bat-CoV at the beginning of the epidemic

### SARS-CoV-2 at the beginning of the epidemic

**Fig. 3:**
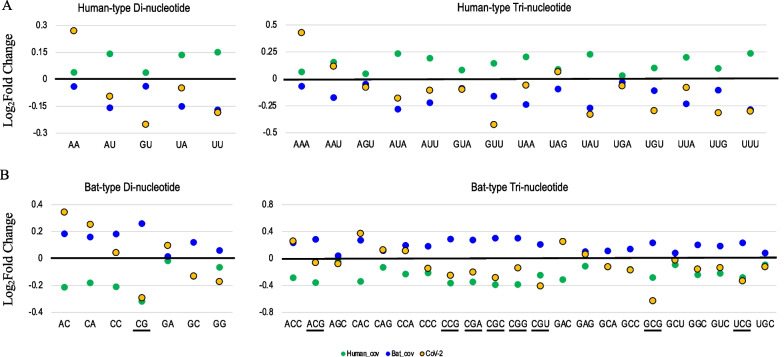
Level of adaptation of each di- and tri-nucleotide to the human cellular environment in SARS-CoV-2. **A**, **B** The level of adaptation of the human- and bat-type oligonucleotides in the three virus types (human-CoV, bat-CoV, and SARS-CoV-2) is displayed. This level is the ratio of the averaged oligonucleotide frequency in each virus to the baseline, which is the midpoint between human- and bat-CoV frequencies. The ratio of SARS-CoV-2 for each oligonucleotide was displayed as an orange-filled circle surrounded by a black line, and those of human- or bat-CoV was displayed as a green or blue circle, respectively

SARS-CoV-2, whose natural reservoir host is believed to be bats, has recently invaded the human population, possibly through an intermediate host animal [1]. We examined the extent to which SARS-CoV-2 had human- or bat-type characteristics at the beginning of the pandemic. This may indicate the potentiality of initiating an outbreak in the human population and be useful in the search for dangerous strains found in a non-human animal that are feared to cause a pandemic in the future. First, for the SARS-CoV-2 population in the early epidemic stage (22 strains isolated in December 2019), we examined whether the frequency of each focal oligonucleotide listed in Table 3 was similar to that of human- or bat-CoV. In more detail, as described in “Degree of adaptation” in the Methods section, the midpoint between the human- and bat-CoV frequencies was first calculated for each focal oligonucleotide and assumed as a reference level. For each oligonucleotide, we calculated the average frequency of 22 SARS-CoV-2 strains isolated in December 2019 (CoV-2_2019), although their genome sequences were very similar. We next obtained the ratio of this average frequency to the above reference level and showed the log_2_ fold change using an orange-filled circle surrounded by a black line (Fig. 3). For confirmation, the fold-change of human- or bat-CoV to the reference level is displayed in green or blue circles, respectively.

Human-type oligonucleotides, which were determined by incorporating the characteristics of human mRNAs, were examined (Fig. 3A (Di and Tri) and [see Additional file 1: Fig. S1A (Tetra)]). Green circles for human-CoV and blue circles for bat-CoV are located on the upper and lower side by definition, respectively. In the case of CoV-2_2019, the majority of oligonucleotides were located on the lower side and lacked human-type characteristics, showing that the characteristics of bat-CoV prevailed at the start of the SARS-CoV-2 epidemic even though the virus was isolated from humans. Only about 1/4 of the 72 human-type oligonucleotides are human-CoV types, with AA being a typical example of dinucleotides. The abundance of AA is also the case for tri- and tetranucleotides, and that of U also contributes.

Bat-type oligonucleotides, which were determined by incorporating the characteristics of bat mRNAs, were analyzed (Fig. 3B (Di and Tri) and [see Additional file 1: Fig. S1B (Tetra)]). Green circles for human-CoV (or bat-Cov) are located on the lower (or upper) side by definition. Interestingly, for bat-type dinucleotides, there were three cases with characteristics of human-CoV rather than those of bat-CoV. Notably, in the case of CG (underlined in Fig. 3B), the difference in the vertical position between the blue and green circles is large, showing that the difference between bat- and human-CoV is very large, and the level of CoV-2_2019 is almost equal to that of human-CoV. The trinucleotides, including CG (underlined in Fig. 3B), again showed a large difference in the vertical position between the green and blue circles, and the orange circles of SARS-CoV-2 were close to the green circles of human-CoV. Collectively, SARS-CoV-2 appears to have lost appreciably of the characteristics of bat-type oligonucleotides, even at the beginning of its outbreak in the human population. This loss (e.g., low CG levels) may reflect the changes that had already occurred in the non-human host prior to entry into the human population, and may suggest a possible requirement for initiating an efficient outbreak in the human population.

CG is known to be a target of zinc-finger antiviral protein (ZAP), one of the host antiviral systems, and a variety of RNA viruses, including coronaviruses, have been shown to escape the antiviral action of ZAP by maintaining low CG levels [26]. In the present study, SARS-CoV-2 was found to reduce the frequency of CG-containing oligonucleotides to almost the same level as that of human-CoV even at the beginning of the epidemic. This lower CG level would have most likely been achieved in non-human intermediate SARS-CoV-2 hosts. However, there are examples of bat-type oligonucleotides, especially those unrelated to CG, that are not of the human-CoV type (Fig. 3B). In addition, there are more cases of human-type oligonucleotides that are not of the human-CoV type (Fig. 3A). This suggests that the characteristics of intermediate host mRNAs likely differ from those of human mRNAs.

### Changes in SARS-CoV-2 genomes

**Fig. 4:**
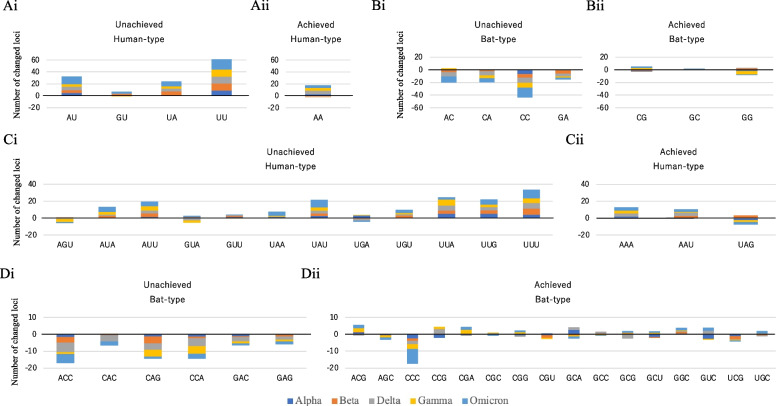
Changes observed for five variants of SARS-CoV-2. The cumulative bar chart shows the difference in the number of oligonucleotide loci per 30 kb (approximate length of the viral genome) from that of SARS-CoV-2 isolated in December 2019. The numbers of changes in alpha, beta, delta, gamma, and omicron are shown in dark blue, orange, gray, yellow, and light blue, respectively. **A** Change in the number of oligonucleotide loci for (Ai) unachieved and (Aii) achieved human-type dinucleotides. **B** Change for (Bi) unachieved and (Bii) achieved bat-type dinucleotides. **C**, **D** Change for (Ci, Di) unachieved and (Cii, Dii) achieved human- or bat-type trinucleotides, respectively

Two years have passed since the start of the SARS-CoV-2 pandemic, many mutations have accumulated in the viral genomes, and five highly infectious variants, termed variants of concern (VOC), have emerged: Alpha, Beta, Gamma, Delta, and Omicron. These five prevalent variants are considered to have adapted more successfully to the human cellular environment than other less prevalent variants. To obtain information on sufficiently accumulated mutations in these prevalent variants, the number of isolates per month was first counted for each variant, and only the genome data from the month with the highest number of isolates were used as a representative viral population that has accumulated sufficient mutations. Although we used sequences that were complete and had high coverage, there were slight differences in the length and presence of undetermined nucleotides (Ns). After normalization of the sequence length without N for each viral genome, the occurrence frequency of each focal oligonucleotide was calculated, and the average of the occurrence frequency was obtained for each variant. Its differences from the average frequency in the population at the beginning of the pandemic (CoV-2_2019) is displayed in Fig. 4. As above mentioned, Fig. 3 shows that at the beginning of the SARS-CoV-2 pandemic, the frequencies of some focal oligonucleotides were already closer to that of human-CoV than that of bat-CoV, and these oligonucleotides were specified as having already achieved adaptation to the human host, distinguishing them from unachieved oligonucleotides (Fig. 4).

Almost all unachieved human-type dinucleotides (Fig. 4Ai) increased their frequency regardless of the variant type, indicating increased adaptation to the human host. In the case of the achieved human-type dinucleotide (Fig. 4Aii), its increase level was not high, and one variant decreased. For unachieved bat-type dinucleotides (Fig. 4Bi), there was a clear tendency to reduce the frequency of these oligonucleotides and avoid their use, regardless of the variant type. In contrast, the frequencies of the achieved bat-type dinucleotides (Fig. 4Bii), and the degree of their increase or decrease, were small and varied in different directions for different variants, most likely because their frequency was sufficiently low even at the start of the outbreak. A similar trend was observed for trinucleotides, but there was also a marked increase in UUU (Fig. 4Ci) and a marked decrease in CCC (Fig. 4Dii), which seems to be an effect of APOBEC3G in human cells [21, 26, 27].

In this study, by analyzing the usage frequency of short oligonucleotides, we have analyzed the evolutionary changes of SARS-CoV-2 and focused mainly on the variant-independent changes. However, when considering the SARS-CoV-2 evolution, it is also important to study the variant-specific changes. In particular, regarding the emergence of the Omicron variant [32], peculiar evulutiobnary processes in immunosuppressed humans or even in non-human hosts are hypothesized [33, 34], and the relationship of its emergence with the specific host's cellular environment is of particular interest. When focusing on long oligonucleotides, the relationship with functions may become clearer while the types of target variables become large, and variant-specific changes can be characterized. Independently of the present study, our group have shown that variant-specific clustering, and thus variant-specific feature extraction, is possible by using an unsupervised AI suitable for analysis of a large number of variables and thus of long oligonucleotides [22, 23, 35]. By analyzing the usage of long oligonucleotides (e.g.,20-mers) in Omicron and other variants, we have recently characterized advantageous mutations that spread convergently in multiple lineages [36].

## Discussion

The oligonucleotide composition of SARS-CoV-2 at the beginning of the outbreak is thought to reflect the cellular environment of the hypothesized intermediate host [14]. Thus, some changes that have accumulated during the progression of the current pandemic may reflect, at least in part, the differences in cellular environment between the intermediate host and humans. CG-containing oligonucleotides were sufficiently low even at the outbreak start indicating that the ZAP system [37] may have been fully functional in the intermediate host. The low CG level at the beginning of the current pandemic may be one cause of the SARS-CoV-2 outbreak and seemes to be one guideline for searching for dangerous coronavirus strains that may cause a future pandemic from non-human hosts.

The marked decrease in C-rich oligonucleotides and increase in U-rich oligonucleotides after the outbreak start indicates that APOBEC3G [26, 27] may have been less functional in the intermediate host than in humans. In the present study, tetranucleotides were also analyzed, but the results are mainly shown as supplementary data. Although the general trend is similar to that of di- and trinucleotides, some cases show different trends among variants. In the case of tetranucleotides, some are functional motifs, such as binding motifs for RNA-binding proteins [14, 15]. Mutations that occur within such functional sequences may significantly alter the fitness of the mutated strain, leading to variant-specific differences.

## Conclusion

In this study, we first found that the oligonucleotide frequency in coronaviruses with host-dependent chractersitics was clearly correlated with the frequency in exon regions of the host animal. Changing the oligonucleotide frequency in the viral genome is considered as a crucial host adaptation process after changing the host, and based on this perspective, we examined the oligonucleotide frequency in SARS-CoV-2 and found that the frequencies of some oligonucleotides expected to be associated with the adaptation to humans were already close to those of human-CoV even at the beginning of the pandemic, suggesting that they had been ready to adapt to the human cell environment before the invasion of the human population; these oligonucleotides may include ZAP- and other possible host immunity-related oligonucleotide motifs. In addition, investigating the oligonucleotides that were rather closer in frequency to bat-CoV at the beginning of the pandemic revealed that their frequencies approached those of human-CoV during the course of the present pandemic, showing that SARS-CoV-2 is trying to further mimic the frequency of exon regions of humans. These oligonucleotides may also play an important role in the efficient survival of coronaviruses in the human cellular environment.

## Supplementary Information

**Additional file 1**. **Figure S1**: Level of adaptation of each tetranucleotide to the human cellular environment in SARS-CoV-2. Level of adaptation of the human- and bat-type tetranucleotides for the three virus types, human-CoV, bat-CoV, and SARS-CoV-2. This level is the ratio of the averaged oligonucleotide frequency in each virus type to the baseline, which is the midpoint between the human- and bat-CoV frequencies. The value of SARS-CoV-2 is displayed as an orange-filled circle surrounded by a black line, and that of human- or bat-CoV is displayed as a green or blue circle, respectively. **Figure S2**: Changes in human-type tetranucleotides observed for five variants of SARS-CoV-2. The cumulative bar chart shows the difference in the number of loci per 30 kb for human-type nucleotides from that of SARS-CoV-2 isolated in December 2019; the following five variants were considered. The numbers of changes of alpha, beta, delta, gamma, and omicron variants are represented by dark blue, orange, gray, yellow, and light blue, respectively. **Figure S3**: Changes in bat-type tetranucleotides in SARS-CoV-2. The cumulative bar chart shows the diffrence in the number of loci per 30 kb for bat-type tetranucleotides from that of SARS-CoV-2 isolated in December 2019, as displayed in Fig. S2.

**Additional file 2**. **Table S1**: The list of strains of human- and bat-CoV. **Table S2**: Comparison of the frequency of di-, tri-, and tetra-nucleotides between human- and bat-CoV. **Table S3**: Comparison of the frequency of di-, tri-, and tetra-nucleotides between human and bat genomes. **Table S4**: The list of oligonucleotides with significant differences in frequency between human and bat introns. **Table S5**: The list of oligonucleotides with significant differences in frequency between human and bat genomes. **Table S6**: Comparisons of the frequency of oligonucleotides between human exons and those of additional five bat species. **Table S7**: Level of adaptation of each oligonucleotide. **Table S8**: The number of isolates per month of each SARS-CoV-2 variant. **Table S9**: Changes in the oligonucleotide locus number of SARS-CoV-2.

**Additional file 3**. **Data S1**: The list of accession numbers of CoV-2_2019 used in this study. **Data S2**: The list of accession numbers of alpha variants used in this study. **Data S3**: The list of accession numbers of beta variants used in this study. **Data S4**: The list of accession numbers of delta variants used in this study. **Data S5**: The list of accession numbers of gamma variants used in this study.** Data S6**: The list of accession numbers of omicron variants used in this study.

## Data Availability

The accession numbers of all sequences analyzed in this study are listed in Additional File 2 or 3, and the corresponding sequence data are available in the GISAID database (https://www.gisaid.org/) or the NCBI virus database (https://www.ncbi.nlm.nih.gov/labs/virus/). Other numerical data generated or analyzed during this study are included either in this published article or in the additional files.

## Abbreviations

SARS-CoV-2: Severe acute respiratory syndrome coronavirus 2
Human-CoV: Human coronavirus
Bat-CoV: Bat coronavirus
CoV-2_2019: SARS-CoV-2 isolated in Dec. 2019
VOCs: Variants of concern
ZAP: Zinc-finger antiviral protein
