# Supplementary figures and images for: Oligonucleotide usage in coronavirus genomes mimics that in exon regions in host genomes
Source: Virol J. 2023 Mar 1;20:39. doi: 10.1186/s12985-023-01995-3 (PMC9976658; doi:10.1186/s12985-023-01995-3)

## Slide 1
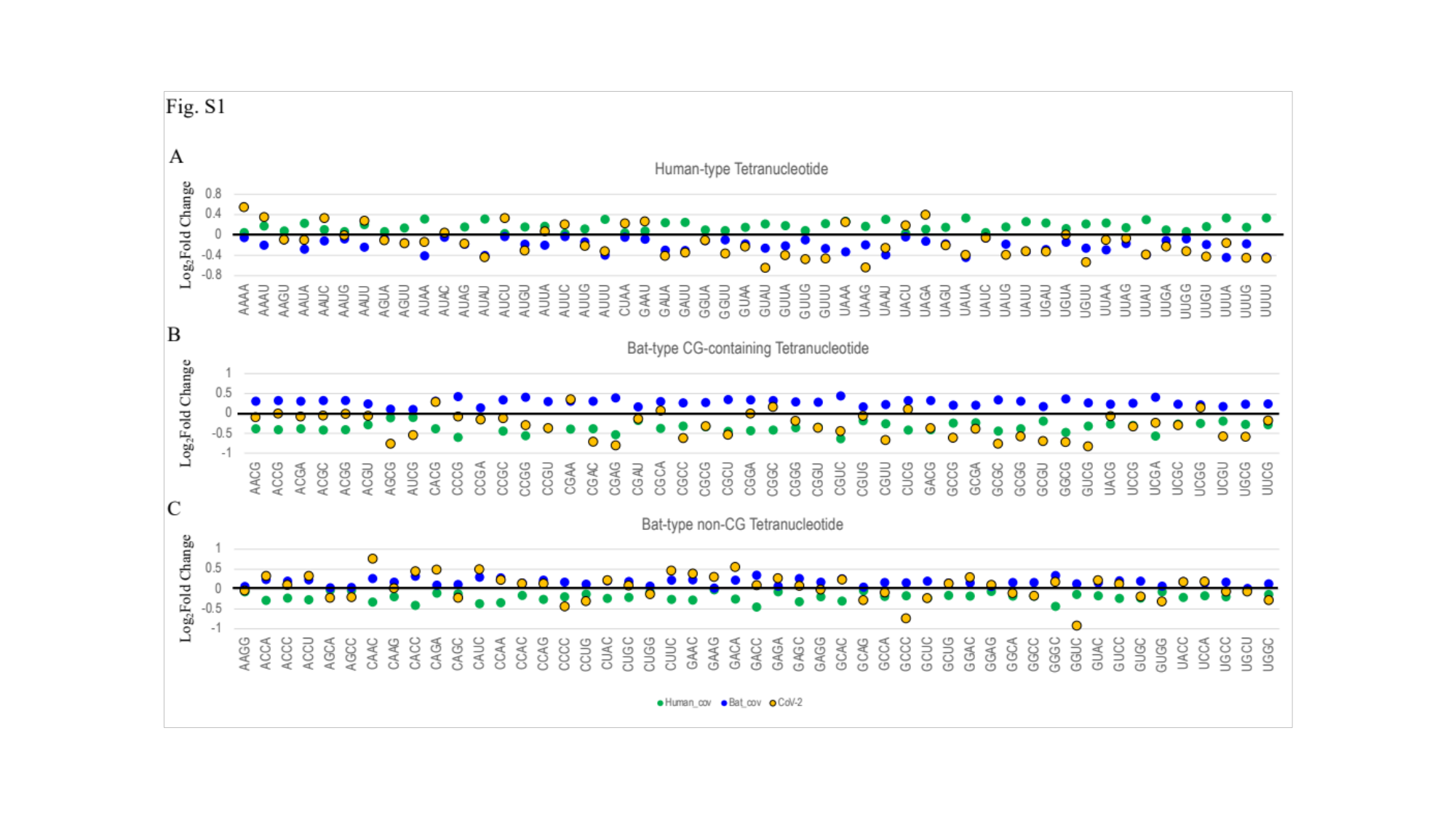

## Slide 2
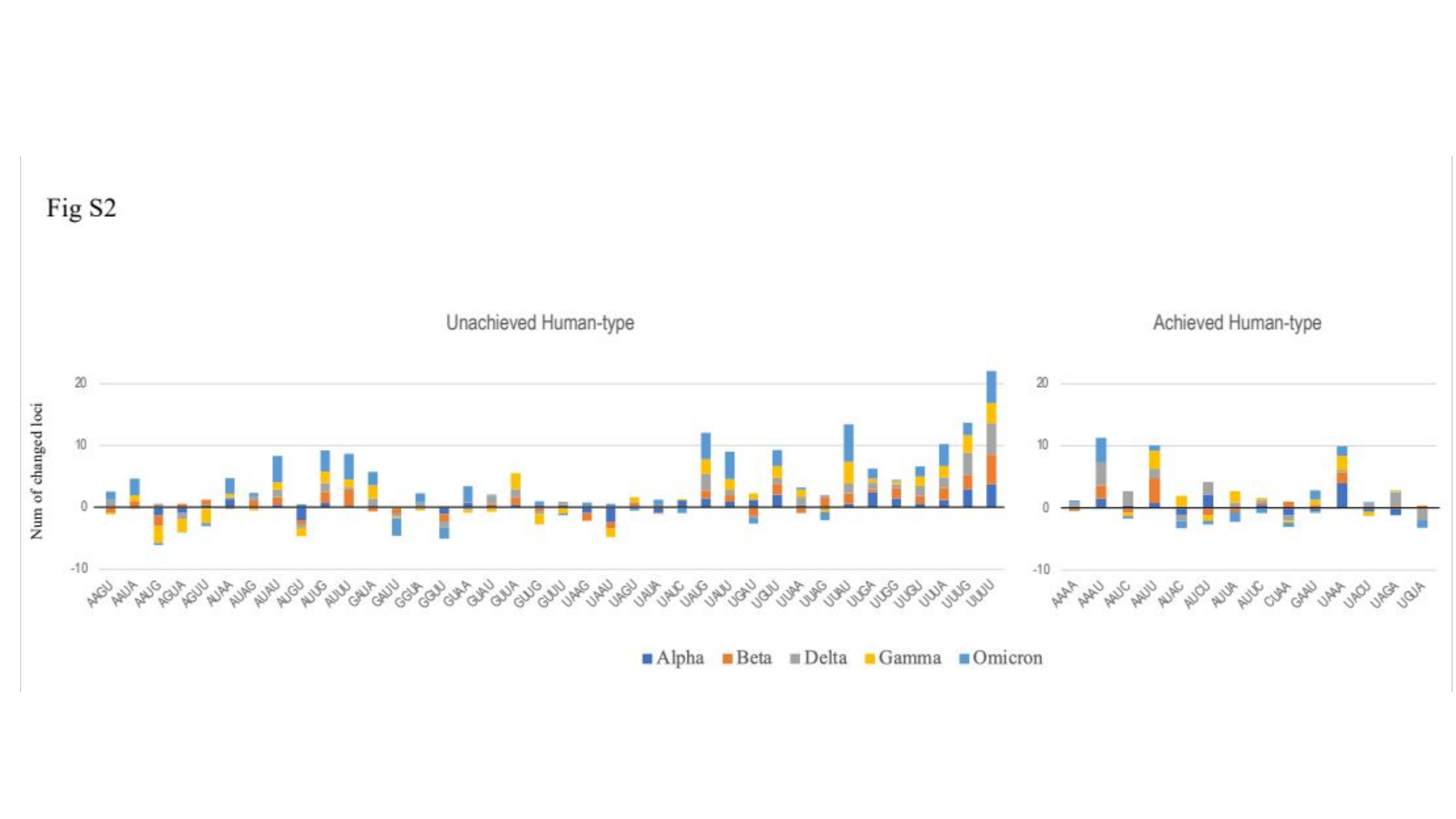

## Slide 3
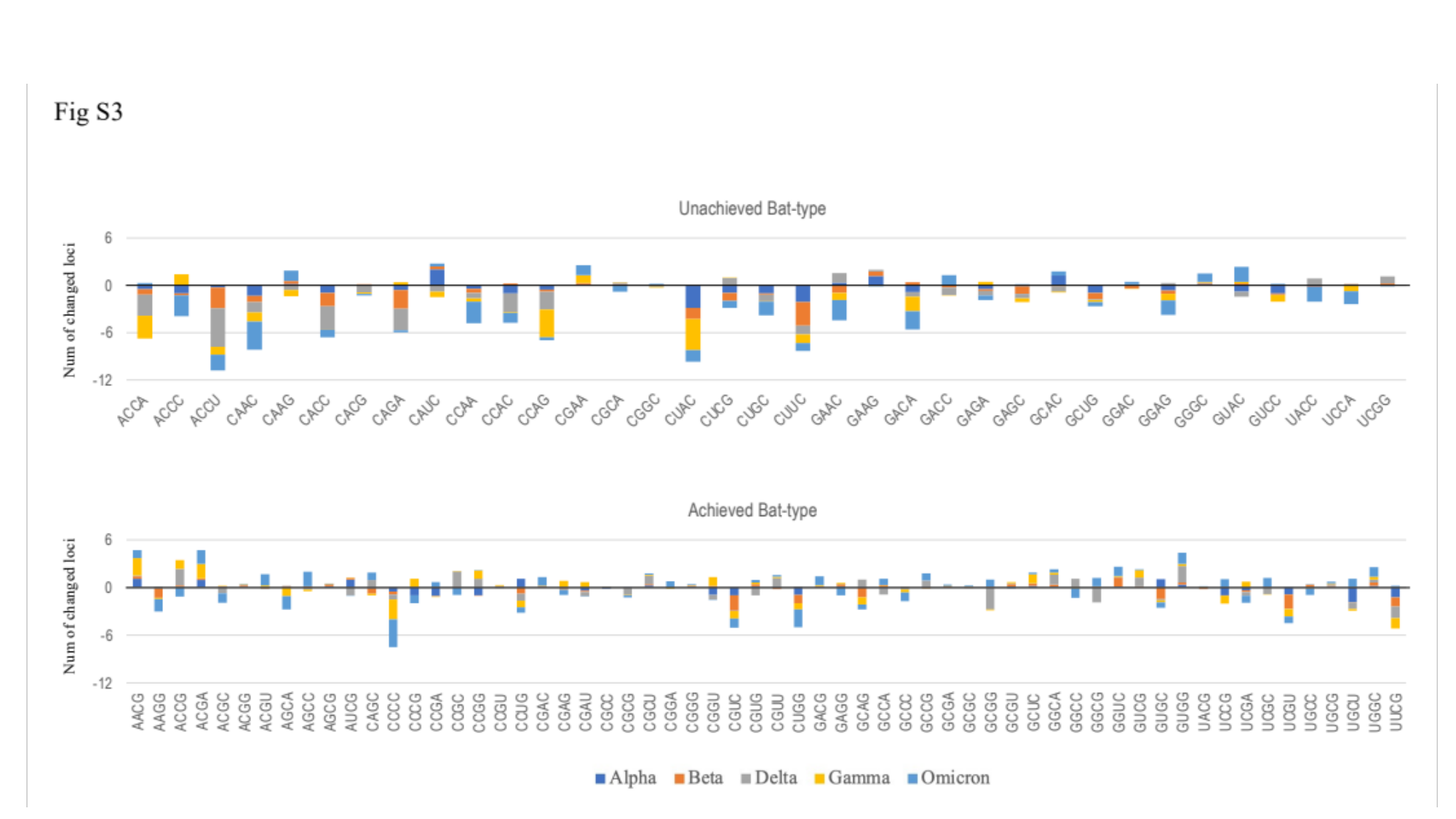

Supplement: Supplementary file 1 — Additional file 1. Figure S1: Level of adaptation of each tetranucleotide to the human cellular environment in SARS-CoV-2. Level of adaptation of the human- and bat-type tetranucleotides for the three virus types, human-CoV, bat-CoV, and SARS-CoV-2. This level is the ratio of the averaged oligonucleotide frequency in each virus type to the baseline, which is the midpoint between the human- and bat-CoV frequencies. The value of SARS-CoV-2 is displayed as an orange-filled circle surrounded by a black line, and that of human- or bat-CoV is displayed as a green or blue circle, respectively. Figure S2: Changes in human-type tetranucleotides observed for five variants of SARS-CoV-2. The cumulative bar chart shows the difference in the number of loci per 30 kb for human-type nucleotides from that of SARS-CoV-2 isolated in December 2019; the following five variants were considered. The numbers of changes of alpha, beta, delta, gamma, and omicron variants are represented by dark blue, orange, gray, yellow, and light blue, respectively. Figure S3: Changes in bat-type tetranucleotides in SARS-CoV-2. The cumulative bar chart shows the diffrence in the number of loci per 30 kb for bat-type tetranucleotides from that of SARS-CoV-2 isolated in December 2019, as displayed in Fig. S2. [file 12985_2023_1995_MOESM1_ESM.pptx]
