# Supplementary material for: Oligonucleotide usage in coronavirus genomes mimics that in exon regions in host genomes
Source: Virol J. 2023 Mar 1;20:39. doi: 10.1186/s12985-023-01995-3 (PMC9976658; doi:10.1186/s12985-023-01995-3)
Supplement: Supplementary file 3 — Additional file 3. Data S1: The list of accession numbers of CoV-2_2019 used in this study. Data S2: The list of accession numbers of alpha variants used in this study. Data S3: The list of accession numbers of beta variants used in this study. Data S4: The list of accession numbers of delta variants used in this study. Data S5: The list of accession numbers of gamma variants used in this study. Data S6: The list of accession numbers of omicron variants used in this study. [file 12985_2023_1995_MOESM3_ESM.docx]

Data S1: The list of accession numbers of CoV-2_2019 used in this study.

EPI_ISL_402119

EPI_ISL_434534

EPI_ISL_402121

EPI_ISL_406798

EPI_ISL_402125

EPI_ISL_412899

EPI_ISL_412898

EPI_ISL_403931

EPI_ISL_403929

EPI_ISL_402129

EPI_ISL_402124

EPI_ISL_529214

EPI_ISL_529215

EPI_ISL_529217

EPI_ISL_529216

EPI_ISL_529213

EPI_ISL_402123

EPI_ISL_402127

EPI_ISL_402128

EPI_ISL_402130

EPI_ISL_402132

EPI_ISL_403930

Data S2: The list of accession numbers of alpha variants used in this study.

EPI_ISL_1700219

EPI_ISL_1700222

EPI_ISL_1700223

EPI_ISL_1862992

EPI_ISL_1700224

EPI_ISL_1700225

EPI_ISL_1700226

EPI_ISL_1700228

EPI_ISL_1863416

EPI_ISL_1700229

EPI_ISL_1700230

EPI_ISL_1700231

EPI_ISL_1700232

EPI_ISL_1700233

EPI_ISL_1700234

EPI_ISL_2442721

EPI_ISL_1700235

EPI_ISL_1700237

EPI_ISL_1700238

EPI_ISL_1863411

EPI_ISL_1700239

EPI_ISL_1700240

EPI_ISL_1700241

EPI_ISL_2257773

EPI_ISL_1700242

EPI_ISL_1700243

EPI_ISL_1700245

EPI_ISL_1700246

EPI_ISL_1700247

EPI_ISL_1700248

EPI_ISL_1700249

EPI_ISL_1700250

EPI_ISL_1700251

EPI_ISL_2442734

EPI_ISL_1700252

EPI_ISL_1701385

EPI_ISL_1862758

EPI_ISL_1700253

EPI_ISL_1700255

EPI_ISL_1700256

EPI_ISL_1700259

EPI_ISL_1700260

EPI_ISL_1700261

EPI_ISL_1700263

EPI_ISL_1701386

EPI_ISL_1700264

EPI_ISL_1700266

EPI_ISL_1700267

EPI_ISL_1700268

EPI_ISL_1700269

EPI_ISL_1700270

EPI_ISL_1700271

EPI_ISL_1700272

EPI_ISL_1700273

EPI_ISL_1700274

EPI_ISL_1701389

EPI_ISL_1700275

EPI_ISL_1700276

EPI_ISL_1700278

EPI_ISL_1700279

EPI_ISL_1700281

EPI_ISL_1700283

EPI_ISL_1863427

EPI_ISL_1701390

EPI_ISL_1701392

EPI_ISL_1701393

EPI_ISL_1700284

EPI_ISL_1700285

EPI_ISL_1700286

EPI_ISL_1701395

EPI_ISL_1700287

EPI_ISL_1700289

EPI_ISL_1700292

EPI_ISL_2257767

EPI_ISL_1700295

EPI_ISL_1700297

EPI_ISL_1700298

EPI_ISL_1700299

EPI_ISL_1700301

EPI_ISL_1700302

EPI_ISL_2670368

EPI_ISL_1863300

EPI_ISL_1700303

EPI_ISL_1700304

EPI_ISL_1700306

EPI_ISL_1700307

EPI_ISL_1700310

EPI_ISL_2670372

EPI_ISL_1700311

EPI_ISL_1700312

EPI_ISL_1700314

EPI_ISL_1700316

EPI_ISL_1700317

EPI_ISL_1700318

EPI_ISL_1700319

EPI_ISL_1700320

EPI_ISL_1700321

EPI_ISL_1700323

EPI_ISL_1700324

EPI_ISL_1700326

EPI_ISL_1863302

EPI_ISL_2442778

EPI_ISL_1700327

EPI_ISL_1700328

EPI_ISL_1700329

EPI_ISL_2442779

EPI_ISL_1863549

EPI_ISL_1700332

EPI_ISL_1700334

EPI_ISL_1700335

EPI_ISL_1700336

EPI_ISL_1700337

EPI_ISL_1700338

EPI_ISL_1700339

EPI_ISL_1700340

EPI_ISL_1700341

EPI_ISL_2090303

EPI_ISL_1863308

EPI_ISL_1700342

EPI_ISL_1700343

EPI_ISL_1862968

EPI_ISL_1700344

EPI_ISL_1700346

EPI_ISL_1700347

EPI_ISL_2442790

EPI_ISL_1700348

EPI_ISL_1700349

EPI_ISL_1700350

EPI_ISL_1700351

EPI_ISL_1862969

EPI_ISL_1700353

EPI_ISL_1700354

EPI_ISL_1700355

EPI_ISL_2670403

EPI_ISL_1700357

EPI_ISL_1700358

EPI_ISL_1700359

EPI_ISL_1700360

EPI_ISL_1700361

EPI_ISL_1700362

EPI_ISL_1700363

EPI_ISL_1700366

EPI_ISL_1700367

EPI_ISL_1700368

EPI_ISL_1700369

EPI_ISL_1700370

EPI_ISL_1700371

EPI_ISL_2670416

EPI_ISL_1700372

EPI_ISL_1863233

EPI_ISL_1700373

EPI_ISL_1700375

EPI_ISL_1700376

EPI_ISL_1700377

EPI_ISL_1700378

EPI_ISL_2670425

EPI_ISL_1700379

EPI_ISL_1700380

EPI_ISL_1700381

EPI_ISL_1700382

EPI_ISL_1700383

EPI_ISL_2442814

EPI_ISL_1700384

EPI_ISL_1700385

EPI_ISL_1700386

EPI_ISL_1700387

EPI_ISL_1700388

EPI_ISL_1700389

EPI_ISL_1700391

EPI_ISL_1700392

EPI_ISL_1700393

EPI_ISL_1700394

EPI_ISL_2670430

EPI_ISL_1863235

EPI_ISL_1700395

EPI_ISL_1700396

EPI_ISL_1700397

EPI_ISL_1700399

EPI_ISL_1700400

EPI_ISL_1700401

EPI_ISL_1700404

EPI_ISL_1863592

EPI_ISL_1700406

EPI_ISL_1700407

EPI_ISL_2262557

EPI_ISL_1700408

EPI_ISL_1700410

EPI_ISL_1863473

EPI_ISL_1700411

EPI_ISL_1700412

EPI_ISL_1700415

EPI_ISL_1700416

EPI_ISL_2257744

EPI_ISL_2262561

EPI_ISL_1700417

EPI_ISL_1700418

EPI_ISL_2089956

EPI_ISL_1700419

EPI_ISL_1700420

EPI_ISL_2089955

EPI_ISL_1863590

EPI_ISL_1700421

EPI_ISL_1700423

EPI_ISL_1700424

EPI_ISL_1700425

EPI_ISL_1700427

EPI_ISL_1700428

EPI_ISL_1700430

EPI_ISL_1700432

EPI_ISL_1700433

EPI_ISL_1700434

EPI_ISL_1700435

EPI_ISL_1700436

EPI_ISL_1700437

EPI_ISL_1700438

EPI_ISL_1862859

EPI_ISL_2257748

EPI_ISL_1700439

EPI_ISL_1700440

EPI_ISL_1700441

EPI_ISL_1700443

EPI_ISL_1700444

EPI_ISL_1700445

EPI_ISL_1700446

EPI_ISL_1700447

EPI_ISL_1863246

EPI_ISL_1700448

EPI_ISL_1700450

EPI_ISL_1700451

EPI_ISL_1700452

EPI_ISL_1863247

EPI_ISL_1700453

EPI_ISL_1700456

EPI_ISL_1863483

EPI_ISL_1700457

EPI_ISL_1700460

EPI_ISL_1700461

EPI_ISL_1700462

EPI_ISL_1700464

EPI_ISL_1700466

EPI_ISL_1700467

EPI_ISL_1700469

EPI_ISL_1700470

EPI_ISL_1700472

EPI_ISL_1700474

EPI_ISL_1700475

EPI_ISL_1700476

EPI_ISL_1700477

EPI_ISL_1700478

EPI_ISL_1700479

EPI_ISL_1700480

EPI_ISL_1700483

EPI_ISL_1700487

EPI_ISL_1700488

EPI_ISL_1700489

EPI_ISL_1700490

EPI_ISL_1700491

EPI_ISL_1700492

EPI_ISL_1700495

EPI_ISL_1700496

EPI_ISL_1700497

EPI_ISL_1700498

EPI_ISL_1700499

EPI_ISL_1700502

EPI_ISL_1700503

EPI_ISL_1700504

EPI_ISL_1700505

EPI_ISL_1700506

EPI_ISL_1700507

EPI_ISL_1700510

EPI_ISL_1700511

EPI_ISL_2090167

EPI_ISL_2670573

EPI_ISL_1700590

EPI_ISL_2090162

EPI_ISL_1700591

EPI_ISL_1700593

EPI_ISL_1863364

EPI_ISL_1700605

EPI_ISL_1700606

EPI_ISL_1700607

EPI_ISL_1700609

EPI_ISL_1700610

EPI_ISL_1700611

EPI_ISL_1700612

EPI_ISL_1700613

EPI_ISL_1700618

EPI_ISL_1700619

EPI_ISL_2521752

EPI_ISL_1700620

EPI_ISL_1700621

EPI_ISL_1700622

EPI_ISL_1863579

EPI_ISL_1700627

EPI_ISL_1700628

EPI_ISL_1700629

EPI_ISL_1700632

EPI_ISL_1700633

EPI_ISL_1700634

EPI_ISL_1700635

EPI_ISL_1700636

EPI_ISL_2521791

EPI_ISL_1700638

EPI_ISL_2521794

EPI_ISL_2521796

EPI_ISL_1700642

EPI_ISL_1700643

EPI_ISL_1700645

EPI_ISL_1700646

EPI_ISL_1862673

EPI_ISL_2090075

EPI_ISL_1700652

EPI_ISL_1700654

EPI_ISL_1700655

EPI_ISL_1700656

EPI_ISL_1700657

EPI_ISL_1700658

EPI_ISL_1700659

EPI_ISL_1700661

EPI_ISL_2262577

EPI_ISL_1700662

EPI_ISL_1700663

EPI_ISL_1863574

EPI_ISL_1700664

EPI_ISL_1700665

EPI_ISL_1700666

EPI_ISL_1701315

EPI_ISL_1862790

EPI_ISL_2257960

EPI_ISL_2258013

EPI_ISL_1862885

EPI_ISL_1701316

EPI_ISL_1700700

EPI_ISL_1700703

EPI_ISL_1700704

EPI_ISL_1700711

EPI_ISL_1700712

EPI_ISL_2670906

EPI_ISL_1863456

EPI_ISL_1863572

EPI_ISL_2670624

EPI_ISL_1701335

EPI_ISL_1701351

EPI_ISL_1701353

EPI_ISL_1701354

EPI_ISL_2090454

EPI_ISL_1701356

EPI_ISL_1700766

EPI_ISL_1700767

EPI_ISL_1700768

EPI_ISL_1700769

EPI_ISL_1700770

EPI_ISL_1700772

EPI_ISL_1700774

EPI_ISL_1700775

EPI_ISL_1700776

EPI_ISL_1700777

EPI_ISL_1700778

EPI_ISL_1700779

EPI_ISL_2262590

EPI_ISL_1862889

EPI_ISL_1701357

EPI_ISL_2670659

EPI_ISL_7275801

EPI_ISL_2670660

EPI_ISL_2262594

EPI_ISL_1700842

EPI_ISL_7275831

EPI_ISL_1700844

EPI_ISL_1701358

EPI_ISL_1700845

EPI_ISL_1700846

EPI_ISL_1700847

EPI_ISL_2262595

EPI_ISL_1863223

EPI_ISL_1700848

EPI_ISL_1700849

EPI_ISL_1700850

EPI_ISL_2262597

EPI_ISL_1700851

EPI_ISL_1700852

EPI_ISL_1700853

EPI_ISL_1700854

EPI_ISL_1700855

EPI_ISL_1700856

EPI_ISL_1700857

EPI_ISL_1862655

EPI_ISL_1701359

EPI_ISL_2089570

EPI_ISL_2670692

EPI_ISL_1700859

EPI_ISL_1700860

EPI_ISL_1700862

EPI_ISL_1701360

EPI_ISL_1700864

EPI_ISL_2257824

EPI_ISL_2089454

EPI_ISL_1863460

EPI_ISL_1863582

EPI_ISL_1700865

EPI_ISL_1701361

EPI_ISL_2257828

EPI_ISL_1700876

EPI_ISL_1700877

EPI_ISL_1700879

EPI_ISL_1700880

EPI_ISL_1700881

EPI_ISL_1862895

EPI_ISL_2262604

EPI_ISL_1701362

EPI_ISL_2262609

EPI_ISL_2089542

EPI_ISL_2089662

EPI_ISL_1701364

EPI_ISL_1701365

EPI_ISL_1701366

EPI_ISL_1701367

EPI_ISL_1701368

EPI_ISL_2262645

EPI_ISL_2670816

EPI_ISL_1701370

EPI_ISL_1701371

EPI_ISL_1701372

EPI_ISL_1701373

EPI_ISL_1701374

EPI_ISL_2443122

EPI_ISL_1701375

EPI_ISL_1701376

EPI_ISL_2089560

EPI_ISL_1701377

EPI_ISL_1701378

EPI_ISL_1701379

EPI_ISL_1701380

EPI_ISL_1701381

EPI_ISL_1891974

EPI_ISL_1701383

EPI_ISL_1701425

EPI_ISL_2646962

EPI_ISL_3737721

EPI_ISL_2670964

EPI_ISL_3737727

EPI_ISL_7550940

EPI_ISL_7550955

EPI_ISL_7550956

EPI_ISL_7276411

EPI_ISL_1701667

EPI_ISL_1701668

EPI_ISL_1701673

EPI_ISL_2671028

EPI_ISL_1701674

EPI_ISL_1892500

EPI_ISL_7550968

EPI_ISL_2671033

EPI_ISL_7550971

EPI_ISL_1701716

EPI_ISL_7550985

EPI_ISL_7550986

EPI_ISL_1701790

EPI_ISL_1862186

EPI_ISL_1701792

EPI_ISL_7550989

EPI_ISL_1892504

EPI_ISL_1701804

EPI_ISL_1701810

EPI_ISL_1701811

EPI_ISL_1701821

EPI_ISL_1701824

EPI_ISL_1701836

EPI_ISL_1701845

EPI_ISL_1701847

EPI_ISL_1701848

EPI_ISL_1701852

EPI_ISL_1701853

EPI_ISL_1701856

EPI_ISL_1701857

EPI_ISL_1701858

EPI_ISL_1701865

EPI_ISL_1701866

EPI_ISL_1701883

EPI_ISL_1862164

EPI_ISL_1892511

EPI_ISL_1862178

EPI_ISL_1892513

EPI_ISL_2089526

EPI_ISL_2089631

EPI_ISL_2671133

EPI_ISL_2671138

EPI_ISL_1701953

EPI_ISL_1701955

EPI_ISL_1892517

EPI_ISL_1701956

EPI_ISL_1701958

EPI_ISL_1701959

EPI_ISL_1861904

EPI_ISL_2089876

EPI_ISL_1892521

EPI_ISL_2089518

EPI_ISL_7276637

EPI_ISL_2089517

EPI_ISL_2089629

EPI_ISL_1892526

EPI_ISL_1702123

EPI_ISL_1892530

EPI_ISL_1702147

EPI_ISL_1702150

EPI_ISL_1702155

EPI_ISL_1702158

EPI_ISL_2671216

EPI_ISL_2671243

EPI_ISL_2256344

EPI_ISL_1892537

EPI_ISL_2671262

EPI_ISL_2089713

EPI_ISL_1892542

EPI_ISL_7276911

EPI_ISL_2671336

EPI_ISL_1892547

EPI_ISL_2089904

EPI_ISL_1862622

EPI_ISL_1892549

EPI_ISL_1702540

EPI_ISL_1702541

EPI_ISL_1702544

EPI_ISL_1702545

EPI_ISL_1702549

EPI_ISL_1702552

EPI_ISL_1862624

EPI_ISL_1702553

EPI_ISL_1702555

EPI_ISL_1702557

EPI_ISL_1892551

EPI_ISL_2257607

EPI_ISL_1862620

EPI_ISL_1702569

EPI_ISL_1892553

EPI_ISL_2257711

EPI_ISL_1702581

EPI_ISL_1702583

EPI_ISL_1702585

EPI_ISL_1702586

EPI_ISL_1702587

EPI_ISL_1702588

EPI_ISL_1702589

EPI_ISL_1702590

EPI_ISL_2086784

EPI_ISL_1702593

EPI_ISL_1702594

EPI_ISL_1702595

EPI_ISL_1702596

EPI_ISL_1702597

EPI_ISL_1702599

EPI_ISL_1702600

EPI_ISL_2086821

EPI_ISL_1702603

EPI_ISL_1702604

EPI_ISL_1702606

EPI_ISL_1702607

EPI_ISL_1702608

EPI_ISL_1702609

EPI_ISL_1702610

EPI_ISL_2086822

EPI_ISL_2256322

EPI_ISL_2256325

EPI_ISL_1702617

EPI_ISL_1702618

EPI_ISL_2086814

EPI_ISL_1702956

EPI_ISL_1702625

EPI_ISL_1702626

EPI_ISL_1702627

EPI_ISL_1702629

EPI_ISL_2086851

EPI_ISL_1702631

EPI_ISL_1702633

EPI_ISL_1702634

EPI_ISL_1702640

EPI_ISL_1702642

EPI_ISL_1892564

EPI_ISL_1702655

EPI_ISL_1702656

EPI_ISL_1702657

EPI_ISL_1702658

EPI_ISL_1702660

EPI_ISL_1702662

EPI_ISL_1702665

EPI_ISL_1702668

EPI_ISL_1702670

EPI_ISL_1702672

EPI_ISL_1702673

EPI_ISL_1702674

EPI_ISL_1702676

EPI_ISL_1702677

EPI_ISL_1702680

EPI_ISL_1702682

EPI_ISL_1702685

EPI_ISL_2086842

EPI_ISL_1702687

EPI_ISL_1862602

EPI_ISL_1702692

EPI_ISL_1702693

EPI_ISL_1702694

EPI_ISL_1702697

EPI_ISL_1702699

EPI_ISL_1702700

EPI_ISL_1702702

EPI_ISL_1702704

EPI_ISL_1702705

EPI_ISL_1702707

EPI_ISL_1702710

EPI_ISL_1702711

EPI_ISL_1702713

EPI_ISL_1702717

EPI_ISL_1702718

EPI_ISL_1862612

EPI_ISL_1702810

EPI_ISL_1702811

EPI_ISL_1702812

EPI_ISL_1862614

EPI_ISL_1702817

EPI_ISL_1702818

EPI_ISL_1702960

EPI_ISL_1702823

EPI_ISL_1702968

EPI_ISL_1702826

EPI_ISL_1702827

EPI_ISL_1702831

EPI_ISL_1702832

EPI_ISL_1702836

EPI_ISL_1702838

EPI_ISL_1702842

EPI_ISL_1702852

EPI_ISL_1862426

EPI_ISL_1702866

EPI_ISL_1702880

EPI_ISL_1702883

EPI_ISL_1702884

EPI_ISL_1702892

EPI_ISL_1702894

EPI_ISL_1702895

EPI_ISL_1702897

EPI_ISL_1702902

EPI_ISL_1702903

EPI_ISL_1702904

EPI_ISL_1702908

EPI_ISL_1702913

EPI_ISL_1702914

EPI_ISL_1892588

EPI_ISL_1702922

EPI_ISL_1702923

EPI_ISL_1702928

EPI_ISL_1702931

EPI_ISL_1702939

EPI_ISL_2090658

EPI_ISL_1702975

EPI_ISL_1702979

EPI_ISL_1702981

EPI_ISL_1703029

EPI_ISL_1703030

EPI_ISL_1703037

EPI_ISL_1703039

EPI_ISL_1703047

EPI_ISL_1703055

EPI_ISL_1703056

EPI_ISL_1703057

EPI_ISL_7551385

EPI_ISL_1703064

EPI_ISL_1860953

EPI_ISL_1892592

EPI_ISL_1703071

EPI_ISL_1703072

EPI_ISL_1703079

EPI_ISL_1862552

EPI_ISL_1703080

EPI_ISL_1862310

EPI_ISL_1703085

EPI_ISL_1703086

EPI_ISL_1703101

EPI_ISL_1703102

EPI_ISL_1703103

EPI_ISL_1703104

EPI_ISL_1703105

EPI_ISL_1703106

EPI_ISL_1862559

EPI_ISL_2671896

EPI_ISL_1703112

EPI_ISL_1703113

EPI_ISL_1703114

EPI_ISL_2671897

EPI_ISL_1703118

EPI_ISL_2671898

EPI_ISL_1676454

EPI_ISL_1703123

EPI_ISL_1703124

EPI_ISL_1703127

EPI_ISL_1703129

EPI_ISL_1703133

EPI_ISL_1703134

EPI_ISL_1703135

EPI_ISL_1703136

EPI_ISL_1703138

EPI_ISL_1860764

EPI_ISL_1703145

EPI_ISL_1703146

EPI_ISL_1703147

EPI_ISL_1703155

EPI_ISL_1703156

EPI_ISL_1862521

EPI_ISL_1892609

EPI_ISL_1703164

EPI_ISL_1703165

EPI_ISL_1703166

EPI_ISL_1703168

EPI_ISL_1703174

EPI_ISL_1703186

EPI_ISL_1703197

EPI_ISL_1862528

EPI_ISL_1703207

EPI_ISL_1892613

EPI_ISL_1703229

EPI_ISL_1678310

EPI_ISL_1703245

EPI_ISL_1703255

EPI_ISL_1703256

EPI_ISL_1862588

EPI_ISL_5309954

EPI_ISL_1678268

EPI_ISL_1862225

EPI_ISL_2671961

EPI_ISL_1862463

EPI_ISL_1676562

EPI_ISL_1862464

EPI_ISL_1862586

EPI_ISL_1703351

EPI_ISL_1703352

EPI_ISL_1703358

EPI_ISL_1703360

EPI_ISL_1703361

EPI_ISL_1703368

EPI_ISL_1703369

EPI_ISL_1703370

EPI_ISL_1676555

EPI_ISL_1703379

EPI_ISL_1703380

EPI_ISL_1703381

EPI_ISL_1703390

EPI_ISL_1703391

EPI_ISL_1703392

EPI_ISL_1862582

EPI_ISL_1703400

EPI_ISL_2257412

EPI_ISL_1703401

EPI_ISL_1703408

EPI_ISL_1703409

EPI_ISL_1703411

EPI_ISL_1676437

EPI_ISL_7551545

EPI_ISL_1703417

EPI_ISL_1703419

EPI_ISL_1862341

EPI_ISL_1703422

EPI_ISL_1703424

EPI_ISL_1703425

EPI_ISL_7551552

EPI_ISL_1703426

EPI_ISL_1703427

EPI_ISL_1703428

EPI_ISL_1703429

EPI_ISL_1703430

EPI_ISL_1703431

EPI_ISL_1703432

EPI_ISL_1703433

EPI_ISL_1703435

EPI_ISL_1703436

EPI_ISL_1703437

EPI_ISL_1703438

EPI_ISL_1703439

EPI_ISL_1703440

EPI_ISL_1862359

EPI_ISL_1703442

EPI_ISL_1703444

EPI_ISL_1703445

EPI_ISL_1703446

EPI_ISL_1703447

EPI_ISL_1703448

EPI_ISL_1703449

EPI_ISL_1703450

EPI_ISL_1703452

EPI_ISL_1703453

EPI_ISL_1703454

EPI_ISL_1703456

EPI_ISL_1703457

EPI_ISL_1703458

EPI_ISL_1703460

EPI_ISL_1703461

EPI_ISL_1703462

EPI_ISL_1703463

EPI_ISL_1703464

EPI_ISL_1703465

EPI_ISL_1703466

EPI_ISL_1703471

EPI_ISL_1703472

EPI_ISL_1703473

EPI_ISL_1703474

EPI_ISL_1703475

EPI_ISL_1703476

EPI_ISL_1703477

EPI_ISL_1703478

EPI_ISL_1678240

EPI_ISL_1703479

EPI_ISL_1703480

EPI_ISL_1703481

EPI_ISL_1703482

EPI_ISL_1862475

EPI_ISL_1703484

EPI_ISL_1703485

EPI_ISL_1703486

EPI_ISL_1703487

EPI_ISL_2257418

EPI_ISL_1703488

EPI_ISL_1703489

EPI_ISL_1703490

EPI_ISL_1703491

EPI_ISL_1703492

EPI_ISL_1703493

EPI_ISL_1703494

EPI_ISL_1703495

EPI_ISL_1703497

EPI_ISL_1703498

EPI_ISL_1703499

EPI_ISL_1703500

EPI_ISL_1703501

EPI_ISL_1703502

EPI_ISL_1703503

EPI_ISL_1703504

EPI_ISL_1703505

EPI_ISL_1703506

EPI_ISL_1703507

EPI_ISL_1703509

EPI_ISL_1703510

EPI_ISL_1703511

EPI_ISL_1703512

EPI_ISL_1703513

EPI_ISL_1703514

EPI_ISL_2442313

EPI_ISL_1703520

EPI_ISL_1703521

EPI_ISL_1703522

EPI_ISL_1703523

EPI_ISL_1703524

EPI_ISL_1703525

EPI_ISL_1703526

EPI_ISL_1703527

EPI_ISL_1703528

EPI_ISL_1703531

EPI_ISL_1703532

EPI_ISL_1703533

EPI_ISL_1703536

EPI_ISL_1678242

EPI_ISL_1703538

EPI_ISL_1703542

EPI_ISL_1862592

EPI_ISL_1703543

EPI_ISL_1703545

EPI_ISL_1703546

EPI_ISL_1703547

EPI_ISL_1703549

EPI_ISL_1703553

EPI_ISL_1703555

EPI_ISL_1703556

EPI_ISL_1703557

EPI_ISL_1703563

EPI_ISL_1703564

EPI_ISL_1703565

EPI_ISL_1703566

EPI_ISL_1703573

EPI_ISL_1703574

EPI_ISL_1703575

EPI_ISL_1703576

EPI_ISL_1703577

EPI_ISL_1703579

EPI_ISL_1703584

EPI_ISL_1703585

EPI_ISL_1703586

EPI_ISL_1703587

EPI_ISL_1703588

EPI_ISL_1703589

EPI_ISL_1703590

EPI_ISL_1703594

EPI_ISL_1703595

EPI_ISL_1703596

EPI_ISL_1703597

EPI_ISL_1703598

EPI_ISL_1703599

EPI_ISL_1703600

EPI_ISL_1703601

EPI_ISL_1703602

EPI_ISL_1703603

EPI_ISL_1703604

EPI_ISL_1703607

EPI_ISL_1703608

EPI_ISL_1703609

EPI_ISL_1703610

EPI_ISL_1703611

EPI_ISL_1703612

EPI_ISL_1703613

EPI_ISL_1703614

EPI_ISL_1703618

EPI_ISL_1703619

EPI_ISL_1703621

EPI_ISL_1703622

EPI_ISL_1703623

EPI_ISL_1703624

EPI_ISL_1703625

EPI_ISL_1703629

EPI_ISL_1703630

EPI_ISL_1703631

EPI_ISL_1703632

EPI_ISL_1703633

EPI_ISL_1703634

EPI_ISL_1862445

EPI_ISL_1862568

EPI_ISL_1861226

EPI_ISL_7551667

EPI_ISL_1676535

EPI_ISL_1676409

EPI_ISL_7551678

EPI_ISL_1862565

EPI_ISL_1676407

EPI_ISL_7551688

EPI_ISL_7551689

EPI_ISL_1862337

EPI_ISL_1862458

EPI_ISL_1703849

EPI_ISL_1862217

EPI_ISL_1703857

EPI_ISL_1703867

EPI_ISL_1703874

EPI_ISL_1703885

EPI_ISL_2091003

EPI_ISL_1703897

EPI_ISL_1703905

EPI_ISL_1703919

EPI_ISL_1862339

EPI_ISL_7551734

EPI_ISL_7551735

EPI_ISL_1676512

EPI_ISL_1703926

EPI_ISL_1703936

EPI_ISL_1703947

EPI_ISL_1703958

EPI_ISL_1703970

EPI_ISL_1862145

EPI_ISL_1703991

EPI_ISL_1703998

EPI_ISL_1704010

EPI_ISL_1861029

EPI_ISL_1704023

EPI_ISL_7551766

EPI_ISL_1862143

EPI_ISL_1704031

EPI_ISL_1704041

EPI_ISL_1704051

EPI_ISL_1704059

EPI_ISL_1862157

EPI_ISL_1862398

EPI_ISL_1704068

EPI_ISL_1704075

EPI_ISL_1704098

EPI_ISL_1704112

EPI_ISL_1861115

EPI_ISL_1704124

EPI_ISL_1862396

EPI_ISL_1704136

EPI_ISL_1704147

EPI_ISL_7551814

EPI_ISL_1862270

EPI_ISL_1704160

EPI_ISL_2091070

EPI_ISL_2256246

EPI_ISL_1704171

EPI_ISL_1862243

EPI_ISL_1704192

EPI_ISL_1704204

EPI_ISL_1704216

EPI_ISL_1704228

EPI_ISL_2091129

EPI_ISL_1704239

EPI_ISL_1704248

EPI_ISL_1704260

EPI_ISL_1704273

EPI_ISL_1862480

EPI_ISL_1704286

EPI_ISL_1704296

EPI_ISL_1704305

EPI_ISL_1862496

EPI_ISL_1704317

EPI_ISL_1862497

EPI_ISL_1861170

EPI_ISL_1862377

EPI_ISL_1862498

EPI_ISL_1862493

EPI_ISL_1705353

EPI_ISL_2822964

EPI_ISL_1705354

EPI_ISL_6159114

EPI_ISL_1860737

EPI_ISL_1705360

EPI_ISL_1705361

EPI_ISL_1705362

EPI_ISL_2257455

EPI_ISL_1705363

EPI_ISL_1860400

EPI_ISL_1860648

EPI_ISL_1860647

EPI_ISL_1860526

EPI_ISL_1860646

EPI_ISL_1704686

EPI_ISL_1704687

EPI_ISL_1704689

EPI_ISL_1704692

EPI_ISL_1704694

EPI_ISL_1704696

EPI_ISL_1704699

EPI_ISL_1859934

EPI_ISL_1704701

EPI_ISL_1704703

EPI_ISL_1704704

EPI_ISL_1704706

EPI_ISL_1704707

EPI_ISL_1704709

EPI_ISL_1704710

EPI_ISL_1704711

EPI_ISL_1704713

EPI_ISL_1705364

EPI_ISL_1705367

EPI_ISL_1704734

EPI_ISL_1859935

EPI_ISL_1860528

EPI_ISL_1704736

EPI_ISL_1704737

EPI_ISL_1704739

EPI_ISL_1704742

EPI_ISL_1704743

EPI_ISL_1704745

EPI_ISL_1704746

EPI_ISL_1704747

EPI_ISL_1704748

EPI_ISL_1704749

EPI_ISL_1704754

EPI_ISL_1704755

EPI_ISL_1704757

EPI_ISL_1704762

EPI_ISL_1704796

EPI_ISL_1704797

EPI_ISL_1704801

EPI_ISL_1704802

EPI_ISL_1704803

EPI_ISL_1704804

EPI_ISL_1704806

EPI_ISL_1704807

EPI_ISL_4372312

EPI_ISL_1704825

EPI_ISL_1704826

EPI_ISL_1704827

EPI_ISL_1860537

EPI_ISL_1704852

EPI_ISL_1704854

EPI_ISL_1704857

EPI_ISL_1704860

EPI_ISL_1704861

EPI_ISL_1704862

EPI_ISL_1704863

EPI_ISL_1704864

EPI_ISL_1704865

EPI_ISL_1704866

EPI_ISL_1704870

EPI_ISL_1704872

EPI_ISL_1704873

EPI_ISL_1704874

EPI_ISL_1704875

EPI_ISL_1704876

EPI_ISL_1704877

EPI_ISL_1704878

EPI_ISL_1704881

EPI_ISL_1704882

EPI_ISL_1704883

EPI_ISL_1704884

EPI_ISL_1704885

EPI_ISL_1704886

EPI_ISL_1704887

EPI_ISL_1704889

EPI_ISL_1704890

EPI_ISL_1704893

EPI_ISL_1704894

EPI_ISL_1704895

EPI_ISL_1704896

EPI_ISL_1704897

EPI_ISL_1704898

EPI_ISL_1704899

EPI_ISL_1704900

EPI_ISL_1704909

EPI_ISL_1704911

EPI_ISL_1704912

EPI_ISL_1704913

EPI_ISL_1704921

EPI_ISL_1704923

EPI_ISL_1704924

EPI_ISL_1704925

EPI_ISL_1704926

EPI_ISL_1704927

EPI_ISL_1704933

EPI_ISL_1704934

EPI_ISL_1704954

EPI_ISL_1704956

EPI_ISL_1704958

EPI_ISL_1704959

EPI_ISL_1704965

EPI_ISL_1704966

EPI_ISL_1704967

EPI_ISL_1704968

EPI_ISL_1860747

EPI_ISL_1704970

EPI_ISL_1704971

EPI_ISL_1704976

EPI_ISL_1704977

EPI_ISL_1704978

EPI_ISL_1704979

EPI_ISL_1704981

EPI_ISL_1704982

EPI_ISL_1859922

EPI_ISL_1704988

EPI_ISL_1704990

EPI_ISL_1704992

EPI_ISL_1704993

EPI_ISL_1704994

EPI_ISL_1704995

EPI_ISL_1704997

EPI_ISL_1704998

EPI_ISL_1704999

EPI_ISL_1705003

EPI_ISL_1705004

EPI_ISL_1705005

EPI_ISL_1705007

EPI_ISL_1705008

EPI_ISL_1705009

EPI_ISL_1860623

EPI_ISL_5314406

EPI_ISL_5314411

EPI_ISL_5314417

EPI_ISL_1705012

EPI_ISL_1705014

EPI_ISL_5314437

EPI_ISL_1705020

EPI_ISL_1705021

EPI_ISL_1705022

EPI_ISL_1705023

EPI_ISL_1705025

EPI_ISL_1705026

EPI_ISL_1705030

EPI_ISL_1705031

EPI_ISL_1705032

EPI_ISL_1705033

EPI_ISL_1705034

EPI_ISL_1705035

EPI_ISL_1705036

EPI_ISL_2091419

EPI_ISL_1705040

EPI_ISL_1705041

EPI_ISL_1860749

EPI_ISL_1705043

EPI_ISL_1705044

EPI_ISL_1705045

EPI_ISL_1705046

EPI_ISL_1705051

EPI_ISL_1860507

EPI_ISL_1705052

EPI_ISL_1705054

EPI_ISL_1705057

EPI_ISL_1705058

EPI_ISL_1705059

EPI_ISL_1860750

EPI_ISL_1705060

EPI_ISL_1705061

EPI_ISL_1705063

EPI_ISL_1705067

EPI_ISL_1705068

EPI_ISL_1705071

EPI_ISL_1705072

EPI_ISL_1705073

EPI_ISL_1705081

EPI_ISL_1860633

EPI_ISL_1705082

EPI_ISL_1705090

EPI_ISL_1705091

EPI_ISL_1859976

EPI_ISL_1705098

EPI_ISL_1678855

EPI_ISL_1705100

EPI_ISL_1705101

EPI_ISL_1705108

EPI_ISL_1705110

EPI_ISL_1860631

EPI_ISL_1705118

EPI_ISL_1860510

EPI_ISL_1705119

EPI_ISL_1860630

EPI_ISL_1705127

EPI_ISL_1705128

EPI_ISL_1705133

EPI_ISL_1705136

EPI_ISL_1705137

EPI_ISL_1705138

EPI_ISL_1705144

EPI_ISL_1705147

EPI_ISL_1705148

EPI_ISL_1705149

EPI_ISL_1705150

EPI_ISL_1705152

EPI_ISL_1705153

EPI_ISL_1705154

EPI_ISL_1705155

EPI_ISL_1705159

EPI_ISL_1705161

EPI_ISL_1705162

EPI_ISL_1705163

EPI_ISL_1705164

EPI_ISL_1705166

EPI_ISL_1705167

EPI_ISL_1705170

EPI_ISL_1705171

EPI_ISL_1705172

EPI_ISL_1705173

EPI_ISL_1705174

EPI_ISL_1705177

EPI_ISL_1705181

EPI_ISL_1705182

EPI_ISL_1705183

EPI_ISL_1859983

EPI_ISL_1860684

EPI_ISL_1705185

EPI_ISL_1705186

EPI_ISL_1705187

EPI_ISL_1705188

EPI_ISL_1705189

EPI_ISL_1705193

EPI_ISL_1705194

EPI_ISL_1705195

EPI_ISL_1705198

EPI_ISL_1705199

EPI_ISL_1705200

EPI_ISL_1705201

EPI_ISL_1705202

EPI_ISL_1705204

EPI_ISL_1705205

EPI_ISL_1705206

EPI_ISL_1705207

EPI_ISL_1705208

EPI_ISL_1705209

EPI_ISL_1705210

EPI_ISL_1705211

EPI_ISL_1705212

EPI_ISL_1705215

EPI_ISL_1705216

EPI_ISL_1705217

EPI_ISL_1705218

EPI_ISL_1705219

EPI_ISL_1705220

EPI_ISL_1705222

EPI_ISL_1705225

EPI_ISL_1705227

EPI_ISL_1705228

EPI_ISL_1705229

EPI_ISL_1705230

EPI_ISL_1705232

EPI_ISL_1705235

EPI_ISL_1705236

EPI_ISL_1705237

EPI_ISL_1705239

EPI_ISL_1705242

EPI_ISL_1705243

EPI_ISL_1705244

EPI_ISL_1705245

EPI_ISL_1705246

EPI_ISL_1705247

EPI_ISL_1705250

EPI_ISL_1859986

EPI_ISL_1705251

EPI_ISL_1705252

EPI_ISL_1705253

EPI_ISL_1705254

EPI_ISL_1705255

EPI_ISL_1705256

EPI_ISL_1705257

EPI_ISL_1705258

EPI_ISL_1705259

EPI_ISL_1705260

EPI_ISL_1705261

EPI_ISL_1705262

EPI_ISL_1705263

EPI_ISL_1705264

EPI_ISL_1705265

EPI_ISL_1705266

EPI_ISL_1705267

EPI_ISL_1705268

EPI_ISL_1705269

EPI_ISL_1705270

EPI_ISL_1705271

EPI_ISL_1705272

EPI_ISL_1705274

EPI_ISL_1705275

EPI_ISL_1705278

EPI_ISL_1705280

EPI_ISL_1705282

EPI_ISL_1705283

EPI_ISL_1705284

EPI_ISL_1705285

EPI_ISL_1705286

EPI_ISL_7040765

EPI_ISL_1859950

EPI_ISL_1705287

EPI_ISL_1705288

EPI_ISL_1705290

EPI_ISL_1705291

EPI_ISL_1705292

EPI_ISL_1705294

EPI_ISL_1705295

EPI_ISL_1705297

EPI_ISL_1705298

EPI_ISL_1705299

EPI_ISL_1705300

EPI_ISL_1705301

EPI_ISL_1705302

EPI_ISL_1705303

EPI_ISL_1705304

EPI_ISL_5843943

EPI_ISL_1705305

EPI_ISL_1705306

EPI_ISL_1705307

EPI_ISL_1705308

EPI_ISL_1860444

EPI_ISL_1705312

EPI_ISL_1705313

EPI_ISL_1705315

EPI_ISL_1705316

EPI_ISL_1705317

EPI_ISL_1705318

EPI_ISL_1705320

EPI_ISL_1705323

EPI_ISL_1705324

EPI_ISL_1705325

EPI_ISL_1705326

EPI_ISL_1705327

EPI_ISL_1705329

EPI_ISL_1705330

EPI_ISL_1859956

EPI_ISL_1705337

EPI_ISL_1705338

EPI_ISL_1705339

EPI_ISL_5315403

EPI_ISL_1574403

EPI_ISL_1574402

EPI_ISL_1705340

EPI_ISL_1574408

EPI_ISL_1705341

EPI_ISL_1705342

EPI_ISL_1705343

EPI_ISL_1574507

EPI_ISL_1574501

EPI_ISL_7041060

EPI_ISL_1705348

EPI_ISL_1705349

EPI_ISL_1705350

EPI_ISL_1705351

EPI_ISL_1705352

EPI_ISL_1572812

EPI_ISL_1705371

EPI_ISL_1705372

EPI_ISL_1705373

EPI_ISL_2686290

EPI_ISL_1705374

EPI_ISL_5315456

EPI_ISL_1705375

EPI_ISL_5315469

EPI_ISL_1705377

EPI_ISL_1705427

EPI_ISL_2709154

EPI_ISL_1741463

EPI_ISL_1705383

EPI_ISL_1741472

EPI_ISL_1705384

EPI_ISL_1705386

EPI_ISL_1705387

EPI_ISL_5315940

EPI_ISL_1705389

EPI_ISL_5316021

EPI_ISL_1741284

EPI_ISL_5316039

EPI_ISL_5316056

EPI_ISL_5316063

EPI_ISL_5316068

EPI_ISL_5316083

EPI_ISL_5316084

EPI_ISL_5316086

EPI_ISL_5316087

EPI_ISL_5316088

EPI_ISL_5316089

EPI_ISL_5316090

EPI_ISL_5316091

EPI_ISL_5316092

EPI_ISL_5316093

EPI_ISL_5316094

EPI_ISL_5316096

EPI_ISL_5316099

EPI_ISL_5316101

EPI_ISL_5316103

EPI_ISL_5316104

EPI_ISL_5316105

EPI_ISL_5316106

EPI_ISL_5316108

EPI_ISL_5316109

EPI_ISL_5316110

EPI_ISL_1573341

EPI_ISL_5316111

EPI_ISL_5316113

EPI_ISL_5316114

EPI_ISL_5316115

EPI_ISL_5316129

EPI_ISL_5316130

EPI_ISL_5316131

EPI_ISL_5316133

EPI_ISL_1572868

EPI_ISL_5316149

EPI_ISL_1573353

EPI_ISL_1572708

EPI_ISL_1705394

EPI_ISL_1705395

EPI_ISL_1705396

EPI_ISL_1705397

EPI_ISL_1572952

EPI_ISL_1705403

EPI_ISL_5316256

EPI_ISL_5316307

EPI_ISL_2328325

EPI_ISL_2687641

EPI_ISL_1705404

EPI_ISL_2328256

EPI_ISL_1705405

EPI_ISL_1572722

EPI_ISL_2328275

EPI_ISL_1705406

EPI_ISL_1705408

EPI_ISL_2328323

EPI_ISL_2328346

EPI_ISL_1573216

EPI_ISL_1705410

EPI_ISL_2328382

EPI_ISL_1705412

EPI_ISL_2688485

EPI_ISL_1705415

EPI_ISL_4470922

EPI_ISL_1705417

EPI_ISL_1705418

EPI_ISL_1705419

EPI_ISL_5317339

EPI_ISL_1705423

EPI_ISL_1705425

EPI_ISL_2328457

EPI_ISL_1705426

EPI_ISL_1705429

EPI_ISL_1705436

EPI_ISL_1705437

EPI_ISL_1705439

EPI_ISL_1705440

EPI_ISL_1705441

EPI_ISL_2150676

EPI_ISL_1705447

EPI_ISL_1705448

EPI_ISL_1705449

EPI_ISL_1705450

EPI_ISL_1705457

EPI_ISL_1705458

EPI_ISL_1705459

EPI_ISL_1705461

EPI_ISL_1705462

EPI_ISL_1705468

EPI_ISL_1705469

EPI_ISL_1705478

EPI_ISL_1705479

EPI_ISL_1705481

EPI_ISL_1705482

EPI_ISL_1705483

EPI_ISL_2690137

EPI_ISL_1705494

EPI_ISL_1705495

EPI_ISL_1705496

EPI_ISL_1705497

EPI_ISL_1705498

EPI_ISL_1705504

EPI_ISL_1705505

EPI_ISL_1705506

EPI_ISL_1705507

EPI_ISL_1705508

EPI_ISL_1705509

EPI_ISL_1705511

EPI_ISL_1705514

EPI_ISL_1705515

EPI_ISL_1705518

EPI_ISL_1705519

EPI_ISL_1705526

EPI_ISL_1705527

EPI_ISL_1705531

EPI_ISL_1705532

EPI_ISL_1705533

EPI_ISL_1705537

EPI_ISL_1705540

EPI_ISL_1705541

EPI_ISL_1705542

EPI_ISL_1705550

EPI_ISL_1705551

EPI_ISL_1705553

EPI_ISL_1705557

EPI_ISL_1705562

EPI_ISL_1705563

EPI_ISL_1705564

EPI_ISL_1705668

EPI_ISL_1705567

EPI_ISL_1705568

EPI_ISL_1705569

EPI_ISL_1705570

EPI_ISL_1705572

EPI_ISL_1705573

EPI_ISL_1705575

EPI_ISL_1705576

EPI_ISL_1705577

EPI_ISL_1705578

EPI_ISL_1705579

EPI_ISL_1705580

EPI_ISL_1705581

EPI_ISL_1705582

EPI_ISL_1705583

EPI_ISL_1705585

EPI_ISL_1705586

EPI_ISL_1705589

EPI_ISL_1705590

EPI_ISL_1705591

EPI_ISL_1705592

EPI_ISL_1705593

EPI_ISL_1705594

EPI_ISL_1705595

EPI_ISL_1705597

EPI_ISL_1705598

EPI_ISL_1705599

EPI_ISL_1705600

EPI_ISL_1705601

EPI_ISL_1705602

EPI_ISL_1705603

EPI_ISL_1705606

EPI_ISL_1705671

EPI_ISL_1705607

EPI_ISL_1705608

EPI_ISL_1705672

EPI_ISL_1705609

EPI_ISL_1705610

EPI_ISL_1705611

EPI_ISL_1705614

EPI_ISL_1573314

EPI_ISL_1705615

EPI_ISL_1573315

EPI_ISL_1705617

EPI_ISL_1705618

EPI_ISL_1572882

EPI_ISL_1705619

EPI_ISL_1705620

EPI_ISL_1705621

EPI_ISL_1573401

EPI_ISL_1705622

EPI_ISL_1573406

EPI_ISL_1705623

EPI_ISL_1705624

EPI_ISL_1705628

EPI_ISL_1705629

EPI_ISL_1572768

EPI_ISL_1705630

EPI_ISL_1705631

EPI_ISL_1705632

EPI_ISL_1572694

EPI_ISL_1705633

EPI_ISL_2672777

EPI_ISL_1705634

EPI_ISL_1705636

EPI_ISL_1705637

EPI_ISL_1572796

EPI_ISL_1705638

EPI_ISL_1705639

EPI_ISL_1705640

EPI_ISL_1705641

EPI_ISL_1705642

EPI_ISL_1705644

EPI_ISL_1705645

EPI_ISL_1705646

EPI_ISL_1705649

EPI_ISL_1705650

EPI_ISL_2328915

EPI_ISL_1705652

EPI_ISL_1705653

EPI_ISL_1705654

EPI_ISL_1705655

EPI_ISL_1705656

EPI_ISL_1705657

EPI_ISL_1705658

EPI_ISL_2328968

EPI_ISL_1705660

EPI_ISL_1705661

EPI_ISL_1705662

EPI_ISL_1705663

EPI_ISL_1705664

EPI_ISL_1705674

EPI_ISL_1705675

EPI_ISL_1705680

EPI_ISL_1705683

EPI_ISL_1705687

EPI_ISL_1705692

EPI_ISL_1705693

EPI_ISL_1705694

EPI_ISL_1705695

EPI_ISL_7672053

EPI_ISL_1705696

EPI_ISL_1705698

EPI_ISL_3473420

EPI_ISL_3473428

EPI_ISL_3473429

EPI_ISL_3473433

EPI_ISL_3473434

EPI_ISL_3473435

EPI_ISL_1705702

EPI_ISL_1705703

EPI_ISL_1705704

EPI_ISL_1705705

EPI_ISL_1705708

EPI_ISL_1705709

EPI_ISL_1705710

EPI_ISL_2672834

EPI_ISL_1705715

EPI_ISL_7686165

EPI_ISL_1705716

EPI_ISL_1705717

EPI_ISL_1705719

EPI_ISL_1705720

EPI_ISL_1705721

EPI_ISL_1705722

EPI_ISL_1919798

EPI_ISL_1705785

EPI_ISL_1705727

EPI_ISL_1705728

EPI_ISL_1705729

EPI_ISL_1705730

EPI_ISL_1705731

EPI_ISL_1705732

EPI_ISL_1705733

EPI_ISL_1705738

EPI_ISL_1705739

EPI_ISL_1705740

EPI_ISL_1705741

EPI_ISL_1705742

EPI_ISL_1705743

EPI_ISL_1705744

EPI_ISL_1705745

EPI_ISL_1705786

EPI_ISL_1705746

EPI_ISL_1705750

EPI_ISL_1705751

EPI_ISL_1705752

EPI_ISL_1705753

EPI_ISL_1705754

EPI_ISL_1705755

EPI_ISL_1705756

EPI_ISL_1705757

EPI_ISL_1705759

EPI_ISL_1919942

EPI_ISL_1705761

EPI_ISL_1705762

EPI_ISL_3474499

EPI_ISL_3474502

EPI_ISL_3474504

EPI_ISL_3474511

EPI_ISL_3474512

EPI_ISL_3474513

EPI_ISL_1744357

EPI_ISL_3474516

EPI_ISL_3474517

EPI_ISL_3474519

EPI_ISL_3474521

EPI_ISL_3474526

EPI_ISL_1705763

EPI_ISL_3474550

EPI_ISL_3474553

EPI_ISL_3474556

EPI_ISL_3474557

EPI_ISL_3474558

EPI_ISL_3474559

EPI_ISL_3474561

EPI_ISL_3474562

EPI_ISL_3474572

EPI_ISL_3474603

EPI_ISL_3474604

EPI_ISL_3474605

EPI_ISL_3474606

EPI_ISL_2329815

EPI_ISL_3474615

EPI_ISL_3474616

EPI_ISL_3474619

EPI_ISL_3474620

EPI_ISL_3474621

EPI_ISL_3474632

EPI_ISL_1705764

EPI_ISL_3474633

EPI_ISL_3474638

EPI_ISL_1744382

EPI_ISL_1705765

EPI_ISL_3474674

EPI_ISL_1705766

EPI_ISL_1705767

EPI_ISL_2329838

EPI_ISL_3474691

EPI_ISL_3474692

EPI_ISL_1705768

EPI_ISL_3474699

EPI_ISL_3474700

EPI_ISL_3474723

EPI_ISL_3474724

EPI_ISL_1705769

EPI_ISL_1705770

EPI_ISL_1705771

EPI_ISL_1705772

EPI_ISL_1705773

EPI_ISL_1705774

EPI_ISL_1705775

EPI_ISL_1705776

EPI_ISL_1705777

EPI_ISL_3474833

EPI_ISL_3474834

EPI_ISL_1705778

EPI_ISL_1705780

EPI_ISL_1744473

EPI_ISL_1705781

EPI_ISL_1705782

EPI_ISL_1705784

EPI_ISL_1705787

EPI_ISL_1705788

EPI_ISL_1705789

EPI_ISL_1705790

EPI_ISL_1705791

EPI_ISL_1705792

EPI_ISL_1705793

EPI_ISL_1705794

EPI_ISL_1705795

EPI_ISL_1705796

EPI_ISL_1705798

EPI_ISL_1705799

EPI_ISL_3475487

EPI_ISL_1705800

EPI_ISL_1705802

EPI_ISL_1705803

EPI_ISL_1705804

EPI_ISL_1705806

EPI_ISL_1705809

EPI_ISL_1705810

EPI_ISL_1705965

EPI_ISL_1705814

EPI_ISL_1705815

EPI_ISL_1705816

EPI_ISL_1705817

EPI_ISL_1705819

EPI_ISL_1705820

EPI_ISL_1705821

EPI_ISL_1705822

EPI_ISL_1705823

EPI_ISL_1705828

EPI_ISL_1705830

EPI_ISL_1705831

EPI_ISL_1705832

EPI_ISL_1705833

EPI_ISL_1705834

EPI_ISL_1920224

EPI_ISL_1705837

EPI_ISL_1705839

EPI_ISL_1705840

EPI_ISL_1705841

EPI_ISL_1705842

EPI_ISL_1705843

EPI_ISL_1705844

EPI_ISL_1705845

EPI_ISL_1705846

EPI_ISL_1705847

EPI_ISL_1705966

EPI_ISL_1705848

EPI_ISL_1705849

EPI_ISL_1705850

EPI_ISL_1705852

EPI_ISL_1705854

EPI_ISL_1705855

EPI_ISL_1705856

EPI_ISL_1705857

EPI_ISL_1705859

EPI_ISL_1705860

EPI_ISL_1705861

EPI_ISL_1705862

EPI_ISL_1705863

EPI_ISL_1705865

EPI_ISL_1705866

EPI_ISL_1705868

EPI_ISL_1705871

EPI_ISL_1705872

EPI_ISL_1705873

EPI_ISL_1705874

EPI_ISL_1705875

EPI_ISL_1705876

EPI_ISL_1705878

EPI_ISL_1705879

EPI_ISL_1705881

EPI_ISL_1705882

EPI_ISL_1705883

EPI_ISL_1705884

EPI_ISL_1705886

EPI_ISL_2149781

EPI_ISL_1705887

EPI_ISL_1705888

EPI_ISL_1705889

EPI_ISL_1705890

EPI_ISL_1705891

EPI_ISL_1705892

EPI_ISL_1705893

EPI_ISL_1705894

EPI_ISL_1705895

EPI_ISL_1705896

EPI_ISL_1705898

EPI_ISL_1705899

EPI_ISL_1705900

EPI_ISL_1705901

EPI_ISL_1705902

EPI_ISL_1705903

EPI_ISL_1705904

EPI_ISL_1705905

EPI_ISL_1705906

EPI_ISL_1705908

EPI_ISL_1705909

EPI_ISL_1705910

EPI_ISL_1705911

EPI_ISL_1705912

EPI_ISL_1705913

EPI_ISL_1705914

EPI_ISL_1705915

EPI_ISL_1705916

EPI_ISL_1705918

EPI_ISL_1705919

EPI_ISL_1705921

EPI_ISL_1705922

EPI_ISL_1705923

EPI_ISL_1705924

EPI_ISL_1705927

EPI_ISL_1705928

EPI_ISL_1705930

EPI_ISL_2330064

EPI_ISL_1705931

EPI_ISL_1705932

EPI_ISL_1705933

EPI_ISL_2149925

EPI_ISL_1705934

EPI_ISL_1705935

EPI_ISL_1705936

EPI_ISL_1705937

EPI_ISL_1705939

EPI_ISL_1705940

EPI_ISL_1705942

EPI_ISL_1705943

EPI_ISL_1705944

EPI_ISL_1705945

EPI_ISL_1705946

EPI_ISL_1705947

EPI_ISL_1705948

EPI_ISL_1705949

EPI_ISL_1705951

EPI_ISL_1705953

EPI_ISL_1705967

EPI_ISL_1705956

EPI_ISL_1705957

EPI_ISL_1572673

EPI_ISL_1705960

EPI_ISL_1705961

EPI_ISL_1705963

EPI_ISL_1705964

EPI_ISL_1705968

EPI_ISL_1705969

EPI_ISL_1705972

EPI_ISL_3480790

EPI_ISL_1705974

EPI_ISL_1705976

EPI_ISL_1705977

EPI_ISL_1705978

EPI_ISL_1705979

EPI_ISL_1705981

EPI_ISL_1705982

EPI_ISL_1705983

EPI_ISL_1705984

EPI_ISL_1705986

EPI_ISL_1705987

EPI_ISL_1705988

EPI_ISL_1705989

EPI_ISL_1705990

EPI_ISL_1705992

EPI_ISL_1705995

EPI_ISL_1705996

EPI_ISL_1705997

EPI_ISL_1705998

EPI_ISL_1705999

EPI_ISL_1706000

EPI_ISL_1706001

EPI_ISL_1706002

EPI_ISL_1706003

EPI_ISL_1706004

EPI_ISL_1706005

EPI_ISL_1706007

EPI_ISL_1706008

EPI_ISL_1706009

EPI_ISL_1706011

EPI_ISL_1706012

EPI_ISL_1706013

EPI_ISL_1706014

EPI_ISL_1706018

EPI_ISL_1706019

EPI_ISL_1706023

EPI_ISL_1706025

EPI_ISL_1706026

EPI_ISL_1706027

EPI_ISL_1706030

EPI_ISL_1706031

EPI_ISL_1706033

EPI_ISL_1706034

EPI_ISL_1706035

EPI_ISL_1706036

EPI_ISL_1706037

EPI_ISL_1706038

EPI_ISL_1706039

EPI_ISL_1706040

EPI_ISL_1706041

EPI_ISL_2712913

EPI_ISL_1706042

EPI_ISL_1706043

EPI_ISL_1706045

EPI_ISL_1706046

EPI_ISL_1706047

EPI_ISL_1706048

EPI_ISL_5323929

EPI_ISL_1706049

EPI_ISL_1706050

EPI_ISL_1706052

EPI_ISL_1706053

EPI_ISL_1706055

EPI_ISL_1706056

EPI_ISL_1706057

EPI_ISL_1706058

EPI_ISL_1706059

EPI_ISL_1706060

EPI_ISL_1706063

EPI_ISL_1706064

EPI_ISL_1706065

EPI_ISL_1706066

EPI_ISL_1706067

EPI_ISL_1706069

EPI_ISL_1706070

EPI_ISL_1706071

EPI_ISL_1706072

EPI_ISL_1706073

EPI_ISL_1706074

EPI_ISL_1706075

EPI_ISL_1706076

EPI_ISL_1706077

EPI_ISL_1706078

EPI_ISL_1706079

EPI_ISL_1706080

EPI_ISL_1706081

EPI_ISL_1706082

EPI_ISL_1706083

EPI_ISL_1706087

EPI_ISL_1706088

EPI_ISL_1706089

EPI_ISL_1706090

EPI_ISL_1706091

EPI_ISL_1706092

EPI_ISL_1706093

EPI_ISL_3480794

EPI_ISL_3480795

EPI_ISL_3478931

EPI_ISL_3478932

EPI_ISL_3478934

EPI_ISL_3478940

EPI_ISL_3478944

EPI_ISL_3478951

EPI_ISL_3478952

EPI_ISL_3478959

EPI_ISL_3478967

EPI_ISL_3478968

EPI_ISL_3478971

EPI_ISL_3478972

EPI_ISL_3478973

EPI_ISL_3480796

EPI_ISL_1706224

EPI_ISL_3478980

EPI_ISL_3478981

EPI_ISL_3478982

EPI_ISL_3478983

EPI_ISL_3478985

EPI_ISL_3478986

EPI_ISL_3478989

EPI_ISL_3478990

EPI_ISL_3478993

EPI_ISL_3478994

EPI_ISL_3478999

EPI_ISL_3479002

EPI_ISL_1706303

EPI_ISL_1706307

EPI_ISL_1706309

EPI_ISL_1706315

EPI_ISL_1706319

EPI_ISL_1706328

EPI_ISL_3479339

EPI_ISL_3479342

EPI_ISL_3479348

EPI_ISL_3479356

EPI_ISL_3479362

EPI_ISL_3479363

EPI_ISL_3479365

EPI_ISL_3479366

EPI_ISL_3479367

EPI_ISL_3479373

EPI_ISL_1706367

EPI_ISL_3479377

EPI_ISL_3479389

EPI_ISL_3479403

EPI_ISL_3479405

EPI_ISL_3479422

EPI_ISL_3479426

EPI_ISL_3479437

EPI_ISL_3479439

EPI_ISL_3479441

EPI_ISL_3479448

EPI_ISL_3479449

EPI_ISL_3479453

EPI_ISL_3479454

EPI_ISL_3479456

EPI_ISL_3479457

EPI_ISL_3479460

EPI_ISL_3479468

EPI_ISL_3479469

EPI_ISL_3479471

EPI_ISL_3479473

EPI_ISL_3479475

EPI_ISL_3479476

EPI_ISL_3479480

EPI_ISL_3479483

EPI_ISL_3479484

EPI_ISL_3479486

EPI_ISL_3479487

EPI_ISL_3479488

EPI_ISL_3479502

EPI_ISL_3479503

EPI_ISL_3479504

EPI_ISL_3479512

EPI_ISL_3479520

EPI_ISL_3479534

EPI_ISL_3479543

EPI_ISL_3479549

EPI_ISL_3479563

EPI_ISL_3479566

EPI_ISL_3479568

EPI_ISL_3479571

EPI_ISL_3479572

EPI_ISL_3479582

EPI_ISL_3479586

EPI_ISL_3479587

EPI_ISL_3479589

EPI_ISL_3479590

EPI_ISL_3479591

EPI_ISL_3479593

EPI_ISL_3479604

EPI_ISL_3479608

EPI_ISL_3479611

EPI_ISL_3479612

EPI_ISL_3479614

EPI_ISL_3479620

EPI_ISL_3479648

EPI_ISL_3479656

EPI_ISL_3479661

EPI_ISL_3479665

EPI_ISL_3479667

EPI_ISL_3479673

EPI_ISL_3479679

EPI_ISL_3479681

EPI_ISL_3479687

EPI_ISL_3479699

EPI_ISL_3479709

EPI_ISL_3479711

EPI_ISL_3479716

EPI_ISL_3479719

EPI_ISL_3479735

EPI_ISL_3479736

EPI_ISL_3479744

EPI_ISL_3479748

EPI_ISL_3479789

EPI_ISL_3479792

EPI_ISL_3479802

EPI_ISL_3479804

EPI_ISL_3479811

EPI_ISL_3479815

EPI_ISL_3479819

EPI_ISL_3479830

EPI_ISL_3479855

EPI_ISL_3479860

EPI_ISL_3479866

EPI_ISL_3479867

EPI_ISL_3480770

EPI_ISL_3480772

EPI_ISL_3479872

EPI_ISL_3479878

EPI_ISL_3480773

EPI_ISL_3479912

EPI_ISL_3479919

EPI_ISL_3479925

EPI_ISL_3479926

EPI_ISL_3479928

EPI_ISL_3479935

EPI_ISL_3479936

EPI_ISL_3479937

EPI_ISL_3479943

EPI_ISL_3479944

EPI_ISL_3479945

EPI_ISL_3479954

EPI_ISL_3479956

EPI_ISL_3479957

EPI_ISL_3479958

EPI_ISL_3479961

EPI_ISL_3479962

EPI_ISL_3479973

EPI_ISL_3479978

EPI_ISL_3479979

EPI_ISL_3480774

EPI_ISL_3479984

EPI_ISL_3479985

EPI_ISL_3479992

EPI_ISL_3480005

EPI_ISL_3480021

EPI_ISL_3480026

EPI_ISL_3480027

EPI_ISL_3480031

EPI_ISL_3480033

EPI_ISL_3480040

EPI_ISL_3480044

EPI_ISL_3480045

EPI_ISL_3480046

EPI_ISL_3480049

EPI_ISL_3480055

EPI_ISL_3480061

EPI_ISL_3480071

EPI_ISL_3480093

EPI_ISL_3480094

EPI_ISL_3480102

EPI_ISL_3480111

EPI_ISL_3480115

EPI_ISL_3480777

EPI_ISL_3480148

EPI_ISL_3480149

EPI_ISL_3480151

EPI_ISL_3480161

EPI_ISL_3480778

EPI_ISL_3480780

EPI_ISL_3480171

EPI_ISL_3480178

EPI_ISL_3480182

EPI_ISL_3480184

EPI_ISL_3480186

EPI_ISL_3480188

EPI_ISL_3480191

EPI_ISL_3480194

EPI_ISL_3480196

EPI_ISL_3480197

EPI_ISL_3480200

EPI_ISL_3480205

EPI_ISL_3480206

EPI_ISL_1921044

EPI_ISL_3480207

EPI_ISL_3480210

EPI_ISL_3480222

EPI_ISL_3480229

EPI_ISL_3480235

EPI_ISL_3480276

EPI_ISL_3480279

EPI_ISL_3480281

EPI_ISL_3480283

EPI_ISL_3480293

EPI_ISL_3480294

EPI_ISL_3480305

EPI_ISL_3480313

EPI_ISL_3480314

EPI_ISL_3480315

EPI_ISL_3480317

EPI_ISL_3480318

EPI_ISL_3480319

EPI_ISL_3480320

EPI_ISL_3480321

EPI_ISL_3480322

EPI_ISL_3480324

EPI_ISL_3480325

EPI_ISL_3480326

EPI_ISL_3480327

EPI_ISL_3480328

EPI_ISL_3480329

EPI_ISL_3480340

EPI_ISL_3480341

EPI_ISL_3480343

EPI_ISL_3480344

EPI_ISL_3480368

EPI_ISL_3480374

EPI_ISL_3480375

EPI_ISL_1706483

EPI_ISL_3480382

EPI_ISL_1706484

EPI_ISL_3480384

EPI_ISL_3480390

EPI_ISL_3480397

EPI_ISL_3480399

EPI_ISL_3480401

EPI_ISL_1706485

EPI_ISL_3480402

EPI_ISL_3480404

EPI_ISL_3480405

EPI_ISL_1706491

EPI_ISL_3480406

EPI_ISL_3480412

EPI_ISL_3480415

EPI_ISL_3480416

EPI_ISL_3480418

EPI_ISL_3480419

EPI_ISL_3480421

EPI_ISL_3480425

EPI_ISL_3480432

EPI_ISL_3480440

EPI_ISL_3480441

EPI_ISL_3480442

EPI_ISL_3480443

EPI_ISL_3480444

EPI_ISL_3480445

EPI_ISL_3480451

EPI_ISL_3480455

EPI_ISL_3480458

EPI_ISL_3480461

EPI_ISL_3480465

EPI_ISL_3480470

EPI_ISL_3480471

EPI_ISL_3480475

EPI_ISL_3480476

EPI_ISL_3480479

EPI_ISL_3480486

EPI_ISL_3480491

EPI_ISL_3480497

EPI_ISL_3480498

EPI_ISL_3480500

EPI_ISL_3480504

EPI_ISL_3480507

EPI_ISL_3480508

EPI_ISL_3480513

EPI_ISL_3480525

EPI_ISL_3480527

EPI_ISL_3480529

EPI_ISL_3480530

EPI_ISL_3480531

EPI_ISL_3480532

EPI_ISL_3480533

EPI_ISL_3480537

EPI_ISL_3480542

EPI_ISL_3480548

EPI_ISL_3480549

EPI_ISL_3480550

EPI_ISL_3480553

EPI_ISL_3480555

EPI_ISL_3480784

EPI_ISL_3480559

EPI_ISL_3480562

EPI_ISL_3480563

EPI_ISL_3480566

EPI_ISL_3480567

EPI_ISL_3480568

EPI_ISL_3480569

EPI_ISL_3480570

EPI_ISL_3480573

EPI_ISL_3480574

EPI_ISL_3480575

EPI_ISL_3480581

EPI_ISL_3480585

EPI_ISL_3480589

EPI_ISL_3480598

EPI_ISL_3480607

EPI_ISL_3480610

EPI_ISL_3480615

EPI_ISL_3480625

EPI_ISL_3480628

EPI_ISL_3480629

EPI_ISL_3480630

EPI_ISL_3480632

EPI_ISL_3480637

EPI_ISL_3480640

EPI_ISL_3480643

EPI_ISL_3480644

EPI_ISL_3480645

EPI_ISL_3480646

EPI_ISL_3480647

EPI_ISL_3480648

EPI_ISL_3480651

EPI_ISL_3480653

EPI_ISL_3480658

EPI_ISL_3480662

EPI_ISL_3480663

EPI_ISL_3480664

EPI_ISL_3480666

EPI_ISL_3480667

EPI_ISL_3480669

EPI_ISL_3480670

EPI_ISL_3480674

EPI_ISL_3480679

EPI_ISL_3480680

EPI_ISL_3480682

EPI_ISL_3480686

EPI_ISL_3480687

EPI_ISL_3480688

EPI_ISL_3480690

EPI_ISL_3480692

EPI_ISL_3480695

EPI_ISL_3480697

EPI_ISL_3480699

EPI_ISL_3480705

EPI_ISL_3480707

EPI_ISL_3480709

EPI_ISL_3480710

EPI_ISL_3480711

EPI_ISL_3480712

EPI_ISL_3480714

EPI_ISL_3480715

EPI_ISL_3480716

EPI_ISL_3480717

EPI_ISL_3480718

EPI_ISL_3480719

EPI_ISL_3480721

EPI_ISL_3480723

EPI_ISL_3480725

EPI_ISL_3480729

EPI_ISL_3480730

EPI_ISL_3480731

EPI_ISL_3480732

EPI_ISL_3480742

EPI_ISL_3480743

EPI_ISL_3480744

EPI_ISL_3480746

EPI_ISL_3480749

EPI_ISL_3480753

EPI_ISL_3480756

EPI_ISL_3480759

EPI_ISL_3480762

EPI_ISL_3480764

EPI_ISL_3480765

EPI_ISL_3480766

EPI_ISL_3480798

EPI_ISL_3480799

EPI_ISL_3480800

EPI_ISL_3480801

EPI_ISL_3480804

EPI_ISL_3480814

EPI_ISL_3480817

EPI_ISL_3480818

EPI_ISL_3480821

EPI_ISL_3480822

EPI_ISL_3480824

EPI_ISL_3480828

EPI_ISL_3480830

EPI_ISL_3480831

EPI_ISL_3480833

EPI_ISL_3480834

EPI_ISL_3480837

EPI_ISL_3480839

EPI_ISL_3480840

EPI_ISL_3480844

EPI_ISL_3480847

EPI_ISL_3480849

EPI_ISL_3480850

EPI_ISL_3480857

EPI_ISL_3480864

EPI_ISL_3480867

EPI_ISL_3480869

EPI_ISL_3480870

EPI_ISL_3480874

EPI_ISL_2673068

EPI_ISL_3480885

EPI_ISL_3480889

EPI_ISL_3480891

EPI_ISL_3480894

EPI_ISL_3480897

EPI_ISL_3480898

EPI_ISL_3480899

EPI_ISL_3480903

EPI_ISL_3480911

EPI_ISL_3480912

EPI_ISL_3480916

EPI_ISL_3480918

EPI_ISL_3480921

EPI_ISL_3480931

EPI_ISL_2673069

EPI_ISL_3480938

EPI_ISL_2673071

EPI_ISL_1571570

EPI_ISL_3480940

EPI_ISL_3480941

EPI_ISL_3480946

EPI_ISL_3480952

EPI_ISL_3480954

EPI_ISL_3480959

EPI_ISL_3480960

EPI_ISL_3480961

EPI_ISL_3480963

EPI_ISL_3480969

EPI_ISL_3480972

EPI_ISL_3480976

EPI_ISL_3480982

EPI_ISL_3480983

EPI_ISL_3480989

EPI_ISL_3480997

EPI_ISL_2719308

EPI_ISL_3481001

EPI_ISL_3481003

EPI_ISL_3481007

EPI_ISL_3481008

EPI_ISL_3481010

EPI_ISL_3481015

EPI_ISL_3481017

EPI_ISL_3481019

EPI_ISL_3481022

EPI_ISL_3481023

EPI_ISL_3481035

EPI_ISL_3481042

EPI_ISL_3481044

EPI_ISL_3481051

EPI_ISL_3481052

EPI_ISL_3481054

EPI_ISL_3481055

EPI_ISL_3481060

EPI_ISL_3481069

EPI_ISL_3481073

EPI_ISL_3481074

EPI_ISL_3481075

EPI_ISL_3481082

EPI_ISL_3481083

EPI_ISL_3481084

EPI_ISL_3481085

EPI_ISL_2673077

EPI_ISL_3481091

EPI_ISL_3481092

EPI_ISL_3481097

EPI_ISL_3481098

EPI_ISL_3481108

EPI_ISL_2673078

EPI_ISL_3481111

EPI_ISL_3481112

EPI_ISL_3481113

EPI_ISL_3481114

EPI_ISL_3481115

EPI_ISL_3481117

EPI_ISL_3481118

EPI_ISL_3481120

EPI_ISL_3481121

EPI_ISL_3481122

EPI_ISL_3481123

EPI_ISL_3481129

EPI_ISL_3481131

EPI_ISL_3481132

EPI_ISL_3481133

EPI_ISL_3481134

EPI_ISL_3481135

EPI_ISL_3481142

EPI_ISL_3481145

EPI_ISL_3481148

EPI_ISL_2719289

EPI_ISL_3481153

EPI_ISL_3481157

EPI_ISL_3481158

EPI_ISL_3481170

EPI_ISL_3481171

EPI_ISL_2673079

EPI_ISL_3481181

EPI_ISL_3481182

EPI_ISL_3481183

EPI_ISL_3481184

EPI_ISL_3481185

EPI_ISL_3481190

EPI_ISL_3481191

EPI_ISL_3481193

EPI_ISL_3481197

EPI_ISL_3481198

EPI_ISL_3481199

EPI_ISL_3481200

EPI_ISL_3481201

EPI_ISL_3481204

EPI_ISL_3481205

EPI_ISL_3481207

EPI_ISL_3481208

EPI_ISL_3481209

EPI_ISL_3481210

EPI_ISL_3481211

EPI_ISL_3481215

EPI_ISL_3481217

EPI_ISL_3481219

EPI_ISL_3481222

EPI_ISL_3481223

EPI_ISL_3481224

EPI_ISL_3481227

EPI_ISL_3481229

EPI_ISL_3481234

EPI_ISL_3481235

EPI_ISL_3481238

EPI_ISL_3481239

EPI_ISL_3481240

EPI_ISL_3481244

EPI_ISL_3481245

EPI_ISL_3481246

EPI_ISL_3481252

EPI_ISL_3481253

EPI_ISL_3481254

EPI_ISL_3481256

EPI_ISL_3481259

EPI_ISL_2719294

EPI_ISL_3481262

EPI_ISL_3481265

EPI_ISL_3481266

EPI_ISL_3481269

EPI_ISL_3481270

EPI_ISL_3481271

EPI_ISL_3481272

EPI_ISL_3481278

EPI_ISL_3481281

EPI_ISL_3481282

EPI_ISL_3481289

EPI_ISL_3481293

EPI_ISL_3481297

EPI_ISL_3481298

EPI_ISL_3481300

EPI_ISL_3481301

EPI_ISL_3481302

EPI_ISL_3481305

EPI_ISL_3481306

EPI_ISL_3481308

EPI_ISL_3481310

EPI_ISL_3481322

EPI_ISL_3481323

EPI_ISL_3481326

EPI_ISL_3481330

EPI_ISL_3481336

EPI_ISL_3481340

EPI_ISL_3481341

EPI_ISL_3481347

EPI_ISL_2673080

EPI_ISL_3481348

EPI_ISL_1706577

EPI_ISL_3481351

EPI_ISL_3481356

EPI_ISL_1706578

EPI_ISL_3481367

EPI_ISL_3481371

EPI_ISL_3481373

EPI_ISL_3481374

EPI_ISL_3481382

EPI_ISL_3481388

EPI_ISL_3481392

EPI_ISL_3481394

EPI_ISL_3481396

EPI_ISL_1706582

EPI_ISL_3481400

EPI_ISL_3481402

EPI_ISL_1706583

EPI_ISL_3481409

EPI_ISL_3481420

EPI_ISL_3481427

EPI_ISL_1706584

EPI_ISL_3481439

EPI_ISL_3481445

EPI_ISL_3481452

EPI_ISL_3481454

EPI_ISL_3481455

EPI_ISL_3481457

EPI_ISL_3481462

EPI_ISL_3481466

EPI_ISL_3481467

EPI_ISL_3481479

EPI_ISL_3481486

EPI_ISL_3481489

EPI_ISL_3481502

EPI_ISL_3481507

EPI_ISL_3481513

EPI_ISL_3481515

EPI_ISL_3481525

EPI_ISL_3481531

EPI_ISL_3481533

EPI_ISL_2673087

EPI_ISL_3481550

EPI_ISL_3481554

EPI_ISL_2673088

EPI_ISL_3481558

EPI_ISL_3481563

EPI_ISL_3481567

EPI_ISL_3481571

EPI_ISL_3481576

EPI_ISL_3481577

EPI_ISL_3481581

EPI_ISL_3481588

EPI_ISL_3481591

EPI_ISL_3481602

EPI_ISL_3481606

EPI_ISL_3481610

EPI_ISL_3481611

EPI_ISL_3481613

EPI_ISL_3481615

EPI_ISL_3481636

EPI_ISL_3481638

EPI_ISL_3481640

EPI_ISL_3481645

EPI_ISL_3481647

EPI_ISL_3481650

EPI_ISL_3481655

EPI_ISL_3481657

EPI_ISL_3481659

EPI_ISL_3481681

EPI_ISL_3481694

EPI_ISL_3481696

EPI_ISL_3481717

EPI_ISL_3481719

EPI_ISL_2673089

EPI_ISL_3481733

EPI_ISL_3481735

EPI_ISL_3481738

EPI_ISL_3481746

EPI_ISL_3481751

EPI_ISL_3481754

EPI_ISL_2673090

EPI_ISL_3481759

EPI_ISL_3481766

EPI_ISL_3481769

EPI_ISL_3481783

EPI_ISL_3481793

EPI_ISL_3481794

EPI_ISL_3481802

EPI_ISL_3481805

EPI_ISL_3481808

EPI_ISL_3481810

EPI_ISL_3481816

EPI_ISL_3481819

EPI_ISL_3481823

EPI_ISL_3481824

EPI_ISL_3481826

EPI_ISL_3481827

EPI_ISL_3481828

EPI_ISL_3481830

EPI_ISL_3481838

EPI_ISL_3481842

EPI_ISL_3481846

EPI_ISL_3481849

EPI_ISL_3481850

EPI_ISL_3481851

EPI_ISL_3481852

EPI_ISL_3481853

EPI_ISL_3481855

EPI_ISL_3481856

EPI_ISL_3481857

EPI_ISL_3481858

EPI_ISL_3481859

EPI_ISL_3481860

EPI_ISL_3481861

EPI_ISL_3481864

EPI_ISL_3481868

EPI_ISL_3481871

EPI_ISL_3481872

EPI_ISL_3481873

EPI_ISL_3481874

EPI_ISL_3481875

EPI_ISL_3481877

EPI_ISL_3481882

EPI_ISL_3481890

EPI_ISL_3481896

EPI_ISL_3481897

EPI_ISL_3481908

EPI_ISL_3481913

EPI_ISL_3481914

EPI_ISL_3481915

EPI_ISL_3481916

EPI_ISL_3481919

EPI_ISL_3481920

EPI_ISL_3481927

EPI_ISL_3481933

EPI_ISL_3481938

EPI_ISL_3481945

EPI_ISL_3481946

EPI_ISL_3481949

EPI_ISL_3481950

EPI_ISL_3481951

EPI_ISL_3481953

EPI_ISL_3481956

EPI_ISL_3481958

EPI_ISL_3481961

EPI_ISL_3481963

EPI_ISL_3481965

EPI_ISL_3481967

EPI_ISL_3481972

EPI_ISL_3481975

EPI_ISL_3481977

EPI_ISL_3481978

EPI_ISL_3481986

EPI_ISL_3481996

EPI_ISL_3482008

EPI_ISL_3482013

EPI_ISL_3482028

EPI_ISL_3482039

EPI_ISL_3482043

EPI_ISL_3482050

EPI_ISL_3482052

EPI_ISL_3482070

EPI_ISL_3482077

EPI_ISL_2508116

EPI_ISL_3482086

EPI_ISL_3482096

EPI_ISL_3482103

EPI_ISL_3482104

EPI_ISL_3482110

EPI_ISL_3482114

EPI_ISL_3482115

EPI_ISL_3482120

EPI_ISL_3482121

EPI_ISL_3482123

EPI_ISL_3482127

EPI_ISL_3482128

EPI_ISL_3482130

EPI_ISL_3482138

EPI_ISL_3482139

EPI_ISL_3482140

EPI_ISL_3482141

EPI_ISL_3482143

EPI_ISL_3482144

EPI_ISL_3482145

EPI_ISL_3482148

EPI_ISL_3482153

EPI_ISL_3482154

EPI_ISL_3482155

EPI_ISL_3482163

EPI_ISL_3482170

EPI_ISL_3482172

EPI_ISL_3482174

EPI_ISL_3482175

EPI_ISL_3482182

EPI_ISL_3482188

EPI_ISL_3482191

EPI_ISL_3482199

EPI_ISL_3482205

EPI_ISL_3482210

EPI_ISL_3482213

EPI_ISL_3482214

EPI_ISL_3482215

EPI_ISL_3482219

EPI_ISL_3482220

EPI_ISL_3482223

EPI_ISL_3482225

EPI_ISL_3482226

EPI_ISL_3482228

EPI_ISL_3482237

EPI_ISL_3482244

EPI_ISL_3482245

EPI_ISL_3482246

EPI_ISL_3482253

EPI_ISL_2673096

EPI_ISL_2673097

EPI_ISL_3482267

EPI_ISL_3482273

EPI_ISL_3482274

EPI_ISL_3482277

EPI_ISL_3482283

EPI_ISL_3482284

EPI_ISL_3482286

EPI_ISL_3482288

EPI_ISL_2673098

EPI_ISL_3482292

EPI_ISL_3482295

EPI_ISL_3482304

EPI_ISL_3482305

EPI_ISL_3482316

EPI_ISL_3482325

EPI_ISL_3482326

EPI_ISL_3482337

EPI_ISL_3482346

EPI_ISL_3482347

EPI_ISL_3482348

EPI_ISL_3482349

EPI_ISL_3482350

EPI_ISL_3482354

EPI_ISL_3482356

EPI_ISL_3482358

EPI_ISL_3482359

EPI_ISL_3482362

EPI_ISL_3482367

EPI_ISL_3482370

EPI_ISL_3482381

EPI_ISL_3482384

EPI_ISL_3482386

EPI_ISL_3482388

EPI_ISL_3482389

EPI_ISL_3482393

EPI_ISL_3482394

EPI_ISL_3482397

EPI_ISL_3482399

EPI_ISL_3482405

EPI_ISL_2673102

EPI_ISL_3482410

EPI_ISL_3482415

EPI_ISL_3482418

EPI_ISL_2673103

EPI_ISL_3482422

EPI_ISL_3482425

EPI_ISL_2673104

EPI_ISL_3482427

EPI_ISL_3482435

EPI_ISL_3482437

EPI_ISL_3482438

EPI_ISL_3482440

EPI_ISL_3482441

EPI_ISL_3482450

EPI_ISL_3482453

EPI_ISL_3482507

EPI_ISL_3482511

EPI_ISL_3482513

EPI_ISL_3482543

EPI_ISL_2673106

EPI_ISL_5329846

EPI_ISL_3482768

EPI_ISL_3482773

EPI_ISL_3482778

EPI_ISL_3482791

EPI_ISL_3482797

EPI_ISL_2508247

EPI_ISL_3482954

EPI_ISL_3482963

EPI_ISL_2673113

EPI_ISL_2673114

EPI_ISL_1571584

EPI_ISL_3482965

EPI_ISL_3482980

EPI_ISL_1922168

EPI_ISL_2673121

EPI_ISL_2508283

EPI_ISL_2673122

EPI_ISL_2673123

EPI_ISL_1922182

EPI_ISL_2718874

EPI_ISL_2718928

EPI_ISL_2718938

EPI_ISL_2673129

EPI_ISL_2673130

EPI_ISL_2673131

EPI_ISL_2508342

EPI_ISL_2719353

EPI_ISL_2719379

EPI_ISL_2719622

EPI_ISL_2153369

EPI_ISL_2530152

EPI_ISL_2153574

EPI_ISL_7796064

EPI_ISL_7796055

EPI_ISL_2724586

EPI_ISL_2724622

EPI_ISL_1707045

EPI_ISL_1707046

EPI_ISL_1707048

EPI_ISL_1707057

EPI_ISL_1707059

EPI_ISL_1707060

EPI_ISL_1707061

EPI_ISL_1707089

EPI_ISL_1707098

EPI_ISL_1749484

EPI_ISL_1749564

EPI_ISL_1749573

EPI_ISL_2888931

EPI_ISL_2727412

EPI_ISL_2727554

EPI_ISL_1707663

EPI_ISL_1707664

EPI_ISL_1707665

EPI_ISL_3494220

EPI_ISL_3494221

EPI_ISL_3494229

EPI_ISL_3494232

EPI_ISL_3494235

EPI_ISL_3494237

EPI_ISL_3494242

EPI_ISL_3494247

EPI_ISL_3494313

EPI_ISL_3494324

EPI_ISL_3494327

EPI_ISL_3494332

EPI_ISL_3494370

EPI_ISL_3494371

EPI_ISL_3494375

EPI_ISL_3494376

EPI_ISL_3494380

EPI_ISL_3494381

EPI_ISL_3494384

EPI_ISL_3494385

EPI_ISL_3494386

EPI_ISL_3494388

EPI_ISL_3494389

EPI_ISL_3494390

EPI_ISL_3494391

EPI_ISL_3494393

EPI_ISL_3494395

EPI_ISL_3494397

EPI_ISL_3494400

EPI_ISL_3494402

EPI_ISL_3494404

EPI_ISL_3494406

EPI_ISL_3494407

EPI_ISL_3494408

EPI_ISL_3494409

EPI_ISL_3494410

EPI_ISL_3494412

EPI_ISL_3494415

EPI_ISL_3494416

EPI_ISL_3494419

EPI_ISL_3494420

EPI_ISL_3494421

EPI_ISL_3494422

EPI_ISL_3494427

EPI_ISL_3494428

EPI_ISL_3494431

EPI_ISL_3494435

EPI_ISL_3494439

EPI_ISL_3494440

EPI_ISL_3494441

EPI_ISL_3494442

EPI_ISL_3494446

EPI_ISL_3494448

EPI_ISL_3494456

EPI_ISL_3494459

EPI_ISL_2673452

EPI_ISL_1707529

EPI_ISL_1707531

EPI_ISL_1707539

EPI_ISL_1707541

EPI_ISL_1707542

EPI_ISL_1707543

EPI_ISL_1707544

EPI_ISL_1707545

EPI_ISL_1707546

EPI_ISL_1707547

EPI_ISL_2673453

EPI_ISL_1707548

EPI_ISL_1707549

EPI_ISL_1707550

EPI_ISL_1707552

EPI_ISL_1707553

EPI_ISL_1707554

EPI_ISL_1707555

EPI_ISL_1707556

EPI_ISL_1707557

EPI_ISL_1707559

EPI_ISL_1707560

EPI_ISL_1707561

EPI_ISL_1707562

EPI_ISL_1707564

EPI_ISL_1707565

EPI_ISL_1707566

EPI_ISL_1707567

EPI_ISL_1707568

EPI_ISL_1707569

EPI_ISL_1707570

EPI_ISL_1707571

EPI_ISL_1707572

EPI_ISL_1707573

EPI_ISL_1707575

EPI_ISL_1707576

EPI_ISL_1707577

EPI_ISL_1707578

EPI_ISL_1707579

EPI_ISL_1707582

EPI_ISL_1707583

EPI_ISL_1707584

EPI_ISL_1707585

EPI_ISL_1707586

EPI_ISL_1707587

EPI_ISL_1707589

EPI_ISL_2673423

EPI_ISL_2531091

EPI_ISL_2673424

EPI_ISL_2673425

EPI_ISL_1707590

EPI_ISL_2531093

EPI_ISL_2673426

EPI_ISL_2673427

EPI_ISL_2673428

EPI_ISL_2673429

EPI_ISL_1707591

EPI_ISL_1707592

EPI_ISL_1707593

EPI_ISL_1707594

EPI_ISL_1707596

EPI_ISL_1707597

EPI_ISL_1707598

EPI_ISL_1707599

EPI_ISL_2673431

EPI_ISL_1707600

EPI_ISL_1707602

EPI_ISL_1707603

EPI_ISL_1707604

EPI_ISL_1707606

EPI_ISL_2673432

EPI_ISL_1707608

EPI_ISL_2673433

EPI_ISL_2673434

EPI_ISL_1707609

EPI_ISL_1707610

EPI_ISL_1707611

EPI_ISL_1707612

EPI_ISL_1707613

EPI_ISL_1707614

EPI_ISL_2673435

EPI_ISL_1707615

EPI_ISL_1707616

EPI_ISL_2673436

EPI_ISL_1707617

EPI_ISL_1707618

EPI_ISL_1707619

EPI_ISL_1707620

EPI_ISL_2673437

EPI_ISL_2673438

EPI_ISL_2673439

EPI_ISL_2673440

EPI_ISL_2673441

EPI_ISL_2673442

EPI_ISL_1707621

EPI_ISL_1707622

EPI_ISL_1707623

EPI_ISL_2673443

EPI_ISL_1707624

EPI_ISL_1707625

EPI_ISL_1707626

EPI_ISL_1707627

EPI_ISL_1707628

EPI_ISL_1707629

EPI_ISL_1707630

EPI_ISL_1707631

EPI_ISL_2673444

EPI_ISL_1707632

EPI_ISL_1707633

EPI_ISL_1707634

EPI_ISL_1707635

EPI_ISL_1707636

EPI_ISL_1707637

EPI_ISL_1707638

EPI_ISL_1707639

EPI_ISL_1707640

EPI_ISL_1707641

EPI_ISL_1707642

EPI_ISL_1707643

EPI_ISL_2673446

EPI_ISL_1707644

EPI_ISL_2673447

EPI_ISL_1707646

EPI_ISL_1707647

EPI_ISL_1707648

EPI_ISL_1707649

EPI_ISL_1707651

EPI_ISL_1707652

EPI_ISL_1707653

EPI_ISL_1707654

EPI_ISL_2673448

EPI_ISL_2673449

EPI_ISL_2673450

EPI_ISL_2673451

EPI_ISL_1707655

EPI_ISL_1707657

EPI_ISL_1707658

EPI_ISL_1707659

EPI_ISL_1707661

EPI_ISL_1707662

EPI_ISL_2673454

EPI_ISL_2673554

EPI_ISL_1707667

EPI_ISL_1707668

EPI_ISL_1707669

EPI_ISL_1707670

EPI_ISL_1707671

EPI_ISL_2673455

EPI_ISL_2673456

EPI_ISL_2673457

EPI_ISL_1707672

EPI_ISL_1707674

EPI_ISL_2673458

EPI_ISL_2673459

EPI_ISL_2673460

EPI_ISL_2673462

EPI_ISL_1707680

EPI_ISL_1707682

EPI_ISL_1707683

EPI_ISL_1707684

EPI_ISL_1707685

EPI_ISL_2673463

EPI_ISL_2673464

EPI_ISL_2673465

EPI_ISL_2673466

EPI_ISL_2673467

EPI_ISL_2673468

EPI_ISL_2673469

EPI_ISL_2673470

EPI_ISL_1707689

EPI_ISL_2673472

EPI_ISL_1707690

EPI_ISL_2673474

EPI_ISL_2975713

EPI_ISL_2673476

EPI_ISL_2673477

EPI_ISL_1707691

EPI_ISL_2975714

EPI_ISL_2975712

EPI_ISL_1707692

EPI_ISL_2673479

EPI_ISL_2673480

EPI_ISL_2673482

EPI_ISL_2673483

EPI_ISL_2673484

EPI_ISL_2673486

EPI_ISL_2333372

EPI_ISL_2673487

EPI_ISL_2673488

EPI_ISL_2673489

EPI_ISL_2673490

EPI_ISL_2673491

EPI_ISL_2673492

EPI_ISL_2673493

EPI_ISL_2673494

EPI_ISL_2673495

EPI_ISL_2673496

EPI_ISL_2673497

EPI_ISL_2673498

EPI_ISL_2673499

EPI_ISL_2673500

EPI_ISL_2673502

EPI_ISL_3796984

EPI_ISL_2673522

EPI_ISL_2673523

EPI_ISL_2673524

EPI_ISL_2673525

EPI_ISL_2673526

EPI_ISL_2673527

EPI_ISL_2673528

EPI_ISL_2673529

EPI_ISL_2673530

EPI_ISL_2673531

EPI_ISL_2673532

EPI_ISL_2673533

EPI_ISL_2673534

EPI_ISL_2673535

EPI_ISL_2673536

EPI_ISL_2673537

EPI_ISL_2673538

EPI_ISL_2673539

EPI_ISL_2673541

EPI_ISL_2673542

EPI_ISL_2673544

EPI_ISL_2673545

EPI_ISL_2673546

EPI_ISL_2673547

EPI_ISL_2673548

EPI_ISL_2673549

EPI_ISL_2673550

EPI_ISL_2673551

EPI_ISL_2673552

EPI_ISL_2673553

EPI_ISL_2673555

EPI_ISL_2673556

EPI_ISL_2673557

EPI_ISL_2673558

EPI_ISL_2673559

EPI_ISL_2673560

EPI_ISL_2673561

EPI_ISL_2673562

EPI_ISL_2673563

EPI_ISL_2673564

EPI_ISL_2673565

EPI_ISL_2673566

EPI_ISL_2673567

EPI_ISL_2673568

EPI_ISL_2673569

EPI_ISL_2673570

EPI_ISL_2673571

EPI_ISL_2673572

EPI_ISL_2673573

EPI_ISL_1707990

EPI_ISL_1707991

EPI_ISL_1707992

EPI_ISL_2673591

EPI_ISL_2673592

EPI_ISL_2673593

EPI_ISL_2673594

EPI_ISL_2673595

EPI_ISL_2673596

EPI_ISL_2673597

EPI_ISL_2673598

EPI_ISL_2673599

EPI_ISL_2673600

EPI_ISL_2673602

EPI_ISL_2673603

EPI_ISL_2673604

EPI_ISL_1707996

EPI_ISL_2673606

EPI_ISL_2673607

EPI_ISL_2673608

EPI_ISL_1707997

EPI_ISL_2673609

EPI_ISL_2673610

EPI_ISL_2673611

EPI_ISL_1707955

EPI_ISL_1707956

EPI_ISL_1707957

EPI_ISL_1707958

EPI_ISL_1707959

EPI_ISL_1707960

EPI_ISL_1707961

EPI_ISL_1707964

EPI_ISL_1707965

EPI_ISL_1707966

EPI_ISL_1707967

EPI_ISL_1707968

EPI_ISL_1707969

EPI_ISL_1707970

EPI_ISL_1707971

EPI_ISL_1707972

EPI_ISL_1707973

EPI_ISL_1707974

EPI_ISL_3796802

EPI_ISL_1707975

EPI_ISL_1707976

EPI_ISL_1707977

EPI_ISL_1707978

EPI_ISL_1707979

EPI_ISL_2334152

EPI_ISL_2334160

EPI_ISL_1707980

EPI_ISL_1707982

EPI_ISL_1707983

EPI_ISL_3501887

EPI_ISL_3501889

EPI_ISL_3501890

EPI_ISL_1707984

EPI_ISL_1707985

EPI_ISL_1707986

EPI_ISL_2334177

EPI_ISL_2334178

EPI_ISL_1707988

EPI_ISL_1707989

EPI_ISL_1707998

EPI_ISL_1707999

EPI_ISL_2334208

EPI_ISL_1708000

EPI_ISL_2334225

EPI_ISL_2334239

EPI_ISL_1708001

EPI_ISL_1708004

EPI_ISL_2334260

EPI_ISL_1708005

EPI_ISL_1708007

EPI_ISL_1708008

EPI_ISL_1708009

EPI_ISL_1708010

EPI_ISL_1708094

EPI_ISL_2334334

EPI_ISL_2334342

EPI_ISL_1708011

EPI_ISL_1708012

EPI_ISL_1708013

EPI_ISL_2334384

EPI_ISL_2334405

EPI_ISL_1708014

EPI_ISL_1708015

EPI_ISL_1708016

EPI_ISL_3502490

EPI_ISL_2334412

EPI_ISL_3502497

EPI_ISL_3502498

EPI_ISL_3502502

EPI_ISL_3502504

EPI_ISL_1708017

EPI_ISL_3502519

EPI_ISL_1708018

EPI_ISL_1708019

EPI_ISL_1708020

EPI_ISL_3502529

EPI_ISL_3502530

EPI_ISL_3502532

EPI_ISL_1708021

EPI_ISL_3502534

EPI_ISL_3502535

EPI_ISL_3502536

EPI_ISL_3502537

EPI_ISL_3502538

EPI_ISL_3502539

EPI_ISL_3502540

EPI_ISL_3502541

EPI_ISL_3502542

EPI_ISL_3502544

EPI_ISL_3502545

EPI_ISL_3502546

EPI_ISL_3502548

EPI_ISL_3502549

EPI_ISL_3502550

EPI_ISL_3502551

EPI_ISL_3502552

EPI_ISL_3502553

EPI_ISL_3502554

EPI_ISL_3502555

EPI_ISL_3502556

EPI_ISL_3502557

EPI_ISL_3502558

EPI_ISL_1708022

EPI_ISL_1708023

EPI_ISL_1708024

EPI_ISL_1708025

EPI_ISL_1708026

EPI_ISL_1708027

EPI_ISL_1708028

EPI_ISL_1708029

EPI_ISL_1708030

EPI_ISL_1708031

EPI_ISL_1708032

EPI_ISL_1708033

EPI_ISL_1708034

EPI_ISL_1708035

EPI_ISL_1708036

EPI_ISL_1708038

EPI_ISL_1708040

EPI_ISL_1708041

EPI_ISL_1708042

EPI_ISL_1708043

EPI_ISL_2334493

EPI_ISL_2334490

EPI_ISL_1708044

EPI_ISL_1708046

EPI_ISL_1708047

EPI_ISL_1708048

EPI_ISL_1708049

EPI_ISL_1708050

EPI_ISL_1708051

EPI_ISL_1708052

EPI_ISL_1708054

EPI_ISL_1708055

EPI_ISL_1708056

EPI_ISL_1708057

EPI_ISL_1708058

EPI_ISL_1708060

EPI_ISL_1708061

EPI_ISL_1708062

EPI_ISL_1708064

EPI_ISL_1708065

EPI_ISL_1708066

EPI_ISL_2334515

EPI_ISL_1708067

EPI_ISL_1708068

EPI_ISL_1708069

EPI_ISL_1708070

EPI_ISL_1708071

EPI_ISL_1708072

EPI_ISL_1708073

EPI_ISL_3502894

EPI_ISL_1708074

EPI_ISL_1708075

EPI_ISL_1708076

EPI_ISL_1708077

EPI_ISL_1708078

EPI_ISL_1708079

EPI_ISL_1708080

EPI_ISL_1708081

EPI_ISL_1708082

EPI_ISL_1708097

EPI_ISL_1708083

EPI_ISL_1708084

EPI_ISL_1708085

EPI_ISL_1708086

EPI_ISL_1708087

EPI_ISL_1708088

EPI_ISL_1708089

EPI_ISL_1708091

EPI_ISL_1708098

EPI_ISL_1708099

EPI_ISL_1708093

EPI_ISL_1708100

EPI_ISL_1708101

EPI_ISL_1708102

EPI_ISL_1708103

EPI_ISL_1708104

EPI_ISL_1708105

EPI_ISL_1708108

EPI_ISL_1708109

EPI_ISL_1708110

EPI_ISL_1708111

EPI_ISL_1708112

EPI_ISL_1708114

EPI_ISL_1708116

EPI_ISL_1708118

EPI_ISL_1708119

EPI_ISL_1708120

EPI_ISL_1708121

EPI_ISL_1708123

EPI_ISL_1708125

EPI_ISL_1708126

EPI_ISL_1708127

EPI_ISL_1708128

EPI_ISL_1708129

EPI_ISL_1708130

EPI_ISL_1708133

EPI_ISL_1708134

EPI_ISL_1708136

EPI_ISL_1708137

EPI_ISL_1708138

EPI_ISL_1708140

EPI_ISL_1708141

EPI_ISL_1708143

EPI_ISL_1708144

EPI_ISL_1708145

EPI_ISL_1708146

EPI_ISL_1708147

EPI_ISL_1708148

EPI_ISL_1708149

EPI_ISL_1708150

EPI_ISL_1708151

EPI_ISL_1708152

EPI_ISL_1708153

EPI_ISL_1708154

EPI_ISL_1708155

EPI_ISL_1708156

EPI_ISL_1708157

EPI_ISL_1708158

EPI_ISL_1708159

EPI_ISL_1708160

EPI_ISL_1708162

EPI_ISL_1708163

EPI_ISL_1708165

EPI_ISL_1708166

EPI_ISL_1708167

EPI_ISL_1708168

EPI_ISL_1708170

EPI_ISL_1708171

EPI_ISL_1708172

EPI_ISL_1708173

EPI_ISL_1708174

EPI_ISL_1708175

EPI_ISL_1708176

EPI_ISL_1708177

EPI_ISL_1708178

EPI_ISL_1708179

EPI_ISL_1708180

EPI_ISL_1708181

EPI_ISL_1708182

EPI_ISL_1708183

EPI_ISL_1708184

EPI_ISL_2673782

EPI_ISL_2532379

EPI_ISL_2532380

EPI_ISL_2532381

EPI_ISL_2532382

EPI_ISL_2532383

EPI_ISL_1708185

EPI_ISL_2532384

EPI_ISL_2532385

EPI_ISL_2532386

EPI_ISL_2532387

EPI_ISL_1708187

EPI_ISL_1708188

EPI_ISL_1708189

EPI_ISL_1708190

EPI_ISL_1708193

EPI_ISL_1708194

EPI_ISL_1708197

EPI_ISL_1708199

EPI_ISL_1708200

EPI_ISL_1708201

EPI_ISL_1708202

EPI_ISL_1708203

EPI_ISL_1708204

EPI_ISL_1708205

EPI_ISL_1708207

EPI_ISL_1708208

EPI_ISL_1708209

EPI_ISL_1708210

EPI_ISL_1708211

EPI_ISL_1708213

EPI_ISL_1708216

EPI_ISL_1708217

EPI_ISL_1708218

EPI_ISL_1708219

EPI_ISL_1708220

EPI_ISL_1708221

EPI_ISL_1708222

EPI_ISL_1708225

EPI_ISL_1708226

EPI_ISL_1708228

EPI_ISL_1708230

EPI_ISL_1708231

EPI_ISL_1708232

EPI_ISL_1708233

EPI_ISL_1708234

EPI_ISL_1708237

EPI_ISL_1708238

EPI_ISL_2335152

EPI_ISL_1708242

EPI_ISL_2157207

EPI_ISL_1708252

EPI_ISL_2532483

EPI_ISL_1708253

EPI_ISL_1708255

EPI_ISL_1708256

EPI_ISL_1708260

EPI_ISL_2532560

EPI_ISL_2532561

EPI_ISL_2532562

EPI_ISL_2532563

EPI_ISL_2532564

EPI_ISL_2532565

EPI_ISL_2532569

EPI_ISL_2532570

EPI_ISL_2532571

EPI_ISL_2532572

EPI_ISL_2532573

EPI_ISL_2532574

EPI_ISL_2532575

EPI_ISL_2532576

EPI_ISL_2532578

EPI_ISL_2532582

EPI_ISL_2532583

EPI_ISL_2532584

EPI_ISL_2532585

EPI_ISL_1708275

EPI_ISL_2532586

EPI_ISL_2532587

EPI_ISL_1708279

EPI_ISL_2532588

EPI_ISL_2532642

EPI_ISL_2532643

EPI_ISL_2532646

EPI_ISL_2532647

EPI_ISL_2532648

EPI_ISL_1708291

EPI_ISL_1708293

EPI_ISL_1708297

EPI_ISL_1708298

EPI_ISL_1708299

EPI_ISL_1708300

EPI_ISL_1708301

EPI_ISL_1708303

EPI_ISL_1708306

EPI_ISL_1708307

EPI_ISL_1708310

EPI_ISL_1708315

EPI_ISL_1708316

EPI_ISL_2673913

EPI_ISL_2673917

EPI_ISL_2673918

EPI_ISL_2335644

EPI_ISL_2673919

EPI_ISL_2673920

EPI_ISL_2673922

EPI_ISL_2890573

EPI_ISL_2673924

EPI_ISL_2673925

EPI_ISL_2673929

EPI_ISL_2673931

EPI_ISL_3507314

EPI_ISL_3507315

EPI_ISL_3507317

EPI_ISL_3507318

EPI_ISL_3507319

EPI_ISL_3507326

EPI_ISL_3507328

EPI_ISL_3507330

EPI_ISL_3507331

EPI_ISL_3507332

EPI_ISL_3507333

EPI_ISL_3507334

EPI_ISL_3507335

EPI_ISL_3507336

EPI_ISL_3507337

EPI_ISL_3507338

EPI_ISL_3507342

EPI_ISL_3507344

EPI_ISL_2335897

EPI_ISL_1708627

EPI_ISL_3508203

EPI_ISL_3508209

EPI_ISL_3508214

EPI_ISL_3508216

EPI_ISL_3508217

EPI_ISL_3508220

EPI_ISL_2890894

EPI_ISL_2336005

EPI_ISL_3508254

EPI_ISL_3508255

EPI_ISL_3508258

EPI_ISL_3508270

EPI_ISL_3508271

EPI_ISL_3508272

EPI_ISL_3508273

EPI_ISL_3508274

EPI_ISL_3508275

EPI_ISL_3508276

EPI_ISL_3508277

EPI_ISL_3508321

EPI_ISL_3508322

EPI_ISL_3508323

EPI_ISL_2673983

EPI_ISL_3508330

EPI_ISL_3508339

EPI_ISL_3508343

EPI_ISL_3508345

EPI_ISL_3508346

EPI_ISL_3508347

EPI_ISL_3508348

EPI_ISL_3508349

EPI_ISL_3508350

EPI_ISL_3508355

EPI_ISL_3508356

EPI_ISL_3508357

EPI_ISL_3508360

EPI_ISL_3508361

EPI_ISL_3508364

EPI_ISL_3508365

EPI_ISL_3508366

EPI_ISL_3508367

EPI_ISL_3508368

EPI_ISL_3508369

EPI_ISL_3508370

EPI_ISL_3508371

EPI_ISL_3508375

EPI_ISL_3508376

EPI_ISL_3508377

EPI_ISL_3508378

EPI_ISL_3508379

EPI_ISL_3508380

EPI_ISL_3508381

EPI_ISL_3508382

EPI_ISL_3508383

EPI_ISL_3508384

EPI_ISL_3508385

EPI_ISL_3508386

EPI_ISL_3508387

EPI_ISL_3508388

EPI_ISL_3508389

EPI_ISL_3508390

EPI_ISL_3508391

EPI_ISL_3508392

EPI_ISL_3508393

EPI_ISL_3508394

EPI_ISL_3508395

EPI_ISL_3508396

EPI_ISL_3508397

EPI_ISL_3508398

EPI_ISL_3508399

EPI_ISL_3508400

EPI_ISL_3508402

EPI_ISL_3508403

EPI_ISL_3508404

EPI_ISL_3508405

EPI_ISL_3508406

EPI_ISL_3508407

EPI_ISL_3508408

EPI_ISL_3508409

EPI_ISL_3508410

EPI_ISL_3508411

EPI_ISL_3508412

EPI_ISL_3508413

EPI_ISL_3508414

EPI_ISL_3508416

EPI_ISL_3508419

EPI_ISL_3508420

EPI_ISL_3508423

EPI_ISL_3508425

EPI_ISL_3508426

EPI_ISL_3508427

EPI_ISL_2673985

EPI_ISL_3508428

EPI_ISL_2673987

EPI_ISL_2673989

EPI_ISL_2673990

EPI_ISL_2673991

EPI_ISL_2673992

EPI_ISL_3508478

EPI_ISL_3508479

EPI_ISL_3508480

EPI_ISL_3508481

EPI_ISL_3508482

EPI_ISL_2673993

EPI_ISL_3508483

EPI_ISL_3508484

EPI_ISL_3508486

EPI_ISL_3508487

EPI_ISL_3508488

EPI_ISL_1708533

EPI_ISL_1708534

EPI_ISL_1708535

EPI_ISL_1708536

EPI_ISL_1708537

EPI_ISL_1708538

EPI_ISL_1708545

EPI_ISL_2673994

EPI_ISL_2673995

EPI_ISL_1708548

EPI_ISL_1708550

EPI_ISL_1708551

EPI_ISL_1708553

EPI_ISL_1708554

EPI_ISL_1708555

EPI_ISL_1708556

EPI_ISL_2336103

EPI_ISL_3508563

EPI_ISL_1708558

EPI_ISL_1708559

EPI_ISL_3508567

EPI_ISL_3508568

EPI_ISL_3508571

EPI_ISL_3508573

EPI_ISL_3508574

EPI_ISL_3508576

EPI_ISL_3508577

EPI_ISL_2673996

EPI_ISL_3508579

EPI_ISL_3508583

EPI_ISL_3508584

EPI_ISL_3508585

EPI_ISL_3508587

EPI_ISL_3508588

EPI_ISL_3508589

EPI_ISL_3508590

EPI_ISL_3508593

EPI_ISL_3508595

EPI_ISL_3508596

EPI_ISL_1708564

EPI_ISL_3508597

EPI_ISL_3508598

EPI_ISL_3508599

EPI_ISL_3508600

EPI_ISL_3508601

EPI_ISL_2673997

EPI_ISL_1708567

EPI_ISL_1708568

EPI_ISL_1708569

EPI_ISL_1708571

EPI_ISL_3508604

EPI_ISL_1708572

EPI_ISL_3508605

EPI_ISL_3508614

EPI_ISL_1708576

EPI_ISL_2673999

EPI_ISL_3508628

EPI_ISL_3508630

EPI_ISL_3508636

EPI_ISL_1708577

EPI_ISL_1708579

EPI_ISL_1708582

EPI_ISL_1708585

EPI_ISL_3508661

EPI_ISL_1708586

EPI_ISL_3508662

EPI_ISL_3508663

EPI_ISL_3508664

EPI_ISL_1708588

EPI_ISL_1708589

EPI_ISL_3508666

EPI_ISL_3508667

EPI_ISL_3508670

EPI_ISL_3508671

EPI_ISL_3508672

EPI_ISL_3508681

EPI_ISL_3508682

EPI_ISL_2674000

EPI_ISL_1708590

EPI_ISL_3508684

EPI_ISL_3508685

EPI_ISL_3508687

EPI_ISL_1708592

EPI_ISL_1708593

EPI_ISL_3508688

EPI_ISL_2674002

EPI_ISL_3508689

EPI_ISL_3508690

EPI_ISL_1708598

EPI_ISL_1708599

EPI_ISL_1708600

EPI_ISL_3508710

EPI_ISL_2674003

EPI_ISL_3508711

EPI_ISL_3508712

EPI_ISL_3508713

EPI_ISL_3508715

EPI_ISL_3508716

EPI_ISL_3508718

EPI_ISL_3508719

EPI_ISL_3508720

EPI_ISL_3508735

EPI_ISL_3508738

EPI_ISL_3508741

EPI_ISL_2674007

EPI_ISL_3508742

EPI_ISL_3508743

EPI_ISL_3508744

EPI_ISL_3508747

EPI_ISL_3508748

EPI_ISL_3508749

EPI_ISL_2674009

EPI_ISL_2674010

EPI_ISL_2674011

EPI_ISL_3508787

EPI_ISL_3508788

EPI_ISL_3508801

EPI_ISL_3508837

EPI_ISL_3508838

EPI_ISL_2674012

EPI_ISL_3508840

EPI_ISL_3508842

EPI_ISL_3508850

EPI_ISL_1708614

EPI_ISL_3508851

EPI_ISL_3508853

EPI_ISL_3508854

EPI_ISL_2674017

EPI_ISL_2674020

EPI_ISL_1708618

EPI_ISL_2674021

EPI_ISL_1708621

EPI_ISL_2674023

EPI_ISL_1708622

EPI_ISL_3508913

EPI_ISL_3508914

EPI_ISL_3508932

EPI_ISL_3508933

EPI_ISL_3508934

EPI_ISL_3508936

EPI_ISL_3508937

EPI_ISL_3508938

EPI_ISL_3508939

EPI_ISL_3508940

EPI_ISL_2674024

EPI_ISL_3508941

EPI_ISL_3508942

EPI_ISL_3508943

EPI_ISL_3508944

EPI_ISL_3508945

EPI_ISL_3508946

EPI_ISL_3508947

EPI_ISL_3508948

EPI_ISL_3508949

EPI_ISL_2674027

EPI_ISL_2674028

EPI_ISL_1708623

EPI_ISL_3508988

EPI_ISL_3508989

EPI_ISL_3508995

EPI_ISL_3508996

EPI_ISL_3508997

EPI_ISL_3509001

EPI_ISL_2674032

EPI_ISL_1708624

EPI_ISL_1708625

EPI_ISL_2674033

EPI_ISL_2674034

EPI_ISL_2674035

EPI_ISL_1708626

EPI_ISL_2674040

EPI_ISL_2674041

EPI_ISL_2674044

EPI_ISL_2674045

EPI_ISL_2674048

EPI_ISL_2674049

EPI_ISL_2674051

EPI_ISL_1708630

EPI_ISL_2674052

EPI_ISL_2674053

EPI_ISL_2674055

EPI_ISL_2674056

EPI_ISL_2674058

EPI_ISL_1708632

EPI_ISL_1708633

EPI_ISL_1708634

EPI_ISL_1708636

EPI_ISL_1708637

EPI_ISL_2674060

EPI_ISL_2674062

EPI_ISL_2674063

EPI_ISL_2674064

EPI_ISL_1708639

EPI_ISL_1708642

EPI_ISL_1708643

EPI_ISL_1860448

EPI_ISL_1708646

EPI_ISL_1708647

EPI_ISL_1708648

EPI_ISL_1708651

EPI_ISL_1708654

EPI_ISL_2648732

EPI_ISL_1708655

EPI_ISL_1708656

EPI_ISL_1708657

EPI_ISL_1708658

EPI_ISL_1860447

EPI_ISL_1708696

EPI_ISL_1708698

EPI_ISL_1708700

EPI_ISL_1708701

EPI_ISL_1708702

EPI_ISL_1708703

EPI_ISL_1708705

EPI_ISL_1708706

EPI_ISL_1708709

EPI_ISL_1860694

EPI_ISL_1708712

EPI_ISL_1708714

EPI_ISL_1708716

EPI_ISL_1708717

EPI_ISL_1708719

EPI_ISL_1708721

EPI_ISL_1708724

EPI_ISL_1708726

EPI_ISL_1708727

EPI_ISL_1708728

EPI_ISL_1859962

EPI_ISL_5843951

EPI_ISL_1708730

EPI_ISL_4372227

EPI_ISL_1708742

EPI_ISL_5843954

EPI_ISL_1860336

EPI_ISL_2092886

EPI_ISL_1680118

EPI_ISL_1860696

EPI_ISL_5843957

EPI_ISL_2092902

EPI_ISL_1860662

EPI_ISL_1680199

EPI_ISL_1860660

EPI_ISL_5843963

EPI_ISL_5843964

EPI_ISL_2674195

EPI_ISL_2674196

EPI_ISL_2674197

EPI_ISL_2674198

EPI_ISL_1860423

EPI_ISL_5843966

EPI_ISL_1860422

EPI_ISL_1859992

EPI_ISL_2092960

EPI_ISL_2092961

EPI_ISL_2092963

EPI_ISL_5843969

EPI_ISL_5843970

EPI_ISL_1860427

EPI_ISL_5843971

EPI_ISL_2092977

EPI_ISL_1680333

EPI_ISL_2533662

EPI_ISL_5843973

EPI_ISL_2533663

EPI_ISL_1860304

EPI_ISL_5843975

EPI_ISL_1860551

EPI_ISL_1860481

EPI_ISL_2533751

EPI_ISL_1860670

EPI_ISL_2533752

EPI_ISL_2533753

EPI_ISL_1859761

EPI_ISL_2533754

EPI_ISL_2093014

EPI_ISL_5843978

EPI_ISL_2093021

EPI_ISL_2674296

EPI_ISL_1860554

EPI_ISL_1860312

EPI_ISL_2648919

EPI_ISL_2648920

EPI_ISL_1680381

EPI_ISL_1680386

EPI_ISL_5843981

EPI_ISL_5843982

EPI_ISL_1680394

EPI_ISL_2093048

EPI_ISL_2093049

EPI_ISL_2533885

EPI_ISL_1860557

EPI_ISL_2533886

EPI_ISL_2533887

EPI_ISL_2533888

EPI_ISL_2093057

EPI_ISL_5843985

EPI_ISL_5843986

EPI_ISL_1860365

EPI_ISL_1860364

EPI_ISL_5843987

EPI_ISL_1680419

EPI_ISL_1860487

EPI_ISL_5843988

EPI_ISL_2534010

EPI_ISL_2534013

EPI_ISL_2534015

EPI_ISL_2534017

EPI_ISL_1860361

EPI_ISL_2534019

EPI_ISL_2534022

EPI_ISL_5843990

EPI_ISL_5843991

EPI_ISL_1860480

EPI_ISL_1860496

EPI_ISL_2648987

EPI_ISL_2093100

EPI_ISL_1860495

EPI_ISL_5843993

EPI_ISL_5843994

EPI_ISL_2534091

EPI_ISL_2534093

EPI_ISL_2674369

EPI_ISL_2534094

EPI_ISL_2534095

EPI_ISL_2534097

EPI_ISL_2534098

EPI_ISL_5843995

EPI_ISL_2534102

EPI_ISL_2534105

EPI_ISL_2534107

EPI_ISL_2534108

EPI_ISL_2534110

EPI_ISL_2534112

EPI_ISL_2534114

EPI_ISL_2534122

EPI_ISL_5843997

EPI_ISL_2674381

EPI_ISL_2674382

EPI_ISL_1860127

EPI_ISL_2674389

EPI_ISL_2093115

EPI_ISL_5843998

EPI_ISL_2534145

EPI_ISL_2534146

EPI_ISL_5843999

EPI_ISL_5844000

EPI_ISL_2534177

EPI_ISL_2093123

EPI_ISL_2093124

EPI_ISL_5844003

EPI_ISL_2268099

EPI_ISL_5844004

EPI_ISL_1860463

EPI_ISL_2093146

EPI_ISL_5844008

EPI_ISL_3888871

EPI_ISL_5844010

EPI_ISL_5844011

EPI_ISL_5844012

EPI_ISL_1860588

EPI_ISL_5844013

EPI_ISL_1860346

EPI_ISL_2674446

EPI_ISL_2674447

EPI_ISL_2674448

EPI_ISL_2674451

EPI_ISL_5844015

EPI_ISL_2674452

EPI_ISL_2674453

EPI_ISL_2674454

EPI_ISL_2674455

EPI_ISL_2674457

EPI_ISL_2674458

EPI_ISL_5844018

EPI_ISL_5844019

EPI_ISL_2674465

EPI_ISL_3888865

EPI_ISL_2674466

EPI_ISL_2674467

EPI_ISL_2674468

EPI_ISL_2674469

EPI_ISL_5844021

EPI_ISL_2093206

EPI_ISL_2674471

EPI_ISL_2674473

EPI_ISL_2674475

EPI_ISL_2674476

EPI_ISL_2674477

EPI_ISL_5844024

EPI_ISL_2674478

EPI_ISL_2674479

EPI_ISL_2674481

EPI_ISL_2674484

EPI_ISL_2674485

EPI_ISL_5844026

EPI_ISL_2674487

EPI_ISL_5844027

EPI_ISL_2674489

EPI_ISL_2674490

EPI_ISL_2674491

EPI_ISL_2674492

EPI_ISL_2674493

EPI_ISL_2674494

EPI_ISL_3888860

EPI_ISL_2674497

EPI_ISL_5844028

EPI_ISL_5844029

EPI_ISL_5844030

EPI_ISL_2674499

EPI_ISL_5844032

EPI_ISL_2674500

EPI_ISL_5844034

EPI_ISL_5844035

EPI_ISL_1860355

EPI_ISL_5844038

EPI_ISL_5844039

EPI_ISL_1860592

EPI_ISL_2649121

EPI_ISL_2674502

EPI_ISL_5844040

EPI_ISL_2674503

EPI_ISL_2674504

EPI_ISL_2674505

EPI_ISL_2674508

EPI_ISL_1860590

EPI_ISL_2674509

EPI_ISL_5844041

EPI_ISL_5844042

EPI_ISL_2674510

EPI_ISL_2674511

EPI_ISL_2674512

EPI_ISL_2674514

EPI_ISL_2674515

EPI_ISL_2674516

EPI_ISL_2674517

EPI_ISL_2093278

EPI_ISL_2093279

EPI_ISL_5844043

EPI_ISL_5844044

EPI_ISL_1860280

EPI_ISL_5844045

EPI_ISL_2649147

EPI_ISL_5844046

EPI_ISL_2093285

EPI_ISL_2649149

EPI_ISL_1860046

EPI_ISL_5844047

EPI_ISL_1860296

EPI_ISL_5844049

EPI_ISL_5844050

EPI_ISL_5844052

EPI_ISL_1860266

EPI_ISL_6166385

EPI_ISL_1860383

EPI_ISL_1860261

EPI_ISL_5844089

EPI_ISL_2649208

EPI_ISL_5844090

EPI_ISL_5844091

EPI_ISL_2093348

EPI_ISL_1860277

EPI_ISL_1860397

EPI_ISL_1860173

EPI_ISL_5844106

EPI_ISL_2649233

EPI_ISL_1682557

EPI_ISL_2093385

EPI_ISL_1860154

EPI_ISL_2535302

EPI_ISL_2535310

EPI_ISL_2649258

EPI_ISL_2535311

EPI_ISL_2535312

EPI_ISL_2535313

EPI_ISL_2535314

EPI_ISL_2535315

EPI_ISL_2535316

EPI_ISL_2535317

EPI_ISL_2535320

EPI_ISL_2535321

EPI_ISL_2535322

EPI_ISL_2535323

EPI_ISL_2535325

EPI_ISL_2535326

EPI_ISL_2535328

EPI_ISL_2535329

EPI_ISL_2535330

EPI_ISL_2535331

EPI_ISL_2535332

EPI_ISL_2535333

EPI_ISL_2535334

EPI_ISL_2535335

EPI_ISL_2535336

EPI_ISL_2535344

EPI_ISL_1860080

EPI_ISL_5844159

EPI_ISL_2535347

EPI_ISL_2535348

EPI_ISL_1860099

EPI_ISL_2535349

EPI_ISL_2535350

EPI_ISL_2535353

EPI_ISL_2535356

EPI_ISL_2535357

EPI_ISL_2535370

EPI_ISL_1860187

EPI_ISL_2535387

EPI_ISL_2535397

EPI_ISL_2535398

EPI_ISL_2535400

EPI_ISL_2535402

EPI_ISL_1709848

EPI_ISL_2535403

EPI_ISL_2535404

EPI_ISL_2093422

EPI_ISL_1709855

EPI_ISL_1709858

EPI_ISL_1709859

EPI_ISL_1709860

EPI_ISL_2093427

EPI_ISL_1709862

EPI_ISL_1682637

EPI_ISL_1709867

EPI_ISL_1709869

EPI_ISL_5844184

EPI_ISL_5365743

EPI_ISL_5365744

EPI_ISL_1709874

EPI_ISL_1860198

EPI_ISL_1709876

EPI_ISL_1709878

EPI_ISL_5365747

EPI_ISL_1709880

EPI_ISL_1709883

EPI_ISL_1709884

EPI_ISL_5844186

EPI_ISL_2093441

EPI_ISL_1709890

EPI_ISL_2271428

EPI_ISL_1709893

EPI_ISL_1709895

EPI_ISL_1709896

EPI_ISL_1709898

EPI_ISL_1709899

EPI_ISL_1709900

EPI_ISL_2535471

EPI_ISL_1709901

EPI_ISL_2093443

EPI_ISL_1709902

EPI_ISL_5365773

EPI_ISL_1709904

EPI_ISL_1709914

EPI_ISL_5844188

EPI_ISL_1709916

EPI_ISL_1709918

EPI_ISL_1709921

EPI_ISL_1709922

EPI_ISL_2271438

EPI_ISL_1709925

EPI_ISL_1709926

EPI_ISL_1709927

EPI_ISL_1709928

EPI_ISL_1709929

EPI_ISL_1709930

EPI_ISL_1709931

EPI_ISL_2271441

EPI_ISL_1709934

EPI_ISL_5844191

EPI_ISL_5844192

EPI_ISL_1709943

EPI_ISL_2093457

EPI_ISL_1709948

EPI_ISL_1709949

EPI_ISL_1709951

EPI_ISL_5844195

EPI_ISL_1709963

EPI_ISL_1709968

EPI_ISL_1709969

EPI_ISL_5844197

EPI_ISL_1709973

EPI_ISL_1709974

EPI_ISL_1709976

EPI_ISL_1709977

EPI_ISL_1709982

EPI_ISL_1709984

EPI_ISL_1709987

EPI_ISL_1709989

EPI_ISL_2271464

EPI_ISL_1709994

EPI_ISL_5844198

EPI_ISL_1710004

EPI_ISL_1710005

EPI_ISL_1710006

EPI_ISL_5844201

EPI_ISL_2093487

EPI_ISL_1710015

EPI_ISL_1710016

EPI_ISL_1710017

EPI_ISL_1710018

EPI_ISL_1710019

EPI_ISL_1710021

EPI_ISL_1710022

EPI_ISL_1710023

EPI_ISL_1682733

EPI_ISL_1710024

EPI_ISL_1710025

EPI_ISL_1710026

EPI_ISL_1710027

EPI_ISL_1710028

EPI_ISL_5844202

EPI_ISL_5844203

EPI_ISL_1710029

EPI_ISL_1710030

EPI_ISL_1710031

EPI_ISL_1710032

EPI_ISL_1710033

EPI_ISL_1710034

EPI_ISL_1710035

EPI_ISL_1710036

EPI_ISL_1710037

EPI_ISL_1710038

EPI_ISL_1710039

EPI_ISL_1710040

EPI_ISL_2271479

EPI_ISL_1710041

EPI_ISL_1710042

EPI_ISL_1710043

EPI_ISL_1710044

EPI_ISL_1710045

EPI_ISL_1710046

EPI_ISL_1710047

EPI_ISL_5844205

EPI_ISL_2271494

EPI_ISL_5844206

EPI_ISL_2674720

EPI_ISL_2674721

EPI_ISL_5844207

EPI_ISL_2271497

EPI_ISL_2674722

EPI_ISL_2674723

EPI_ISL_5844208

EPI_ISL_2674724

EPI_ISL_2674725

EPI_ISL_2674726

EPI_ISL_2674727

EPI_ISL_1866309

EPI_ISL_2674728

EPI_ISL_2674729

EPI_ISL_5844209

EPI_ISL_2674730

EPI_ISL_5844210

EPI_ISL_5366166

EPI_ISL_5366167

EPI_ISL_5366168

EPI_ISL_5366170

EPI_ISL_5366171

EPI_ISL_5844211

EPI_ISL_2271511

EPI_ISL_2271512

EPI_ISL_5366176

EPI_ISL_5366181

EPI_ISL_5366182

EPI_ISL_2674731

EPI_ISL_2674732

EPI_ISL_2674733

EPI_ISL_2674734

EPI_ISL_5366183

EPI_ISL_5366185

EPI_ISL_5366190

EPI_ISL_5366191

EPI_ISL_5366192

EPI_ISL_5844212

EPI_ISL_5366193

EPI_ISL_2674735

EPI_ISL_5366194

EPI_ISL_5366199

EPI_ISL_2093503

EPI_ISL_2535792

EPI_ISL_5366200

EPI_ISL_5366201

EPI_ISL_5366206

EPI_ISL_5366207

EPI_ISL_5366208

EPI_ISL_5366209

EPI_ISL_5366213

EPI_ISL_2271522

EPI_ISL_5844214

EPI_ISL_1866305

EPI_ISL_5366214

EPI_ISL_5366215

EPI_ISL_5366216

EPI_ISL_5844215

EPI_ISL_2271529

EPI_ISL_2271532

EPI_ISL_5844217

EPI_ISL_1859508

EPI_ISL_2649413

EPI_ISL_2271539

EPI_ISL_2093526

EPI_ISL_1682816

EPI_ISL_2649417

EPI_ISL_2649418

EPI_ISL_5366340

EPI_ISL_2093534

EPI_ISL_5366356

EPI_ISL_5844224

EPI_ISL_5844227

EPI_ISL_2536041

EPI_ISL_2536042

EPI_ISL_5366431

EPI_ISL_5366432

EPI_ISL_2536043

EPI_ISL_2536045

EPI_ISL_5366441

EPI_ISL_2536046

EPI_ISL_5844228

EPI_ISL_2674812

EPI_ISL_2674813

EPI_ISL_1859655

EPI_ISL_1866430

EPI_ISL_2674814

EPI_ISL_5844229

EPI_ISL_2271576

EPI_ISL_2674815

EPI_ISL_2674816

EPI_ISL_2674817

EPI_ISL_2674818

EPI_ISL_5844231

EPI_ISL_2536110

EPI_ISL_1866408

EPI_ISL_2674819

EPI_ISL_2674820

EPI_ISL_2674821

EPI_ISL_5844232

EPI_ISL_2536132

EPI_ISL_2674822

EPI_ISL_2674823

EPI_ISL_1859419

EPI_ISL_2674824

EPI_ISL_5844233

EPI_ISL_5844234

EPI_ISL_2674825

EPI_ISL_2674826

EPI_ISL_2674827

EPI_ISL_2674828

EPI_ISL_1866401

EPI_ISL_5844235

EPI_ISL_2674829

EPI_ISL_2674831

EPI_ISL_5844236

EPI_ISL_1866416

EPI_ISL_5844237

EPI_ISL_2093587

EPI_ISL_5844238

EPI_ISL_5844240

EPI_ISL_2674837

EPI_ISL_2674838

EPI_ISL_2674839

EPI_ISL_2674840

EPI_ISL_2093598

EPI_ISL_5844241

EPI_ISL_2674841

EPI_ISL_2674842

EPI_ISL_2674843

EPI_ISL_2674844

EPI_ISL_2674845

EPI_ISL_2674846

EPI_ISL_2674847

EPI_ISL_2674848

EPI_ISL_2674849

EPI_ISL_2674850

EPI_ISL_2674851

EPI_ISL_2674852

EPI_ISL_2674853

EPI_ISL_1859306

EPI_ISL_1859548

EPI_ISL_1866228

EPI_ISL_2674854

EPI_ISL_2674855

EPI_ISL_2093609

EPI_ISL_5844243

EPI_ISL_2674856

EPI_ISL_2674857

EPI_ISL_5844242

EPI_ISL_2674858

EPI_ISL_2674860

EPI_ISL_5844244

EPI_ISL_2674862

EPI_ISL_2093611

EPI_ISL_5844245

EPI_ISL_2536376

EPI_ISL_2674863

EPI_ISL_2674864

EPI_ISL_2674865

EPI_ISL_2674866

EPI_ISL_2536378

EPI_ISL_2536380

EPI_ISL_5844246

EPI_ISL_2536383

EPI_ISL_2093617

EPI_ISL_2536389

EPI_ISL_2536394

EPI_ISL_2674867

EPI_ISL_2536395

EPI_ISL_1866344

EPI_ISL_2536397

EPI_ISL_2536398

EPI_ISL_2536402

EPI_ISL_2674868

EPI_ISL_2674870

EPI_ISL_2674871

EPI_ISL_2674872

EPI_ISL_2536405

EPI_ISL_2536410

EPI_ISL_2536411

EPI_ISL_2536412

EPI_ISL_2536413

EPI_ISL_2536417

EPI_ISL_2093621

EPI_ISL_2536419

EPI_ISL_5844248

EPI_ISL_2674873

EPI_ISL_2536426

EPI_ISL_2674874

EPI_ISL_2674875

EPI_ISL_2536441

EPI_ISL_2536442

EPI_ISL_2536443

EPI_ISL_5844250

EPI_ISL_2674876

EPI_ISL_2674877

EPI_ISL_2674878

EPI_ISL_2674879

EPI_ISL_2674880

EPI_ISL_2674881

EPI_ISL_5844252

EPI_ISL_2674882

EPI_ISL_2674883

EPI_ISL_2674885

EPI_ISL_2093640

EPI_ISL_1859644

EPI_ISL_1866464

EPI_ISL_5844253

EPI_ISL_2674886

EPI_ISL_2674887

EPI_ISL_2674888

EPI_ISL_2536510

EPI_ISL_2536516

EPI_ISL_5844254

EPI_ISL_2536528

EPI_ISL_2536530

EPI_ISL_2536532

EPI_ISL_2674890

EPI_ISL_2674892

EPI_ISL_1866462

EPI_ISL_2536540

EPI_ISL_2536541

EPI_ISL_2093651

EPI_ISL_2093652

EPI_ISL_2674893

EPI_ISL_2674895

EPI_ISL_1710468

EPI_ISL_2674896

EPI_ISL_1866238

EPI_ISL_5844258

EPI_ISL_5844259

EPI_ISL_1866357

EPI_ISL_5844260

EPI_ISL_5844261

EPI_ISL_5844262

EPI_ISL_2536656

EPI_ISL_2536659

EPI_ISL_2536660

EPI_ISL_2536664

EPI_ISL_2536666

EPI_ISL_1866354

EPI_ISL_5844265

EPI_ISL_2536678

EPI_ISL_2536680

EPI_ISL_2536683

EPI_ISL_1866351

EPI_ISL_5844266

EPI_ISL_2536697

EPI_ISL_2536701

EPI_ISL_5844267

EPI_ISL_2093693

EPI_ISL_2093695

EPI_ISL_1866207

EPI_ISL_1866328

EPI_ISL_5844270

EPI_ISL_5844271

EPI_ISL_2093708

EPI_ISL_2093709

EPI_ISL_5844273

EPI_ISL_1710577

EPI_ISL_1710578

EPI_ISL_1710579

EPI_ISL_1710583

EPI_ISL_1859221

EPI_ISL_1866206

EPI_ISL_1710585

EPI_ISL_1710586

EPI_ISL_1710587

EPI_ISL_1710589

EPI_ISL_1710590

EPI_ISL_1866324

EPI_ISL_1866445

EPI_ISL_1710594

EPI_ISL_1710595

EPI_ISL_1710596

EPI_ISL_1710597

EPI_ISL_1866204

EPI_ISL_1710637

EPI_ISL_1710638

EPI_ISL_1710639

EPI_ISL_1710643

EPI_ISL_1710644

EPI_ISL_1710645

EPI_ISL_1710646

EPI_ISL_1710647

EPI_ISL_1866440

EPI_ISL_2536909

EPI_ISL_1710674

EPI_ISL_1859580

EPI_ISL_1710683

EPI_ISL_1710684

EPI_ISL_1710685

EPI_ISL_1710686

EPI_ISL_1710687

EPI_ISL_1710688

EPI_ISL_1710689

EPI_ISL_1710690

EPI_ISL_1710691

EPI_ISL_1866214

EPI_ISL_1859108

EPI_ISL_1866215

EPI_ISL_1710717

EPI_ISL_1710722

EPI_ISL_1712213

EPI_ISL_1712214

EPI_ISL_2093774

EPI_ISL_1712215

EPI_ISL_1712216

EPI_ISL_1712217

EPI_ISL_1712219

EPI_ISL_1712220

EPI_ISL_1712221

EPI_ISL_1712222

EPI_ISL_1712223

EPI_ISL_1712224

EPI_ISL_1712225

EPI_ISL_1866330

EPI_ISL_1712229

EPI_ISL_1859557

EPI_ISL_1712231

EPI_ISL_1712232

EPI_ISL_1712233

EPI_ISL_1712234

EPI_ISL_1712235

EPI_ISL_1712236

EPI_ISL_1712238

EPI_ISL_1712239

EPI_ISL_1712240

EPI_ISL_1710872

EPI_ISL_1710884

EPI_ISL_1710885

EPI_ISL_1710886

EPI_ISL_1710888

EPI_ISL_5844300

EPI_ISL_1866276

EPI_ISL_1710913

EPI_ISL_1710914

EPI_ISL_1710920

EPI_ISL_1710925

EPI_ISL_1866398

EPI_ISL_5844303

EPI_ISL_1710929

EPI_ISL_1710931

EPI_ISL_1710935

EPI_ISL_1710941

EPI_ISL_1710942

EPI_ISL_1710947

EPI_ISL_1710949

EPI_ISL_1710952

EPI_ISL_1710954

EPI_ISL_1710955

EPI_ISL_1859497

EPI_ISL_1710956

EPI_ISL_1710961

EPI_ISL_1710962

EPI_ISL_1859255

EPI_ISL_2537364

EPI_ISL_2537366

EPI_ISL_1710986

EPI_ISL_1866273

EPI_ISL_1710987

EPI_ISL_1710988

EPI_ISL_1710989

EPI_ISL_1710990

EPI_ISL_1710991

EPI_ISL_1710992

EPI_ISL_1710993

EPI_ISL_1710994

EPI_ISL_1710995

EPI_ISL_1710996

EPI_ISL_1710997

EPI_ISL_1710998

EPI_ISL_1710999

EPI_ISL_1866247

EPI_ISL_1711000

EPI_ISL_1711001

EPI_ISL_1711002

EPI_ISL_5844305

EPI_ISL_2537400

EPI_ISL_2537401

EPI_ISL_2537402

EPI_ISL_1711003

EPI_ISL_1711005

EPI_ISL_1711006

EPI_ISL_2537404

EPI_ISL_1711007

EPI_ISL_1866248

EPI_ISL_1711008

EPI_ISL_1711009

EPI_ISL_1711010

EPI_ISL_1711011

EPI_ISL_1711012

EPI_ISL_1711013

EPI_ISL_1711014

EPI_ISL_1711015

EPI_ISL_1711016

EPI_ISL_1711017

EPI_ISL_1711018

EPI_ISL_1711019

EPI_ISL_1711020

EPI_ISL_1711021

EPI_ISL_1711022

EPI_ISL_1711023

EPI_ISL_2537415

EPI_ISL_1711024

EPI_ISL_2537416

EPI_ISL_1711025

EPI_ISL_2537418

EPI_ISL_1711026

EPI_ISL_1711027

EPI_ISL_1711028

EPI_ISL_1711029

EPI_ISL_1711030

EPI_ISL_1711032

EPI_ISL_5844313

EPI_ISL_1711049

EPI_ISL_2537440

EPI_ISL_2537441

EPI_ISL_1711057

EPI_ISL_1711059

EPI_ISL_2537451

EPI_ISL_2537452

EPI_ISL_2537453

EPI_ISL_1711065

EPI_ISL_5844317

EPI_ISL_5844318

EPI_ISL_5844319

EPI_ISL_5844320

EPI_ISL_1711097

EPI_ISL_1711104

EPI_ISL_1711105

EPI_ISL_1711107

EPI_ISL_1711108

EPI_ISL_1711109

EPI_ISL_1711110

EPI_ISL_1711113

EPI_ISL_1711115

EPI_ISL_1711118

EPI_ISL_1711119

EPI_ISL_1866256

EPI_ISL_1866378

EPI_ISL_5844324

EPI_ISL_2675160

EPI_ISL_2675161

EPI_ISL_2675163

EPI_ISL_2649805

EPI_ISL_2675164

EPI_ISL_2675165

EPI_ISL_2675167

EPI_ISL_2675168

EPI_ISL_2675169

EPI_ISL_2675170

EPI_ISL_2675171

EPI_ISL_2675172

EPI_ISL_2675175

EPI_ISL_2675176

EPI_ISL_2675178

EPI_ISL_1866252

EPI_ISL_5844327

EPI_ISL_2675179

EPI_ISL_2675180

EPI_ISL_2675181

EPI_ISL_2675182

EPI_ISL_2675183

EPI_ISL_2675184

EPI_ISL_5844329

EPI_ISL_1893798

EPI_ISL_2675186

EPI_ISL_2675187

EPI_ISL_5844330

EPI_ISL_2093924

EPI_ISL_2675188

EPI_ISL_5844331

EPI_ISL_1893800

EPI_ISL_5844335

EPI_ISL_1893802

EPI_ISL_1866292

EPI_ISL_5844337

EPI_ISL_5844338

EPI_ISL_1866296

EPI_ISL_5844339

EPI_ISL_1866297

EPI_ISL_5844340

EPI_ISL_1711298

EPI_ISL_1711299

EPI_ISL_1711300

EPI_ISL_1711301

EPI_ISL_1711302

EPI_ISL_1859263

EPI_ISL_1893820

EPI_ISL_2093975

EPI_ISL_1859597

EPI_ISL_1711351

EPI_ISL_1711352

EPI_ISL_1711353

EPI_ISL_1711356

EPI_ISL_1711358

EPI_ISL_1711360

EPI_ISL_1711361

EPI_ISL_1711362

EPI_ISL_5844347

EPI_ISL_1711363

EPI_ISL_1711364

EPI_ISL_1711365

EPI_ISL_1711366

EPI_ISL_5844348

EPI_ISL_1859236

EPI_ISL_1859239

EPI_ISL_5844353

EPI_ISL_5844356

EPI_ISL_5844357

EPI_ISL_2094018

EPI_ISL_1859246

EPI_ISL_1893879

EPI_ISL_5844358

EPI_ISL_5844361

EPI_ISL_5844362

EPI_ISL_2675304

EPI_ISL_1893886

EPI_ISL_4371600

EPI_ISL_5844365

EPI_ISL_5844367

EPI_ISL_1711522

EPI_ISL_1711523

EPI_ISL_1711528

EPI_ISL_1711529

EPI_ISL_5844369

EPI_ISL_1711531

EPI_ISL_5844370

EPI_ISL_5844372

EPI_ISL_5844373

EPI_ISL_2094070

EPI_ISL_5844374

EPI_ISL_5844376

EPI_ISL_5844377

EPI_ISL_5844378

EPI_ISL_1859398

EPI_ISL_1712375

EPI_ISL_1712376

EPI_ISL_2094103

EPI_ISL_5844384

EPI_ISL_5844385

EPI_ISL_5844387

EPI_ISL_1859150

EPI_ISL_1712377

EPI_ISL_1711677

EPI_ISL_1711693

EPI_ISL_1711696

EPI_ISL_1711698

EPI_ISL_1711699

EPI_ISL_1711701

EPI_ISL_1711702

EPI_ISL_1711703

EPI_ISL_1711719

EPI_ISL_1711737

EPI_ISL_1711743

EPI_ISL_1711748

EPI_ISL_1711749

EPI_ISL_1712325

EPI_ISL_1711771

EPI_ISL_1711772

EPI_ISL_1711774

EPI_ISL_1711785

EPI_ISL_1711787

EPI_ISL_1711790

EPI_ISL_2094170

EPI_ISL_1711796

EPI_ISL_2094175

EPI_ISL_1711805

EPI_ISL_1711809

EPI_ISL_1711811

EPI_ISL_1711814

EPI_ISL_1711815

EPI_ISL_1711816

EPI_ISL_1711817

EPI_ISL_1711821

EPI_ISL_1711822

EPI_ISL_1711823

EPI_ISL_1711824

EPI_ISL_1711826

EPI_ISL_1711827

EPI_ISL_1711829

EPI_ISL_1711830

EPI_ISL_1711831

EPI_ISL_1711832

EPI_ISL_1711834

EPI_ISL_1711835

EPI_ISL_1711836

EPI_ISL_1711838

EPI_ISL_2444733

EPI_ISL_1711840

EPI_ISL_1711842

EPI_ISL_1711845

EPI_ISL_2444742

EPI_ISL_2094204

EPI_ISL_2094207

EPI_ISL_1894426

EPI_ISL_1711920

EPI_ISL_1711922

EPI_ISL_1711924

EPI_ISL_1711940

EPI_ISL_1711949

EPI_ISL_2094231

EPI_ISL_2094232

EPI_ISL_1712348

EPI_ISL_1712349

EPI_ISL_2094235

EPI_ISL_5366172

EPI_ISL_5366184

EPI_ISL_1865335

EPI_ISL_1711976

EPI_ISL_1711977

EPI_ISL_1711978

EPI_ISL_1711979

EPI_ISL_1711980

EPI_ISL_1711981

EPI_ISL_1711982

EPI_ISL_1711983

EPI_ISL_1711984

EPI_ISL_1711985

EPI_ISL_1711986

EPI_ISL_1711987

EPI_ISL_1711988

EPI_ISL_1711989

EPI_ISL_1711990

EPI_ISL_1711991

EPI_ISL_1711992

EPI_ISL_1711993

EPI_ISL_1711994

EPI_ISL_1711995

EPI_ISL_1711996

EPI_ISL_1711997

EPI_ISL_1711999

EPI_ISL_1712000

EPI_ISL_1712001

EPI_ISL_1712002

EPI_ISL_1712003

EPI_ISL_1712004

EPI_ISL_1712005

EPI_ISL_1712006

EPI_ISL_1712008

EPI_ISL_1712009

EPI_ISL_1712010

EPI_ISL_1712011

EPI_ISL_1712012

EPI_ISL_1712013

EPI_ISL_1712014

EPI_ISL_1712015

EPI_ISL_1712016

EPI_ISL_1865216

EPI_ISL_1712017

EPI_ISL_1712018

EPI_ISL_1712020

EPI_ISL_1712022

EPI_ISL_1712023

EPI_ISL_1712026

EPI_ISL_1712027

EPI_ISL_1712028

EPI_ISL_1712029

EPI_ISL_1712030

EPI_ISL_1712031

EPI_ISL_1712033

EPI_ISL_1712034

EPI_ISL_1712035

EPI_ISL_1712036

EPI_ISL_1712037

EPI_ISL_1712040

EPI_ISL_1712041

EPI_ISL_1712043

EPI_ISL_1712044

EPI_ISL_1712045

EPI_ISL_1712046

EPI_ISL_1712047

EPI_ISL_1712049

EPI_ISL_1712050

EPI_ISL_1712051

EPI_ISL_1712054

EPI_ISL_1712056

EPI_ISL_1712057

EPI_ISL_1712058

EPI_ISL_1712059

EPI_ISL_1712060

EPI_ISL_1712061

EPI_ISL_1712063

EPI_ISL_1712064

EPI_ISL_1712065

EPI_ISL_1712066

EPI_ISL_1712067

EPI_ISL_1712068

EPI_ISL_1712070

EPI_ISL_1712071

EPI_ISL_1712072

EPI_ISL_1712073

EPI_ISL_1712074

EPI_ISL_1712075

EPI_ISL_1712076

EPI_ISL_1712077

EPI_ISL_1712078

EPI_ISL_1712079

EPI_ISL_1865693

EPI_ISL_1712080

EPI_ISL_1712081

EPI_ISL_1712082

EPI_ISL_1712083

EPI_ISL_1712084

EPI_ISL_1712085

EPI_ISL_2675581

EPI_ISL_1712086

EPI_ISL_1712087

EPI_ISL_1712088

EPI_ISL_1712089

EPI_ISL_1712090

EPI_ISL_1712091

EPI_ISL_1712092

EPI_ISL_1712093

EPI_ISL_1712094

EPI_ISL_1712095

EPI_ISL_1712098

EPI_ISL_1712100

EPI_ISL_1712101

EPI_ISL_1712102

EPI_ISL_1712103

EPI_ISL_1712104

EPI_ISL_1712105

EPI_ISL_1712106

EPI_ISL_1712107

EPI_ISL_1712108

EPI_ISL_1712109

EPI_ISL_1712110

EPI_ISL_1712111

EPI_ISL_1712113

EPI_ISL_1712115

EPI_ISL_1712116

EPI_ISL_1712117

EPI_ISL_1712119

EPI_ISL_1712120

EPI_ISL_1712121

EPI_ISL_1712122

EPI_ISL_1712123

EPI_ISL_1712124

EPI_ISL_1712125

EPI_ISL_1712126

EPI_ISL_1712127

EPI_ISL_1712128

EPI_ISL_1712129

EPI_ISL_1712130

EPI_ISL_1712131

EPI_ISL_1712132

EPI_ISL_1712133

EPI_ISL_1712134

EPI_ISL_1712135

EPI_ISL_1712136

EPI_ISL_1712137

EPI_ISL_1712138

EPI_ISL_1865144

EPI_ISL_1712139

EPI_ISL_1712140

EPI_ISL_1712141

EPI_ISL_1712142

EPI_ISL_1712143

EPI_ISL_1712144

EPI_ISL_1712145

EPI_ISL_1712146

EPI_ISL_1712148

EPI_ISL_1712149

EPI_ISL_1712150

EPI_ISL_1712151

EPI_ISL_1712152

EPI_ISL_1712153

EPI_ISL_1712154

EPI_ISL_1712156

EPI_ISL_1712157

EPI_ISL_1712158

EPI_ISL_1712159

EPI_ISL_1712160

EPI_ISL_1712161

EPI_ISL_1712162

EPI_ISL_1712163

EPI_ISL_1712165

EPI_ISL_1712166

EPI_ISL_1712167

EPI_ISL_1712168

EPI_ISL_1712169

EPI_ISL_1865212

EPI_ISL_1865695

EPI_ISL_1712171

EPI_ISL_1712172

EPI_ISL_1712173

EPI_ISL_1712174

EPI_ISL_1712175

EPI_ISL_1712176

EPI_ISL_1712177

EPI_ISL_1712179

EPI_ISL_1712180

EPI_ISL_1712181

EPI_ISL_1712182

EPI_ISL_2675598

EPI_ISL_1712183

EPI_ISL_1712184

EPI_ISL_1712185

EPI_ISL_1712186

EPI_ISL_1865145

EPI_ISL_1712190

EPI_ISL_1712191

EPI_ISL_1712193

EPI_ISL_1712196

EPI_ISL_1712197

EPI_ISL_1712198

EPI_ISL_1712200

EPI_ISL_1712201

EPI_ISL_1712202

EPI_ISL_1712204

EPI_ISL_1712205

EPI_ISL_1712207

EPI_ISL_1712208

EPI_ISL_1712209

EPI_ISL_1712210

EPI_ISL_1865450

EPI_ISL_2094318

EPI_ISL_1865346

EPI_ISL_3356728

EPI_ISL_1865587

EPI_ISL_1865468

EPI_ISL_1865342

EPI_ISL_1712536

EPI_ISL_3888858

EPI_ISL_1865221

EPI_ISL_1712541

EPI_ISL_3888856

EPI_ISL_1712553

EPI_ISL_1712554

EPI_ISL_1712555

EPI_ISL_3888854

EPI_ISL_1865171

EPI_ISL_1712556

EPI_ISL_1712558

EPI_ISL_1865343

EPI_ISL_1865464

EPI_ISL_3888850

EPI_ISL_1865461

EPI_ISL_1712597

EPI_ISL_2675742

EPI_ISL_1712600

EPI_ISL_1712602

EPI_ISL_1712603

EPI_ISL_1712605

EPI_ISL_1712606

EPI_ISL_1712607

EPI_ISL_1865173

EPI_ISL_1865438

EPI_ISL_1712610

EPI_ISL_1712611

EPI_ISL_1712612

EPI_ISL_1712615

EPI_ISL_1712616

EPI_ISL_1712617

EPI_ISL_1712618

EPI_ISL_1712619

EPI_ISL_1712620

EPI_ISL_1712624

EPI_ISL_1712625

EPI_ISL_1712628

EPI_ISL_1712629

EPI_ISL_1865316

EPI_ISL_1712630

EPI_ISL_1712631

EPI_ISL_3356777

EPI_ISL_1865439

EPI_ISL_1865676

EPI_ISL_1865187

EPI_ISL_2271592

EPI_ISL_1865430

EPI_ISL_2675817

EPI_ISL_1865311

EPI_ISL_1865431

EPI_ISL_1865328

EPI_ISL_2271614

EPI_ISL_7068173

EPI_ISL_7068190

EPI_ISL_2675861

EPI_ISL_7068204

EPI_ISL_7068215

EPI_ISL_7068224

EPI_ISL_7068245

EPI_ISL_7068250

EPI_ISL_7068282

EPI_ISL_7068290

EPI_ISL_7068305

EPI_ISL_7068314

EPI_ISL_7068323

EPI_ISL_2675881

EPI_ISL_7068357

EPI_ISL_7068365

EPI_ISL_7068370

EPI_ISL_7068386

EPI_ISL_7068393

EPI_ISL_7068399

EPI_ISL_7068409

EPI_ISL_7068419

EPI_ISL_7068427

EPI_ISL_1865164

EPI_ISL_1865565

EPI_ISL_7068432

EPI_ISL_7068471

EPI_ISL_7068510

EPI_ISL_7068514

EPI_ISL_7068519

EPI_ISL_7068526

EPI_ISL_7068552

EPI_ISL_7068556

EPI_ISL_7068569

EPI_ISL_7068583

EPI_ISL_7068590

EPI_ISL_7068594

EPI_ISL_7068603

EPI_ISL_7068624

EPI_ISL_7068634

EPI_ISL_7068648

EPI_ISL_7068653

EPI_ISL_7068680

EPI_ISL_7068687

EPI_ISL_7068696

EPI_ISL_7068704

EPI_ISL_7068725

EPI_ISL_7068738

EPI_ISL_7068757

EPI_ISL_7068776

EPI_ISL_7068782

EPI_ISL_7068807

EPI_ISL_7068831

EPI_ISL_7068856

EPI_ISL_7068879

EPI_ISL_7068894

EPI_ISL_7068909

EPI_ISL_7068910

EPI_ISL_7068914

EPI_ISL_7068922

EPI_ISL_7068941

EPI_ISL_7068966

EPI_ISL_7068991

EPI_ISL_7068998

EPI_ISL_7069005

EPI_ISL_7069010

EPI_ISL_7069074

EPI_ISL_7069075

EPI_ISL_7069084

EPI_ISL_7069090

EPI_ISL_7069097

EPI_ISL_7069110

EPI_ISL_7069127

EPI_ISL_7069132

EPI_ISL_7069154

EPI_ISL_7069173

EPI_ISL_7069175

EPI_ISL_7069202

EPI_ISL_7069218

EPI_ISL_7069225

EPI_ISL_7069233

EPI_ISL_7069248

EPI_ISL_7069255

EPI_ISL_1865254

EPI_ISL_7069274

EPI_ISL_7069291

EPI_ISL_7069302

EPI_ISL_7069308

EPI_ISL_7069313

EPI_ISL_7069327

EPI_ISL_7069356

EPI_ISL_7069374

EPI_ISL_7069393

EPI_ISL_7069431

EPI_ISL_7069444

EPI_ISL_7069455

EPI_ISL_1865371

EPI_ISL_7069481

EPI_ISL_7069489

EPI_ISL_7069493

EPI_ISL_7069509

EPI_ISL_7069524

EPI_ISL_7069544

EPI_ISL_7069562

EPI_ISL_2271622

EPI_ISL_1865385

EPI_ISL_7070013

EPI_ISL_1865239

EPI_ISL_7070022

EPI_ISL_1865236

EPI_ISL_7070081

EPI_ISL_7070090

EPI_ISL_7070096

EPI_ISL_7070127

EPI_ISL_7070136

EPI_ISL_7070145

EPI_ISL_7070156

EPI_ISL_7070167

EPI_ISL_7070177

EPI_ISL_1865235

EPI_ISL_7070188

EPI_ISL_7070207

EPI_ISL_7070217

EPI_ISL_7070223

EPI_ISL_1865237

EPI_ISL_1865358

EPI_ISL_7070260

EPI_ISL_7070266

EPI_ISL_7070276

EPI_ISL_7070282

EPI_ISL_7070291

EPI_ISL_7070298

EPI_ISL_7070305

EPI_ISL_7070330

EPI_ISL_7070339

EPI_ISL_7070350

EPI_ISL_7070364

EPI_ISL_7070371

EPI_ISL_7070377

EPI_ISL_7070386

EPI_ISL_7070398

EPI_ISL_7070407

EPI_ISL_7070410

EPI_ISL_2094648

EPI_ISL_7070416

EPI_ISL_7070429

EPI_ISL_7070433

EPI_ISL_7070442

EPI_ISL_7070456

EPI_ISL_1865476

EPI_ISL_7070471

EPI_ISL_7070479

EPI_ISL_7070484

EPI_ISL_7070499

EPI_ISL_7070507

EPI_ISL_7070512

EPI_ISL_7070534

EPI_ISL_7070539

EPI_ISL_7070552

EPI_ISL_7070565

EPI_ISL_7070582

EPI_ISL_7070588

EPI_ISL_7070603

EPI_ISL_7070621

EPI_ISL_7070629

EPI_ISL_7070647

EPI_ISL_7070654

EPI_ISL_7070666

EPI_ISL_7070673

EPI_ISL_7070687

EPI_ISL_7070704

EPI_ISL_7070710

EPI_ISL_7070720

EPI_ISL_7070729

EPI_ISL_7070739

EPI_ISL_7070740

EPI_ISL_7070746

EPI_ISL_1865592

EPI_ISL_7070755

EPI_ISL_7070774

EPI_ISL_7070778

EPI_ISL_7070786

EPI_ISL_7070796

EPI_ISL_7070804

EPI_ISL_1865350

EPI_ISL_7070822

EPI_ISL_7070833

EPI_ISL_1865247

EPI_ISL_7070864

EPI_ISL_7070872

EPI_ISL_7070880

EPI_ISL_2094701

EPI_ISL_1864969

EPI_ISL_7070893

EPI_ISL_7070904

EPI_ISL_7070909

EPI_ISL_7070917

EPI_ISL_2094703

EPI_ISL_7070932

EPI_ISL_7070939

EPI_ISL_2271630

EPI_ISL_2094708

EPI_ISL_7070946

EPI_ISL_7070951

EPI_ISL_1865485

EPI_ISL_7070966

EPI_ISL_7070974

EPI_ISL_7070982

EPI_ISL_7070990

EPI_ISL_2094714

EPI_ISL_7071004

EPI_ISL_7071010

EPI_ISL_7071013

EPI_ISL_7071044

EPI_ISL_7071048

EPI_ISL_7071055

EPI_ISL_7071068

EPI_ISL_7071074

EPI_ISL_1865242

EPI_ISL_7071078

EPI_ISL_2271631

EPI_ISL_7071103

EPI_ISL_7071107

EPI_ISL_7071125

EPI_ISL_1864975

EPI_ISL_1865245

EPI_ISL_7071153

EPI_ISL_7071169

EPI_ISL_1865486

EPI_ISL_7071186

EPI_ISL_7071201

EPI_ISL_7071208

EPI_ISL_7071213

EPI_ISL_7071214

EPI_ISL_7071215

EPI_ISL_7071216

EPI_ISL_7071222

EPI_ISL_7071223

EPI_ISL_7071234

EPI_ISL_7071235

EPI_ISL_7071236

EPI_ISL_7071237

EPI_ISL_7071247

EPI_ISL_7071248

EPI_ISL_7071258

EPI_ISL_7071262

EPI_ISL_7071268

EPI_ISL_7071275

EPI_ISL_1865241

EPI_ISL_2271633

EPI_ISL_2094761

EPI_ISL_1865294

EPI_ISL_1865296

EPI_ISL_2271634

EPI_ISL_1864940

EPI_ISL_2271635

EPI_ISL_1865394

EPI_ISL_2271637

EPI_ISL_2094813

EPI_ISL_1865612

EPI_ISL_3909450

EPI_ISL_1679301

EPI_ISL_1865504

EPI_ISL_1865624

EPI_ISL_1679061

EPI_ISL_1715323

EPI_ISL_1681908

EPI_ISL_2271639

EPI_ISL_1715324

EPI_ISL_1715325

EPI_ISL_1865621

EPI_ISL_1865502

EPI_ISL_1865507

EPI_ISL_1865603

EPI_ISL_1681890

EPI_ISL_2271641

EPI_ISL_1865608

EPI_ISL_1681779

EPI_ISL_1865416

EPI_ISL_2094888

EPI_ISL_1865418

EPI_ISL_2271642

EPI_ISL_1865538

EPI_ISL_2094908

EPI_ISL_1864891

EPI_ISL_1681663

EPI_ISL_1865535

EPI_ISL_2676474

EPI_ISL_2676475

EPI_ISL_1681669

EPI_ISL_2676476

EPI_ISL_2271644

EPI_ISL_1681671

EPI_ISL_1681793

EPI_ISL_1865307

EPI_ISL_1681677

EPI_ISL_1681678

EPI_ISL_2094957

EPI_ISL_1681675

EPI_ISL_1681682

EPI_ISL_1681688

EPI_ISL_1865425

EPI_ISL_2271648

EPI_ISL_1864878

EPI_ISL_1681696

EPI_ISL_1681694

EPI_ISL_1681690

EPI_ISL_3187231

EPI_ISL_1865663

EPI_ISL_1681697

EPI_ISL_1865420

EPI_ISL_1865636

EPI_ISL_1865517

EPI_ISL_1865516

EPI_ISL_1865632

EPI_ISL_1865634

EPI_ISL_2095034

EPI_ISL_1681477

EPI_ISL_1865513

EPI_ISL_3187278

EPI_ISL_2271653

EPI_ISL_6174465

EPI_ISL_6174466

EPI_ISL_3187285

EPI_ISL_6174468

EPI_ISL_6174469

EPI_ISL_1865648

EPI_ISL_3187288

EPI_ISL_1865403

EPI_ISL_1865644

EPI_ISL_1894964

EPI_ISL_1858921

EPI_ISL_1864367

EPI_ISL_2271655

EPI_ISL_3187321

EPI_ISL_1864366

EPI_ISL_1864245

EPI_ISL_1864489

EPI_ISL_1864484

EPI_ISL_3187341

EPI_ISL_1715326

EPI_ISL_1715327

EPI_ISL_1715328

EPI_ISL_1864362

EPI_ISL_1715329

EPI_ISL_1714770

EPI_ISL_1714771

EPI_ISL_1714772

EPI_ISL_1714773

EPI_ISL_1714774

EPI_ISL_1714775

EPI_ISL_1714776

EPI_ISL_1714777

EPI_ISL_1714779

EPI_ISL_1714780

EPI_ISL_1715330

EPI_ISL_3187377

EPI_ISL_3187379

EPI_ISL_1714807

EPI_ISL_1714808

EPI_ISL_1714809

EPI_ISL_1714811

EPI_ISL_1714812

EPI_ISL_1714813

EPI_ISL_1714814

EPI_ISL_1714815

EPI_ISL_1714816

EPI_ISL_1714817

EPI_ISL_1714818

EPI_ISL_1714819

EPI_ISL_1714820

EPI_ISL_1714821

EPI_ISL_3187386

EPI_ISL_1714822

EPI_ISL_1714823

EPI_ISL_1714824

EPI_ISL_1714826

EPI_ISL_1714827

EPI_ISL_1714828

EPI_ISL_1714829

EPI_ISL_1714833

EPI_ISL_1714834

EPI_ISL_1714835

EPI_ISL_1714836

EPI_ISL_3187393

EPI_ISL_1714837

EPI_ISL_1714838

EPI_ISL_1714840

EPI_ISL_1864259

EPI_ISL_1714842

EPI_ISL_3187397

EPI_ISL_1714845

EPI_ISL_1715331

EPI_ISL_1714846

EPI_ISL_1714847

EPI_ISL_1714848

EPI_ISL_1714849

EPI_ISL_1714850

EPI_ISL_1714852

EPI_ISL_1714853

EPI_ISL_1714854

EPI_ISL_1714855

EPI_ISL_1714856

EPI_ISL_1714857

EPI_ISL_1714858

EPI_ISL_1714859

EPI_ISL_1714860

EPI_ISL_1714861

EPI_ISL_1714862

EPI_ISL_1714863

EPI_ISL_1714864

EPI_ISL_1714865

EPI_ISL_1714866

EPI_ISL_1714867

EPI_ISL_1714869

EPI_ISL_1714870

EPI_ISL_1714871

EPI_ISL_1714872

EPI_ISL_1714873

EPI_ISL_1714875

EPI_ISL_1714876

EPI_ISL_1714877

EPI_ISL_1714878

EPI_ISL_1714879

EPI_ISL_3187417

EPI_ISL_1714880

EPI_ISL_1714881

EPI_ISL_1714882

EPI_ISL_1864253

EPI_ISL_1864495

EPI_ISL_1714883

EPI_ISL_1714884

EPI_ISL_1714885

EPI_ISL_1714886

EPI_ISL_1714887

EPI_ISL_1714888

EPI_ISL_1714889

EPI_ISL_1714890

EPI_ISL_2825773

EPI_ISL_1714891

EPI_ISL_1714892

EPI_ISL_1714893

EPI_ISL_3187424

EPI_ISL_1714895

EPI_ISL_1714896

EPI_ISL_3187426

EPI_ISL_1714897

EPI_ISL_1714899

EPI_ISL_1714900

EPI_ISL_1714901

EPI_ISL_1714903

EPI_ISL_1714904

EPI_ISL_1714905

EPI_ISL_1714906

EPI_ISL_1714908

EPI_ISL_1681612

EPI_ISL_1864370

EPI_ISL_1714909

EPI_ISL_1714910

EPI_ISL_1714911

EPI_ISL_1714912

EPI_ISL_1714913

EPI_ISL_1714914

EPI_ISL_1714915

EPI_ISL_1714916

EPI_ISL_1714917

EPI_ISL_1714918

EPI_ISL_2271662

EPI_ISL_1714920

EPI_ISL_1714922

EPI_ISL_1714923

EPI_ISL_1714924

EPI_ISL_1714925

EPI_ISL_1714926

EPI_ISL_1714927

EPI_ISL_3187439

EPI_ISL_1681610

EPI_ISL_1714928

EPI_ISL_1681618

EPI_ISL_1714931

EPI_ISL_1714932

EPI_ISL_1714933

EPI_ISL_1714934

EPI_ISL_1714935

EPI_ISL_1714936

EPI_ISL_1714938

EPI_ISL_1714939

EPI_ISL_1714940

EPI_ISL_1714941

EPI_ISL_1714942

EPI_ISL_1714943

EPI_ISL_2271664

EPI_ISL_3364188

EPI_ISL_1714945

EPI_ISL_1714946

EPI_ISL_1714947

EPI_ISL_1714948

EPI_ISL_1714949

EPI_ISL_1858821

EPI_ISL_1714950

EPI_ISL_1714951

EPI_ISL_1714952

EPI_ISL_1714953

EPI_ISL_3187459

EPI_ISL_2271665

EPI_ISL_1714954

EPI_ISL_1714955

EPI_ISL_1714956

EPI_ISL_1714957

EPI_ISL_1714958

EPI_ISL_1714959

EPI_ISL_1714960

EPI_ISL_1714961

EPI_ISL_1864588

EPI_ISL_1714963

EPI_ISL_1681623

EPI_ISL_1864346

EPI_ISL_1714964

EPI_ISL_1714966

EPI_ISL_1714967

EPI_ISL_1714968

EPI_ISL_1714969

EPI_ISL_1714970

EPI_ISL_1714971

EPI_ISL_1681620

EPI_ISL_1714972

EPI_ISL_2095257

EPI_ISL_1714973

EPI_ISL_1714975

EPI_ISL_1714976

EPI_ISL_1714977

EPI_ISL_1714978

EPI_ISL_1714980

EPI_ISL_1714981

EPI_ISL_1714982

EPI_ISL_1714983

EPI_ISL_1714984

EPI_ISL_1714985

EPI_ISL_1714986

EPI_ISL_1714987

EPI_ISL_1864220

EPI_ISL_1714988

EPI_ISL_1714989

EPI_ISL_1714990

EPI_ISL_1714991

EPI_ISL_1714992

EPI_ISL_1714993

EPI_ISL_1714995

EPI_ISL_1714996

EPI_ISL_1714997

EPI_ISL_1714998

EPI_ISL_1714999

EPI_ISL_1864585

EPI_ISL_1715000

EPI_ISL_1715001

EPI_ISL_1715002

EPI_ISL_1715003

EPI_ISL_1715004

EPI_ISL_1715005

EPI_ISL_1715006

EPI_ISL_1715007

EPI_ISL_3187494

EPI_ISL_1715008

EPI_ISL_1715009

EPI_ISL_1715010

EPI_ISL_1715011

EPI_ISL_1715012

EPI_ISL_1715013

EPI_ISL_1715014

EPI_ISL_1715015

EPI_ISL_1715016

EPI_ISL_3187502

EPI_ISL_3187503

EPI_ISL_1715017

EPI_ISL_1715018

EPI_ISL_1715019

EPI_ISL_1715020

EPI_ISL_1715021

EPI_ISL_1715022

EPI_ISL_1715023

EPI_ISL_1715024

EPI_ISL_1864581

EPI_ISL_1715026

EPI_ISL_1715027

EPI_ISL_1715028

EPI_ISL_1715029

EPI_ISL_1715030

EPI_ISL_3187509

EPI_ISL_1715031

EPI_ISL_1681633

EPI_ISL_1715032

EPI_ISL_1715033

EPI_ISL_1715034

EPI_ISL_1715035

EPI_ISL_1715038

EPI_ISL_1715039

EPI_ISL_1858710

EPI_ISL_1864460

EPI_ISL_1681873

EPI_ISL_1715040

EPI_ISL_1715041

EPI_ISL_1715042

EPI_ISL_1715043

EPI_ISL_1715045

EPI_ISL_3187512

EPI_ISL_1715046

EPI_ISL_1715047

EPI_ISL_1715050

EPI_ISL_1715051

EPI_ISL_1715052

EPI_ISL_1715053

EPI_ISL_1715054

EPI_ISL_1715055

EPI_ISL_1715058

EPI_ISL_3187520

EPI_ISL_2095305

EPI_ISL_2676841

EPI_ISL_3187533

EPI_ISL_1681880

EPI_ISL_1715332

EPI_ISL_2676842

EPI_ISL_2676843

EPI_ISL_2676844

EPI_ISL_2676846

EPI_ISL_2676847

EPI_ISL_2676848

EPI_ISL_3187540

EPI_ISL_3364357

EPI_ISL_2676850

EPI_ISL_2676852

EPI_ISL_1715120

EPI_ISL_1681525

EPI_ISL_1681886

EPI_ISL_2676853

EPI_ISL_1864478

EPI_ISL_2676854

EPI_ISL_2676855

EPI_ISL_2676856

EPI_ISL_2676857

EPI_ISL_1864231

EPI_ISL_1715333

EPI_ISL_2825891

EPI_ISL_1715204

EPI_ISL_1715205

EPI_ISL_1715206

EPI_ISL_1715207

EPI_ISL_1715208

EPI_ISL_1715209

EPI_ISL_1715210

EPI_ISL_1715212

EPI_ISL_1715334

EPI_ISL_1715214

EPI_ISL_1715215

EPI_ISL_1715216

EPI_ISL_1715217

EPI_ISL_2825892

EPI_ISL_1715335

EPI_ISL_1858887

EPI_ISL_1681906

EPI_ISL_1715219

EPI_ISL_1715220

EPI_ISL_3187575

EPI_ISL_3187578

EPI_ISL_1715221

EPI_ISL_1715222

EPI_ISL_1715223

EPI_ISL_1715224

EPI_ISL_1715225

EPI_ISL_1715226

EPI_ISL_1715227

EPI_ISL_1715229

EPI_ISL_1715230

EPI_ISL_1715231

EPI_ISL_1715232

EPI_ISL_1715233

EPI_ISL_1715234

EPI_ISL_1715235

EPI_ISL_1715236

EPI_ISL_1715237

EPI_ISL_1715238

EPI_ISL_1715239

EPI_ISL_1715241

EPI_ISL_1715242

EPI_ISL_1715243

EPI_ISL_1715244

EPI_ISL_1864474

EPI_ISL_1864232

EPI_ISL_1715245

EPI_ISL_1715246

EPI_ISL_1715247

EPI_ISL_1715336

EPI_ISL_1715248

EPI_ISL_1715249

EPI_ISL_1715250

EPI_ISL_1715251

EPI_ISL_1715252

EPI_ISL_1715253

EPI_ISL_1715254

EPI_ISL_1858647

EPI_ISL_1715256

EPI_ISL_1715257

EPI_ISL_1715258

EPI_ISL_1715260

EPI_ISL_1715261

EPI_ISL_1715262

EPI_ISL_1715263

EPI_ISL_1715264

EPI_ISL_1715265

EPI_ISL_1715266

EPI_ISL_1715267

EPI_ISL_1715268

EPI_ISL_1715269

EPI_ISL_1679165

EPI_ISL_1715271

EPI_ISL_1715272

EPI_ISL_1715273

EPI_ISL_1715274

EPI_ISL_1715275

EPI_ISL_1679285

EPI_ISL_1715276

EPI_ISL_1715277

EPI_ISL_1715278

EPI_ISL_3187598

EPI_ISL_1715279

EPI_ISL_1715280

EPI_ISL_1715281

EPI_ISL_1715283

EPI_ISL_1715285

EPI_ISL_1679284

EPI_ISL_1715286

EPI_ISL_1715287

EPI_ISL_3187602

EPI_ISL_1715288

EPI_ISL_1715289

EPI_ISL_3187605

EPI_ISL_1715290

EPI_ISL_1715291

EPI_ISL_1715292

EPI_ISL_1715293

EPI_ISL_1679162

EPI_ISL_1715295

EPI_ISL_1715297

EPI_ISL_1858770

EPI_ISL_1715299

EPI_ISL_1715301

EPI_ISL_1715302

EPI_ISL_1715303

EPI_ISL_1715304

EPI_ISL_1715305

EPI_ISL_1715306

EPI_ISL_1715307

EPI_ISL_3187608

EPI_ISL_1715308

EPI_ISL_1715310

EPI_ISL_1715311

EPI_ISL_1715312

EPI_ISL_1715313

EPI_ISL_1715314

EPI_ISL_1858895

EPI_ISL_1864285

EPI_ISL_1715316

EPI_ISL_1715317

EPI_ISL_1715318

EPI_ISL_1715319

EPI_ISL_1715321

EPI_ISL_1715322

EPI_ISL_1715337

EPI_ISL_1715338

EPI_ISL_1715339

EPI_ISL_1715340

EPI_ISL_1715341

EPI_ISL_1715342

EPI_ISL_1679166

EPI_ISL_1715343

EPI_ISL_1715344

EPI_ISL_1715346

EPI_ISL_1715347

EPI_ISL_1679159

EPI_ISL_1715348

EPI_ISL_1715349

EPI_ISL_1715350

EPI_ISL_1715351

EPI_ISL_1858412

EPI_ISL_1679161

EPI_ISL_1715352

EPI_ISL_1715353

EPI_ISL_1864166

EPI_ISL_3187625

EPI_ISL_1715354

EPI_ISL_2677752

EPI_ISL_1679280

EPI_ISL_1715355

EPI_ISL_1715356

EPI_ISL_1715357

EPI_ISL_1715358

EPI_ISL_1715359

EPI_ISL_1715360

EPI_ISL_1715361

EPI_ISL_1715362

EPI_ISL_1715363

EPI_ISL_1679033

EPI_ISL_1679154

EPI_ISL_2677754

EPI_ISL_1864284

EPI_ISL_1679032

EPI_ISL_1679273

EPI_ISL_1679152

EPI_ISL_1864283

EPI_ISL_1679272

EPI_ISL_1679030

EPI_ISL_1679151

EPI_ISL_1864179

EPI_ISL_1864291

EPI_ISL_2825964

EPI_ISL_1679158

EPI_ISL_1858620

EPI_ISL_1864290

EPI_ISL_1679278

EPI_ISL_1858863

EPI_ISL_1864176

EPI_ISL_1715440

EPI_ISL_1715441

EPI_ISL_1715442

EPI_ISL_1715443

EPI_ISL_1858865

EPI_ISL_1715446

EPI_ISL_1679155

EPI_ISL_1715448

EPI_ISL_1715450

EPI_ISL_1715452

EPI_ISL_1715453

EPI_ISL_1715454

EPI_ISL_3187656

EPI_ISL_1715458

EPI_ISL_3187657

EPI_ISL_1715459

EPI_ISL_1715461

EPI_ISL_1715462

EPI_ISL_1715463

EPI_ISL_1715464

EPI_ISL_1715468

EPI_ISL_1715469

EPI_ISL_1715471

EPI_ISL_1715472

EPI_ISL_3187658

EPI_ISL_1717514

EPI_ISL_1715474

EPI_ISL_1715476

EPI_ISL_1715477

EPI_ISL_1715479

EPI_ISL_1715480

EPI_ISL_3187665

EPI_ISL_1715483

EPI_ISL_1679143

EPI_ISL_1715487

EPI_ISL_1715489

EPI_ISL_1864171

EPI_ISL_1715491

EPI_ISL_1715492

EPI_ISL_3187688

EPI_ISL_1715495

EPI_ISL_1715497

EPI_ISL_1715499

EPI_ISL_1715502

EPI_ISL_1715504

EPI_ISL_1715507

EPI_ISL_1715509

EPI_ISL_1715510

EPI_ISL_1715511

EPI_ISL_1715517

EPI_ISL_1715518

EPI_ISL_1715521

EPI_ISL_1679268

EPI_ISL_1715524

EPI_ISL_1715525

EPI_ISL_1715527

EPI_ISL_1715528

EPI_ISL_1679146

EPI_ISL_1679266

EPI_ISL_1679267

EPI_ISL_1715532

EPI_ISL_1715533

EPI_ISL_1715535

EPI_ISL_1715537

EPI_ISL_1715539

EPI_ISL_1715541

EPI_ISL_1715542

EPI_ISL_1715545

EPI_ISL_1715547

EPI_ISL_1864146

EPI_ISL_1864268

EPI_ISL_1715549

EPI_ISL_1679024

EPI_ISL_1679145

EPI_ISL_1679265

EPI_ISL_1715554

EPI_ISL_2826007

EPI_ISL_1715561

EPI_ISL_1715566

EPI_ISL_1715567

EPI_ISL_1715568

EPI_ISL_1715569

EPI_ISL_1715573

EPI_ISL_1715574

EPI_ISL_1864149

EPI_ISL_1715576

EPI_ISL_1715582

EPI_ISL_1715585

EPI_ISL_1679138

EPI_ISL_1679259

EPI_ISL_1715589

EPI_ISL_1715590

EPI_ISL_3187694

EPI_ISL_1715598

EPI_ISL_1864385

EPI_ISL_1715599

EPI_ISL_1679137

EPI_ISL_1679374

EPI_ISL_1715606

EPI_ISL_1715607

EPI_ISL_1715611

EPI_ISL_1715614

EPI_ISL_1715616

EPI_ISL_1715620

EPI_ISL_2826018

EPI_ISL_2826021

EPI_ISL_1679373

EPI_ISL_1858759

EPI_ISL_1715642

EPI_ISL_1715643

EPI_ISL_1715645

EPI_ISL_1679010

EPI_ISL_1715646

EPI_ISL_3187708

EPI_ISL_1715655

EPI_ISL_1864266

EPI_ISL_1715662

EPI_ISL_1715664

EPI_ISL_1715670

EPI_ISL_1715674

EPI_ISL_1715677

EPI_ISL_1715678

EPI_ISL_1679250

EPI_ISL_1679256

EPI_ISL_1715683

EPI_ISL_1715690

EPI_ISL_1864144

EPI_ISL_3187722

EPI_ISL_1679255

EPI_ISL_1715693

EPI_ISL_1715697

EPI_ISL_1715699

EPI_ISL_1715703

EPI_ISL_1864260

EPI_ISL_1679129

EPI_ISL_1679370

EPI_ISL_1715727

EPI_ISL_1715729

EPI_ISL_1715730

EPI_ISL_1715734

EPI_ISL_1715737

EPI_ISL_1715743

EPI_ISL_1715744

EPI_ISL_1679128

EPI_ISL_1858687

EPI_ISL_1715748

EPI_ISL_1715750

EPI_ISL_1715755

EPI_ISL_1715758

EPI_ISL_1715759

EPI_ISL_1679126

EPI_ISL_1679127

EPI_ISL_1679248

EPI_ISL_1715766

EPI_ISL_1679242

EPI_ISL_1679360

EPI_ISL_1715828

EPI_ISL_1715830

EPI_ISL_1864157

EPI_ISL_1679367

EPI_ISL_1715833

EPI_ISL_1715835

EPI_ISL_1715842

EPI_ISL_1715848

EPI_ISL_1715849

EPI_ISL_1715853

EPI_ISL_1715854

EPI_ISL_1715855

EPI_ISL_1715857

EPI_ISL_1715858

EPI_ISL_1679245

EPI_ISL_1715859

EPI_ISL_1864153

EPI_ISL_1679123

EPI_ISL_1679244

EPI_ISL_1679237

EPI_ISL_1679358

EPI_ISL_3187775

EPI_ISL_1679119

EPI_ISL_1679231

EPI_ISL_1679230

EPI_ISL_1679348

EPI_ISL_1679235

EPI_ISL_1679355

EPI_ISL_1858692

EPI_ISL_1679233

EPI_ISL_1864180

EPI_ISL_1858339

EPI_ISL_2677767

EPI_ISL_1679226

EPI_ISL_1679346

EPI_ISL_1679347

EPI_ISL_1679225

EPI_ISL_1679229

EPI_ISL_1679341

EPI_ISL_1864199

EPI_ISL_1679224

EPI_ISL_1679345

EPI_ISL_1679344

EPI_ISL_1858787

EPI_ISL_1858425

EPI_ISL_1679216

EPI_ISL_1679330

EPI_ISL_1679339

EPI_ISL_1864611

EPI_ISL_1679333

EPI_ISL_1864616

EPI_ISL_1864618

EPI_ISL_1678912

EPI_ISL_1858678

EPI_ISL_1679090

EPI_ISL_1678903

EPI_ISL_1679099

EPI_ISL_1864647

EPI_ISL_1858242

EPI_ISL_1864641

EPI_ISL_1858364

EPI_ISL_1864402

EPI_ISL_1679083

EPI_ISL_1679197

EPI_ISL_1864640

EPI_ISL_1679075

EPI_ISL_1716560

EPI_ISL_1716562

EPI_ISL_1679190

EPI_ISL_1858249

EPI_ISL_1679193

EPI_ISL_1679192

EPI_ISL_1864658

EPI_ISL_1679191

EPI_ISL_1864531

EPI_ISL_1858362

EPI_ISL_1864410

EPI_ISL_1679183

EPI_ISL_1679295

EPI_ISL_1679297

EPI_ISL_1864412

EPI_ISL_1679296

EPI_ISL_1858133

EPI_ISL_1679174

EPI_ISL_1679294

EPI_ISL_1679173

EPI_ISL_1864650

EPI_ISL_1679178

EPI_ISL_1679293

EPI_ISL_2677770

EPI_ISL_2677774

EPI_ISL_2095946

EPI_ISL_1679171

EPI_ISL_1679292

EPI_ISL_1864505

EPI_ISL_1679170

EPI_ISL_1679290

EPI_ISL_1678996

EPI_ISL_1678875

EPI_ISL_1864627

EPI_ISL_1678994

EPI_ISL_2677776

EPI_ISL_2677778

EPI_ISL_1858251

EPI_ISL_1864508

EPI_ISL_1864629

EPI_ISL_2677785

EPI_ISL_2677790

EPI_ISL_2677791

EPI_ISL_2677793

EPI_ISL_1864635

EPI_ISL_2677796

EPI_ISL_1864513

EPI_ISL_1678988

EPI_ISL_1716862

EPI_ISL_1716863

EPI_ISL_2677799

EPI_ISL_1716864

EPI_ISL_1716866

EPI_ISL_1716867

EPI_ISL_2677800

EPI_ISL_1864515

EPI_ISL_1716871

EPI_ISL_1716883

EPI_ISL_1716884

EPI_ISL_1716885

EPI_ISL_2677802

EPI_ISL_1716887

EPI_ISL_1716888

EPI_ISL_1716889

EPI_ISL_2677804

EPI_ISL_2677805

EPI_ISL_1716896

EPI_ISL_1716897

EPI_ISL_1858465

EPI_ISL_1864510

EPI_ISL_1716906

EPI_ISL_1716907

EPI_ISL_1716908

EPI_ISL_1716909

EPI_ISL_1716912

EPI_ISL_1716913

EPI_ISL_1716914

EPI_ISL_1716915

EPI_ISL_1716916

EPI_ISL_1716917

EPI_ISL_2677810

EPI_ISL_2677811

EPI_ISL_1716919

EPI_ISL_1716920

EPI_ISL_1716921

EPI_ISL_1716923

EPI_ISL_1864511

EPI_ISL_1716926

EPI_ISL_1716927

EPI_ISL_1716928

EPI_ISL_1716929

EPI_ISL_1716930

EPI_ISL_1716931

EPI_ISL_1716933

EPI_ISL_1716934

EPI_ISL_1716936

EPI_ISL_1716937

EPI_ISL_1716938

EPI_ISL_2677812

EPI_ISL_2677813

EPI_ISL_1864632

EPI_ISL_1716941

EPI_ISL_2677814

EPI_ISL_1716942

EPI_ISL_1716943

EPI_ISL_1716944

EPI_ISL_1716945

EPI_ISL_1716946

EPI_ISL_1716947

EPI_ISL_1716948

EPI_ISL_1716949

EPI_ISL_1858589

EPI_ISL_2677816

EPI_ISL_1716951

EPI_ISL_1716952

EPI_ISL_1716953

EPI_ISL_2677821

EPI_ISL_1716954

EPI_ISL_1716955

EPI_ISL_1716956

EPI_ISL_1716958

EPI_ISL_1716959

EPI_ISL_2677825

EPI_ISL_1716962

EPI_ISL_1716963

EPI_ISL_2677829

EPI_ISL_1716964

EPI_ISL_1716965

EPI_ISL_1716966

EPI_ISL_1716968

EPI_ISL_1716969

EPI_ISL_1716970

EPI_ISL_1678948

EPI_ISL_1678949

EPI_ISL_1716973

EPI_ISL_1716975

EPI_ISL_1716976

EPI_ISL_1716977

EPI_ISL_1716979

EPI_ISL_1716983

EPI_ISL_1716984

EPI_ISL_1716985

EPI_ISL_2677833

EPI_ISL_1716988

EPI_ISL_2677838

EPI_ISL_2677839

EPI_ISL_1716991

EPI_ISL_1716992

EPI_ISL_1716993

EPI_ISL_1858595

EPI_ISL_2677842

EPI_ISL_1678930

EPI_ISL_2677843

EPI_ISL_2677844

EPI_ISL_2677845

EPI_ISL_2677846

EPI_ISL_2677847

EPI_ISL_2677851

EPI_ISL_2677854

EPI_ISL_2677857

EPI_ISL_2677860

EPI_ISL_1678920

EPI_ISL_1679324

EPI_ISL_1679329

EPI_ISL_1717108

EPI_ISL_2677864

EPI_ISL_1717118

EPI_ISL_2095969

EPI_ISL_2677870

EPI_ISL_1717128

EPI_ISL_1717144

EPI_ISL_1679319

EPI_ISL_2677871

EPI_ISL_2677872

EPI_ISL_1717161

EPI_ISL_2677873

EPI_ISL_1717170

EPI_ISL_1717173

EPI_ISL_1717175

EPI_ISL_1864561

EPI_ISL_1717179

EPI_ISL_1717186

EPI_ISL_1717187

EPI_ISL_1717189

EPI_ISL_1717193

EPI_ISL_1717195

EPI_ISL_1679304

EPI_ISL_2095846

EPI_ISL_1717198

EPI_ISL_1717528

EPI_ISL_1717200

EPI_ISL_2677877

EPI_ISL_1679300

EPI_ISL_2677878

EPI_ISL_3526042

EPI_ISL_2677879

EPI_ISL_2677880

EPI_ISL_3526047

EPI_ISL_3526049

EPI_ISL_1717228

EPI_ISL_1717229

EPI_ISL_1678895

EPI_ISL_3526053

EPI_ISL_1717234

EPI_ISL_1717236

EPI_ISL_1717237

EPI_ISL_1717533

EPI_ISL_1717241

EPI_ISL_1717242

EPI_ISL_1864579

EPI_ISL_1717254

EPI_ISL_1717257

EPI_ISL_1717258

EPI_ISL_1717260

EPI_ISL_1717267

EPI_ISL_1717273

EPI_ISL_2677885

EPI_ISL_2677886

EPI_ISL_1717288

EPI_ISL_1864216

EPI_ISL_1864219

EPI_ISL_1717291

EPI_ISL_1717294

EPI_ISL_2677888

EPI_ISL_1717301

EPI_ISL_2677891

EPI_ISL_1717305

EPI_ISL_2095882

EPI_ISL_1717310

EPI_ISL_1717312

EPI_ISL_1717535

EPI_ISL_1717328

EPI_ISL_1717333

EPI_ISL_1717338

EPI_ISL_1864454

EPI_ISL_1717357

EPI_ISL_1717358

EPI_ISL_1717360

EPI_ISL_1717371

EPI_ISL_1717374

EPI_ISL_1717375

EPI_ISL_1717376

EPI_ISL_1717380

EPI_ISL_1717384

EPI_ISL_1717389

EPI_ISL_1694733

EPI_ISL_1717404

EPI_ISL_1717405

EPI_ISL_1858148

EPI_ISL_1717413

EPI_ISL_1717414

EPI_ISL_1717417

EPI_ISL_1717424

EPI_ISL_1717428

EPI_ISL_1717429

EPI_ISL_1717431

EPI_ISL_1864693

EPI_ISL_1717440

EPI_ISL_1717442

EPI_ISL_1858262

EPI_ISL_1717464

EPI_ISL_1717468

EPI_ISL_1717470

EPI_ISL_1717473

EPI_ISL_1717474

EPI_ISL_2095933

EPI_ISL_1864332

EPI_ISL_1717481

EPI_ISL_1717483

EPI_ISL_1717485

EPI_ISL_1717489

EPI_ISL_4376007

EPI_ISL_1717498

EPI_ISL_1717505

EPI_ISL_2095939

EPI_ISL_1717509

EPI_ISL_1717511

EPI_ISL_1717930

EPI_ISL_2677898

EPI_ISL_1864668

EPI_ISL_2095950

EPI_ISL_1717563

EPI_ISL_1717568

EPI_ISL_2677909

EPI_ISL_1717574

EPI_ISL_2677911

EPI_ISL_1717581

EPI_ISL_1717582

EPI_ISL_1717584

EPI_ISL_2677912

EPI_ISL_2677915

EPI_ISL_2095968

EPI_ISL_1717588

EPI_ISL_1717591

EPI_ISL_1864548

EPI_ISL_1717603

EPI_ISL_1717605

EPI_ISL_1864306

EPI_ISL_1717617

EPI_ISL_1717618

EPI_ISL_1717622

EPI_ISL_1717627

EPI_ISL_1858187

EPI_ISL_1864663

EPI_ISL_1717638

EPI_ISL_1717643

EPI_ISL_1717658

EPI_ISL_1864545

EPI_ISL_1864666

EPI_ISL_1717659

EPI_ISL_1717671

EPI_ISL_1717675

EPI_ISL_1717677

EPI_ISL_1717684

EPI_ISL_1503233

EPI_ISL_1717691

EPI_ISL_1503236

EPI_ISL_1717699

EPI_ISL_1717700

EPI_ISL_1717701

EPI_ISL_2543563

EPI_ISL_1717724

EPI_ISL_1717750

EPI_ISL_1717753

EPI_ISL_1694837

EPI_ISL_1694838

EPI_ISL_1717762

EPI_ISL_1717769

EPI_ISL_1717771

EPI_ISL_1864558

EPI_ISL_1717773

EPI_ISL_1694845

EPI_ISL_1717789

EPI_ISL_1717793

EPI_ISL_3781783

EPI_ISL_2677998

EPI_ISL_2159503

EPI_ISL_2164289

EPI_ISL_2339722

EPI_ISL_2159528

EPI_ISL_2339728

EPI_ISL_1717806

EPI_ISL_2678011

EPI_ISL_2339751

EPI_ISL_1717811

EPI_ISL_1717814

EPI_ISL_1717819

EPI_ISL_2678028

EPI_ISL_1717831

EPI_ISL_2159912

EPI_ISL_1717834

EPI_ISL_1717840

EPI_ISL_1717844

EPI_ISL_2159814

EPI_ISL_1717847

EPI_ISL_1717862

EPI_ISL_1717867

EPI_ISL_2166354

EPI_ISL_2162808

EPI_ISL_1717879

EPI_ISL_1717897

EPI_ISL_1717898

EPI_ISL_1717900

EPI_ISL_1717906

EPI_ISL_1717907

EPI_ISL_2544198

EPI_ISL_2544200

EPI_ISL_1717908

EPI_ISL_1717910

EPI_ISL_1717923

EPI_ISL_1717924

EPI_ISL_1717942

EPI_ISL_1717962

EPI_ISL_1717963

EPI_ISL_1717968

EPI_ISL_2159260

EPI_ISL_1717988

EPI_ISL_3784317

EPI_ISL_1718003

EPI_ISL_1718008

EPI_ISL_3784687

EPI_ISL_3784566

EPI_ISL_3784324

EPI_ISL_1718012

EPI_ISL_1718015

EPI_ISL_1718023

EPI_ISL_3784229

EPI_ISL_1718027

EPI_ISL_1930794

EPI_ISL_3783781

EPI_ISL_1718067

EPI_ISL_1718074

EPI_ISL_1718087

EPI_ISL_1718101

EPI_ISL_1579233

EPI_ISL_1718117

EPI_ISL_2160578

EPI_ISL_3784391

EPI_ISL_3784039

EPI_ISL_3785008

EPI_ISL_3785002

EPI_ISL_1718134

EPI_ISL_3532454

EPI_ISL_3532466

EPI_ISL_3532467

EPI_ISL_1718137

EPI_ISL_1718138

EPI_ISL_5696514

EPI_ISL_1718147

EPI_ISL_1718339

EPI_ISL_3532973

EPI_ISL_3784134

EPI_ISL_1718181

EPI_ISL_3783901

EPI_ISL_1718205

EPI_ISL_1718212

EPI_ISL_1718215

EPI_ISL_1718216

EPI_ISL_1718225

EPI_ISL_1718235

EPI_ISL_3785195

EPI_ISL_1718323

EPI_ISL_2544707

EPI_ISL_1718324

EPI_ISL_1718325

EPI_ISL_1718326

EPI_ISL_1718327

EPI_ISL_2170859

EPI_ISL_1718330

EPI_ISL_1718331

EPI_ISL_1718332

EPI_ISL_1718333

EPI_ISL_1718334

EPI_ISL_1718335

EPI_ISL_1718336

EPI_ISL_1718337

EPI_ISL_1718338

EPI_ISL_2544759

EPI_ISL_1718341

EPI_ISL_1718342

EPI_ISL_1718343

EPI_ISL_1718345

EPI_ISL_3783777

EPI_ISL_1718347

EPI_ISL_1718348

EPI_ISL_1718349

EPI_ISL_1718350

EPI_ISL_1718352

EPI_ISL_1718353

EPI_ISL_1718355

EPI_ISL_1718356

EPI_ISL_1718357

EPI_ISL_1718360

EPI_ISL_1718361

EPI_ISL_1718363

EPI_ISL_1718365

EPI_ISL_1718367

EPI_ISL_1718368

EPI_ISL_1718369

EPI_ISL_1718370

EPI_ISL_1718371

EPI_ISL_1718372

EPI_ISL_1718373

EPI_ISL_1718374

EPI_ISL_1718377

EPI_ISL_1718378

EPI_ISL_1718379

EPI_ISL_1718380

EPI_ISL_1718382

EPI_ISL_1718385

EPI_ISL_1718387

EPI_ISL_1718523

EPI_ISL_1718389

EPI_ISL_1718390

EPI_ISL_1718391

EPI_ISL_1718392

EPI_ISL_1718393

EPI_ISL_1756701

EPI_ISL_1718395

EPI_ISL_1718396

EPI_ISL_1718397

EPI_ISL_1718399

EPI_ISL_1718401

EPI_ISL_1718402

EPI_ISL_1718403

EPI_ISL_1718404

EPI_ISL_1718405

EPI_ISL_1718406

EPI_ISL_1718407

EPI_ISL_1718409

EPI_ISL_1718410

EPI_ISL_1718411

EPI_ISL_1718412

EPI_ISL_1718414

EPI_ISL_1718416

EPI_ISL_1718419

EPI_ISL_1718420

EPI_ISL_1718422

EPI_ISL_1718424

EPI_ISL_1718425

EPI_ISL_1718426

EPI_ISL_1718427

EPI_ISL_1718428

EPI_ISL_1718429

EPI_ISL_1718430

EPI_ISL_1718431

EPI_ISL_1718432

EPI_ISL_1718439

EPI_ISL_1718440

EPI_ISL_1718442

EPI_ISL_1718443

EPI_ISL_1718444

EPI_ISL_1718445

EPI_ISL_2341396

EPI_ISL_1718448

EPI_ISL_1718449

EPI_ISL_1718450

EPI_ISL_1718451

EPI_ISL_2160213

EPI_ISL_1718453

EPI_ISL_1718454

EPI_ISL_1718455

EPI_ISL_1718459

EPI_ISL_1718461

EPI_ISL_1718463

EPI_ISL_1718464

EPI_ISL_1718465

EPI_ISL_1718466

EPI_ISL_1718467

EPI_ISL_1718468

EPI_ISL_1718470

EPI_ISL_1718472

EPI_ISL_1718473

EPI_ISL_1718474

EPI_ISL_1718475

EPI_ISL_1718479

EPI_ISL_2762922

EPI_ISL_1718481

EPI_ISL_1718483

EPI_ISL_1718484

EPI_ISL_1718487

EPI_ISL_1718488

EPI_ISL_1756884

EPI_ISL_1718491

EPI_ISL_1718492

EPI_ISL_2341531

EPI_ISL_1718498

EPI_ISL_1718499

EPI_ISL_1718500

EPI_ISL_1718502

EPI_ISL_1718503

EPI_ISL_1718506

EPI_ISL_1718508

EPI_ISL_1718510

EPI_ISL_2160145

EPI_ISL_1718512

EPI_ISL_1718513

EPI_ISL_1718514

EPI_ISL_1718515

EPI_ISL_1718522

EPI_ISL_2763619

EPI_ISL_1718517

EPI_ISL_1718518

EPI_ISL_1718519

EPI_ISL_3536575

EPI_ISL_3536577

EPI_ISL_3536578

EPI_ISL_3536585

EPI_ISL_3536597

EPI_ISL_1718524

EPI_ISL_1718525

EPI_ISL_1718526

EPI_ISL_3536738

EPI_ISL_3536740

EPI_ISL_3536741

EPI_ISL_3536742

EPI_ISL_3536745

EPI_ISL_3536746

EPI_ISL_3536747

EPI_ISL_3536748

EPI_ISL_3536749

EPI_ISL_3536750

EPI_ISL_3536757

EPI_ISL_1718529

EPI_ISL_1718530

EPI_ISL_3536775

EPI_ISL_3536777

EPI_ISL_3536780

EPI_ISL_3536784

EPI_ISL_3536785

EPI_ISL_3536786

EPI_ISL_3536787

EPI_ISL_3536788

EPI_ISL_3536789

EPI_ISL_3536790

EPI_ISL_3536791

EPI_ISL_3536793

EPI_ISL_3536794

EPI_ISL_3536795

EPI_ISL_3536796

EPI_ISL_3536797

EPI_ISL_3536798

EPI_ISL_3536799

EPI_ISL_3536800

EPI_ISL_3536801

EPI_ISL_3536802

EPI_ISL_3536803

EPI_ISL_3536804

EPI_ISL_3536805

EPI_ISL_3536806

EPI_ISL_3536807

EPI_ISL_3536809

EPI_ISL_3536811

EPI_ISL_1718536

EPI_ISL_1718538

EPI_ISL_3536853

EPI_ISL_3536884

EPI_ISL_1718540

EPI_ISL_1718541

EPI_ISL_1718546

EPI_ISL_1718548

EPI_ISL_1718549

EPI_ISL_1718551

EPI_ISL_1718552

EPI_ISL_1718553

EPI_ISL_2160155

EPI_ISL_2160034

EPI_ISL_1718556

EPI_ISL_1718557

EPI_ISL_1718560

EPI_ISL_1718561

EPI_ISL_1718562

EPI_ISL_1718563

EPI_ISL_1718564

EPI_ISL_1718565

EPI_ISL_1718568

EPI_ISL_1718569

EPI_ISL_1718570

EPI_ISL_1718573

EPI_ISL_1718574

EPI_ISL_1718575

EPI_ISL_1718577

EPI_ISL_1718580

EPI_ISL_1718581

EPI_ISL_2545379

EPI_ISL_1718582

EPI_ISL_1718584

EPI_ISL_1718586

EPI_ISL_1718587

EPI_ISL_1718589

EPI_ISL_1718590

EPI_ISL_1718591

EPI_ISL_1718592

EPI_ISL_1718593

EPI_ISL_1718594

EPI_ISL_1718595

EPI_ISL_1718596

EPI_ISL_1718599

EPI_ISL_1718601

EPI_ISL_1718602

EPI_ISL_1718603

EPI_ISL_1718604

EPI_ISL_1718605

EPI_ISL_1718606

EPI_ISL_1718607

EPI_ISL_1718608

EPI_ISL_1718609

EPI_ISL_1718611

EPI_ISL_1718612

EPI_ISL_1718613

EPI_ISL_1718614

EPI_ISL_1718615

EPI_ISL_1718616

EPI_ISL_1718617

EPI_ISL_1718618

EPI_ISL_1928222

EPI_ISL_1718619

EPI_ISL_1718620

EPI_ISL_2545235

EPI_ISL_2545237

EPI_ISL_2545239

EPI_ISL_2545244

EPI_ISL_1718631

EPI_ISL_1718632

EPI_ISL_1718633

EPI_ISL_2545245

EPI_ISL_1718634

EPI_ISL_2545249

EPI_ISL_2678835

EPI_ISL_2545253

EPI_ISL_2545256

EPI_ISL_2678837

EPI_ISL_2678841

EPI_ISL_2678845

EPI_ISL_1580644

EPI_ISL_2545268

EPI_ISL_2545269

EPI_ISL_2545272

EPI_ISL_2545275

EPI_ISL_2545380

EPI_ISL_1718636

EPI_ISL_1718637

EPI_ISL_2545282

EPI_ISL_2545285

EPI_ISL_2545289

EPI_ISL_1718638

EPI_ISL_1718639

EPI_ISL_1718640

EPI_ISL_1718642

EPI_ISL_1718643

EPI_ISL_1718644

EPI_ISL_1718645

EPI_ISL_2678879

EPI_ISL_2545301

EPI_ISL_2678881

EPI_ISL_1718646

EPI_ISL_1718647

EPI_ISL_2678890

EPI_ISL_2342096

EPI_ISL_2678892

EPI_ISL_2545308

EPI_ISL_2545309

EPI_ISL_2545310

EPI_ISL_2545312

EPI_ISL_2678897

EPI_ISL_2678898

EPI_ISL_1718648

EPI_ISL_2678899

EPI_ISL_2678900

EPI_ISL_1718649

EPI_ISL_2678903

EPI_ISL_2678906

EPI_ISL_1718650

EPI_ISL_2678913

EPI_ISL_2678921

EPI_ISL_2678922

EPI_ISL_2545336

EPI_ISL_2545337

EPI_ISL_2678925

EPI_ISL_2678927

EPI_ISL_2678928

EPI_ISL_2545338

EPI_ISL_2545339

EPI_ISL_2545340

EPI_ISL_2545342

EPI_ISL_2545344

EPI_ISL_2545345

EPI_ISL_2545346

EPI_ISL_2545347

EPI_ISL_1718654

EPI_ISL_2545348

EPI_ISL_2545349

EPI_ISL_2545350

EPI_ISL_2545351

EPI_ISL_2545352

EPI_ISL_2545354

EPI_ISL_2545355

EPI_ISL_1718658

EPI_ISL_2545356

EPI_ISL_2545357

EPI_ISL_2545358

EPI_ISL_1718660

EPI_ISL_2545359

EPI_ISL_2545361

EPI_ISL_2545362

EPI_ISL_1718661

EPI_ISL_2545363

EPI_ISL_2545364

EPI_ISL_1927934

EPI_ISL_2545365

EPI_ISL_1718662

EPI_ISL_2545366

EPI_ISL_2545367

EPI_ISL_2765340

EPI_ISL_2545368

EPI_ISL_1718664

EPI_ISL_2545370

EPI_ISL_2545371

EPI_ISL_2545372

EPI_ISL_2545373

EPI_ISL_2545374

EPI_ISL_2545377

EPI_ISL_2545378

EPI_ISL_2545381

EPI_ISL_2545383

EPI_ISL_2545384

EPI_ISL_2545385

EPI_ISL_1718665

EPI_ISL_1718667

EPI_ISL_2545386

EPI_ISL_2545387

EPI_ISL_2545388

EPI_ISL_2679154

EPI_ISL_2545389

EPI_ISL_2545390

EPI_ISL_2545393

EPI_ISL_1718670

EPI_ISL_1718671

EPI_ISL_2545394

EPI_ISL_2545395

EPI_ISL_2545396

EPI_ISL_1718672

EPI_ISL_2545397

EPI_ISL_2545398

EPI_ISL_2545400

EPI_ISL_1718676

EPI_ISL_2545402

EPI_ISL_2545406

EPI_ISL_1718678

EPI_ISL_2545407

EPI_ISL_1718680

EPI_ISL_1718682

EPI_ISL_1718684

EPI_ISL_2545420

EPI_ISL_1718685

EPI_ISL_1718686

EPI_ISL_2679002

EPI_ISL_2679003

EPI_ISL_2679005

EPI_ISL_2679008

EPI_ISL_1923897

EPI_ISL_1718688

EPI_ISL_2679015

EPI_ISL_1718692

EPI_ISL_1718693

EPI_ISL_2679024

EPI_ISL_1718694

EPI_ISL_2679030

EPI_ISL_2679031

EPI_ISL_2679032

EPI_ISL_1718695

EPI_ISL_1718696

EPI_ISL_2679034

EPI_ISL_1718698

EPI_ISL_1718699

EPI_ISL_2679040

EPI_ISL_1757794

EPI_ISL_1718702

EPI_ISL_2679048

EPI_ISL_2679053

EPI_ISL_1923947

EPI_ISL_1718703

EPI_ISL_1718704

EPI_ISL_1718705

EPI_ISL_1718707

EPI_ISL_1718708

EPI_ISL_1718709

EPI_ISL_1718710

EPI_ISL_1718711

EPI_ISL_1718712

EPI_ISL_1718713

EPI_ISL_1718714

EPI_ISL_1718716

EPI_ISL_1718717

EPI_ISL_1718722

EPI_ISL_1718724

EPI_ISL_1718725

EPI_ISL_1718726

EPI_ISL_1718728

EPI_ISL_1718729

EPI_ISL_1718730

EPI_ISL_1718732

EPI_ISL_1718733

EPI_ISL_1718734

EPI_ISL_1718735

EPI_ISL_1718736

EPI_ISL_1718737

EPI_ISL_1718739

EPI_ISL_1718740

EPI_ISL_1718743

EPI_ISL_1718748

EPI_ISL_1718749

EPI_ISL_1718750

EPI_ISL_1718751

EPI_ISL_1718753

EPI_ISL_1718754

EPI_ISL_1718755

EPI_ISL_1718756

EPI_ISL_1718757

EPI_ISL_1718759

EPI_ISL_1718761

EPI_ISL_1718762

EPI_ISL_1718763

EPI_ISL_1718764

EPI_ISL_1718765

EPI_ISL_1718766

EPI_ISL_1718767

EPI_ISL_1718768

EPI_ISL_1718769

EPI_ISL_1718773

EPI_ISL_1718774

EPI_ISL_1718776

EPI_ISL_1718777

EPI_ISL_1718780

EPI_ISL_1718782

EPI_ISL_1718783

EPI_ISL_1718787

EPI_ISL_1718788

EPI_ISL_1718789

EPI_ISL_1718790

EPI_ISL_1718791

EPI_ISL_1718792

EPI_ISL_1718794

EPI_ISL_2679060

EPI_ISL_1718795

EPI_ISL_1718796

EPI_ISL_1718797

EPI_ISL_2679066

EPI_ISL_1718798

EPI_ISL_2679070

EPI_ISL_1718800

EPI_ISL_2545581

EPI_ISL_1718801

EPI_ISL_1718802

EPI_ISL_2545582

EPI_ISL_2679082

EPI_ISL_2679084

EPI_ISL_1718804

EPI_ISL_2679092

EPI_ISL_2679093

EPI_ISL_1718805

EPI_ISL_1718807

EPI_ISL_1718808

EPI_ISL_1718810

EPI_ISL_1718814

EPI_ISL_1718816

EPI_ISL_1718817

EPI_ISL_1718818

EPI_ISL_1718821

EPI_ISL_1718822

EPI_ISL_1718824

EPI_ISL_1718825

EPI_ISL_1718827

EPI_ISL_1718828

EPI_ISL_1718829

EPI_ISL_1718830

EPI_ISL_1718831

EPI_ISL_1718832

EPI_ISL_1718833

EPI_ISL_1924072

EPI_ISL_1718836

EPI_ISL_1718837

EPI_ISL_1718838

EPI_ISL_1718839

EPI_ISL_1718840

EPI_ISL_1718842

EPI_ISL_2545583

EPI_ISL_2545584

EPI_ISL_1718844

EPI_ISL_1718845

EPI_ISL_1718846

EPI_ISL_1718847

EPI_ISL_1718850

EPI_ISL_1718851

EPI_ISL_1718852

EPI_ISL_1718853

EPI_ISL_1718854

EPI_ISL_1718855

EPI_ISL_1718857

EPI_ISL_1718860

EPI_ISL_1718861

EPI_ISL_1718862

EPI_ISL_1718863

EPI_ISL_1718865

EPI_ISL_1718866

EPI_ISL_1718867

EPI_ISL_1718868

EPI_ISL_1718869

EPI_ISL_1718870

EPI_ISL_1718871

EPI_ISL_1718873

EPI_ISL_1718874

EPI_ISL_1718875

EPI_ISL_1718878

EPI_ISL_1718879

EPI_ISL_1718880

EPI_ISL_1718881

EPI_ISL_1718883

EPI_ISL_1718884

EPI_ISL_1718885

EPI_ISL_1718886

EPI_ISL_1718887

EPI_ISL_1718888

EPI_ISL_3785646

EPI_ISL_1718891

EPI_ISL_1718893

EPI_ISL_2679131

EPI_ISL_1718895

EPI_ISL_1758069

EPI_ISL_1924158

EPI_ISL_1718897

EPI_ISL_1718898

EPI_ISL_1718899

EPI_ISL_2725462

EPI_ISL_1718900

EPI_ISL_1718901

EPI_ISL_1718903

EPI_ISL_1718904

EPI_ISL_1718905

EPI_ISL_2679144

EPI_ISL_2679145

EPI_ISL_2679147

EPI_ISL_1718907

EPI_ISL_1718908

EPI_ISL_1718909

EPI_ISL_1718911

EPI_ISL_1718912

EPI_ISL_1718913

EPI_ISL_1718915

EPI_ISL_3544246

EPI_ISL_1718916

EPI_ISL_1718917

EPI_ISL_1718918

EPI_ISL_1718919

EPI_ISL_1718920

EPI_ISL_1718921

EPI_ISL_1718923

EPI_ISL_1718924

EPI_ISL_3544250

EPI_ISL_1718925

EPI_ISL_2679166

EPI_ISL_1718926

EPI_ISL_1718927

EPI_ISL_1718928

EPI_ISL_1718929

EPI_ISL_1718930

EPI_ISL_1718932

EPI_ISL_2679171

EPI_ISL_2679173

EPI_ISL_1718933

EPI_ISL_1718936

EPI_ISL_1718937

EPI_ISL_1718938

EPI_ISL_1718939

EPI_ISL_1718941

EPI_ISL_2679176

EPI_ISL_1718943

EPI_ISL_1718944

EPI_ISL_1718945

EPI_ISL_1718946

EPI_ISL_1718947

EPI_ISL_1718948

EPI_ISL_1718949

EPI_ISL_1718950

EPI_ISL_2679179

EPI_ISL_1718952

EPI_ISL_1718953

EPI_ISL_2679181

EPI_ISL_1718954

EPI_ISL_2679185

EPI_ISL_1718957

EPI_ISL_1718958

EPI_ISL_1718960

EPI_ISL_2679189

EPI_ISL_2679190

EPI_ISL_1718961

EPI_ISL_1718962

EPI_ISL_1718963

EPI_ISL_1718965

EPI_ISL_3785655

EPI_ISL_1718966

EPI_ISL_1718967

EPI_ISL_2679192

EPI_ISL_1718968

EPI_ISL_2679199

EPI_ISL_2679204

EPI_ISL_1718970

EPI_ISL_1718971

EPI_ISL_1718972

EPI_ISL_1718975

EPI_ISL_1718976

EPI_ISL_1718977

EPI_ISL_1718980

EPI_ISL_1718982

EPI_ISL_1718984

EPI_ISL_2679217

EPI_ISL_1718985

EPI_ISL_1718988

EPI_ISL_1718990

EPI_ISL_1718992

EPI_ISL_1718993

EPI_ISL_1718994

EPI_ISL_1718995

EPI_ISL_1718996

EPI_ISL_1718999

EPI_ISL_1719001

EPI_ISL_1719002

EPI_ISL_1719003

EPI_ISL_1719005

EPI_ISL_1719006

EPI_ISL_1719007

EPI_ISL_1719010

EPI_ISL_1719011

EPI_ISL_1719012

EPI_ISL_1719013

EPI_ISL_1719014

EPI_ISL_1719017

EPI_ISL_1719019

EPI_ISL_1719020

EPI_ISL_1719021

EPI_ISL_1719023

EPI_ISL_2679224

EPI_ISL_1719028

EPI_ISL_2679226

EPI_ISL_1719029

EPI_ISL_1719030

EPI_ISL_1719031

EPI_ISL_1719032

EPI_ISL_1719033

EPI_ISL_1719034

EPI_ISL_1719036

EPI_ISL_1719037

EPI_ISL_1719038

EPI_ISL_1719039

EPI_ISL_1719040

EPI_ISL_1719041

EPI_ISL_3785786

EPI_ISL_1719043

EPI_ISL_1719044

EPI_ISL_1719045

EPI_ISL_1719046

EPI_ISL_1719047

EPI_ISL_1719049

EPI_ISL_1719050

EPI_ISL_1719051

EPI_ISL_1719052

EPI_ISL_1719053

EPI_ISL_1719054

EPI_ISL_1719055

EPI_ISL_1719057

EPI_ISL_1719059

EPI_ISL_1719060

EPI_ISL_1719061

EPI_ISL_1719063

EPI_ISL_1719064

EPI_ISL_1719065

EPI_ISL_1719068

EPI_ISL_1719069

EPI_ISL_1719070

EPI_ISL_1719071

EPI_ISL_1719072

EPI_ISL_1719073

EPI_ISL_1719074

EPI_ISL_1719075

EPI_ISL_1719077

EPI_ISL_1719078

EPI_ISL_1719080

EPI_ISL_1719081

EPI_ISL_1719082

EPI_ISL_1719085

EPI_ISL_1719086

EPI_ISL_1719087

EPI_ISL_1719088

EPI_ISL_1719090

EPI_ISL_1924202

EPI_ISL_1719093

EPI_ISL_1719094

EPI_ISL_1719095

EPI_ISL_1719100

EPI_ISL_1719101

EPI_ISL_1719102

EPI_ISL_1719103

EPI_ISL_1719104

EPI_ISL_1719105

EPI_ISL_1719106

EPI_ISL_1719110

EPI_ISL_1719112

EPI_ISL_1719113

EPI_ISL_1719114

EPI_ISL_1719115

EPI_ISL_1719116

EPI_ISL_1719117

EPI_ISL_1719119

EPI_ISL_1719121

EPI_ISL_1719122

EPI_ISL_1719123

EPI_ISL_1719124

EPI_ISL_1719125

EPI_ISL_1719129

EPI_ISL_1719131

EPI_ISL_1719132

EPI_ISL_1719133

EPI_ISL_1719137

EPI_ISL_1719138

EPI_ISL_1719139

EPI_ISL_1719140

EPI_ISL_1719141

EPI_ISL_1719145

EPI_ISL_1719146

EPI_ISL_3544268

EPI_ISL_1719147

EPI_ISL_1719149

EPI_ISL_1719151

EPI_ISL_1719152

EPI_ISL_1719153

EPI_ISL_1719155

EPI_ISL_1719156

EPI_ISL_1719157

EPI_ISL_1719158

EPI_ISL_1719159

EPI_ISL_1719160

EPI_ISL_1719161

EPI_ISL_1719163

EPI_ISL_1719164

EPI_ISL_1719165

EPI_ISL_1719167

EPI_ISL_1719168

EPI_ISL_1719169

EPI_ISL_1719170

EPI_ISL_1719171

EPI_ISL_1719172

EPI_ISL_1719173

EPI_ISL_1719174

EPI_ISL_1719175

EPI_ISL_1719176

EPI_ISL_1719177

EPI_ISL_1719178

EPI_ISL_1719179

EPI_ISL_1719180

EPI_ISL_1719181

EPI_ISL_1719185

EPI_ISL_1719186

EPI_ISL_1719187

EPI_ISL_1719189

EPI_ISL_1719190

EPI_ISL_1719192

EPI_ISL_1719193

EPI_ISL_1719195

EPI_ISL_1719196

EPI_ISL_1719197

EPI_ISL_1719199

EPI_ISL_1719200

EPI_ISL_1719202

EPI_ISL_1719203

EPI_ISL_1719204

EPI_ISL_1719205

EPI_ISL_1719206

EPI_ISL_1719207

EPI_ISL_1719208

EPI_ISL_1719209

EPI_ISL_1719210

EPI_ISL_1719213

EPI_ISL_1719214

EPI_ISL_1719215

EPI_ISL_1719216

EPI_ISL_1719217

EPI_ISL_1719218

EPI_ISL_1719219

EPI_ISL_1719220

EPI_ISL_1719221

EPI_ISL_1719222

EPI_ISL_1719223

EPI_ISL_1719224

EPI_ISL_1719226

EPI_ISL_1719227

EPI_ISL_1719229

EPI_ISL_1719230

EPI_ISL_1719231

EPI_ISL_1719232

EPI_ISL_1719233

EPI_ISL_1719234

EPI_ISL_1719235

EPI_ISL_1719236

EPI_ISL_1719237

EPI_ISL_1719238

EPI_ISL_1719240

EPI_ISL_1719241

EPI_ISL_1719242

EPI_ISL_1719243

EPI_ISL_1719245

EPI_ISL_1719246

EPI_ISL_1719247

EPI_ISL_1719248

EPI_ISL_1719249

EPI_ISL_1719250

EPI_ISL_1719251

EPI_ISL_1719252

EPI_ISL_1719253

EPI_ISL_1719254

EPI_ISL_1719255

EPI_ISL_1719256

EPI_ISL_1719257

EPI_ISL_1719258

EPI_ISL_1719259

EPI_ISL_1719260

EPI_ISL_1719262

EPI_ISL_1719263

EPI_ISL_1719264

EPI_ISL_1719265

EPI_ISL_1719266

EPI_ISL_1719267

EPI_ISL_1719270

EPI_ISL_1719271

EPI_ISL_1719272

EPI_ISL_1719273

EPI_ISL_1719274

EPI_ISL_1719275

EPI_ISL_3785844

EPI_ISL_1719276

EPI_ISL_1719277

EPI_ISL_1719278

EPI_ISL_1719279

EPI_ISL_1719280

EPI_ISL_1719281

EPI_ISL_3785724

EPI_ISL_2725766

EPI_ISL_1719283

EPI_ISL_1719284

EPI_ISL_1719285

EPI_ISL_1719286

EPI_ISL_1719287

EPI_ISL_1719288

EPI_ISL_1719291

EPI_ISL_1719292

EPI_ISL_1719294

EPI_ISL_1719295

EPI_ISL_1719296

EPI_ISL_1719297

EPI_ISL_1719298

EPI_ISL_1719299

EPI_ISL_1719300

EPI_ISL_1719301

EPI_ISL_1719302

EPI_ISL_1719303

EPI_ISL_1719305

EPI_ISL_1719306

EPI_ISL_1719308

EPI_ISL_1719309

EPI_ISL_1719310

EPI_ISL_1719311

EPI_ISL_1719312

EPI_ISL_1719313

EPI_ISL_1719314

EPI_ISL_1719315

EPI_ISL_1719316

EPI_ISL_1719317

EPI_ISL_1719318

EPI_ISL_1719319

EPI_ISL_1719320

EPI_ISL_1719321

EPI_ISL_1719322

EPI_ISL_1719323

EPI_ISL_1719324

EPI_ISL_1719325

EPI_ISL_1719326

EPI_ISL_1719327

EPI_ISL_1719328

EPI_ISL_1719329

EPI_ISL_1719330

EPI_ISL_1719332

EPI_ISL_1719333

EPI_ISL_1719334

EPI_ISL_1719335

EPI_ISL_1719336

EPI_ISL_1719337

EPI_ISL_1719338

EPI_ISL_1719340

EPI_ISL_1719341

EPI_ISL_1719342

EPI_ISL_1719343

EPI_ISL_1719344

EPI_ISL_1719345

EPI_ISL_1719347

EPI_ISL_1719348

EPI_ISL_1719349

EPI_ISL_1719351

EPI_ISL_1719353

EPI_ISL_1719354

EPI_ISL_1719355

EPI_ISL_1719356

EPI_ISL_1719709

EPI_ISL_1719357

EPI_ISL_1719358

EPI_ISL_1719359

EPI_ISL_1719360

EPI_ISL_1719363

EPI_ISL_1719364

EPI_ISL_1719365

EPI_ISL_1719367

EPI_ISL_1719368

EPI_ISL_1719369

EPI_ISL_1719370

EPI_ISL_1719371

EPI_ISL_1719372

EPI_ISL_1719373

EPI_ISL_1719374

EPI_ISL_1719375

EPI_ISL_1719376

EPI_ISL_1719377

EPI_ISL_1719378

EPI_ISL_1719379

EPI_ISL_1719380

EPI_ISL_1719381

EPI_ISL_1719383

EPI_ISL_1719384

EPI_ISL_1719385

EPI_ISL_1719386

EPI_ISL_1719387

EPI_ISL_1719388

EPI_ISL_1719389

EPI_ISL_1719390

EPI_ISL_1719391

EPI_ISL_1719392

EPI_ISL_1719393

EPI_ISL_1719394

EPI_ISL_1719395

EPI_ISL_1719396

EPI_ISL_1719397

EPI_ISL_1719399

EPI_ISL_1719400

EPI_ISL_1719401

EPI_ISL_1719402

EPI_ISL_1719404

EPI_ISL_1719405

EPI_ISL_1719406

EPI_ISL_1719407

EPI_ISL_1719408

EPI_ISL_1719409

EPI_ISL_2545822

EPI_ISL_2545823

EPI_ISL_1719410

EPI_ISL_1719411

EPI_ISL_1719412

EPI_ISL_1719414

EPI_ISL_1719415

EPI_ISL_1719416

EPI_ISL_1719417

EPI_ISL_1719418

EPI_ISL_2545824

EPI_ISL_1719420

EPI_ISL_2545825

EPI_ISL_2545826

EPI_ISL_1719422

EPI_ISL_1719423

EPI_ISL_1719424

EPI_ISL_2545827

EPI_ISL_1719425

EPI_ISL_1719426

EPI_ISL_2545828

EPI_ISL_2545829

EPI_ISL_2545830

EPI_ISL_1719427

EPI_ISL_1719428

EPI_ISL_1719429

EPI_ISL_1719430

EPI_ISL_1719431

EPI_ISL_1719432

EPI_ISL_1719433

EPI_ISL_2545832

EPI_ISL_1719434

EPI_ISL_1719435

EPI_ISL_2545833

EPI_ISL_1719436

EPI_ISL_2545834

EPI_ISL_2545835

EPI_ISL_2545836

EPI_ISL_1719437

EPI_ISL_1719438

EPI_ISL_2545838

EPI_ISL_2545839

EPI_ISL_2545840

EPI_ISL_2545841

EPI_ISL_1719440

EPI_ISL_1719442

EPI_ISL_2545842

EPI_ISL_1719443

EPI_ISL_1719444

EPI_ISL_1719446

EPI_ISL_1719448

EPI_ISL_1719449

EPI_ISL_1719450

EPI_ISL_2545843

EPI_ISL_2545844

EPI_ISL_1719452

EPI_ISL_1719453

EPI_ISL_1719454

EPI_ISL_1719455

EPI_ISL_1719456

EPI_ISL_1719457

EPI_ISL_1719458

EPI_ISL_1719459

EPI_ISL_1719460

EPI_ISL_1719461

EPI_ISL_1719462

EPI_ISL_1719463

EPI_ISL_1719464

EPI_ISL_1719465

EPI_ISL_1719468

EPI_ISL_1719469

EPI_ISL_1719470

EPI_ISL_1719471

EPI_ISL_1719472

EPI_ISL_2545845

EPI_ISL_2545846

EPI_ISL_2545847

EPI_ISL_2545848

EPI_ISL_2545849

EPI_ISL_2545850

EPI_ISL_2545851

EPI_ISL_1719473

EPI_ISL_2545854

EPI_ISL_1719474

EPI_ISL_2545855

EPI_ISL_2545856

EPI_ISL_2545857

EPI_ISL_2545858

EPI_ISL_2545859

EPI_ISL_2545860

EPI_ISL_2545861

EPI_ISL_2545862

EPI_ISL_1719710

EPI_ISL_2545863

EPI_ISL_2545864

EPI_ISL_1719475

EPI_ISL_2545865

EPI_ISL_1923664

EPI_ISL_2545867

EPI_ISL_2545868

EPI_ISL_2545869

EPI_ISL_1719477

EPI_ISL_2545870

EPI_ISL_2545871

EPI_ISL_2545872

EPI_ISL_2545873

EPI_ISL_2545874

EPI_ISL_2545875

EPI_ISL_1719479

EPI_ISL_2545877

EPI_ISL_2545878

EPI_ISL_1719480

EPI_ISL_1719481

EPI_ISL_1719484

EPI_ISL_1719485

EPI_ISL_1719486

EPI_ISL_1719487

EPI_ISL_1719488

EPI_ISL_1719489

EPI_ISL_1719490

EPI_ISL_2545879

EPI_ISL_2545880

EPI_ISL_2545881

EPI_ISL_2545882

EPI_ISL_1719491

EPI_ISL_1719492

EPI_ISL_1719494

EPI_ISL_1719495

EPI_ISL_1719496

EPI_ISL_2545883

EPI_ISL_1719497

EPI_ISL_1719498

EPI_ISL_1719711

EPI_ISL_1719499

EPI_ISL_1719500

EPI_ISL_2545884

EPI_ISL_1719501

EPI_ISL_1719502

EPI_ISL_1719503

EPI_ISL_2545885

EPI_ISL_1719504

EPI_ISL_1719505

EPI_ISL_1719506

EPI_ISL_1719507

EPI_ISL_1719508

EPI_ISL_1719509

EPI_ISL_1719510

EPI_ISL_1719511

EPI_ISL_1719512

EPI_ISL_1719513

EPI_ISL_1719514

EPI_ISL_2545886

EPI_ISL_1719515

EPI_ISL_1719516

EPI_ISL_1719517

EPI_ISL_1719518

EPI_ISL_1719519

EPI_ISL_1719520

EPI_ISL_1719521

EPI_ISL_2545887

EPI_ISL_2545888

EPI_ISL_1923303

EPI_ISL_2545889

EPI_ISL_2545890

EPI_ISL_2545891

EPI_ISL_2545892

EPI_ISL_2545893

EPI_ISL_1719523

EPI_ISL_1719524

EPI_ISL_1719525

EPI_ISL_1719526

EPI_ISL_1719528

EPI_ISL_2545894

EPI_ISL_2545895

EPI_ISL_1719532

EPI_ISL_1719533

EPI_ISL_1719534

EPI_ISL_1719535

EPI_ISL_1719536

EPI_ISL_2545896

EPI_ISL_1719537

EPI_ISL_1719538

EPI_ISL_1719540

EPI_ISL_1719541

EPI_ISL_1719542

EPI_ISL_1719543

EPI_ISL_1719544

EPI_ISL_1582804

EPI_ISL_1582805

EPI_ISL_1719545

EPI_ISL_1719546

EPI_ISL_1719547

EPI_ISL_1719548

EPI_ISL_1719549

EPI_ISL_1719550

EPI_ISL_1719551

EPI_ISL_2545897

EPI_ISL_2545898

EPI_ISL_1719554

EPI_ISL_2545899

EPI_ISL_2545900

EPI_ISL_1719555

EPI_ISL_1719556

EPI_ISL_1719557

EPI_ISL_1719558

EPI_ISL_1719559

EPI_ISL_1719560

EPI_ISL_1719561

EPI_ISL_2545901

EPI_ISL_2545902

EPI_ISL_2545903

EPI_ISL_2545904

EPI_ISL_1719562

EPI_ISL_1719563

EPI_ISL_1719564

EPI_ISL_2545905

EPI_ISL_2545906

EPI_ISL_1719565

EPI_ISL_2545907

EPI_ISL_1719566

EPI_ISL_1719567

EPI_ISL_1719568

EPI_ISL_1719569

EPI_ISL_2545908

EPI_ISL_1719570

EPI_ISL_1719571

EPI_ISL_1719572

EPI_ISL_1719574

EPI_ISL_1719575

EPI_ISL_1719576

EPI_ISL_2545910

EPI_ISL_2545911

EPI_ISL_1719577

EPI_ISL_2545913

EPI_ISL_2545914

EPI_ISL_2545915

EPI_ISL_1719578

EPI_ISL_1719579

EPI_ISL_1719581

EPI_ISL_1719582

EPI_ISL_1719583

EPI_ISL_1719585

EPI_ISL_2545916

EPI_ISL_1719586

EPI_ISL_1719587

EPI_ISL_1719588

EPI_ISL_1719589

EPI_ISL_1719592

EPI_ISL_1719593

EPI_ISL_1719594

EPI_ISL_1719596

EPI_ISL_1719597

EPI_ISL_1719598

EPI_ISL_1719599

EPI_ISL_1719600

EPI_ISL_1719601

EPI_ISL_1719602

EPI_ISL_1719603

EPI_ISL_1719604

EPI_ISL_1719606

EPI_ISL_2545918

EPI_ISL_1719607

EPI_ISL_1719608

EPI_ISL_2545919

EPI_ISL_2545920

EPI_ISL_1719609

EPI_ISL_2545921

EPI_ISL_1719610

EPI_ISL_1719611

EPI_ISL_1719612

EPI_ISL_1719613

EPI_ISL_2545922

EPI_ISL_1923319

EPI_ISL_1719614

EPI_ISL_2545926

EPI_ISL_2545927

EPI_ISL_2545928

EPI_ISL_2545930

EPI_ISL_2545931

EPI_ISL_2545932

EPI_ISL_2545933

EPI_ISL_1719615

EPI_ISL_2545934

EPI_ISL_2545935

EPI_ISL_2545937

EPI_ISL_2545938

EPI_ISL_2545939

EPI_ISL_2545941

EPI_ISL_2545942

EPI_ISL_2545943

EPI_ISL_2545944

EPI_ISL_2545945

EPI_ISL_2545946

EPI_ISL_1719617

EPI_ISL_1760187

EPI_ISL_1923677

EPI_ISL_2545947

EPI_ISL_1923311

EPI_ISL_2545949

EPI_ISL_2545950

EPI_ISL_2545951

EPI_ISL_2545952

EPI_ISL_2545953

EPI_ISL_1719619

EPI_ISL_2545954

EPI_ISL_2545956

EPI_ISL_2545957

EPI_ISL_2545958

EPI_ISL_2545959

EPI_ISL_2545960

EPI_ISL_1719620

EPI_ISL_2545961

EPI_ISL_2545962

EPI_ISL_1923434

EPI_ISL_2545965

EPI_ISL_1719621

EPI_ISL_2545967

EPI_ISL_2545968

EPI_ISL_2545969

EPI_ISL_2545970

EPI_ISL_2545971

EPI_ISL_1719622

EPI_ISL_2545972

EPI_ISL_2545973

EPI_ISL_1719624

EPI_ISL_2545974

EPI_ISL_2545975

EPI_ISL_2545976

EPI_ISL_2545978

EPI_ISL_1719625

EPI_ISL_2545979

EPI_ISL_2545981

EPI_ISL_2545982

EPI_ISL_2545983

EPI_ISL_2545984

EPI_ISL_2545985

EPI_ISL_2545986

EPI_ISL_3785509

EPI_ISL_1719626

EPI_ISL_1719627

EPI_ISL_1719628

EPI_ISL_2545989

EPI_ISL_1923406

EPI_ISL_1719629

EPI_ISL_1719630

EPI_ISL_1719631

EPI_ISL_1923408

EPI_ISL_1719633

EPI_ISL_1923400

EPI_ISL_2725949

EPI_ISL_6181176

EPI_ISL_3543805

EPI_ISL_3543806

EPI_ISL_1719634

EPI_ISL_1719636

EPI_ISL_1719637

EPI_ISL_1719639

EPI_ISL_1719640

EPI_ISL_1719641

EPI_ISL_1719643

EPI_ISL_1719644

EPI_ISL_1719645

EPI_ISL_1719646

EPI_ISL_1719648

EPI_ISL_1719650

EPI_ISL_1719651

EPI_ISL_1719652

EPI_ISL_1719653

EPI_ISL_1719654

EPI_ISL_1719656

EPI_ISL_1719657

EPI_ISL_1719658

EPI_ISL_1719659

EPI_ISL_1719660

EPI_ISL_1719661

EPI_ISL_1760286

EPI_ISL_1719662

EPI_ISL_1719663

EPI_ISL_1719664

EPI_ISL_1719665

EPI_ISL_1719666

EPI_ISL_1719667

EPI_ISL_1719669

EPI_ISL_5392882

EPI_ISL_1719670

EPI_ISL_1719671

EPI_ISL_6181185

EPI_ISL_1719672

EPI_ISL_1719673

EPI_ISL_1719675

EPI_ISL_1719676

EPI_ISL_1719677

EPI_ISL_1719678

EPI_ISL_1719679

EPI_ISL_1719681

EPI_ISL_1719682

EPI_ISL_1719683

EPI_ISL_1719684

EPI_ISL_1719686

EPI_ISL_1719687

EPI_ISL_1719688

EPI_ISL_1719689

EPI_ISL_1719690

EPI_ISL_1719692

EPI_ISL_1719693

EPI_ISL_1719696

EPI_ISL_1719697

EPI_ISL_1719698

EPI_ISL_1719699

EPI_ISL_1719700

EPI_ISL_1719701

EPI_ISL_1719702

EPI_ISL_1719704

EPI_ISL_1719705

EPI_ISL_1719706

EPI_ISL_1719707

EPI_ISL_1719712

EPI_ISL_1719713

EPI_ISL_1719714

EPI_ISL_1719715

EPI_ISL_1719716

EPI_ISL_1719717

EPI_ISL_1719718

EPI_ISL_1719719

EPI_ISL_1719720

EPI_ISL_1719721

EPI_ISL_1719722

EPI_ISL_1719723

EPI_ISL_1719724

EPI_ISL_1719725

EPI_ISL_1719726

EPI_ISL_1719727

EPI_ISL_1719729

EPI_ISL_1719730

EPI_ISL_1719731

EPI_ISL_1719732

EPI_ISL_1719733

EPI_ISL_1719734

EPI_ISL_1719735

EPI_ISL_1719737

EPI_ISL_1719738

EPI_ISL_1719739

EPI_ISL_1719741

EPI_ISL_1719742

EPI_ISL_1719744

EPI_ISL_1719745

EPI_ISL_1719746

EPI_ISL_1719747

EPI_ISL_1719748

EPI_ISL_1719749

EPI_ISL_1719752

EPI_ISL_1719753

EPI_ISL_1719754

EPI_ISL_1719755

EPI_ISL_1719756

EPI_ISL_1719757

EPI_ISL_1719758

EPI_ISL_1719759

EPI_ISL_1719761

EPI_ISL_1719762

EPI_ISL_1719763

EPI_ISL_1719764

EPI_ISL_1719765

EPI_ISL_1719767

EPI_ISL_1719768

EPI_ISL_1719769

EPI_ISL_1719771

EPI_ISL_1719772

EPI_ISL_1719773

EPI_ISL_1719774

EPI_ISL_1719775

EPI_ISL_1719776

EPI_ISL_1719777

EPI_ISL_1719779

EPI_ISL_1719780

EPI_ISL_1719781

EPI_ISL_1719782

EPI_ISL_1719783

EPI_ISL_1719784

EPI_ISL_1719785

EPI_ISL_1719786

EPI_ISL_1719787

EPI_ISL_1719788

EPI_ISL_1719789

EPI_ISL_1719791

EPI_ISL_1719792

EPI_ISL_1719793

EPI_ISL_1719795

EPI_ISL_1719798

EPI_ISL_1719799

EPI_ISL_1719800

EPI_ISL_1719801

EPI_ISL_1719802

EPI_ISL_1719803

EPI_ISL_1719806

EPI_ISL_1719807

EPI_ISL_1719808

EPI_ISL_1719810

EPI_ISL_1719811

EPI_ISL_1719812

EPI_ISL_1719813

EPI_ISL_1760602

EPI_ISL_1719815

EPI_ISL_1719816

EPI_ISL_1719817

EPI_ISL_1719818

EPI_ISL_1719820

EPI_ISL_1719821

EPI_ISL_1719822

EPI_ISL_1719823

EPI_ISL_1719824

EPI_ISL_1719825

EPI_ISL_1719826

EPI_ISL_1760611

EPI_ISL_1719828

EPI_ISL_1719829

EPI_ISL_1719830

EPI_ISL_1719831

EPI_ISL_1719832

EPI_ISL_1719834

EPI_ISL_1719836

EPI_ISL_1719837

EPI_ISL_1719838

EPI_ISL_1719839

EPI_ISL_1719840

EPI_ISL_1719842

EPI_ISL_1719844

EPI_ISL_1719846

EPI_ISL_1719860

EPI_ISL_1719866

EPI_ISL_1719878

EPI_ISL_1719879

EPI_ISL_1719880

EPI_ISL_1719881

EPI_ISL_1719882

EPI_ISL_1719883

EPI_ISL_1719884

EPI_ISL_2679530

EPI_ISL_2679531

EPI_ISL_2679536

EPI_ISL_2679552

EPI_ISL_2679554

EPI_ISL_1720172

EPI_ISL_1720173

EPI_ISL_1720175

EPI_ISL_1720176

EPI_ISL_1720177

EPI_ISL_1720179

EPI_ISL_1720180

EPI_ISL_1720182

EPI_ISL_1720183

EPI_ISL_1720184

EPI_ISL_1720187

EPI_ISL_1720188

EPI_ISL_1720191

EPI_ISL_1720193

EPI_ISL_1720196

EPI_ISL_1720198

EPI_ISL_1720200

EPI_ISL_1720203

EPI_ISL_1720208

EPI_ISL_1720209

EPI_ISL_1720211

EPI_ISL_1720212

EPI_ISL_1720213

EPI_ISL_1720214

EPI_ISL_1720215

EPI_ISL_1720216

EPI_ISL_1720218

EPI_ISL_1720219

EPI_ISL_1720220

EPI_ISL_1720221

EPI_ISL_1720222

EPI_ISL_1720225

EPI_ISL_1720228

EPI_ISL_1720229

EPI_ISL_1720233

EPI_ISL_1720234

EPI_ISL_1720235

EPI_ISL_1720236

EPI_ISL_1720241

EPI_ISL_1720250

EPI_ISL_1720252

EPI_ISL_1720260

EPI_ISL_1720268

EPI_ISL_1720279

EPI_ISL_1720281

EPI_ISL_1720287

EPI_ISL_1720293

EPI_ISL_1720301

EPI_ISL_1720302

EPI_ISL_1720305

EPI_ISL_1720306

EPI_ISL_1720309

EPI_ISL_1720310

EPI_ISL_1720311

EPI_ISL_1720312

EPI_ISL_1720314

EPI_ISL_1720315

EPI_ISL_1720318

EPI_ISL_1720324

EPI_ISL_1720326

EPI_ISL_1720327

EPI_ISL_1720329

EPI_ISL_1720335

EPI_ISL_1720339

EPI_ISL_1720340

EPI_ISL_1720342

EPI_ISL_1720347

EPI_ISL_1720352

EPI_ISL_1720357

EPI_ISL_1720366

EPI_ISL_1720367

EPI_ISL_1720370

EPI_ISL_1720371

EPI_ISL_1720377

EPI_ISL_1720381

EPI_ISL_1720385

EPI_ISL_1720391

EPI_ISL_1720398

EPI_ISL_1720402

EPI_ISL_1720406

EPI_ISL_1720408

EPI_ISL_1720411

EPI_ISL_1720413

EPI_ISL_1720414

EPI_ISL_1720415

EPI_ISL_1720416

EPI_ISL_1720418

EPI_ISL_1720419

EPI_ISL_1720422

EPI_ISL_1720424

EPI_ISL_1720425

EPI_ISL_1720426

EPI_ISL_1720428

EPI_ISL_1720433

EPI_ISL_1720434

EPI_ISL_1720435

EPI_ISL_1720436

EPI_ISL_1720437

EPI_ISL_1720439

EPI_ISL_1720442

EPI_ISL_1720444

EPI_ISL_1720445

EPI_ISL_1720446

EPI_ISL_1720448

EPI_ISL_1720455

EPI_ISL_1720457

EPI_ISL_1720461

EPI_ISL_1720462

EPI_ISL_1720463

EPI_ISL_1720464

EPI_ISL_1720465

EPI_ISL_1720466

EPI_ISL_1720467

EPI_ISL_1720468

EPI_ISL_1720470

EPI_ISL_1720473

EPI_ISL_1720474

EPI_ISL_1720476

EPI_ISL_1720478

EPI_ISL_1720483

EPI_ISL_1720487

EPI_ISL_1720488

EPI_ISL_1720491

EPI_ISL_1720492

EPI_ISL_1720493

EPI_ISL_1720494

EPI_ISL_2679566

EPI_ISL_1720496

EPI_ISL_1720497

EPI_ISL_1720499

EPI_ISL_1720500

EPI_ISL_1720502

EPI_ISL_1720504

EPI_ISL_1720505

EPI_ISL_1720507

EPI_ISL_1720508

EPI_ISL_1720509

EPI_ISL_1720510

EPI_ISL_1720513

EPI_ISL_1720516

EPI_ISL_1720517

EPI_ISL_1720518

EPI_ISL_1720522

EPI_ISL_1720527

EPI_ISL_1720529

EPI_ISL_1720530

EPI_ISL_1720531

EPI_ISL_2679576

EPI_ISL_2679577

EPI_ISL_1720535

EPI_ISL_1720536

EPI_ISL_1720537

EPI_ISL_1720539

EPI_ISL_1720540

EPI_ISL_1720541

EPI_ISL_1720542

EPI_ISL_1720543

EPI_ISL_1720544

EPI_ISL_1720545

EPI_ISL_1720546

EPI_ISL_2178594

EPI_ISL_1720548

EPI_ISL_1720549

EPI_ISL_1720550

EPI_ISL_1720552

EPI_ISL_1720554

EPI_ISL_1720555

EPI_ISL_1720556

EPI_ISL_1720557

EPI_ISL_1720559

EPI_ISL_1720560

EPI_ISL_1720562

EPI_ISL_1720565

EPI_ISL_1720566

EPI_ISL_1720568

EPI_ISL_1720569

EPI_ISL_1720570

EPI_ISL_1720571

EPI_ISL_1720573

EPI_ISL_1720575

EPI_ISL_1720578

EPI_ISL_1720579

EPI_ISL_1720580

EPI_ISL_1720583

EPI_ISL_1720584

EPI_ISL_1720585

EPI_ISL_1720588

EPI_ISL_1720589

EPI_ISL_1720591

EPI_ISL_1720593

EPI_ISL_1720594

EPI_ISL_1720595

EPI_ISL_1720596

EPI_ISL_1720597

EPI_ISL_1720599

EPI_ISL_1720601

EPI_ISL_1720607

EPI_ISL_1720608

EPI_ISL_1720610

EPI_ISL_1721493

EPI_ISL_1720615

EPI_ISL_1720617

EPI_ISL_1720620

EPI_ISL_1720621

EPI_ISL_1720623

EPI_ISL_1720624

EPI_ISL_1720625

EPI_ISL_1720626

EPI_ISL_1720627

EPI_ISL_1720630

EPI_ISL_1720631

EPI_ISL_1720632

EPI_ISL_1720634

EPI_ISL_1720635

EPI_ISL_1720636

EPI_ISL_1720637

EPI_ISL_1720638

EPI_ISL_1720639

EPI_ISL_1720642

EPI_ISL_1721497

EPI_ISL_1720644

EPI_ISL_1720645

EPI_ISL_1720646

EPI_ISL_1720648

EPI_ISL_1721499

EPI_ISL_1721500

EPI_ISL_1721502

EPI_ISL_1721506

EPI_ISL_1721508

EPI_ISL_1720653

EPI_ISL_1721510

EPI_ISL_1720655

EPI_ISL_1720658

EPI_ISL_1720659

EPI_ISL_1720660

EPI_ISL_1720661

EPI_ISL_1720662

EPI_ISL_1720663

EPI_ISL_1720664

EPI_ISL_1720665

EPI_ISL_1720666

EPI_ISL_1720667

EPI_ISL_1720668

EPI_ISL_1720670

EPI_ISL_2679658

EPI_ISL_1720671

EPI_ISL_1721576

EPI_ISL_1720676

EPI_ISL_1720678

EPI_ISL_1720680

EPI_ISL_1720681

EPI_ISL_1720686

EPI_ISL_1720687

EPI_ISL_1720688

EPI_ISL_1720689

EPI_ISL_1720691

EPI_ISL_1758658

EPI_ISL_1721579

EPI_ISL_2679660

EPI_ISL_1720693

EPI_ISL_1720694

EPI_ISL_1720696

EPI_ISL_1720699

EPI_ISL_1720700

EPI_ISL_1720701

EPI_ISL_1720702

EPI_ISL_1720703

EPI_ISL_1720705

EPI_ISL_1720706

EPI_ISL_1720707

EPI_ISL_1720709

EPI_ISL_1720710

EPI_ISL_1720711

EPI_ISL_2679661

EPI_ISL_1720713

EPI_ISL_1720714

EPI_ISL_1720719

EPI_ISL_1720721

EPI_ISL_1720723

EPI_ISL_1720724

EPI_ISL_1720725

EPI_ISL_1720726

EPI_ISL_1720727

EPI_ISL_1720728

EPI_ISL_1720729

EPI_ISL_2679664

EPI_ISL_1720730

EPI_ISL_1720731

EPI_ISL_1720732

EPI_ISL_1720739

EPI_ISL_1721513

EPI_ISL_1720743

EPI_ISL_1720744

EPI_ISL_2679666

EPI_ISL_1720748

EPI_ISL_1720750

EPI_ISL_1721514

EPI_ISL_1721515

EPI_ISL_1721518

EPI_ISL_1721522

EPI_ISL_1721523

EPI_ISL_1721524

EPI_ISL_1721527

EPI_ISL_1721591

EPI_ISL_2679671

EPI_ISL_2679673

EPI_ISL_3548532

EPI_ISL_1720775

EPI_ISL_1720776

EPI_ISL_1720777

EPI_ISL_1720778

EPI_ISL_1720779

EPI_ISL_1720780

EPI_ISL_1720782

EPI_ISL_2679681

EPI_ISL_2679682

EPI_ISL_2679688

EPI_ISL_2679690

EPI_ISL_1720787

EPI_ISL_1720789

EPI_ISL_1720790

EPI_ISL_2679693

EPI_ISL_1720791

EPI_ISL_2679697

EPI_ISL_1720794

EPI_ISL_2343931

EPI_ISL_2679703

EPI_ISL_2679705

EPI_ISL_1720802

EPI_ISL_1720805

EPI_ISL_1720807

EPI_ISL_1720808

EPI_ISL_1720809

EPI_ISL_1720810

EPI_ISL_1720811

EPI_ISL_1720812

EPI_ISL_1720815

EPI_ISL_1720818

EPI_ISL_1720819

EPI_ISL_1720822

EPI_ISL_2679716

EPI_ISL_2679720

EPI_ISL_2679722

EPI_ISL_1720832

EPI_ISL_1720836

EPI_ISL_1720841

EPI_ISL_2679725

EPI_ISL_2679727

EPI_ISL_1720846

EPI_ISL_1720853

EPI_ISL_1720854

EPI_ISL_1720858

EPI_ISL_1720860

EPI_ISL_1720861

EPI_ISL_1720862

EPI_ISL_1720863

EPI_ISL_1720865

EPI_ISL_1720867

EPI_ISL_1720868

EPI_ISL_1720870

EPI_ISL_2679729

EPI_ISL_1720876

EPI_ISL_1720877

EPI_ISL_1720881

EPI_ISL_1720885

EPI_ISL_1720886

EPI_ISL_1720888

EPI_ISL_2679731

EPI_ISL_1720890

EPI_ISL_1720892

EPI_ISL_1720893

EPI_ISL_1720898

EPI_ISL_1720901

EPI_ISL_2679739

EPI_ISL_2343996

EPI_ISL_2679746

EPI_ISL_2344005

EPI_ISL_1720907

EPI_ISL_1720912

EPI_ISL_2679749

EPI_ISL_2679753

EPI_ISL_2344023

EPI_ISL_1720923

EPI_ISL_1720938

EPI_ISL_1720948

EPI_ISL_2679776

EPI_ISL_1720966

EPI_ISL_1720967

EPI_ISL_5395633

EPI_ISL_1720972

EPI_ISL_1720973

EPI_ISL_1720980

EPI_ISL_1720981

EPI_ISL_1720982

EPI_ISL_1720983

EPI_ISL_1720987

EPI_ISL_1721531

EPI_ISL_1721536

EPI_ISL_1721539

EPI_ISL_1721540

EPI_ISL_1721542

EPI_ISL_1720996

EPI_ISL_1721000

EPI_ISL_1721006

EPI_ISL_1721545

EPI_ISL_1721012

EPI_ISL_1721013

EPI_ISL_1721016

EPI_ISL_1721018

EPI_ISL_1721019

EPI_ISL_1721022

EPI_ISL_1721024

EPI_ISL_1721027

EPI_ISL_1721029

EPI_ISL_1721030

EPI_ISL_1721031

EPI_ISL_1721033

EPI_ISL_1721034

EPI_ISL_1721037

EPI_ISL_1721048

EPI_ISL_1721060

EPI_ISL_1721550

EPI_ISL_1721066

EPI_ISL_1721068

EPI_ISL_1721069

EPI_ISL_1721070

EPI_ISL_1721075

EPI_ISL_1721079

EPI_ISL_1721082

EPI_ISL_1721083

EPI_ISL_1721088

EPI_ISL_1721095

EPI_ISL_1721097

EPI_ISL_1721098

EPI_ISL_1721100

EPI_ISL_1721104

EPI_ISL_1721108

EPI_ISL_1721111

EPI_ISL_1721113

EPI_ISL_1721118

EPI_ISL_1721120

EPI_ISL_1721122

EPI_ISL_1721124

EPI_ISL_1721128

EPI_ISL_1721130

EPI_ISL_1721131

EPI_ISL_1721137

EPI_ISL_1721144

EPI_ISL_1721150

EPI_ISL_1721152

EPI_ISL_1721159

EPI_ISL_1721163

EPI_ISL_1721164

EPI_ISL_1721180

EPI_ISL_1721181

EPI_ISL_1721182

EPI_ISL_1721192

EPI_ISL_1721194

EPI_ISL_1721196

EPI_ISL_1721203

EPI_ISL_1721208

EPI_ISL_1721212

EPI_ISL_1721217

EPI_ISL_1721553

EPI_ISL_1721219

EPI_ISL_1721220

EPI_ISL_1721222

EPI_ISL_1721227

EPI_ISL_1721228

EPI_ISL_1721231

EPI_ISL_1721232

EPI_ISL_1721236

EPI_ISL_1721240

EPI_ISL_1721245

EPI_ISL_1721247

EPI_ISL_1721249

EPI_ISL_1721252

EPI_ISL_1721255

EPI_ISL_1721262

EPI_ISL_1721555

EPI_ISL_1721269

EPI_ISL_2896995

EPI_ISL_1721270

EPI_ISL_1721277

EPI_ISL_1721280

EPI_ISL_1721283

EPI_ISL_1721563

EPI_ISL_1721291

EPI_ISL_1721302

EPI_ISL_1721304

EPI_ISL_1721564

EPI_ISL_1721306

EPI_ISL_1721311

EPI_ISL_1721313

EPI_ISL_1721314

EPI_ISL_1721315

EPI_ISL_1721316

EPI_ISL_1721318

EPI_ISL_1721322

EPI_ISL_1721324

EPI_ISL_1721339

EPI_ISL_1721341

EPI_ISL_1721347

EPI_ISL_1721358

EPI_ISL_1721366

EPI_ISL_1721367

EPI_ISL_1721371

EPI_ISL_1721379

EPI_ISL_1721400

EPI_ISL_1721405

EPI_ISL_1721411

EPI_ISL_1721415

EPI_ISL_1758583

EPI_ISL_1721421

EPI_ISL_2546486

EPI_ISL_1721423

EPI_ISL_1721427

EPI_ISL_1721429

EPI_ISL_1721430

EPI_ISL_1721431

EPI_ISL_1721435

EPI_ISL_1721436

EPI_ISL_1721440

EPI_ISL_1721442

EPI_ISL_1721443

EPI_ISL_1721447

EPI_ISL_1721567

EPI_ISL_1721449

EPI_ISL_1721453

EPI_ISL_1721457

EPI_ISL_1721464

EPI_ISL_1721465

EPI_ISL_1721467

EPI_ISL_1721473

EPI_ISL_1721476

EPI_ISL_1721479

EPI_ISL_1721481

EPI_ISL_1721570

EPI_ISL_1721571

EPI_ISL_1721572

EPI_ISL_1721484

EPI_ISL_1721485

EPI_ISL_1721491

EPI_ISL_1721492

EPI_ISL_1721593

EPI_ISL_1721594

EPI_ISL_1760547

EPI_ISL_6182027

EPI_ISL_1924419

EPI_ISL_1721605

EPI_ISL_1721608

EPI_ISL_1721609

EPI_ISL_1721937

EPI_ISL_1721614

EPI_ISL_1721616

EPI_ISL_1721621

EPI_ISL_1721623

EPI_ISL_1721624

EPI_ISL_1721631

EPI_ISL_1721633

EPI_ISL_2546575

EPI_ISL_1721636

EPI_ISL_1721640

EPI_ISL_1721645

EPI_ISL_1721646

EPI_ISL_1721651

EPI_ISL_1721653

EPI_ISL_1721654

EPI_ISL_1721659

EPI_ISL_1721661

EPI_ISL_1721674

EPI_ISL_1721677

EPI_ISL_1721680

EPI_ISL_1721693

EPI_ISL_1721700

EPI_ISL_1721701

EPI_ISL_1721702

EPI_ISL_1721704

EPI_ISL_1721708

EPI_ISL_1721709

EPI_ISL_1721711

EPI_ISL_1721713

EPI_ISL_1721716

EPI_ISL_1721717

EPI_ISL_1721718

EPI_ISL_1721722

EPI_ISL_1721723

EPI_ISL_1721726

EPI_ISL_1721732

EPI_ISL_1721736

EPI_ISL_1721737

EPI_ISL_1721738

EPI_ISL_1721739

EPI_ISL_1721740

EPI_ISL_1721744

EPI_ISL_1721746

EPI_ISL_1721748

EPI_ISL_1721749

EPI_ISL_1721750

EPI_ISL_1721752

EPI_ISL_1721753

EPI_ISL_1721760

EPI_ISL_1721761

EPI_ISL_1721763

EPI_ISL_1721765

EPI_ISL_1721773

EPI_ISL_1721777

EPI_ISL_1721778

EPI_ISL_1721779

EPI_ISL_1721780

EPI_ISL_1721781

EPI_ISL_1721784

EPI_ISL_1721785

EPI_ISL_1721788

EPI_ISL_1721789

EPI_ISL_1721792

EPI_ISL_1721793

EPI_ISL_1721794

EPI_ISL_1721796

EPI_ISL_1721797

EPI_ISL_1721798

EPI_ISL_1721800

EPI_ISL_1721801

EPI_ISL_1721803

EPI_ISL_1721804

EPI_ISL_1721807

EPI_ISL_1721809

EPI_ISL_1721938

EPI_ISL_1721939

EPI_ISL_1721812

EPI_ISL_1721813

EPI_ISL_1721815

EPI_ISL_1721817

EPI_ISL_1721819

EPI_ISL_1721822

EPI_ISL_1721824

EPI_ISL_1721826

EPI_ISL_1721829

EPI_ISL_1721833

EPI_ISL_1721835

EPI_ISL_1721836

EPI_ISL_1721837

EPI_ISL_1721839

EPI_ISL_1721840

EPI_ISL_1721842

EPI_ISL_1721843

EPI_ISL_1721845

EPI_ISL_1721846

EPI_ISL_1721847

EPI_ISL_1721848

EPI_ISL_1721851

EPI_ISL_1721854

EPI_ISL_1721858

EPI_ISL_1721859

EPI_ISL_1721860

EPI_ISL_1721861

EPI_ISL_1721862

EPI_ISL_1721864

EPI_ISL_1721867

EPI_ISL_1721872

EPI_ISL_1721873

EPI_ISL_1721875

EPI_ISL_1721876

EPI_ISL_1721881

EPI_ISL_1721884

EPI_ISL_1721887

EPI_ISL_1721892

EPI_ISL_1721897

EPI_ISL_1721898

EPI_ISL_1721901

EPI_ISL_1721905

EPI_ISL_1721906

EPI_ISL_1721911

EPI_ISL_1721913

EPI_ISL_1721914

EPI_ISL_1721916

EPI_ISL_1721919

EPI_ISL_1721946

EPI_ISL_1721920

EPI_ISL_1721921

EPI_ISL_1721922

EPI_ISL_1721923

EPI_ISL_1721924

EPI_ISL_1721925

EPI_ISL_1721929

EPI_ISL_2776431

EPI_ISL_1721930

EPI_ISL_1721931

EPI_ISL_1721932

EPI_ISL_1721933

EPI_ISL_1721936

EPI_ISL_1721948

EPI_ISL_1721949

EPI_ISL_1721951

EPI_ISL_1721952

EPI_ISL_1721953

EPI_ISL_1721955

EPI_ISL_1721957

EPI_ISL_1721958

EPI_ISL_3599468

EPI_ISL_1721959

EPI_ISL_1721962

EPI_ISL_1721963

EPI_ISL_1721964

EPI_ISL_1721965

EPI_ISL_1721967

EPI_ISL_1721970

EPI_ISL_1721971

EPI_ISL_1721973

EPI_ISL_1721974

EPI_ISL_1721975

EPI_ISL_1721976

EPI_ISL_1721977

EPI_ISL_1721978

EPI_ISL_1721980

EPI_ISL_1721983

EPI_ISL_1721984

EPI_ISL_1721985

EPI_ISL_1721986

EPI_ISL_1721988

EPI_ISL_1721989

EPI_ISL_3553691

EPI_ISL_1721990

EPI_ISL_1721991

EPI_ISL_1721992

EPI_ISL_1721993

EPI_ISL_1721994

EPI_ISL_1721996

EPI_ISL_1721997

EPI_ISL_1721998

EPI_ISL_1721999

EPI_ISL_1722004

EPI_ISL_1722011

EPI_ISL_1722013

EPI_ISL_1722018

EPI_ISL_1722021

EPI_ISL_1722025

EPI_ISL_1722026

EPI_ISL_1722027

EPI_ISL_1722029

EPI_ISL_1722030

EPI_ISL_1722031

EPI_ISL_1722032

EPI_ISL_1722036

EPI_ISL_1722039

EPI_ISL_1722043

EPI_ISL_1722045

EPI_ISL_1722049

EPI_ISL_1722051

EPI_ISL_1722054

EPI_ISL_1722055

EPI_ISL_1722060

EPI_ISL_1722061

EPI_ISL_1722090

EPI_ISL_1722091

EPI_ISL_1722093

EPI_ISL_1722094

EPI_ISL_1722096

EPI_ISL_1722099

EPI_ISL_1722103

EPI_ISL_1722108

EPI_ISL_1722111

EPI_ISL_1722112

EPI_ISL_1722118

EPI_ISL_1722122

EPI_ISL_1722132

EPI_ISL_1722145

EPI_ISL_1722167

EPI_ISL_1722173

EPI_ISL_1722184

EPI_ISL_1722185

EPI_ISL_1722189

EPI_ISL_1722190

EPI_ISL_1722194

EPI_ISL_1722196

EPI_ISL_1722216

EPI_ISL_1722218

EPI_ISL_1722220

EPI_ISL_3599622

EPI_ISL_1722231

EPI_ISL_1722252

EPI_ISL_1722258

EPI_ISL_1722260

EPI_ISL_1722267

EPI_ISL_1722277

EPI_ISL_1722279

EPI_ISL_1759567

EPI_ISL_1722281

EPI_ISL_1722288

EPI_ISL_1722290

EPI_ISL_1722291

EPI_ISL_1722292

EPI_ISL_1722293

EPI_ISL_1722298

EPI_ISL_1722302

EPI_ISL_1722310

EPI_ISL_1722315

EPI_ISL_1722319

EPI_ISL_1722320

EPI_ISL_1722323

EPI_ISL_1722328

EPI_ISL_1722330

EPI_ISL_1722331

EPI_ISL_1722332

EPI_ISL_1722333

EPI_ISL_1723099

EPI_ISL_1722343

EPI_ISL_1722345

EPI_ISL_1722346

EPI_ISL_1722348

EPI_ISL_1722354

EPI_ISL_1722374

EPI_ISL_1722381

EPI_ISL_1722389

EPI_ISL_1722394

EPI_ISL_1722395

EPI_ISL_1722403

EPI_ISL_1722405

EPI_ISL_1722409

EPI_ISL_1722410

EPI_ISL_1722413

EPI_ISL_1722414

EPI_ISL_1722421

EPI_ISL_1723102

EPI_ISL_1722428

EPI_ISL_1722429

EPI_ISL_1722430

EPI_ISL_1722431

EPI_ISL_1722434

EPI_ISL_1722436

EPI_ISL_1722437

EPI_ISL_1722439

EPI_ISL_1722440

EPI_ISL_1722441

EPI_ISL_1722443

EPI_ISL_1722445

EPI_ISL_1722447

EPI_ISL_1723103

EPI_ISL_1722449

EPI_ISL_1722450

EPI_ISL_1722451

EPI_ISL_1723104

EPI_ISL_1722454

EPI_ISL_1722455

EPI_ISL_1722456

EPI_ISL_1722457

EPI_ISL_1722458

EPI_ISL_1722461

EPI_ISL_1722464

EPI_ISL_1722465

EPI_ISL_1722470

EPI_ISL_1722471

EPI_ISL_1722472

EPI_ISL_1722474

EPI_ISL_1722475

EPI_ISL_1722477

EPI_ISL_1722478

EPI_ISL_1722480

EPI_ISL_1722481

EPI_ISL_1722483

EPI_ISL_1722484

EPI_ISL_1722485

EPI_ISL_1722486

EPI_ISL_1722487

EPI_ISL_1722488

EPI_ISL_1722489

EPI_ISL_1722490

EPI_ISL_1722492

EPI_ISL_1722493

EPI_ISL_1722494

EPI_ISL_1722497

EPI_ISL_1722500

EPI_ISL_1722501

EPI_ISL_1722502

EPI_ISL_1722503

EPI_ISL_1722506

EPI_ISL_1722507

EPI_ISL_1722508

EPI_ISL_1759694

EPI_ISL_1722513

EPI_ISL_1722514

EPI_ISL_1722517

EPI_ISL_1722519

EPI_ISL_1722520

EPI_ISL_1722522

EPI_ISL_1722524

EPI_ISL_1722525

EPI_ISL_1722527

EPI_ISL_1722528

EPI_ISL_1722529

EPI_ISL_1722531

EPI_ISL_1722532

EPI_ISL_1722533

EPI_ISL_1722534

EPI_ISL_1722536

EPI_ISL_1722538

EPI_ISL_1722539

EPI_ISL_1722540

EPI_ISL_1722541

EPI_ISL_1722542

EPI_ISL_1722544

EPI_ISL_1723107

EPI_ISL_1723110

EPI_ISL_1723111

EPI_ISL_1722547

EPI_ISL_1722551

EPI_ISL_1722552

EPI_ISL_1722555

EPI_ISL_1722556

EPI_ISL_1722560

EPI_ISL_1722561

EPI_ISL_1722562

EPI_ISL_1722564

EPI_ISL_1722565

EPI_ISL_1722566

EPI_ISL_1722567

EPI_ISL_1722568

EPI_ISL_1722569

EPI_ISL_1722571

EPI_ISL_1722573

EPI_ISL_1722574

EPI_ISL_1722575

EPI_ISL_1722576

EPI_ISL_1722577

EPI_ISL_1722578

EPI_ISL_1722580

EPI_ISL_1722582

EPI_ISL_1722584

EPI_ISL_1723112

EPI_ISL_1722588

EPI_ISL_1722590

EPI_ISL_1722591

EPI_ISL_1722592

EPI_ISL_1722593

EPI_ISL_1722594

EPI_ISL_1722595

EPI_ISL_1722597

EPI_ISL_1722598

EPI_ISL_1722600

EPI_ISL_1722601

EPI_ISL_1722602

EPI_ISL_1722603

EPI_ISL_1722604

EPI_ISL_1722606

EPI_ISL_1722608

EPI_ISL_1722610

EPI_ISL_1723114

EPI_ISL_1722612

EPI_ISL_2546902

EPI_ISL_2546903

EPI_ISL_2546904

EPI_ISL_1759521

EPI_ISL_1722613

EPI_ISL_1722614

EPI_ISL_1722616

EPI_ISL_1722617

EPI_ISL_1722620

EPI_ISL_2546907

EPI_ISL_1722621

EPI_ISL_2546909

EPI_ISL_2546910

EPI_ISL_1722622

EPI_ISL_2546911

EPI_ISL_2546912

EPI_ISL_2546913

EPI_ISL_2546914

EPI_ISL_3599525

EPI_ISL_1722623

EPI_ISL_1722626

EPI_ISL_1722627

EPI_ISL_1722628

EPI_ISL_2546918

EPI_ISL_2546919

EPI_ISL_3599141

EPI_ISL_1722629

EPI_ISL_1722630

EPI_ISL_1722631

EPI_ISL_1722632

EPI_ISL_1723117

EPI_ISL_1722635

EPI_ISL_1722640

EPI_ISL_1722642

EPI_ISL_1722643

EPI_ISL_1722644

EPI_ISL_1722648

EPI_ISL_1722649

EPI_ISL_1722651

EPI_ISL_1722652

EPI_ISL_1722655

EPI_ISL_1722656

EPI_ISL_2546925

EPI_ISL_2546926

EPI_ISL_1722659

EPI_ISL_1722660

EPI_ISL_1722662

EPI_ISL_3599643

EPI_ISL_1722663

EPI_ISL_1722664

EPI_ISL_2546927

EPI_ISL_2546928

EPI_ISL_2546929

EPI_ISL_1722665

EPI_ISL_1722666

EPI_ISL_1759549

EPI_ISL_1722670

EPI_ISL_1722671

EPI_ISL_2546932

EPI_ISL_2546933

EPI_ISL_2546934

EPI_ISL_2546935

EPI_ISL_2546936

EPI_ISL_2546939

EPI_ISL_2546940

EPI_ISL_2546941

EPI_ISL_2546942

EPI_ISL_2546943

EPI_ISL_2546948

EPI_ISL_3599559

EPI_ISL_2546951

EPI_ISL_2546952

EPI_ISL_2546953

EPI_ISL_2546954

EPI_ISL_2546956

EPI_ISL_2546957

EPI_ISL_2546958

EPI_ISL_2546959

EPI_ISL_2546960

EPI_ISL_2546961

EPI_ISL_2546962

EPI_ISL_2546963

EPI_ISL_2546964

EPI_ISL_2546965

EPI_ISL_2546966

EPI_ISL_2546967

EPI_ISL_2546968

EPI_ISL_2546969

EPI_ISL_2546970

EPI_ISL_2546971

EPI_ISL_2546972

EPI_ISL_2546973

EPI_ISL_2546974

EPI_ISL_2546975

EPI_ISL_2546976

EPI_ISL_2546978

EPI_ISL_1723121

EPI_ISL_3599554

EPI_ISL_2546979

EPI_ISL_2546982

EPI_ISL_2546985

EPI_ISL_2546986

EPI_ISL_1723122

EPI_ISL_1723123

EPI_ISL_1722830

EPI_ISL_1722835

EPI_ISL_1722840

EPI_ISL_1722841

EPI_ISL_1723125

EPI_ISL_2546988

EPI_ISL_2546989

EPI_ISL_1722853

EPI_ISL_2546990

EPI_ISL_1723126

EPI_ISL_1722924

EPI_ISL_1722925

EPI_ISL_1722926

EPI_ISL_1722927

EPI_ISL_1722928

EPI_ISL_1722929

EPI_ISL_1722933

EPI_ISL_1722934

EPI_ISL_1722935

EPI_ISL_1722940

EPI_ISL_1722944

EPI_ISL_1722947

EPI_ISL_1722948

EPI_ISL_1722952

EPI_ISL_1722953

EPI_ISL_1722954

EPI_ISL_1722955

EPI_ISL_1722956

EPI_ISL_2546991

EPI_ISL_1722957

EPI_ISL_1722958

EPI_ISL_1722959

EPI_ISL_1722961

EPI_ISL_1968790

EPI_ISL_2546992

EPI_ISL_1722962

EPI_ISL_1722973

EPI_ISL_1722975

EPI_ISL_1722976

EPI_ISL_1722978

EPI_ISL_1722979

EPI_ISL_1722981

EPI_ISL_1722982

EPI_ISL_1722983

EPI_ISL_1722984

EPI_ISL_1722985

EPI_ISL_1722989

EPI_ISL_1722990

EPI_ISL_1722991

EPI_ISL_1722992

EPI_ISL_1722995

EPI_ISL_1722997

EPI_ISL_1722999

EPI_ISL_2546993

EPI_ISL_1723000

EPI_ISL_1723001

EPI_ISL_1723003

EPI_ISL_1723004

EPI_ISL_1723006

EPI_ISL_1723007

EPI_ISL_1723008

EPI_ISL_1723012

EPI_ISL_1723013

EPI_ISL_1723015

EPI_ISL_1723016

EPI_ISL_1723020

EPI_ISL_1723021

EPI_ISL_1723023

EPI_ISL_1723024

EPI_ISL_1723127

EPI_ISL_2546994

EPI_ISL_2546996

EPI_ISL_2546998

EPI_ISL_3599541

EPI_ISL_3599782

EPI_ISL_2546999

EPI_ISL_2547001

EPI_ISL_2547003

EPI_ISL_1723026

EPI_ISL_1723028

EPI_ISL_1723033

EPI_ISL_2547005

EPI_ISL_2547007

EPI_ISL_1723039

EPI_ISL_1723043

EPI_ISL_2547008

EPI_ISL_1723047

EPI_ISL_2547009

EPI_ISL_1723050

EPI_ISL_1723051

EPI_ISL_1723056

EPI_ISL_1723057

EPI_ISL_1723058

EPI_ISL_1723060

EPI_ISL_1723062

EPI_ISL_1723063

EPI_ISL_1723065

EPI_ISL_1723067

EPI_ISL_1723070

EPI_ISL_1723071

EPI_ISL_1723072

EPI_ISL_2547010

EPI_ISL_1723073

EPI_ISL_1723074

EPI_ISL_1723076

EPI_ISL_1723078

EPI_ISL_1723080

EPI_ISL_1723081

EPI_ISL_1723082

EPI_ISL_2547011

EPI_ISL_1723083

EPI_ISL_1723084

EPI_ISL_1723085

EPI_ISL_1723086

EPI_ISL_1723087

EPI_ISL_1723088

EPI_ISL_1723089

EPI_ISL_1723091

EPI_ISL_1723092

EPI_ISL_2547012

EPI_ISL_2547013

EPI_ISL_2547015

EPI_ISL_1723093

EPI_ISL_1723094

EPI_ISL_1723095

EPI_ISL_2547016

EPI_ISL_1723096

EPI_ISL_1723097

EPI_ISL_2547020

EPI_ISL_2547021

EPI_ISL_2547023

EPI_ISL_2547025

EPI_ISL_2547026

EPI_ISL_2547027

EPI_ISL_2547028

EPI_ISL_2547030

EPI_ISL_2547031

EPI_ISL_2547032

EPI_ISL_2547033

EPI_ISL_2547034

EPI_ISL_2547035

EPI_ISL_2547036

EPI_ISL_2547037

EPI_ISL_2547039

EPI_ISL_2547040

EPI_ISL_2547041

EPI_ISL_2547042

EPI_ISL_2547043

EPI_ISL_2547044

EPI_ISL_2547045

EPI_ISL_2547046

EPI_ISL_2547047

EPI_ISL_2547048

EPI_ISL_2547049

EPI_ISL_1723653

EPI_ISL_2547051

EPI_ISL_2547052

EPI_ISL_2547055

EPI_ISL_1723654

EPI_ISL_1723655

EPI_ISL_1723656

EPI_ISL_2547056

EPI_ISL_1723657

EPI_ISL_1723658

EPI_ISL_1723659

EPI_ISL_1723131

EPI_ISL_1723132

EPI_ISL_1723133

EPI_ISL_1723135

EPI_ISL_1723660

EPI_ISL_1723661

EPI_ISL_1723136

EPI_ISL_1723137

EPI_ISL_1723662

EPI_ISL_1723138

EPI_ISL_1723140

EPI_ISL_1723141

EPI_ISL_1723151

EPI_ISL_1723157

EPI_ISL_1723158

EPI_ISL_1723663

EPI_ISL_1723664

EPI_ISL_1723665

EPI_ISL_1723173

EPI_ISL_1723174

EPI_ISL_1723176

EPI_ISL_1723179

EPI_ISL_1723180

EPI_ISL_1723181

EPI_ISL_1723182

EPI_ISL_1723183

EPI_ISL_1723185

EPI_ISL_1723186

EPI_ISL_1723187

EPI_ISL_1723188

EPI_ISL_1723190

EPI_ISL_1723192

EPI_ISL_1723193

EPI_ISL_1723194

EPI_ISL_1723195

EPI_ISL_1723196

EPI_ISL_1723198

EPI_ISL_1584969

EPI_ISL_1723200

EPI_ISL_1723201

EPI_ISL_1723202

EPI_ISL_1723203

EPI_ISL_1723204

EPI_ISL_1723205

EPI_ISL_1723207

EPI_ISL_1723209

EPI_ISL_1723211

EPI_ISL_1723212

EPI_ISL_1723213

EPI_ISL_1723215

EPI_ISL_1723216

EPI_ISL_1723217

EPI_ISL_1723218

EPI_ISL_1723219

EPI_ISL_1723221

EPI_ISL_1723222

EPI_ISL_1723224

EPI_ISL_1723228

EPI_ISL_1723229

EPI_ISL_1723231

EPI_ISL_1723232

EPI_ISL_1723233

EPI_ISL_1723235

EPI_ISL_1723236

EPI_ISL_1723237

EPI_ISL_1723238

EPI_ISL_1723239

EPI_ISL_1723240

EPI_ISL_1723242

EPI_ISL_1723243

EPI_ISL_1723244

EPI_ISL_1723246

EPI_ISL_1723247

EPI_ISL_1723250

EPI_ISL_1723252

EPI_ISL_1723254

EPI_ISL_1723256

EPI_ISL_1723257

EPI_ISL_1723258

EPI_ISL_1723259

EPI_ISL_1723262

EPI_ISL_1723263

EPI_ISL_1723264

EPI_ISL_1723265

EPI_ISL_1723266

EPI_ISL_1723268

EPI_ISL_1723281

EPI_ISL_1723282

EPI_ISL_1723284

EPI_ISL_1723286

EPI_ISL_1723290

EPI_ISL_1723293

EPI_ISL_1723300

EPI_ISL_1723302

EPI_ISL_1723303

EPI_ISL_1723304

EPI_ISL_1723305

EPI_ISL_1723306

EPI_ISL_1723307

EPI_ISL_1723308

EPI_ISL_1723309

EPI_ISL_1723311

EPI_ISL_1723313

EPI_ISL_1723314

EPI_ISL_1723315

EPI_ISL_1723316

EPI_ISL_1723317

EPI_ISL_1723320

EPI_ISL_1723321

EPI_ISL_1723322

EPI_ISL_1723323

EPI_ISL_1723324

EPI_ISL_1723326

EPI_ISL_1723327

EPI_ISL_1723328

EPI_ISL_1723331

EPI_ISL_1723332

EPI_ISL_1723333

EPI_ISL_1723334

EPI_ISL_1723335

EPI_ISL_1723336

EPI_ISL_1723338

EPI_ISL_1723339

EPI_ISL_1723340

EPI_ISL_1723341

EPI_ISL_1723342

EPI_ISL_1723344

EPI_ISL_1723345

EPI_ISL_1723350

EPI_ISL_1584973

EPI_ISL_1723351

EPI_ISL_1723353

EPI_ISL_1723354

EPI_ISL_1723355

EPI_ISL_1723356

EPI_ISL_1723358

EPI_ISL_1723359

EPI_ISL_1723360

EPI_ISL_1723361

EPI_ISL_1723362

EPI_ISL_1723363

EPI_ISL_1723364

EPI_ISL_1723365

EPI_ISL_1723366

EPI_ISL_1723368

EPI_ISL_1723369

EPI_ISL_1723370

EPI_ISL_1723371

EPI_ISL_1723372

EPI_ISL_1723374

EPI_ISL_1723375

EPI_ISL_1723376

EPI_ISL_1723377

EPI_ISL_1723378

EPI_ISL_1723379

EPI_ISL_1723381

EPI_ISL_1723386

EPI_ISL_1723388

EPI_ISL_1723393

EPI_ISL_1723395

EPI_ISL_1723400

EPI_ISL_1723401

EPI_ISL_1723403

EPI_ISL_1723406

EPI_ISL_1723667

EPI_ISL_1723668

EPI_ISL_1723408

EPI_ISL_1723669

EPI_ISL_1723409

EPI_ISL_1723411

EPI_ISL_1723412

EPI_ISL_1723413

EPI_ISL_1723670

EPI_ISL_1723414

EPI_ISL_1723416

EPI_ISL_1723417

EPI_ISL_1723426

EPI_ISL_1723671

EPI_ISL_1723672

EPI_ISL_1723428

EPI_ISL_1723430

EPI_ISL_1723433

EPI_ISL_1723435

EPI_ISL_1723673

EPI_ISL_1723674

EPI_ISL_1723675

EPI_ISL_1723676

EPI_ISL_1723437

EPI_ISL_1723438

EPI_ISL_1723679

EPI_ISL_5404734

EPI_ISL_5404754

EPI_ISL_1723440

EPI_ISL_1723441

EPI_ISL_1723442

EPI_ISL_1723443

EPI_ISL_1723444

EPI_ISL_1723445

EPI_ISL_1723446

EPI_ISL_1723448

EPI_ISL_1723449

EPI_ISL_1723450

EPI_ISL_1723451

EPI_ISL_1723452

EPI_ISL_1723453

EPI_ISL_1723454

EPI_ISL_1723455

EPI_ISL_5404913

EPI_ISL_5404924

EPI_ISL_1723457

EPI_ISL_3599605

EPI_ISL_1723459

EPI_ISL_1723460

EPI_ISL_1723461

EPI_ISL_1723462

EPI_ISL_1723463

EPI_ISL_1723464

EPI_ISL_1723466

EPI_ISL_1723467

EPI_ISL_1723468

EPI_ISL_1723471

EPI_ISL_1723472

EPI_ISL_1723473

EPI_ISL_1723474

EPI_ISL_1723475

EPI_ISL_1723476

EPI_ISL_1723478

EPI_ISL_1723479

EPI_ISL_1723480

EPI_ISL_1723481

EPI_ISL_1723483

EPI_ISL_1723484

EPI_ISL_1723485

EPI_ISL_1723486

EPI_ISL_1723487

EPI_ISL_1723488

EPI_ISL_1723489

EPI_ISL_1723490

EPI_ISL_1723491

EPI_ISL_1723492

EPI_ISL_1723493

EPI_ISL_1723494

EPI_ISL_1723495

EPI_ISL_1723496

EPI_ISL_1723497

EPI_ISL_1723498

EPI_ISL_1723499

EPI_ISL_1723501

EPI_ISL_1723502

EPI_ISL_1723503

EPI_ISL_1723505

EPI_ISL_1723506

EPI_ISL_1723507

EPI_ISL_1723508

EPI_ISL_1723511

EPI_ISL_1723512

EPI_ISL_1723513

EPI_ISL_1723514

EPI_ISL_1723515

EPI_ISL_1723517

EPI_ISL_1723518

EPI_ISL_1723520

EPI_ISL_1723522

EPI_ISL_1723523

EPI_ISL_1723525

EPI_ISL_1723526

EPI_ISL_1723527

EPI_ISL_1723528

EPI_ISL_1723530

EPI_ISL_1723531

EPI_ISL_1723532

EPI_ISL_1723533

EPI_ISL_1723534

EPI_ISL_1723535

EPI_ISL_1723536

EPI_ISL_1723537

EPI_ISL_1723538

EPI_ISL_1723539

EPI_ISL_1723541

EPI_ISL_1723542

EPI_ISL_1723543

EPI_ISL_1723544

EPI_ISL_1723545

EPI_ISL_1723546

EPI_ISL_1723547

EPI_ISL_1723548

EPI_ISL_1723549

EPI_ISL_1723550

EPI_ISL_1723551

EPI_ISL_1723552

EPI_ISL_1723553

EPI_ISL_1723556

EPI_ISL_1723557

EPI_ISL_1723558

EPI_ISL_1723560

EPI_ISL_1723561

EPI_ISL_1723563

EPI_ISL_1723564

EPI_ISL_1723565

EPI_ISL_1723566

EPI_ISL_1723567

EPI_ISL_1723568

EPI_ISL_1723569

EPI_ISL_1723570

EPI_ISL_1723571

EPI_ISL_1723572

EPI_ISL_1723573

EPI_ISL_1723574

EPI_ISL_1723575

EPI_ISL_1723576

EPI_ISL_1723577

EPI_ISL_1723578

EPI_ISL_1723579

EPI_ISL_1723580

EPI_ISL_1723582

EPI_ISL_1723583

EPI_ISL_1723584

EPI_ISL_1723585

EPI_ISL_1723586

EPI_ISL_1723587

EPI_ISL_1723588

EPI_ISL_1723589

EPI_ISL_1723590

EPI_ISL_1723591

EPI_ISL_1723592

EPI_ISL_1723594

EPI_ISL_1723595

EPI_ISL_1723596

EPI_ISL_1723597

EPI_ISL_1723598

EPI_ISL_1723599

EPI_ISL_1723600

EPI_ISL_1723601

EPI_ISL_1723602

EPI_ISL_1723603

EPI_ISL_1723605

EPI_ISL_1723606

EPI_ISL_1723607

EPI_ISL_1723609

EPI_ISL_1723611

EPI_ISL_1723612

EPI_ISL_1723613

EPI_ISL_1723615

EPI_ISL_1723616

EPI_ISL_1723617

EPI_ISL_1723618

EPI_ISL_1723619

EPI_ISL_1723620

EPI_ISL_1723621

EPI_ISL_1723622

EPI_ISL_1723623

EPI_ISL_1723624

EPI_ISL_1723626

EPI_ISL_1723627

EPI_ISL_1723628

EPI_ISL_1723629

EPI_ISL_1723630

EPI_ISL_1723631

EPI_ISL_1723632

EPI_ISL_1723634

EPI_ISL_1723635

EPI_ISL_1723637

EPI_ISL_1723638

EPI_ISL_1723639

EPI_ISL_1723641

EPI_ISL_1723642

EPI_ISL_1723643

EPI_ISL_1723644

EPI_ISL_1723645

EPI_ISL_1723646

EPI_ISL_1723647

EPI_ISL_1723648

EPI_ISL_1723650

EPI_ISL_1723651

EPI_ISL_1723652

EPI_ISL_1723682

EPI_ISL_1723683

EPI_ISL_1723684

EPI_ISL_1724653

EPI_ISL_1723687

EPI_ISL_1723688

EPI_ISL_1723689

EPI_ISL_1723690

EPI_ISL_1723691

EPI_ISL_1723692

EPI_ISL_1723693

EPI_ISL_1723694

EPI_ISL_1723695

EPI_ISL_1723696

EPI_ISL_1723697

EPI_ISL_1723698

EPI_ISL_1723699

EPI_ISL_1723700

EPI_ISL_1723701

EPI_ISL_1723702

EPI_ISL_1723703

EPI_ISL_1723704

EPI_ISL_1723705

EPI_ISL_1723706

EPI_ISL_1723707

EPI_ISL_1723708

EPI_ISL_1724655

EPI_ISL_1723709

EPI_ISL_1723711

EPI_ISL_1723712

EPI_ISL_1723713

EPI_ISL_1723714

EPI_ISL_1723715

EPI_ISL_1723716

EPI_ISL_1723717

EPI_ISL_1723718

EPI_ISL_1723719

EPI_ISL_1723720

EPI_ISL_1723721

EPI_ISL_1723722

EPI_ISL_1723723

EPI_ISL_1723724

EPI_ISL_1723725

EPI_ISL_1723726

EPI_ISL_1723727

EPI_ISL_1723728

EPI_ISL_1723729

EPI_ISL_1723730

EPI_ISL_1723731

EPI_ISL_1723732

EPI_ISL_1724658

EPI_ISL_1723733

EPI_ISL_1723735

EPI_ISL_1723737

EPI_ISL_1723738

EPI_ISL_1724659

EPI_ISL_1723740

EPI_ISL_1723741

EPI_ISL_1723742

EPI_ISL_1723743

EPI_ISL_1724660

EPI_ISL_1723745

EPI_ISL_1723746

EPI_ISL_1723747

EPI_ISL_1723750

EPI_ISL_1723751

EPI_ISL_1723752

EPI_ISL_1723754

EPI_ISL_1723755

EPI_ISL_1723756

EPI_ISL_1723757

EPI_ISL_1723758

EPI_ISL_1723759

EPI_ISL_1723761

EPI_ISL_1723762

EPI_ISL_1723763

EPI_ISL_1723764

EPI_ISL_1723765

EPI_ISL_1723766

EPI_ISL_1723767

EPI_ISL_1723768

EPI_ISL_1723769

EPI_ISL_1723770

EPI_ISL_1723771

EPI_ISL_1723772

EPI_ISL_1723773

EPI_ISL_1723774

EPI_ISL_1723775

EPI_ISL_1723776

EPI_ISL_1723778

EPI_ISL_1723779

EPI_ISL_1723780

EPI_ISL_1723781

EPI_ISL_1723782

EPI_ISL_1723783

EPI_ISL_1723785

EPI_ISL_1723786

EPI_ISL_1723787

EPI_ISL_1723789

EPI_ISL_1723790

EPI_ISL_1723791

EPI_ISL_1724662

EPI_ISL_1724663

EPI_ISL_1724664

EPI_ISL_1724665

EPI_ISL_1724666

EPI_ISL_1724668

EPI_ISL_1724669

EPI_ISL_1724670

EPI_ISL_1724672

EPI_ISL_1724674

EPI_ISL_1724675

EPI_ISL_1723793

EPI_ISL_1723794

EPI_ISL_1723795

EPI_ISL_1723796

EPI_ISL_1724677

EPI_ISL_1723798

EPI_ISL_1723799

EPI_ISL_1723801

EPI_ISL_1723802

EPI_ISL_1723806

EPI_ISL_1723807

EPI_ISL_1723808

EPI_ISL_1723809

EPI_ISL_1723810

EPI_ISL_1723811

EPI_ISL_1723813

EPI_ISL_1723814

EPI_ISL_1723815

EPI_ISL_1723818

EPI_ISL_1723819

EPI_ISL_1724678

EPI_ISL_1723822

EPI_ISL_1723823

EPI_ISL_1723824

EPI_ISL_1723826

EPI_ISL_1723827

EPI_ISL_1723828

EPI_ISL_1723830

EPI_ISL_1723831

EPI_ISL_1723832

EPI_ISL_1723833

EPI_ISL_1723835

EPI_ISL_1723837

EPI_ISL_1723838

EPI_ISL_1723839

EPI_ISL_1723840

EPI_ISL_1723841

EPI_ISL_1723842

EPI_ISL_1724679

EPI_ISL_1723845

EPI_ISL_1723846

EPI_ISL_2172283

EPI_ISL_1723847

EPI_ISL_1723848

EPI_ISL_1723849

EPI_ISL_1723850

EPI_ISL_1723851

EPI_ISL_1723852

EPI_ISL_1723853

EPI_ISL_1723854

EPI_ISL_1723855

EPI_ISL_1723856

EPI_ISL_1723858

EPI_ISL_1723860

EPI_ISL_1723861

EPI_ISL_1723862

EPI_ISL_1723864

EPI_ISL_1723866

EPI_ISL_1723867

EPI_ISL_1723868

EPI_ISL_1723869

EPI_ISL_1723870

EPI_ISL_1723871

EPI_ISL_1723873

EPI_ISL_1723874

EPI_ISL_1723875

EPI_ISL_1723877

EPI_ISL_1723878

EPI_ISL_1723879

EPI_ISL_1723880

EPI_ISL_1723881

EPI_ISL_1723882

EPI_ISL_1724680

EPI_ISL_1723885

EPI_ISL_1723886

EPI_ISL_1723887

EPI_ISL_1723888

EPI_ISL_1723889

EPI_ISL_1723890

EPI_ISL_1723891

EPI_ISL_1723892

EPI_ISL_1723893

EPI_ISL_1723894

EPI_ISL_1723895

EPI_ISL_1723896

EPI_ISL_1723897

EPI_ISL_1724681

EPI_ISL_1724682

EPI_ISL_1723900

EPI_ISL_1723901

EPI_ISL_1723902

EPI_ISL_1723903

EPI_ISL_1731712

EPI_ISL_1723904

EPI_ISL_1723905

EPI_ISL_1723906

EPI_ISL_1723907

EPI_ISL_1723908

EPI_ISL_1723909

EPI_ISL_1723912

EPI_ISL_1723913

EPI_ISL_1723914

EPI_ISL_1723915

EPI_ISL_1723916

EPI_ISL_1723917

EPI_ISL_1723918

EPI_ISL_1723919

EPI_ISL_1724683

EPI_ISL_1723921

EPI_ISL_1723923

EPI_ISL_1723924

EPI_ISL_1723925

EPI_ISL_1723926

EPI_ISL_1723927

EPI_ISL_1723928

EPI_ISL_1723929

EPI_ISL_1723930

EPI_ISL_1723931

EPI_ISL_1723933

EPI_ISL_1723934

EPI_ISL_1723935

EPI_ISL_1723936

EPI_ISL_1723937

EPI_ISL_1723938

EPI_ISL_1723939

EPI_ISL_1723940

EPI_ISL_1723941

EPI_ISL_1723942

EPI_ISL_1723943

EPI_ISL_1723944

EPI_ISL_1723945

EPI_ISL_1723946

EPI_ISL_1723947

EPI_ISL_1723948

EPI_ISL_1723949

EPI_ISL_1723950

EPI_ISL_1723952

EPI_ISL_1723953

EPI_ISL_1723954

EPI_ISL_1723955

EPI_ISL_1723956

EPI_ISL_1723957

EPI_ISL_1723958

EPI_ISL_1723959

EPI_ISL_1723960

EPI_ISL_1723961

EPI_ISL_1723964

EPI_ISL_1723965

EPI_ISL_1723967

EPI_ISL_1724684

EPI_ISL_1724686

EPI_ISL_1724687

EPI_ISL_1724688

EPI_ISL_1724689

EPI_ISL_1724690

EPI_ISL_1724692

EPI_ISL_1723969

EPI_ISL_1723970

EPI_ISL_1723971

EPI_ISL_1723972

EPI_ISL_1723973

EPI_ISL_1723974

EPI_ISL_1723977

EPI_ISL_1723978

EPI_ISL_1723979

EPI_ISL_1723980

EPI_ISL_1936797

EPI_ISL_1723982

EPI_ISL_1723983

EPI_ISL_1723984

EPI_ISL_1723985

EPI_ISL_1723986

EPI_ISL_1723987

EPI_ISL_1723988

EPI_ISL_1723989

EPI_ISL_1723990

EPI_ISL_1723991

EPI_ISL_1723993

EPI_ISL_2786702

EPI_ISL_2786730

EPI_ISL_1723994

EPI_ISL_1723995

EPI_ISL_1723996

EPI_ISL_1723997

EPI_ISL_1724694

EPI_ISL_1724695

EPI_ISL_1724696

EPI_ISL_1724000

EPI_ISL_1724001

EPI_ISL_1724002

EPI_ISL_1724003

EPI_ISL_1724004

EPI_ISL_1724005

EPI_ISL_1724007

EPI_ISL_1724008

EPI_ISL_1724009

EPI_ISL_1724010

EPI_ISL_1724012

EPI_ISL_1724013

EPI_ISL_1724014

EPI_ISL_1724015

EPI_ISL_1724016

EPI_ISL_1724017

EPI_ISL_1724018

EPI_ISL_1724019

EPI_ISL_1724020

EPI_ISL_1724022

EPI_ISL_1724023

EPI_ISL_1724024

EPI_ISL_1724025

EPI_ISL_1724027

EPI_ISL_1724028

EPI_ISL_1724030

EPI_ISL_1724031

EPI_ISL_1724032

EPI_ISL_1724033

EPI_ISL_1936833

EPI_ISL_1724035

EPI_ISL_1724036

EPI_ISL_1724037

EPI_ISL_1724039

EPI_ISL_1724040

EPI_ISL_1724043

EPI_ISL_1724044

EPI_ISL_1724045

EPI_ISL_1724046

EPI_ISL_1724049

EPI_ISL_1724051

EPI_ISL_1724052

EPI_ISL_1724053

EPI_ISL_1724054

EPI_ISL_1724055

EPI_ISL_1724056

EPI_ISL_1724057

EPI_ISL_1724058

EPI_ISL_1724061

EPI_ISL_1724062

EPI_ISL_1724063

EPI_ISL_1724065

EPI_ISL_1724067

EPI_ISL_1724068

EPI_ISL_1724069

EPI_ISL_1724070

EPI_ISL_1724071

EPI_ISL_1724073

EPI_ISL_1724076

EPI_ISL_1724079

EPI_ISL_1724080

EPI_ISL_1724081

EPI_ISL_1724083

EPI_ISL_1724085

EPI_ISL_1724086

EPI_ISL_1724087

EPI_ISL_1724089

EPI_ISL_1724091

EPI_ISL_1724092

EPI_ISL_1724094

EPI_ISL_1724095

EPI_ISL_1724096

EPI_ISL_1724097

EPI_ISL_1724098

EPI_ISL_1724099

EPI_ISL_1724100

EPI_ISL_1724101

EPI_ISL_1724102

EPI_ISL_1724103

EPI_ISL_1724105

EPI_ISL_1724106

EPI_ISL_1724107

EPI_ISL_1724109

EPI_ISL_1724110

EPI_ISL_1724111

EPI_ISL_1724112

EPI_ISL_1724113

EPI_ISL_1724114

EPI_ISL_1724116

EPI_ISL_1724118

EPI_ISL_1724121

EPI_ISL_1724123

EPI_ISL_1724124

EPI_ISL_1724126

EPI_ISL_1724127

EPI_ISL_1724128

EPI_ISL_1724129

EPI_ISL_1724130

EPI_ISL_1724131

EPI_ISL_1724132

EPI_ISL_1724134

EPI_ISL_1724135

EPI_ISL_1724136

EPI_ISL_1724137

EPI_ISL_1724139

EPI_ISL_1724140

EPI_ISL_1724141

EPI_ISL_1724142

EPI_ISL_1724144

EPI_ISL_1724145

EPI_ISL_1724146

EPI_ISL_1724148

EPI_ISL_1724149

EPI_ISL_1724150

EPI_ISL_1724152

EPI_ISL_1724154

EPI_ISL_1724157

EPI_ISL_1724158

EPI_ISL_1724159

EPI_ISL_1724160

EPI_ISL_1724162

EPI_ISL_1724163

EPI_ISL_1724165

EPI_ISL_1724166

EPI_ISL_1724167

EPI_ISL_1724169

EPI_ISL_1724170

EPI_ISL_1724171

EPI_ISL_1724174

EPI_ISL_2547436

EPI_ISL_1724175

EPI_ISL_1724176

EPI_ISL_1724177

EPI_ISL_1724179

EPI_ISL_1724180

EPI_ISL_1724181

EPI_ISL_1724184

EPI_ISL_1724185

EPI_ISL_2547446

EPI_ISL_1724186

EPI_ISL_1724187

EPI_ISL_1724188

EPI_ISL_1724189

EPI_ISL_1724190

EPI_ISL_1724192

EPI_ISL_1724193

EPI_ISL_1724195

EPI_ISL_1724196

EPI_ISL_1724198

EPI_ISL_1724199

EPI_ISL_1724201

EPI_ISL_1724202

EPI_ISL_1724203

EPI_ISL_1724204

EPI_ISL_1724205

EPI_ISL_1724206

EPI_ISL_1724207

EPI_ISL_1724209

EPI_ISL_1724210

EPI_ISL_1724213

EPI_ISL_1724214

EPI_ISL_1724215

EPI_ISL_1724217

EPI_ISL_1724218

EPI_ISL_1724220

EPI_ISL_1724221

EPI_ISL_1724223

EPI_ISL_1724224

EPI_ISL_1724225

EPI_ISL_1724226

EPI_ISL_1724227

EPI_ISL_1724228

EPI_ISL_1724229

EPI_ISL_1724230

EPI_ISL_1724231

EPI_ISL_1724232

EPI_ISL_1724233

EPI_ISL_1724234

EPI_ISL_1724235

EPI_ISL_1724236

EPI_ISL_2547453

EPI_ISL_2547454

EPI_ISL_2547455

EPI_ISL_2547456

EPI_ISL_2547457

EPI_ISL_1724248

EPI_ISL_1724249

EPI_ISL_1724251

EPI_ISL_1724252

EPI_ISL_1724253

EPI_ISL_1724254

EPI_ISL_1724257

EPI_ISL_1724258

EPI_ISL_1724259

EPI_ISL_1724260

EPI_ISL_1724261

EPI_ISL_1724262

EPI_ISL_1724263

EPI_ISL_1724264

EPI_ISL_1724265

EPI_ISL_1724266

EPI_ISL_1724267

EPI_ISL_1724268

EPI_ISL_1724269

EPI_ISL_1724270

EPI_ISL_1724271

EPI_ISL_1724272

EPI_ISL_1724274

EPI_ISL_1724276

EPI_ISL_1724277

EPI_ISL_1724278

EPI_ISL_1724279

EPI_ISL_1724280

EPI_ISL_1724281

EPI_ISL_1724282

EPI_ISL_1724283

EPI_ISL_1724285

EPI_ISL_1724286

EPI_ISL_1724288

EPI_ISL_1936908

EPI_ISL_1724289

EPI_ISL_1724290

EPI_ISL_1724291

EPI_ISL_1724292

EPI_ISL_1724698

EPI_ISL_1724295

EPI_ISL_1724296

EPI_ISL_1724297

EPI_ISL_1724298

EPI_ISL_1724300

EPI_ISL_1724301

EPI_ISL_2346128

EPI_ISL_1724302

EPI_ISL_2547476

EPI_ISL_1724303

EPI_ISL_1724304

EPI_ISL_1724305

EPI_ISL_1724306

EPI_ISL_1724312

EPI_ISL_1724699

EPI_ISL_2547477

EPI_ISL_1724700

EPI_ISL_1724315

EPI_ISL_1724316

EPI_ISL_1724317

EPI_ISL_1724320

EPI_ISL_2547479

EPI_ISL_1724322

EPI_ISL_1724323

EPI_ISL_2547480

EPI_ISL_1724324

EPI_ISL_1724326

EPI_ISL_1724327

EPI_ISL_1724337

EPI_ISL_1724701

EPI_ISL_1724702

EPI_ISL_2788392

EPI_ISL_2788644

EPI_ISL_1724361

EPI_ISL_2547482

EPI_ISL_1724374

EPI_ISL_1724703

EPI_ISL_2547483

EPI_ISL_1724704

EPI_ISL_1724705

EPI_ISL_1724706

EPI_ISL_1724707

EPI_ISL_1724708

EPI_ISL_1724393

EPI_ISL_1724394

EPI_ISL_1724395

EPI_ISL_2547488

EPI_ISL_1724396

EPI_ISL_1724397

EPI_ISL_1724709

EPI_ISL_1724398

EPI_ISL_1724399

EPI_ISL_1724400

EPI_ISL_1724401

EPI_ISL_1724402

EPI_ISL_1724403

EPI_ISL_1724404

EPI_ISL_1724405

EPI_ISL_1724407

EPI_ISL_1724408

EPI_ISL_1724409

EPI_ISL_1724410

EPI_ISL_1724411

EPI_ISL_1724412

EPI_ISL_1724413

EPI_ISL_1724414

EPI_ISL_1724415

EPI_ISL_1724416

EPI_ISL_2547504

EPI_ISL_1724417

EPI_ISL_1724418

EPI_ISL_1724419

EPI_ISL_1724420

EPI_ISL_1724421

EPI_ISL_1724423

EPI_ISL_1724425

EPI_ISL_1724426

EPI_ISL_1724427

EPI_ISL_1724428

EPI_ISL_1724430

EPI_ISL_1724432

EPI_ISL_1724433

EPI_ISL_1724434

EPI_ISL_1724710

EPI_ISL_1724711

EPI_ISL_1724712

EPI_ISL_1724713

EPI_ISL_1724439

EPI_ISL_1724440

EPI_ISL_1724441

EPI_ISL_1724445

EPI_ISL_1724715

EPI_ISL_1724448

EPI_ISL_1724450

EPI_ISL_1724451

EPI_ISL_1724452

EPI_ISL_1724453

EPI_ISL_1724455

EPI_ISL_1724456

EPI_ISL_1724457

EPI_ISL_1724458

EPI_ISL_1724459

EPI_ISL_1724460

EPI_ISL_1724461

EPI_ISL_1724462

EPI_ISL_1724463

EPI_ISL_1724717

EPI_ISL_1724719

EPI_ISL_1724720

EPI_ISL_1724465

EPI_ISL_1724466

EPI_ISL_1724467

EPI_ISL_1724468

EPI_ISL_1724469

EPI_ISL_1724470

EPI_ISL_1724471

EPI_ISL_1724472

EPI_ISL_1724473

EPI_ISL_1724474

EPI_ISL_1724475

EPI_ISL_1724476

EPI_ISL_1724477

EPI_ISL_1724721

EPI_ISL_1724478

EPI_ISL_1724479

EPI_ISL_1724480

EPI_ISL_1724481

EPI_ISL_1724482

EPI_ISL_1724722

EPI_ISL_1724483

EPI_ISL_1724484

EPI_ISL_1724723

EPI_ISL_1724724

EPI_ISL_1724725

EPI_ISL_1724486

EPI_ISL_1724487

EPI_ISL_1724488

EPI_ISL_1724489

EPI_ISL_1724490

EPI_ISL_1724491

EPI_ISL_1724492

EPI_ISL_1724493

EPI_ISL_1724726

EPI_ISL_1724727

EPI_ISL_1724495

EPI_ISL_1724496

EPI_ISL_1724497

EPI_ISL_1724498

EPI_ISL_1724499

EPI_ISL_1724500

EPI_ISL_1724502

EPI_ISL_1724503

EPI_ISL_1724505

EPI_ISL_1724506

EPI_ISL_1724728

EPI_ISL_1724730

EPI_ISL_1724508

EPI_ISL_1724509

EPI_ISL_1724510

EPI_ISL_1724511

EPI_ISL_1724512

EPI_ISL_1724513

EPI_ISL_1724514

EPI_ISL_1724516

EPI_ISL_1724518

EPI_ISL_1724520

EPI_ISL_1724521

EPI_ISL_1724522

EPI_ISL_1724523

EPI_ISL_1724524

EPI_ISL_1724525

EPI_ISL_1724526

EPI_ISL_1724527

EPI_ISL_1724528

EPI_ISL_1724529

EPI_ISL_1724530

EPI_ISL_1724531

EPI_ISL_1724532

EPI_ISL_1724533

EPI_ISL_1724534

EPI_ISL_1724535

EPI_ISL_1724536

EPI_ISL_1724537

EPI_ISL_1724539

EPI_ISL_1724540

EPI_ISL_1724541

EPI_ISL_1724542

EPI_ISL_1724543

EPI_ISL_1724544

EPI_ISL_1724545

EPI_ISL_1724546

EPI_ISL_1724547

EPI_ISL_1724548

EPI_ISL_1724550

EPI_ISL_1724551

EPI_ISL_1724552

EPI_ISL_1724553

EPI_ISL_1724554

EPI_ISL_1724555

EPI_ISL_1724556

EPI_ISL_1724557

EPI_ISL_1724558

EPI_ISL_1724559

EPI_ISL_1724560

EPI_ISL_1724561

EPI_ISL_1724562

EPI_ISL_1724563

EPI_ISL_1724564

EPI_ISL_1724565

EPI_ISL_1724566

EPI_ISL_1724567

EPI_ISL_1724569

EPI_ISL_1724570

EPI_ISL_1724571

EPI_ISL_1724572

EPI_ISL_1724573

EPI_ISL_1724575

EPI_ISL_1583986

EPI_ISL_1724576

EPI_ISL_1724577

EPI_ISL_1724578

EPI_ISL_1724579

EPI_ISL_1724580

EPI_ISL_1724581

EPI_ISL_1724583

EPI_ISL_1724584

EPI_ISL_1724585

EPI_ISL_1724586

EPI_ISL_1724588

EPI_ISL_1724589

EPI_ISL_1724590

EPI_ISL_1724591

EPI_ISL_1724593

EPI_ISL_1724594

EPI_ISL_1724595

EPI_ISL_1724596

EPI_ISL_1724597

EPI_ISL_1724598

EPI_ISL_1724599

EPI_ISL_1724600

EPI_ISL_1724601

EPI_ISL_1724602

EPI_ISL_1724603

EPI_ISL_1724604

EPI_ISL_1724605

EPI_ISL_1724606

EPI_ISL_1724607

EPI_ISL_1724608

EPI_ISL_1724609

EPI_ISL_1724610

EPI_ISL_1724611

EPI_ISL_1724612

EPI_ISL_1724614

EPI_ISL_1724615

EPI_ISL_1724616

EPI_ISL_1724617

EPI_ISL_1724618

EPI_ISL_1724619

EPI_ISL_1724620

EPI_ISL_1724621

EPI_ISL_1724622

EPI_ISL_1724624

EPI_ISL_1724626

EPI_ISL_1724627

EPI_ISL_1724628

EPI_ISL_1724629

EPI_ISL_1724630

EPI_ISL_1724631

EPI_ISL_1724632

EPI_ISL_1583999

EPI_ISL_1724634

EPI_ISL_1724635

EPI_ISL_1724637

EPI_ISL_2547555

EPI_ISL_1724638

EPI_ISL_3559278

EPI_ISL_3559281

EPI_ISL_3559282

EPI_ISL_3559283

EPI_ISL_3559284

EPI_ISL_3559285

EPI_ISL_1724640

EPI_ISL_1724641

EPI_ISL_1724642

EPI_ISL_1724643

EPI_ISL_1724644

EPI_ISL_1724645

EPI_ISL_1724646

EPI_ISL_1724647

EPI_ISL_1724648

EPI_ISL_1724650

EPI_ISL_1724651

EPI_ISL_2790322

EPI_ISL_1724732

EPI_ISL_1724733

EPI_ISL_1724734

EPI_ISL_1724735

EPI_ISL_1724736

EPI_ISL_1724738

EPI_ISL_1724742

EPI_ISL_1724743

EPI_ISL_1724744

EPI_ISL_1724745

EPI_ISL_1724746

EPI_ISL_1724747

EPI_ISL_1724748

EPI_ISL_1724750

EPI_ISL_1724751

EPI_ISL_1724752

EPI_ISL_1724754

EPI_ISL_1724757

EPI_ISL_1724758

EPI_ISL_1724759

EPI_ISL_1724760

EPI_ISL_1724762

EPI_ISL_1724763

EPI_ISL_1724764

EPI_ISL_1724765

EPI_ISL_1724766

EPI_ISL_1724767

EPI_ISL_1724768

EPI_ISL_1724769

EPI_ISL_1724771

EPI_ISL_1724772

EPI_ISL_1724773

EPI_ISL_1724774

EPI_ISL_1724775

EPI_ISL_1724776

EPI_ISL_1724778

EPI_ISL_1724779

EPI_ISL_1724780

EPI_ISL_1724782

EPI_ISL_1724783

EPI_ISL_1724784

EPI_ISL_1937466

EPI_ISL_1724786

EPI_ISL_1724787

EPI_ISL_1724788

EPI_ISL_1724789

EPI_ISL_1724790

EPI_ISL_1724791

EPI_ISL_1724792

EPI_ISL_1724793

EPI_ISL_1724794

EPI_ISL_1724796

EPI_ISL_1724797

EPI_ISL_1724798

EPI_ISL_1724799

EPI_ISL_1724800

EPI_ISL_1724802

EPI_ISL_1724803

EPI_ISL_1724805

EPI_ISL_1724808

EPI_ISL_1724810

EPI_ISL_1724811

EPI_ISL_1724812

EPI_ISL_1724813

EPI_ISL_1724814

EPI_ISL_1724815

EPI_ISL_1724816

EPI_ISL_1724818

EPI_ISL_1724819

EPI_ISL_1724820

EPI_ISL_1724821

EPI_ISL_1724823

EPI_ISL_1724824

EPI_ISL_1724825

EPI_ISL_1724826

EPI_ISL_1724827

EPI_ISL_1724828

EPI_ISL_1724829

EPI_ISL_1724830

EPI_ISL_1724831

EPI_ISL_1724832

EPI_ISL_1724833

EPI_ISL_1724834

EPI_ISL_1724837

EPI_ISL_1724839

EPI_ISL_1724840

EPI_ISL_1724841

EPI_ISL_1724842

EPI_ISL_1724843

EPI_ISL_1724844

EPI_ISL_1724845

EPI_ISL_1724846

EPI_ISL_1724847

EPI_ISL_1724849

EPI_ISL_1724850

EPI_ISL_1724851

EPI_ISL_1724853

EPI_ISL_1724854

EPI_ISL_1724855

EPI_ISL_1724857

EPI_ISL_1724858

EPI_ISL_1724859

EPI_ISL_1724860

EPI_ISL_1724861

EPI_ISL_1724862

EPI_ISL_1724864

EPI_ISL_1724865

EPI_ISL_1724866

EPI_ISL_1724867

EPI_ISL_1724868

EPI_ISL_1724869

EPI_ISL_1724871

EPI_ISL_1724872

EPI_ISL_1724874

EPI_ISL_1724875

EPI_ISL_1724876

EPI_ISL_1724877

EPI_ISL_1724879

EPI_ISL_1724880

EPI_ISL_1724881

EPI_ISL_1724882

EPI_ISL_1724883

EPI_ISL_1724884

EPI_ISL_1724885

EPI_ISL_1724888

EPI_ISL_1724889

EPI_ISL_1724892

EPI_ISL_1724893

EPI_ISL_1724894

EPI_ISL_1724895

EPI_ISL_1724896

EPI_ISL_1724897

EPI_ISL_1724898

EPI_ISL_1724899

EPI_ISL_1724901

EPI_ISL_1724904

EPI_ISL_1724905

EPI_ISL_1724906

EPI_ISL_1724908

EPI_ISL_1724909

EPI_ISL_1724910

EPI_ISL_1724911

EPI_ISL_1724912

EPI_ISL_1724914

EPI_ISL_1724915

EPI_ISL_1724916

EPI_ISL_1724917

EPI_ISL_1724918

EPI_ISL_1724919

EPI_ISL_1724920

EPI_ISL_1724921

EPI_ISL_1724922

EPI_ISL_1724923

EPI_ISL_1724924

EPI_ISL_1724925

EPI_ISL_1724931

EPI_ISL_1724932

EPI_ISL_1724934

EPI_ISL_1724937

EPI_ISL_1724938

EPI_ISL_1724940

EPI_ISL_1724941

EPI_ISL_1724943

EPI_ISL_1724944

EPI_ISL_1724945

EPI_ISL_1724946

EPI_ISL_1724947

EPI_ISL_1724948

EPI_ISL_1724951

EPI_ISL_1724952

EPI_ISL_1724954

EPI_ISL_1724956

EPI_ISL_1724957

EPI_ISL_1724958

EPI_ISL_1724959

EPI_ISL_1724960

EPI_ISL_1724961

EPI_ISL_1724962

EPI_ISL_1724964

EPI_ISL_1724965

EPI_ISL_1724966

EPI_ISL_1724968

EPI_ISL_1724969

EPI_ISL_1724970

EPI_ISL_1724971

EPI_ISL_1724972

EPI_ISL_1724974

EPI_ISL_1724975

EPI_ISL_1724976

EPI_ISL_1724977

EPI_ISL_1724978

EPI_ISL_1724980

EPI_ISL_1724981

EPI_ISL_1724982

EPI_ISL_1724983

EPI_ISL_1724984

EPI_ISL_1724986

EPI_ISL_1724987

EPI_ISL_1724988

EPI_ISL_1724989

EPI_ISL_1724991

EPI_ISL_1724992

EPI_ISL_1724993

EPI_ISL_1724994

EPI_ISL_1724995

EPI_ISL_1724996

EPI_ISL_1724997

EPI_ISL_1724998

EPI_ISL_1724999

EPI_ISL_1725000

EPI_ISL_1725001

EPI_ISL_1725002

EPI_ISL_1725003

EPI_ISL_1725004

EPI_ISL_1725005

EPI_ISL_1725006

EPI_ISL_1725007

EPI_ISL_1725008

EPI_ISL_1725009

EPI_ISL_1725010

EPI_ISL_1725011

EPI_ISL_1725012

EPI_ISL_1725014

EPI_ISL_1725016

EPI_ISL_1725018

EPI_ISL_1725019

EPI_ISL_1725022

EPI_ISL_1725023

EPI_ISL_1725025

EPI_ISL_1725026

EPI_ISL_1725027

EPI_ISL_1725028

EPI_ISL_1725029

EPI_ISL_1725031

EPI_ISL_1725032

EPI_ISL_1725033

EPI_ISL_1725034

EPI_ISL_1725035

EPI_ISL_1725036

EPI_ISL_1725041

EPI_ISL_1725042

EPI_ISL_1725043

EPI_ISL_1725044

EPI_ISL_1725045

EPI_ISL_1725046

EPI_ISL_1725047

EPI_ISL_1725048

EPI_ISL_1725049

EPI_ISL_1725051

EPI_ISL_1725052

EPI_ISL_1725053

EPI_ISL_1725054

EPI_ISL_1725055

EPI_ISL_1725056

EPI_ISL_1725060

EPI_ISL_1725061

EPI_ISL_1725062

EPI_ISL_1725063

EPI_ISL_1725065

EPI_ISL_1725066

EPI_ISL_1725067

EPI_ISL_1725068

EPI_ISL_1725069

EPI_ISL_1725070

EPI_ISL_1725071

EPI_ISL_1725072

EPI_ISL_1725073

EPI_ISL_1725075

EPI_ISL_1725078

EPI_ISL_1725079

EPI_ISL_1725080

EPI_ISL_1725081

EPI_ISL_1725084

EPI_ISL_1725086

EPI_ISL_1725087

EPI_ISL_1725088

EPI_ISL_1725089

EPI_ISL_1725090

EPI_ISL_1725091

EPI_ISL_1725092

EPI_ISL_1725093

EPI_ISL_1725097

EPI_ISL_1725098

EPI_ISL_1725099

EPI_ISL_1725100

EPI_ISL_1725101

EPI_ISL_1725103

EPI_ISL_1725105

EPI_ISL_1725106

EPI_ISL_1725107

EPI_ISL_1725108

EPI_ISL_1725109

EPI_ISL_1725110

EPI_ISL_1725111

EPI_ISL_1725113

EPI_ISL_1725114

EPI_ISL_1725115

EPI_ISL_1725116

EPI_ISL_1725119

EPI_ISL_1725120

EPI_ISL_1725121

EPI_ISL_1725122

EPI_ISL_1725123

EPI_ISL_1725124

EPI_ISL_1725125

EPI_ISL_1725127

EPI_ISL_1725128

EPI_ISL_1725129

EPI_ISL_1725131

EPI_ISL_1725132

EPI_ISL_1725133

EPI_ISL_1725134

EPI_ISL_1725135

EPI_ISL_1725136

EPI_ISL_1725137

EPI_ISL_1725138

EPI_ISL_1725139

EPI_ISL_1725140

EPI_ISL_1725141

EPI_ISL_1725142

EPI_ISL_1725143

EPI_ISL_1725146

EPI_ISL_1725148

EPI_ISL_1725149

EPI_ISL_1725150

EPI_ISL_1725151

EPI_ISL_1725152

EPI_ISL_1725153

EPI_ISL_1725154

EPI_ISL_1725155

EPI_ISL_1725156

EPI_ISL_1725157

EPI_ISL_1725158

EPI_ISL_1725162

EPI_ISL_1725166

EPI_ISL_1725167

EPI_ISL_1725168

EPI_ISL_1725169

EPI_ISL_1725170

EPI_ISL_1725172

EPI_ISL_1725173

EPI_ISL_1725174

EPI_ISL_1725190

EPI_ISL_1725191

EPI_ISL_1725193

EPI_ISL_1725194

EPI_ISL_1725195

EPI_ISL_1725196

EPI_ISL_1725197

EPI_ISL_1725198

EPI_ISL_1725199

EPI_ISL_1725201

EPI_ISL_1725202

EPI_ISL_1725203

EPI_ISL_1725204

EPI_ISL_1725206

EPI_ISL_1725207

EPI_ISL_1725208

EPI_ISL_1725209

EPI_ISL_1725210

EPI_ISL_1725211

EPI_ISL_1725212

EPI_ISL_1725213

EPI_ISL_1725215

EPI_ISL_1725216

EPI_ISL_1725217

EPI_ISL_1725221

EPI_ISL_1725230

EPI_ISL_1725231

EPI_ISL_1725236

EPI_ISL_1725238

EPI_ISL_1725239

EPI_ISL_1725240

EPI_ISL_1725241

EPI_ISL_1725242

EPI_ISL_1725244

EPI_ISL_1725246

EPI_ISL_1725250

EPI_ISL_1725251

EPI_ISL_1725252

EPI_ISL_1725253

EPI_ISL_1725254

EPI_ISL_1725255

EPI_ISL_1725256

EPI_ISL_1725259

EPI_ISL_1725260

EPI_ISL_1725262

EPI_ISL_1725264

EPI_ISL_1725265

EPI_ISL_1725267

EPI_ISL_1725268

EPI_ISL_1725269

EPI_ISL_1725270

EPI_ISL_1725271

EPI_ISL_1725274

EPI_ISL_1725277

EPI_ISL_1725312

EPI_ISL_2791983

EPI_ISL_1937129

EPI_ISL_1937352

EPI_ISL_2547819

EPI_ISL_2547821

EPI_ISL_1725431

EPI_ISL_1725438

EPI_ISL_1725831

EPI_ISL_1725832

EPI_ISL_1725833

EPI_ISL_1725834

EPI_ISL_1725835

EPI_ISL_4186753

EPI_ISL_1725836

EPI_ISL_3598020

EPI_ISL_4346610

EPI_ISL_2547913

EPI_ISL_1585831

EPI_ISL_2547924

EPI_ISL_2547925

EPI_ISL_2547926

EPI_ISL_2547927

EPI_ISL_1725811

EPI_ISL_2547928

EPI_ISL_2547929

EPI_ISL_1725597

EPI_ISL_1725598

EPI_ISL_1725599

EPI_ISL_1725600

EPI_ISL_1725601

EPI_ISL_1725602

EPI_ISL_1725603

EPI_ISL_1725604

EPI_ISL_1725605

EPI_ISL_1725606

EPI_ISL_1725607

EPI_ISL_1725608

EPI_ISL_1725610

EPI_ISL_1725611

EPI_ISL_1725613

EPI_ISL_1725620

EPI_ISL_1725621

EPI_ISL_1725624

EPI_ISL_1725625

EPI_ISL_1725627

EPI_ISL_1725628

EPI_ISL_1725630

EPI_ISL_1725631

EPI_ISL_1725632

EPI_ISL_1725634

EPI_ISL_1725637

EPI_ISL_1725638

EPI_ISL_1725642

EPI_ISL_1725643

EPI_ISL_1725647

EPI_ISL_2547932

EPI_ISL_1725649

EPI_ISL_2547933

EPI_ISL_1725650

EPI_ISL_2547934

EPI_ISL_2547935

EPI_ISL_2547936

EPI_ISL_1725653

EPI_ISL_1725654

EPI_ISL_1725655

EPI_ISL_2547937

EPI_ISL_1725656

EPI_ISL_1725658

EPI_ISL_2547938

EPI_ISL_1725659

EPI_ISL_1725660

EPI_ISL_1725661

EPI_ISL_1725662

EPI_ISL_1725663

EPI_ISL_2547941

EPI_ISL_2547942

EPI_ISL_1725664

EPI_ISL_1725665

EPI_ISL_2547943

EPI_ISL_1725668

EPI_ISL_1725669

EPI_ISL_1725670

EPI_ISL_2547944

EPI_ISL_1725673

EPI_ISL_1725674

EPI_ISL_1725675

EPI_ISL_1725676

EPI_ISL_1725678

EPI_ISL_2547945

EPI_ISL_2547946

EPI_ISL_1725679

EPI_ISL_1725680

EPI_ISL_2547949

EPI_ISL_2547951

EPI_ISL_1725682

EPI_ISL_1725683

EPI_ISL_1725685

EPI_ISL_1725686

EPI_ISL_2547954

EPI_ISL_1725813

EPI_ISL_1725814

EPI_ISL_1725688

EPI_ISL_1725689

EPI_ISL_1725690

EPI_ISL_1725692

EPI_ISL_1725693

EPI_ISL_1725695

EPI_ISL_1725696

EPI_ISL_1725697

EPI_ISL_1725699

EPI_ISL_1725700

EPI_ISL_1725701

EPI_ISL_1725702

EPI_ISL_1725703

EPI_ISL_1725704

EPI_ISL_1725706

EPI_ISL_1725707

EPI_ISL_1725709

EPI_ISL_1725710

EPI_ISL_1725713

EPI_ISL_1725714

EPI_ISL_1725715

EPI_ISL_1725716

EPI_ISL_1725717

EPI_ISL_1725718

EPI_ISL_1725719

EPI_ISL_1725724

EPI_ISL_1725725

EPI_ISL_1725726

EPI_ISL_1725728

EPI_ISL_1725729

EPI_ISL_1725730

EPI_ISL_1725731

EPI_ISL_1725732

EPI_ISL_1725733

EPI_ISL_1725734

EPI_ISL_1725735

EPI_ISL_1725736

EPI_ISL_1725737

EPI_ISL_1725738

EPI_ISL_1725739

EPI_ISL_1725740

EPI_ISL_1725741

EPI_ISL_1725742

EPI_ISL_1725743

EPI_ISL_1725744

EPI_ISL_1725745

EPI_ISL_1725746

EPI_ISL_1725747

EPI_ISL_1725748

EPI_ISL_1725817

EPI_ISL_1725750

EPI_ISL_1725751

EPI_ISL_1725752

EPI_ISL_1725818

EPI_ISL_1725754

EPI_ISL_1725755

EPI_ISL_1725756

EPI_ISL_1725757

EPI_ISL_1725759

EPI_ISL_1725819

EPI_ISL_1725820

EPI_ISL_1725821

EPI_ISL_1725761

EPI_ISL_1725763

EPI_ISL_1725764

EPI_ISL_1725765

EPI_ISL_1725822

EPI_ISL_1725766

EPI_ISL_1725767

EPI_ISL_1725768

EPI_ISL_1725769

EPI_ISL_1725770

EPI_ISL_1725771

EPI_ISL_1725823

EPI_ISL_1725824

EPI_ISL_1725825

EPI_ISL_1725773

EPI_ISL_1725774

EPI_ISL_2547966

EPI_ISL_1725775

EPI_ISL_1725826

EPI_ISL_1725777

EPI_ISL_1725778

EPI_ISL_1725779

EPI_ISL_1725780

EPI_ISL_1725782

EPI_ISL_1725827

EPI_ISL_1725828

EPI_ISL_1725829

EPI_ISL_1725784

EPI_ISL_1725786

EPI_ISL_1725787

EPI_ISL_2547967

EPI_ISL_1725788

EPI_ISL_1725791

EPI_ISL_1725793

EPI_ISL_1725794

EPI_ISL_1725795

EPI_ISL_1725796

EPI_ISL_1725797

EPI_ISL_1725798

EPI_ISL_1725799

EPI_ISL_1725800

EPI_ISL_1725801

EPI_ISL_1725802

EPI_ISL_1725803

EPI_ISL_1725805

EPI_ISL_1585347

EPI_ISL_1725806

EPI_ISL_1725807

EPI_ISL_1725808

EPI_ISL_1725838

EPI_ISL_1725840

EPI_ISL_2547978

EPI_ISL_1725841

EPI_ISL_1725842

EPI_ISL_1725843

EPI_ISL_2799581

EPI_ISL_1726381

EPI_ISL_2799582

EPI_ISL_2799583

EPI_ISL_2547981

EPI_ISL_1725846

EPI_ISL_1725847

EPI_ISL_1725848

EPI_ISL_1725849

EPI_ISL_2547982

EPI_ISL_2547983

EPI_ISL_1725852

EPI_ISL_1725853

EPI_ISL_1725854

EPI_ISL_1725855

EPI_ISL_1725856

EPI_ISL_1726412

EPI_ISL_1725857

EPI_ISL_2547984

EPI_ISL_1725859

EPI_ISL_2547986

EPI_ISL_1725860

EPI_ISL_2547987

EPI_ISL_2547988

EPI_ISL_1726383

EPI_ISL_1725863

EPI_ISL_1726384

EPI_ISL_1725865

EPI_ISL_1725866

EPI_ISL_1725867

EPI_ISL_1725869

EPI_ISL_1725870

EPI_ISL_1725871

EPI_ISL_1725872

EPI_ISL_1725873

EPI_ISL_1725874

EPI_ISL_1725875

EPI_ISL_2547989

EPI_ISL_1725876

EPI_ISL_1725877

EPI_ISL_1725878

EPI_ISL_1726385

EPI_ISL_1725880

EPI_ISL_1725881

EPI_ISL_2547990

EPI_ISL_1725882

EPI_ISL_1725883

EPI_ISL_1725884

EPI_ISL_1725885

EPI_ISL_1725886

EPI_ISL_1725887

EPI_ISL_1725888

EPI_ISL_2547992

EPI_ISL_1725889

EPI_ISL_2547993

EPI_ISL_2547994

EPI_ISL_1725890

EPI_ISL_1725891

EPI_ISL_1725892

EPI_ISL_1725893

EPI_ISL_1725895

EPI_ISL_2547995

EPI_ISL_1725896

EPI_ISL_1725898

EPI_ISL_1725899

EPI_ISL_1725900

EPI_ISL_1725901

EPI_ISL_1726413

EPI_ISL_1726414

EPI_ISL_1726387

EPI_ISL_1726388

EPI_ISL_1726390

EPI_ISL_1726391

EPI_ISL_1726392

EPI_ISL_1726393

EPI_ISL_1726415

EPI_ISL_1726416

EPI_ISL_1725903

EPI_ISL_1725904

EPI_ISL_1725905

EPI_ISL_1725906

EPI_ISL_1725907

EPI_ISL_1725908

EPI_ISL_2547997

EPI_ISL_1725909

EPI_ISL_2547998

EPI_ISL_2547999

EPI_ISL_1725912

EPI_ISL_1725913

EPI_ISL_1725914

EPI_ISL_1725915

EPI_ISL_1725916

EPI_ISL_2548000

EPI_ISL_1725917

EPI_ISL_2548002

EPI_ISL_2548003

EPI_ISL_1725918

EPI_ISL_1725919

EPI_ISL_2548004

EPI_ISL_1725921

EPI_ISL_2548006

EPI_ISL_2548007

EPI_ISL_1726417

EPI_ISL_2548008

EPI_ISL_1725923

EPI_ISL_1725925

EPI_ISL_2548012

EPI_ISL_1725926

EPI_ISL_1725928

EPI_ISL_1725929

EPI_ISL_2548013

EPI_ISL_1725931

EPI_ISL_1725932

EPI_ISL_1725933

EPI_ISL_1725935

EPI_ISL_1725936

EPI_ISL_1725937

EPI_ISL_1725938

EPI_ISL_1725939

EPI_ISL_1725940

EPI_ISL_1725941

EPI_ISL_1725942

EPI_ISL_1725943

EPI_ISL_1725944

EPI_ISL_1725946

EPI_ISL_1725948

EPI_ISL_1725949

EPI_ISL_1725950

EPI_ISL_1725951

EPI_ISL_1725952

EPI_ISL_1725954

EPI_ISL_1725959

EPI_ISL_1725960

EPI_ISL_1725963

EPI_ISL_1725964

EPI_ISL_1725965

EPI_ISL_1725966

EPI_ISL_1725967

EPI_ISL_1726419

EPI_ISL_1725969

EPI_ISL_1726420

EPI_ISL_1725971

EPI_ISL_1725972

EPI_ISL_1725973

EPI_ISL_1725974

EPI_ISL_1726421

EPI_ISL_1725975

EPI_ISL_1725976

EPI_ISL_1725977

EPI_ISL_1725978

EPI_ISL_1725979

EPI_ISL_2548015

EPI_ISL_2548017

EPI_ISL_1725980

EPI_ISL_1725981

EPI_ISL_2548018

EPI_ISL_1725983

EPI_ISL_2548020

EPI_ISL_1725984

EPI_ISL_1725985

EPI_ISL_1725987

EPI_ISL_1725988

EPI_ISL_1725989

EPI_ISL_1725990

EPI_ISL_1725991

EPI_ISL_1725992

EPI_ISL_1725994

EPI_ISL_1725995

EPI_ISL_1725996

EPI_ISL_1725998

EPI_ISL_1725999

EPI_ISL_1726000

EPI_ISL_1726001

EPI_ISL_1726002

EPI_ISL_1726003

EPI_ISL_1726004

EPI_ISL_1726005

EPI_ISL_1726007

EPI_ISL_1726008

EPI_ISL_1726009

EPI_ISL_1726012

EPI_ISL_1726013

EPI_ISL_1726014

EPI_ISL_1726015

EPI_ISL_1726017

EPI_ISL_1726023

EPI_ISL_1726024

EPI_ISL_1726026

EPI_ISL_1726027

EPI_ISL_1726028

EPI_ISL_1726029

EPI_ISL_1726030

EPI_ISL_1726031

EPI_ISL_1726034

EPI_ISL_1726035

EPI_ISL_1726037

EPI_ISL_1726038

EPI_ISL_1726040

EPI_ISL_1726041

EPI_ISL_1726042

EPI_ISL_1726043

EPI_ISL_1726044

EPI_ISL_1726045

EPI_ISL_1726046

EPI_ISL_1726047

EPI_ISL_1726048

EPI_ISL_1726050

EPI_ISL_1726052

EPI_ISL_1726054

EPI_ISL_1726055

EPI_ISL_1726056

EPI_ISL_1726059

EPI_ISL_1726060

EPI_ISL_1726062

EPI_ISL_1726063

EPI_ISL_1726064

EPI_ISL_1726065

EPI_ISL_1726066

EPI_ISL_1726067

EPI_ISL_1726069

EPI_ISL_1726070

EPI_ISL_1726071

EPI_ISL_1726072

EPI_ISL_1726073

EPI_ISL_1726074

EPI_ISL_1726076

EPI_ISL_1726082

EPI_ISL_1726083

EPI_ISL_1726084

EPI_ISL_1726085

EPI_ISL_1726086

EPI_ISL_1726087

EPI_ISL_1726090

EPI_ISL_1726092

EPI_ISL_1726093

EPI_ISL_1726095

EPI_ISL_1726096

EPI_ISL_1726097

EPI_ISL_1726098

EPI_ISL_1726099

EPI_ISL_1726100

EPI_ISL_1726101

EPI_ISL_1726102

EPI_ISL_1726104

EPI_ISL_1726106

EPI_ISL_1726107

EPI_ISL_1726108

EPI_ISL_1726109

EPI_ISL_1726111

EPI_ISL_1726112

EPI_ISL_1726113

EPI_ISL_1726114

EPI_ISL_1726115

EPI_ISL_1726116

EPI_ISL_1726117

EPI_ISL_1726118

EPI_ISL_1726119

EPI_ISL_1726120

EPI_ISL_1726121

EPI_ISL_1726122

EPI_ISL_1726123

EPI_ISL_1726124

EPI_ISL_1726125

EPI_ISL_1726126

EPI_ISL_1726127

EPI_ISL_1726129

EPI_ISL_1726130

EPI_ISL_1726131

EPI_ISL_1726132

EPI_ISL_1726133

EPI_ISL_1726134

EPI_ISL_1726135

EPI_ISL_1726136

EPI_ISL_1726137

EPI_ISL_1726138

EPI_ISL_1726139

EPI_ISL_1726141

EPI_ISL_1726422

EPI_ISL_1726423

EPI_ISL_1726394

EPI_ISL_1726396

EPI_ISL_1726424

EPI_ISL_1726144

EPI_ISL_1726145

EPI_ISL_1726147

EPI_ISL_1726148

EPI_ISL_1726149

EPI_ISL_1726150

EPI_ISL_1726425

EPI_ISL_1726153

EPI_ISL_1726155

EPI_ISL_1726156

EPI_ISL_1726157

EPI_ISL_1726159

EPI_ISL_1726161

EPI_ISL_1726162

EPI_ISL_1726163

EPI_ISL_1726164

EPI_ISL_1726165

EPI_ISL_1726167

EPI_ISL_1726168

EPI_ISL_1726169

EPI_ISL_1726170

EPI_ISL_1726171

EPI_ISL_1726172

EPI_ISL_1726173

EPI_ISL_1726174

EPI_ISL_1726175

EPI_ISL_1726178

EPI_ISL_1726179

EPI_ISL_1726180

EPI_ISL_1726181

EPI_ISL_1726182

EPI_ISL_1726183

EPI_ISL_1726185

EPI_ISL_1726187

EPI_ISL_1726188

EPI_ISL_1726426

EPI_ISL_1726189

EPI_ISL_1726191

EPI_ISL_1726397

EPI_ISL_1726193

EPI_ISL_1726194

EPI_ISL_1726195

EPI_ISL_1726197

EPI_ISL_1726398

EPI_ISL_1726199

EPI_ISL_1726200

EPI_ISL_1726202

EPI_ISL_1726203

EPI_ISL_1726204

EPI_ISL_1726205

EPI_ISL_1726206

EPI_ISL_1726208

EPI_ISL_1726209

EPI_ISL_1726210

EPI_ISL_1726211

EPI_ISL_1726212

EPI_ISL_1726213

EPI_ISL_1726214

EPI_ISL_1726215

EPI_ISL_1726216

EPI_ISL_1726217

EPI_ISL_1726218

EPI_ISL_1726219

EPI_ISL_1726220

EPI_ISL_1726221

EPI_ISL_1726222

EPI_ISL_1726223

EPI_ISL_1726224

EPI_ISL_1726226

EPI_ISL_1726228

EPI_ISL_1726230

EPI_ISL_1726231

EPI_ISL_1726232

EPI_ISL_1726234

EPI_ISL_1726235

EPI_ISL_1726236

EPI_ISL_1726238

EPI_ISL_1726240

EPI_ISL_1726241

EPI_ISL_1726242

EPI_ISL_1726243

EPI_ISL_1726244

EPI_ISL_1726245

EPI_ISL_1726246

EPI_ISL_1726247

EPI_ISL_1726248

EPI_ISL_1726249

EPI_ISL_1726250

EPI_ISL_1726251

EPI_ISL_1726253

EPI_ISL_1726254

EPI_ISL_1726256

EPI_ISL_1726257

EPI_ISL_1726258

EPI_ISL_1726259

EPI_ISL_1726260

EPI_ISL_1726261

EPI_ISL_1726262

EPI_ISL_1726263

EPI_ISL_1726264

EPI_ISL_1726266

EPI_ISL_1726269

EPI_ISL_1726270

EPI_ISL_1726271

EPI_ISL_1726272

EPI_ISL_1726273

EPI_ISL_1726274

EPI_ISL_1726276

EPI_ISL_1726277

EPI_ISL_1726278

EPI_ISL_1726279

EPI_ISL_1726280

EPI_ISL_1726281

EPI_ISL_1726282

EPI_ISL_1726285

EPI_ISL_1726286

EPI_ISL_1726288

EPI_ISL_1726289

EPI_ISL_1726290

EPI_ISL_1726291

EPI_ISL_1726292

EPI_ISL_1726294

EPI_ISL_1726295

EPI_ISL_1726296

EPI_ISL_1726297

EPI_ISL_1726298

EPI_ISL_1726302

EPI_ISL_1726303

EPI_ISL_1726399

EPI_ISL_1726306

EPI_ISL_1726307

EPI_ISL_1726308

EPI_ISL_1726309

EPI_ISL_1726310

EPI_ISL_1726311

EPI_ISL_1726400

EPI_ISL_1726401

EPI_ISL_1726402

EPI_ISL_1726404

EPI_ISL_1726405

EPI_ISL_1726313

EPI_ISL_1726314

EPI_ISL_1726315

EPI_ISL_1726316

EPI_ISL_1726318

EPI_ISL_1726319

EPI_ISL_1726320

EPI_ISL_1726321

EPI_ISL_1726322

EPI_ISL_1726323

EPI_ISL_1726324

EPI_ISL_1726325

EPI_ISL_1726326

EPI_ISL_1726327

EPI_ISL_1726328

EPI_ISL_1726329

EPI_ISL_1726330

EPI_ISL_1726331

EPI_ISL_1726333

EPI_ISL_1726334

EPI_ISL_1726335

EPI_ISL_1726336

EPI_ISL_1726337

EPI_ISL_1726338

EPI_ISL_1726340

EPI_ISL_1726341

EPI_ISL_1726342

EPI_ISL_1726343

EPI_ISL_1726344

EPI_ISL_1726345

EPI_ISL_1726408

EPI_ISL_1726347

EPI_ISL_1726348

EPI_ISL_1726349

EPI_ISL_1726350

EPI_ISL_1726351

EPI_ISL_1726352

EPI_ISL_1726353

EPI_ISL_1726409

EPI_ISL_1726354

EPI_ISL_1726355

EPI_ISL_1726356

EPI_ISL_1726357

EPI_ISL_1726358

EPI_ISL_1726359

EPI_ISL_1726361

EPI_ISL_1726362

EPI_ISL_1726363

EPI_ISL_1726364

EPI_ISL_1726365

EPI_ISL_1726366

EPI_ISL_1726367

EPI_ISL_1726368

EPI_ISL_1726369

EPI_ISL_1726370

EPI_ISL_1726371

EPI_ISL_1726372

EPI_ISL_1726373

EPI_ISL_1726410

EPI_ISL_1726411

EPI_ISL_1726376

EPI_ISL_1726377

EPI_ISL_1726378

EPI_ISL_1726379

EPI_ISL_1727140

EPI_ISL_1726429

EPI_ISL_1726430

EPI_ISL_1727141

EPI_ISL_1726431

EPI_ISL_1726432

EPI_ISL_1726433

EPI_ISL_1726434

EPI_ISL_1726435

EPI_ISL_1726436

EPI_ISL_1726437

EPI_ISL_1726440

EPI_ISL_1726441

EPI_ISL_1726442

EPI_ISL_1726443

EPI_ISL_1726444

EPI_ISL_1726445

EPI_ISL_1726446

EPI_ISL_1726447

EPI_ISL_1727142

EPI_ISL_1726449

EPI_ISL_1726450

EPI_ISL_1726451

EPI_ISL_1726452

EPI_ISL_1726453

EPI_ISL_1727143

EPI_ISL_1726454

EPI_ISL_1726456

EPI_ISL_1726457

EPI_ISL_1726458

EPI_ISL_1726459

EPI_ISL_1726461

EPI_ISL_1726462

EPI_ISL_1727144

EPI_ISL_1727145

EPI_ISL_1727146

EPI_ISL_1727147

EPI_ISL_1727148

EPI_ISL_1727149

EPI_ISL_1727150

EPI_ISL_1727151

EPI_ISL_1727152

EPI_ISL_1726464

EPI_ISL_1726465

EPI_ISL_1726466

EPI_ISL_1726467

EPI_ISL_1726470

EPI_ISL_1727153

EPI_ISL_1727154

EPI_ISL_1727155

EPI_ISL_1726473

EPI_ISL_1726478

EPI_ISL_1726479

EPI_ISL_1726480

EPI_ISL_1726481

EPI_ISL_2548166

EPI_ISL_2548167

EPI_ISL_1726484

EPI_ISL_1726485

EPI_ISL_1726486

EPI_ISL_2548168

EPI_ISL_1727156

EPI_ISL_2799865

EPI_ISL_1726490

EPI_ISL_1726491

EPI_ISL_1726492

EPI_ISL_1726493

EPI_ISL_2548169

EPI_ISL_2548170

EPI_ISL_1727158

EPI_ISL_2548171

EPI_ISL_1727159

EPI_ISL_1727160

EPI_ISL_2548172

EPI_ISL_1726497

EPI_ISL_1726498

EPI_ISL_1726499

EPI_ISL_1726502

EPI_ISL_1726503

EPI_ISL_2548173

EPI_ISL_2548174

EPI_ISL_1726504

EPI_ISL_1726508

EPI_ISL_1726509

EPI_ISL_1726510

EPI_ISL_1726511

EPI_ISL_1726512

EPI_ISL_1726513

EPI_ISL_1726515

EPI_ISL_1726516

EPI_ISL_1726517

EPI_ISL_1726519

EPI_ISL_1726520

EPI_ISL_1726521

EPI_ISL_1726522

EPI_ISL_1726524

EPI_ISL_1726525

EPI_ISL_2548175

EPI_ISL_2548176

EPI_ISL_1726526

EPI_ISL_1726527

EPI_ISL_1726528

EPI_ISL_1726529

EPI_ISL_1726530

EPI_ISL_1726531

EPI_ISL_1726532

EPI_ISL_1726533

EPI_ISL_1726535

EPI_ISL_1726537

EPI_ISL_1727161

EPI_ISL_1726539

EPI_ISL_1726540

EPI_ISL_1726541

EPI_ISL_1726542

EPI_ISL_1726544

EPI_ISL_1726546

EPI_ISL_1726547

EPI_ISL_1726548

EPI_ISL_1726550

EPI_ISL_1726551

EPI_ISL_1726553

EPI_ISL_1726554

EPI_ISL_1726556

EPI_ISL_1726557

EPI_ISL_1726561

EPI_ISL_1726563

EPI_ISL_1726565

EPI_ISL_1726566

EPI_ISL_1726570

EPI_ISL_1727162

EPI_ISL_1727163

EPI_ISL_1727164

EPI_ISL_1727165

EPI_ISL_1726572

EPI_ISL_1726573

EPI_ISL_1727166

EPI_ISL_1727167

EPI_ISL_1727168

EPI_ISL_1727169

EPI_ISL_1727170

EPI_ISL_1727171

EPI_ISL_2548177

EPI_ISL_2548178

EPI_ISL_1726580

EPI_ISL_1726581

EPI_ISL_2548179

EPI_ISL_1726582

EPI_ISL_2548180

EPI_ISL_1726583

EPI_ISL_2800155

EPI_ISL_1726584

EPI_ISL_2548182

EPI_ISL_1726587

EPI_ISL_1726588

EPI_ISL_2548183

EPI_ISL_1726590

EPI_ISL_1726591

EPI_ISL_1726592

EPI_ISL_1726593

EPI_ISL_1727172

EPI_ISL_1727173

EPI_ISL_1726597

EPI_ISL_1726598

EPI_ISL_1726599

EPI_ISL_1726601

EPI_ISL_1727174

EPI_ISL_2548184

EPI_ISL_1726602

EPI_ISL_2548185

EPI_ISL_1726604

EPI_ISL_1726606

EPI_ISL_1726607

EPI_ISL_1726609

EPI_ISL_1726610

EPI_ISL_1726611

EPI_ISL_1726612

EPI_ISL_1726614

EPI_ISL_1726615

EPI_ISL_1726618

EPI_ISL_1726619

EPI_ISL_2548187

EPI_ISL_1726623

EPI_ISL_1726624

EPI_ISL_1727175

EPI_ISL_1727177

EPI_ISL_1727178

EPI_ISL_1727179

EPI_ISL_1726631

EPI_ISL_1726632

EPI_ISL_1726634

EPI_ISL_1726635

EPI_ISL_1726636

EPI_ISL_1726637

EPI_ISL_1726638

EPI_ISL_2548188

EPI_ISL_1726641

EPI_ISL_1726642

EPI_ISL_2548189

EPI_ISL_1726644

EPI_ISL_1726646

EPI_ISL_1726647

EPI_ISL_1726648

EPI_ISL_1726651

EPI_ISL_2548190

EPI_ISL_1726652

EPI_ISL_1726654

EPI_ISL_1726655

EPI_ISL_1726656

EPI_ISL_1726660

EPI_ISL_1726661

EPI_ISL_1726662

EPI_ISL_1727180

EPI_ISL_1727181

EPI_ISL_1727182

EPI_ISL_1726665

EPI_ISL_1726666

EPI_ISL_1726669

EPI_ISL_1726671

EPI_ISL_1726672

EPI_ISL_1727184

EPI_ISL_1727185

EPI_ISL_1727186

EPI_ISL_1727187

EPI_ISL_1726675

EPI_ISL_1726676

EPI_ISL_2548192

EPI_ISL_1726678

EPI_ISL_1726679

EPI_ISL_1726680

EPI_ISL_1726682

EPI_ISL_1726683

EPI_ISL_2548193

EPI_ISL_1726684

EPI_ISL_1726685

EPI_ISL_3598385

EPI_ISL_1726686

EPI_ISL_1726687

EPI_ISL_1726688

EPI_ISL_1726690

EPI_ISL_2548195

EPI_ISL_2548196

EPI_ISL_2548197

EPI_ISL_2548198

EPI_ISL_1726691

EPI_ISL_1726692

EPI_ISL_1726693

EPI_ISL_1726694

EPI_ISL_3598492

EPI_ISL_2548201

EPI_ISL_1726695

EPI_ISL_3598370

EPI_ISL_1726697

EPI_ISL_1726698

EPI_ISL_2548203

EPI_ISL_2548204

EPI_ISL_2548205

EPI_ISL_1726699

EPI_ISL_1726700

EPI_ISL_2548208

EPI_ISL_1726702

EPI_ISL_2548209

EPI_ISL_2548210

EPI_ISL_2548211

EPI_ISL_1726703

EPI_ISL_1726704

EPI_ISL_1726705

EPI_ISL_1726706

EPI_ISL_1726708

EPI_ISL_1726709

EPI_ISL_1726711

EPI_ISL_1726713

EPI_ISL_1726715

EPI_ISL_1726716

EPI_ISL_1726717

EPI_ISL_1726718

EPI_ISL_1726720

EPI_ISL_1726721

EPI_ISL_1726723

EPI_ISL_1726725

EPI_ISL_1726726

EPI_ISL_1726727

EPI_ISL_1726728

EPI_ISL_1726730

EPI_ISL_1726732

EPI_ISL_1726733

EPI_ISL_1726735

EPI_ISL_1726737

EPI_ISL_1726739

EPI_ISL_1726742

EPI_ISL_1726744

EPI_ISL_1726747

EPI_ISL_1726748

EPI_ISL_1726750

EPI_ISL_1726751

EPI_ISL_1726752

EPI_ISL_1726753

EPI_ISL_1726754

EPI_ISL_1726755

EPI_ISL_1726757

EPI_ISL_1726758

EPI_ISL_1726759

EPI_ISL_1726760

EPI_ISL_1726761

EPI_ISL_1726762

EPI_ISL_1726763

EPI_ISL_1726766

EPI_ISL_1726767

EPI_ISL_1726768

EPI_ISL_1726769

EPI_ISL_1726770

EPI_ISL_1726771

EPI_ISL_1726772

EPI_ISL_1726774

EPI_ISL_1726778

EPI_ISL_1726781

EPI_ISL_1726782

EPI_ISL_1726783

EPI_ISL_1726784

EPI_ISL_1726786

EPI_ISL_1727189

EPI_ISL_1727190

EPI_ISL_1727191

EPI_ISL_1727192

EPI_ISL_1727193

EPI_ISL_1727194

EPI_ISL_1727196

EPI_ISL_2548214

EPI_ISL_2548215

EPI_ISL_1726788

EPI_ISL_1726789

EPI_ISL_1726790

EPI_ISL_1726792

EPI_ISL_1726793

EPI_ISL_2548216

EPI_ISL_1726795

EPI_ISL_1726797

EPI_ISL_1726799

EPI_ISL_1726801

EPI_ISL_1726802

EPI_ISL_1726804

EPI_ISL_1726805

EPI_ISL_1726806

EPI_ISL_2548218

EPI_ISL_2548219

EPI_ISL_2548220

EPI_ISL_2548221

EPI_ISL_1726809

EPI_ISL_1726811

EPI_ISL_1726812

EPI_ISL_1726815

EPI_ISL_1726817

EPI_ISL_1726818

EPI_ISL_2548223

EPI_ISL_1726820

EPI_ISL_2548224

EPI_ISL_1726822

EPI_ISL_1726826

EPI_ISL_1726827

EPI_ISL_1726830

EPI_ISL_1726831

EPI_ISL_1726832

EPI_ISL_2548225

EPI_ISL_1726834

EPI_ISL_1726835

EPI_ISL_2548227

EPI_ISL_1726839

EPI_ISL_1726842

EPI_ISL_1726844

EPI_ISL_1726845

EPI_ISL_2548228

EPI_ISL_1726846

EPI_ISL_2548230

EPI_ISL_2548231

EPI_ISL_2548233

EPI_ISL_2548234

EPI_ISL_1726856

EPI_ISL_2548235

EPI_ISL_2548236

EPI_ISL_2548237

EPI_ISL_2548238

EPI_ISL_1726857

EPI_ISL_1726858

EPI_ISL_2548240

EPI_ISL_2548241

EPI_ISL_1726865

EPI_ISL_2548245

EPI_ISL_1726870

EPI_ISL_2548246

EPI_ISL_2548247

EPI_ISL_1726871

EPI_ISL_2548249

EPI_ISL_2548250

EPI_ISL_1726875

EPI_ISL_1726876

EPI_ISL_2548251

EPI_ISL_1726884

EPI_ISL_1726892

EPI_ISL_2548254

EPI_ISL_2548255

EPI_ISL_2548256

EPI_ISL_1726897

EPI_ISL_1726898

EPI_ISL_1726899

EPI_ISL_1726903

EPI_ISL_2548258

EPI_ISL_2548259

EPI_ISL_2548260

EPI_ISL_3598662

EPI_ISL_2548261

EPI_ISL_2548262

EPI_ISL_2548263

EPI_ISL_2548264

EPI_ISL_2548265

EPI_ISL_1726917

EPI_ISL_2548267

EPI_ISL_2548268

EPI_ISL_2548269

EPI_ISL_2548270

EPI_ISL_1726923

EPI_ISL_1726924

EPI_ISL_1726926

EPI_ISL_2548271

EPI_ISL_2548272

EPI_ISL_2548273

EPI_ISL_2548274

EPI_ISL_1726927

EPI_ISL_2548275

EPI_ISL_1726928

EPI_ISL_1726929

EPI_ISL_2548276

EPI_ISL_2548277

EPI_ISL_2548278

EPI_ISL_2548279

EPI_ISL_2548280

EPI_ISL_1726931

EPI_ISL_1726932

EPI_ISL_1726934

EPI_ISL_2548282

EPI_ISL_1726935

EPI_ISL_2548283

EPI_ISL_1726936

EPI_ISL_1726937

EPI_ISL_1726938

EPI_ISL_2548284

EPI_ISL_2548285

EPI_ISL_2548286

EPI_ISL_1726940

EPI_ISL_1726942

EPI_ISL_1726943

EPI_ISL_1726944

EPI_ISL_1726945

EPI_ISL_1726946

EPI_ISL_1726947

EPI_ISL_1726948

EPI_ISL_1726949

EPI_ISL_1726950

EPI_ISL_1726951

EPI_ISL_1726952

EPI_ISL_1726953

EPI_ISL_1726954

EPI_ISL_1726955

EPI_ISL_1726957

EPI_ISL_1726958

EPI_ISL_1726959

EPI_ISL_1726960

EPI_ISL_1726961

EPI_ISL_1726962

EPI_ISL_1726963

EPI_ISL_1726964

EPI_ISL_1726965

EPI_ISL_1726966

EPI_ISL_1726967

EPI_ISL_1726969

EPI_ISL_1726971

EPI_ISL_1726973

EPI_ISL_1726975

EPI_ISL_1726976

EPI_ISL_1726977

EPI_ISL_1726978

EPI_ISL_1726979

EPI_ISL_1726980

EPI_ISL_1726982

EPI_ISL_1726983

EPI_ISL_1726984

EPI_ISL_1726985

EPI_ISL_1726986

EPI_ISL_1726988

EPI_ISL_1726989

EPI_ISL_1726990

EPI_ISL_1726991

EPI_ISL_1726992

EPI_ISL_1726993

EPI_ISL_1726994

EPI_ISL_1726995

EPI_ISL_1726996

EPI_ISL_1726997

EPI_ISL_1726998

EPI_ISL_1727000

EPI_ISL_1727001

EPI_ISL_1727002

EPI_ISL_1727003

EPI_ISL_1727005

EPI_ISL_1727006

EPI_ISL_1727007

EPI_ISL_1727008

EPI_ISL_1727009

EPI_ISL_1727010

EPI_ISL_1727011

EPI_ISL_1727012

EPI_ISL_1727013

EPI_ISL_1727014

EPI_ISL_1727015

EPI_ISL_1727016

EPI_ISL_1727017

EPI_ISL_1727018

EPI_ISL_1727019

EPI_ISL_1727021

EPI_ISL_1727022

EPI_ISL_1727023

EPI_ISL_1727024

EPI_ISL_1727026

EPI_ISL_1727029

EPI_ISL_1727030

EPI_ISL_1727031

EPI_ISL_1727032

EPI_ISL_1727033

EPI_ISL_1727034

EPI_ISL_1727036

EPI_ISL_1727037

EPI_ISL_1727038

EPI_ISL_1727039

EPI_ISL_1727040

EPI_ISL_1727041

EPI_ISL_1727042

EPI_ISL_1727043

EPI_ISL_1727044

EPI_ISL_1727045

EPI_ISL_1727046

EPI_ISL_1727048

EPI_ISL_1727050

EPI_ISL_1727051

EPI_ISL_1727052

EPI_ISL_1727053

EPI_ISL_1727054

EPI_ISL_1727055

EPI_ISL_1727056

EPI_ISL_1727057

EPI_ISL_1727059

EPI_ISL_1727061

EPI_ISL_1727062

EPI_ISL_1727063

EPI_ISL_1727064

EPI_ISL_1727065

EPI_ISL_1727066

EPI_ISL_1727068

EPI_ISL_1727072

EPI_ISL_1727075

EPI_ISL_1727077

EPI_ISL_1727081

EPI_ISL_3598433

EPI_ISL_1727090

EPI_ISL_1727093

EPI_ISL_1727094

EPI_ISL_1727095

EPI_ISL_1727097

EPI_ISL_1727098

EPI_ISL_1727099

EPI_ISL_1727101

EPI_ISL_1727102

EPI_ISL_1727104

EPI_ISL_1727105

EPI_ISL_1727106

EPI_ISL_1727107

EPI_ISL_1727108

EPI_ISL_1727109

EPI_ISL_1727110

EPI_ISL_1727112

EPI_ISL_1727114

EPI_ISL_1727115

EPI_ISL_1727116

EPI_ISL_1727118

EPI_ISL_1727119

EPI_ISL_1727198

EPI_ISL_1727199

EPI_ISL_1727200

EPI_ISL_1727201

EPI_ISL_2179054

EPI_ISL_1727202

EPI_ISL_1727122

EPI_ISL_1727125

EPI_ISL_1727129

EPI_ISL_1727131

EPI_ISL_1727132

EPI_ISL_1727134

EPI_ISL_1727136

EPI_ISL_1727137

EPI_ISL_1727138

EPI_ISL_1727139

EPI_ISL_1727204

EPI_ISL_1727205

EPI_ISL_1727206

EPI_ISL_1727207

EPI_ISL_1727208

EPI_ISL_1727209

EPI_ISL_1727210

EPI_ISL_1727211

EPI_ISL_1727212

EPI_ISL_1727213

EPI_ISL_1727215

EPI_ISL_1727216

EPI_ISL_1727689

EPI_ISL_1727690

EPI_ISL_1727218

EPI_ISL_1727219

EPI_ISL_1727220

EPI_ISL_1727691

EPI_ISL_1727221

EPI_ISL_1727222

EPI_ISL_1727224

EPI_ISL_1727225

EPI_ISL_1727226

EPI_ISL_1727723

EPI_ISL_1727692

EPI_ISL_1727228

EPI_ISL_2681408

EPI_ISL_1727229

EPI_ISL_1727230

EPI_ISL_1727693

EPI_ISL_1727231

EPI_ISL_1727232

EPI_ISL_1727233

EPI_ISL_1727234

EPI_ISL_1727235

EPI_ISL_1727236

EPI_ISL_1727237

EPI_ISL_1727238

EPI_ISL_1727694

EPI_ISL_2681409

EPI_ISL_1727240

EPI_ISL_1727241

EPI_ISL_1727242

EPI_ISL_1727724

EPI_ISL_1727243

EPI_ISL_1727244

EPI_ISL_1727245

EPI_ISL_1727725

EPI_ISL_1727246

EPI_ISL_1727248

EPI_ISL_1727249

EPI_ISL_1727250

EPI_ISL_1727251

EPI_ISL_1727252

EPI_ISL_1727254

EPI_ISL_1727257

EPI_ISL_1727258

EPI_ISL_1727259

EPI_ISL_1727260

EPI_ISL_2681410

EPI_ISL_1727261

EPI_ISL_3598485

EPI_ISL_3568437

EPI_ISL_3568438

EPI_ISL_1727262

EPI_ISL_1727263

EPI_ISL_1727264

EPI_ISL_1727265

EPI_ISL_1727266

EPI_ISL_1727268

EPI_ISL_1727269

EPI_ISL_1727270

EPI_ISL_1727271

EPI_ISL_1727272

EPI_ISL_1727273

EPI_ISL_1727275

EPI_ISL_1727276

EPI_ISL_1727277

EPI_ISL_1727278

EPI_ISL_1727280

EPI_ISL_1727281

EPI_ISL_1727282

EPI_ISL_1727283

EPI_ISL_1727284

EPI_ISL_1727285

EPI_ISL_1727286

EPI_ISL_1727287

EPI_ISL_1727288

EPI_ISL_1727289

EPI_ISL_1727290

EPI_ISL_1727291

EPI_ISL_1727292

EPI_ISL_1727294

EPI_ISL_1727295

EPI_ISL_1727296

EPI_ISL_1727297

EPI_ISL_1727298

EPI_ISL_1727299

EPI_ISL_1727696

EPI_ISL_1727300

EPI_ISL_1727301

EPI_ISL_1727302

EPI_ISL_1727303

EPI_ISL_1727304

EPI_ISL_1727305

EPI_ISL_3598472

EPI_ISL_1727307

EPI_ISL_1727308

EPI_ISL_1727310

EPI_ISL_1727311

EPI_ISL_1727312

EPI_ISL_3568743

EPI_ISL_3568745

EPI_ISL_3568747

EPI_ISL_3568748

EPI_ISL_3568749

EPI_ISL_3568750

EPI_ISL_3568751

EPI_ISL_1727698

EPI_ISL_3568752

EPI_ISL_3568753

EPI_ISL_3568754

EPI_ISL_3568755

EPI_ISL_3568756

EPI_ISL_1727314

EPI_ISL_1727315

EPI_ISL_5417772

EPI_ISL_1727320

EPI_ISL_1727321

EPI_ISL_1727322

EPI_ISL_1727323

EPI_ISL_1727324

EPI_ISL_5417790

EPI_ISL_1727325

EPI_ISL_1727326

EPI_ISL_1727327

EPI_ISL_1727328

EPI_ISL_1727329

EPI_ISL_1727330

EPI_ISL_1727331

EPI_ISL_1727332

EPI_ISL_1727333

EPI_ISL_1727334

EPI_ISL_1727335

EPI_ISL_1727336

EPI_ISL_1727337

EPI_ISL_1727338

EPI_ISL_1727339

EPI_ISL_1727340

EPI_ISL_1727341

EPI_ISL_1727342

EPI_ISL_1727343

EPI_ISL_1727344

EPI_ISL_1727345

EPI_ISL_1727346

EPI_ISL_1727347

EPI_ISL_1727348

EPI_ISL_1727349

EPI_ISL_1727350

EPI_ISL_1727351

EPI_ISL_1727352

EPI_ISL_1727353

EPI_ISL_1727354

EPI_ISL_1727355

EPI_ISL_1727356

EPI_ISL_1727357

EPI_ISL_1727358

EPI_ISL_1727359

EPI_ISL_5417922

EPI_ISL_1727360

EPI_ISL_1727361

EPI_ISL_5417932

EPI_ISL_1727363

EPI_ISL_5417946

EPI_ISL_1727364

EPI_ISL_5417947

EPI_ISL_1727365

EPI_ISL_1727366

EPI_ISL_1727367

EPI_ISL_1727368

EPI_ISL_5417958

EPI_ISL_5417962

EPI_ISL_1727700

EPI_ISL_1727371

EPI_ISL_1727372

EPI_ISL_1727373

EPI_ISL_1727375

EPI_ISL_1727376

EPI_ISL_1727377

EPI_ISL_1727379

EPI_ISL_1727380

EPI_ISL_1727381

EPI_ISL_1727382

EPI_ISL_1727383

EPI_ISL_1727384

EPI_ISL_1727385

EPI_ISL_1727386

EPI_ISL_1727701

EPI_ISL_1727702

EPI_ISL_1727387

EPI_ISL_1727388

EPI_ISL_1727389

EPI_ISL_1727390

EPI_ISL_1727391

EPI_ISL_1727392

EPI_ISL_1727393

EPI_ISL_1727394

EPI_ISL_1727395

EPI_ISL_1727397

EPI_ISL_1727398

EPI_ISL_1727399

EPI_ISL_1727400

EPI_ISL_1727402

EPI_ISL_1727403

EPI_ISL_1727404

EPI_ISL_1727405

EPI_ISL_1727406

EPI_ISL_1727407

EPI_ISL_1727408

EPI_ISL_1727409

EPI_ISL_1727410

EPI_ISL_1727411

EPI_ISL_1727413

EPI_ISL_1727414

EPI_ISL_1727416

EPI_ISL_1727417

EPI_ISL_1727418

EPI_ISL_1727419

EPI_ISL_1727420

EPI_ISL_1727421

EPI_ISL_1727422

EPI_ISL_3598931

EPI_ISL_1727424

EPI_ISL_1727425

EPI_ISL_1727426

EPI_ISL_1727428

EPI_ISL_1727429

EPI_ISL_1727430

EPI_ISL_1727431

EPI_ISL_1727432

EPI_ISL_1727434

EPI_ISL_1727435

EPI_ISL_1727436

EPI_ISL_1727437

EPI_ISL_1727439

EPI_ISL_1727440

EPI_ISL_1727442

EPI_ISL_1727443

EPI_ISL_1727444

EPI_ISL_3598601

EPI_ISL_1727445

EPI_ISL_1727447

EPI_ISL_1727448

EPI_ISL_1727449

EPI_ISL_1727450

EPI_ISL_1727452

EPI_ISL_1727453

EPI_ISL_1727454

EPI_ISL_1727457

EPI_ISL_1727461

EPI_ISL_1727462

EPI_ISL_1727465

EPI_ISL_1727468

EPI_ISL_1727469

EPI_ISL_1727470

EPI_ISL_1727703

EPI_ISL_1727704

EPI_ISL_1727705

EPI_ISL_1727706

EPI_ISL_1727707

EPI_ISL_1727708

EPI_ISL_1727709

EPI_ISL_1727710

EPI_ISL_1727473

EPI_ISL_1727474

EPI_ISL_1727475

EPI_ISL_1727480

EPI_ISL_1727481

EPI_ISL_1727483

EPI_ISL_1727484

EPI_ISL_1727485

EPI_ISL_1727486

EPI_ISL_1727487

EPI_ISL_1727488

EPI_ISL_1727489

EPI_ISL_1727490

EPI_ISL_1727491

EPI_ISL_1727493

EPI_ISL_1727494

EPI_ISL_1727495

EPI_ISL_1727496

EPI_ISL_1727498

EPI_ISL_1727499

EPI_ISL_1727500

EPI_ISL_1727501

EPI_ISL_1727502

EPI_ISL_1727503

EPI_ISL_1727506

EPI_ISL_1727507

EPI_ISL_1727508

EPI_ISL_1727509

EPI_ISL_1727510

EPI_ISL_1727511

EPI_ISL_1727512

EPI_ISL_1727513

EPI_ISL_1727514

EPI_ISL_1727516

EPI_ISL_1727517

EPI_ISL_1727518

EPI_ISL_1727519

EPI_ISL_1727520

EPI_ISL_1727521

EPI_ISL_1727522

EPI_ISL_1727523

EPI_ISL_1727524

EPI_ISL_1727525

EPI_ISL_1727526

EPI_ISL_1727528

EPI_ISL_1727529

EPI_ISL_1727530

EPI_ISL_1727711

EPI_ISL_1727712

EPI_ISL_1727713

EPI_ISL_1727714

EPI_ISL_1727715

EPI_ISL_1727532

EPI_ISL_1727533

EPI_ISL_1727534

EPI_ISL_1727535

EPI_ISL_1727536

EPI_ISL_1727537

EPI_ISL_1727538

EPI_ISL_1727539

EPI_ISL_1727540

EPI_ISL_1727541

EPI_ISL_1727542

EPI_ISL_1727543

EPI_ISL_1727544

EPI_ISL_1727546

EPI_ISL_1727716

EPI_ISL_1727547

EPI_ISL_1727548

EPI_ISL_1727549

EPI_ISL_1727550

EPI_ISL_1727551

EPI_ISL_1727717

EPI_ISL_1727552

EPI_ISL_1727553

EPI_ISL_1727555

EPI_ISL_1727556

EPI_ISL_1727718

EPI_ISL_1727557

EPI_ISL_1727558

EPI_ISL_1727719

EPI_ISL_1727559

EPI_ISL_1727560

EPI_ISL_1727561

EPI_ISL_1727562

EPI_ISL_1727563

EPI_ISL_1727564

EPI_ISL_1727565

EPI_ISL_1727569

EPI_ISL_1727570

EPI_ISL_1727573

EPI_ISL_1727574

EPI_ISL_1727576

EPI_ISL_1727577

EPI_ISL_1727578

EPI_ISL_1727579

EPI_ISL_1727580

EPI_ISL_1727581

EPI_ISL_1727582

EPI_ISL_1727584

EPI_ISL_1727585

EPI_ISL_1727586

EPI_ISL_1727587

EPI_ISL_1727588

EPI_ISL_1727589

EPI_ISL_1727590

EPI_ISL_1727591

EPI_ISL_1727592

EPI_ISL_1727593

EPI_ISL_1727594

EPI_ISL_1727595

EPI_ISL_1727596

EPI_ISL_1727597

EPI_ISL_1727598

EPI_ISL_1727599

EPI_ISL_1727601

EPI_ISL_1727603

EPI_ISL_1727604

EPI_ISL_1727605

EPI_ISL_1727606

EPI_ISL_1727607

EPI_ISL_1727608

EPI_ISL_1727609

EPI_ISL_1727610

EPI_ISL_1727611

EPI_ISL_1727612

EPI_ISL_1727613

EPI_ISL_1727614

EPI_ISL_1727615

EPI_ISL_1727616

EPI_ISL_1727617

EPI_ISL_1727618

EPI_ISL_2681394

EPI_ISL_1727619

EPI_ISL_1727620

EPI_ISL_1727621

EPI_ISL_1727623

EPI_ISL_1727624

EPI_ISL_1727625

EPI_ISL_1727626

EPI_ISL_1727627

EPI_ISL_1727628

EPI_ISL_1727629

EPI_ISL_1727631

EPI_ISL_1727632

EPI_ISL_1727633

EPI_ISL_1727634

EPI_ISL_1727635

EPI_ISL_1727636

EPI_ISL_1727637

EPI_ISL_1727638

EPI_ISL_1727639

EPI_ISL_1727640

EPI_ISL_2681395

EPI_ISL_1727641

EPI_ISL_1727642

EPI_ISL_1727643

EPI_ISL_1727644

EPI_ISL_1727645

EPI_ISL_1727646

EPI_ISL_1727648

EPI_ISL_1727649

EPI_ISL_1727650

EPI_ISL_1727651

EPI_ISL_1727652

EPI_ISL_2681397

EPI_ISL_1727653

EPI_ISL_1727654

EPI_ISL_1727655

EPI_ISL_1727656

EPI_ISL_1727658

EPI_ISL_1727659

EPI_ISL_1727660

EPI_ISL_1727661

EPI_ISL_1727662

EPI_ISL_1727663

EPI_ISL_1727664

EPI_ISL_1727665

EPI_ISL_1727666

EPI_ISL_1727667

EPI_ISL_1727669

EPI_ISL_1727670

EPI_ISL_1727671

EPI_ISL_1727672

EPI_ISL_1727673

EPI_ISL_1727674

EPI_ISL_1727675

EPI_ISL_2681399

EPI_ISL_1727676

EPI_ISL_1727677

EPI_ISL_1727679

EPI_ISL_1727722

EPI_ISL_2681400

EPI_ISL_2681401

EPI_ISL_2681403

EPI_ISL_1727680

EPI_ISL_2681404

EPI_ISL_2681405

EPI_ISL_1727681

EPI_ISL_2681407

EPI_ISL_1727682

EPI_ISL_1727683

EPI_ISL_1727684

EPI_ISL_1727685

EPI_ISL_1727687

EPI_ISL_1727688

EPI_ISL_1727727

EPI_ISL_1727728

EPI_ISL_1727730

EPI_ISL_1727731

EPI_ISL_1727732

EPI_ISL_1727733

EPI_ISL_1727734

EPI_ISL_1727735

EPI_ISL_1727736

EPI_ISL_1727737

EPI_ISL_1727738

EPI_ISL_1727739

EPI_ISL_1727740

EPI_ISL_1727741

EPI_ISL_1727742

EPI_ISL_1727743

EPI_ISL_2681417

EPI_ISL_2681418

EPI_ISL_1727745

EPI_ISL_1727746

EPI_ISL_1727747

EPI_ISL_2681421

EPI_ISL_1727748

EPI_ISL_1727750

EPI_ISL_1728365

EPI_ISL_2681423

EPI_ISL_2681425

EPI_ISL_2681426

EPI_ISL_2681427

EPI_ISL_1727752

EPI_ISL_1727753

EPI_ISL_1727754

EPI_ISL_1727755

EPI_ISL_1727756

EPI_ISL_1727758

EPI_ISL_1727759

EPI_ISL_1727760

EPI_ISL_1727761

EPI_ISL_1727762

EPI_ISL_1728366

EPI_ISL_1728367

EPI_ISL_1728368

EPI_ISL_1728369

EPI_ISL_1728370

EPI_ISL_2681432

EPI_ISL_2681433

EPI_ISL_3598991

EPI_ISL_2681436

EPI_ISL_1727765

EPI_ISL_1727767

EPI_ISL_1727768

EPI_ISL_1727769

EPI_ISL_1727770

EPI_ISL_1727771

EPI_ISL_1727774

EPI_ISL_1727775

EPI_ISL_1727776

EPI_ISL_1727777

EPI_ISL_1727778

EPI_ISL_1727780

EPI_ISL_1727781

EPI_ISL_1727782

EPI_ISL_1727783

EPI_ISL_1727784

EPI_ISL_1727785

EPI_ISL_1727786

EPI_ISL_1727787

EPI_ISL_1727788

EPI_ISL_1727789

EPI_ISL_1727791

EPI_ISL_1727792

EPI_ISL_1727793

EPI_ISL_1727794

EPI_ISL_1727795

EPI_ISL_1727796

EPI_ISL_1727797

EPI_ISL_1727798

EPI_ISL_1727799

EPI_ISL_1727800

EPI_ISL_1727801

EPI_ISL_1727802

EPI_ISL_1727803

EPI_ISL_1727804

EPI_ISL_1727807

EPI_ISL_1727808

EPI_ISL_1727809

EPI_ISL_1727810

EPI_ISL_1727811

EPI_ISL_1727812

EPI_ISL_2681440

EPI_ISL_1727815

EPI_ISL_1727816

EPI_ISL_1727817

EPI_ISL_1727818

EPI_ISL_1727819

EPI_ISL_1727820

EPI_ISL_1727821

EPI_ISL_1727822

EPI_ISL_1727824

EPI_ISL_1727825

EPI_ISL_1727826

EPI_ISL_1727827

EPI_ISL_1727828

EPI_ISL_1727829

EPI_ISL_1727830

EPI_ISL_1727832

EPI_ISL_1727833

EPI_ISL_1727834

EPI_ISL_1727835

EPI_ISL_1727837

EPI_ISL_1727838

EPI_ISL_1727839

EPI_ISL_1727840

EPI_ISL_1727841

EPI_ISL_1727842

EPI_ISL_1727843

EPI_ISL_1727844

EPI_ISL_1727845

EPI_ISL_1727846

EPI_ISL_1727847

EPI_ISL_1727848

EPI_ISL_1727849

EPI_ISL_1727851

EPI_ISL_1727852

EPI_ISL_1727853

EPI_ISL_1727854

EPI_ISL_1727856

EPI_ISL_1727857

EPI_ISL_1727858

EPI_ISL_1727859

EPI_ISL_1727860

EPI_ISL_1727861

EPI_ISL_1727862

EPI_ISL_1727863

EPI_ISL_1727864

EPI_ISL_1727865

EPI_ISL_1727866

EPI_ISL_1727867

EPI_ISL_1727868

EPI_ISL_1727869

EPI_ISL_1727870

EPI_ISL_1727872

EPI_ISL_1727874

EPI_ISL_1727875

EPI_ISL_1727876

EPI_ISL_1727878

EPI_ISL_1727879

EPI_ISL_1727880

EPI_ISL_1727881

EPI_ISL_1727882

EPI_ISL_1727883

EPI_ISL_1727884

EPI_ISL_1727887

EPI_ISL_1727888

EPI_ISL_1727889

EPI_ISL_1727890

EPI_ISL_1727891

EPI_ISL_1727892

EPI_ISL_1727893

EPI_ISL_1727894

EPI_ISL_1727897

EPI_ISL_1727898

EPI_ISL_1727899

EPI_ISL_1727900

EPI_ISL_1727901

EPI_ISL_1727902

EPI_ISL_1727903

EPI_ISL_1727904

EPI_ISL_1727905

EPI_ISL_1727906

EPI_ISL_1727907

EPI_ISL_1727908

EPI_ISL_1727909

EPI_ISL_1727910

EPI_ISL_1727911

EPI_ISL_1727913

EPI_ISL_1727914

EPI_ISL_1727915

EPI_ISL_1727916

EPI_ISL_1727917

EPI_ISL_1727919

EPI_ISL_1727920

EPI_ISL_1727921

EPI_ISL_1727922

EPI_ISL_1727923

EPI_ISL_1727924

EPI_ISL_1727925

EPI_ISL_1727926

EPI_ISL_1727928

EPI_ISL_2548634

EPI_ISL_1727929

EPI_ISL_1727930

EPI_ISL_1727932

EPI_ISL_1727933

EPI_ISL_1727934

EPI_ISL_1727935

EPI_ISL_1727936

EPI_ISL_1728378

EPI_ISL_1728379

EPI_ISL_1728380

EPI_ISL_1728381

EPI_ISL_1728382

EPI_ISL_1728383

EPI_ISL_1727938

EPI_ISL_1727939

EPI_ISL_1727940

EPI_ISL_1727942

EPI_ISL_1727943

EPI_ISL_1728384

EPI_ISL_1727945

EPI_ISL_1727947

EPI_ISL_1727948

EPI_ISL_1727949

EPI_ISL_1727950

EPI_ISL_1727951

EPI_ISL_3570053

EPI_ISL_3570062

EPI_ISL_3570068

EPI_ISL_3570071

EPI_ISL_1727952

EPI_ISL_3570079

EPI_ISL_1727954

EPI_ISL_1727955

EPI_ISL_1727956

EPI_ISL_1727958

EPI_ISL_1727959

EPI_ISL_1727960

EPI_ISL_1727961

EPI_ISL_1727962

EPI_ISL_2548679

EPI_ISL_1727964

EPI_ISL_1727965

EPI_ISL_2548680

EPI_ISL_1727966

EPI_ISL_1727967

EPI_ISL_1727969

EPI_ISL_1727971

EPI_ISL_1727972

EPI_ISL_1728386

EPI_ISL_1727974

EPI_ISL_1728387

EPI_ISL_1728388

EPI_ISL_1728389

EPI_ISL_1728390

EPI_ISL_1728391

EPI_ISL_2548685

EPI_ISL_1728392

EPI_ISL_1728394

EPI_ISL_1727977

EPI_ISL_1727978

EPI_ISL_2548686

EPI_ISL_1727979

EPI_ISL_1727980

EPI_ISL_1727981

EPI_ISL_1727982

EPI_ISL_1727983

EPI_ISL_1727984

EPI_ISL_1727985

EPI_ISL_1727986

EPI_ISL_1727987

EPI_ISL_1727988

EPI_ISL_1727989

EPI_ISL_1727990

EPI_ISL_1727992

EPI_ISL_1727993

EPI_ISL_1727995

EPI_ISL_1727996

EPI_ISL_1727997

EPI_ISL_1727998

EPI_ISL_1728000

EPI_ISL_1728001

EPI_ISL_1728002

EPI_ISL_1728003

EPI_ISL_1728004

EPI_ISL_1728005

EPI_ISL_1728006

EPI_ISL_1728007

EPI_ISL_1728008

EPI_ISL_1728009

EPI_ISL_1728010

EPI_ISL_1728011

EPI_ISL_1728012

EPI_ISL_1728013

EPI_ISL_1728014

EPI_ISL_1728015

EPI_ISL_1728016

EPI_ISL_1728017

EPI_ISL_1728018

EPI_ISL_1728019

EPI_ISL_1728020

EPI_ISL_1728021

EPI_ISL_1728024

EPI_ISL_1728025

EPI_ISL_1728026

EPI_ISL_1728027

EPI_ISL_1728029

EPI_ISL_1728395

EPI_ISL_1728396

EPI_ISL_1728397

EPI_ISL_1728031

EPI_ISL_1728032

EPI_ISL_1728034

EPI_ISL_1728035

EPI_ISL_1728036

EPI_ISL_1728037

EPI_ISL_1728038

EPI_ISL_1728039

EPI_ISL_1728040

EPI_ISL_1728041

EPI_ISL_1728043

EPI_ISL_1728044

EPI_ISL_1728045

EPI_ISL_1728047

EPI_ISL_1728048

EPI_ISL_1728049

EPI_ISL_1728050

EPI_ISL_1728051

EPI_ISL_1728052

EPI_ISL_1728053

EPI_ISL_1728054

EPI_ISL_1728055

EPI_ISL_1728056

EPI_ISL_1728057

EPI_ISL_1728058

EPI_ISL_1728060

EPI_ISL_1728061

EPI_ISL_1728062

EPI_ISL_1728063

EPI_ISL_1728064

EPI_ISL_1728065

EPI_ISL_1728066

EPI_ISL_1728067

EPI_ISL_1728068

EPI_ISL_1728069

EPI_ISL_1728070

EPI_ISL_1728071

EPI_ISL_1728072

EPI_ISL_1728074

EPI_ISL_1728075

EPI_ISL_1728077

EPI_ISL_1728398

EPI_ISL_1728399

EPI_ISL_1728080

EPI_ISL_1728081

EPI_ISL_1728082

EPI_ISL_1728083

EPI_ISL_1728085

EPI_ISL_1728086

EPI_ISL_1728087

EPI_ISL_1728088

EPI_ISL_1728089

EPI_ISL_1728090

EPI_ISL_1728091

EPI_ISL_1728092

EPI_ISL_1728095

EPI_ISL_1728096

EPI_ISL_1728097

EPI_ISL_1728098

EPI_ISL_1728099

EPI_ISL_1728100

EPI_ISL_1728101

EPI_ISL_1728102

EPI_ISL_1728104

EPI_ISL_1728105

EPI_ISL_1728106

EPI_ISL_1728107

EPI_ISL_1728108

EPI_ISL_1728109

EPI_ISL_1728110

EPI_ISL_1728111

EPI_ISL_1728112

EPI_ISL_1728400

EPI_ISL_1728115

EPI_ISL_1728116

EPI_ISL_1728118

EPI_ISL_1728119

EPI_ISL_1728120

EPI_ISL_1728121

EPI_ISL_1728122

EPI_ISL_1728123

EPI_ISL_1728124

EPI_ISL_1728125

EPI_ISL_1728126

EPI_ISL_1728127

EPI_ISL_1728128

EPI_ISL_1728129

EPI_ISL_1728130

EPI_ISL_1728131

EPI_ISL_1728132

EPI_ISL_1728133

EPI_ISL_1728134

EPI_ISL_1728135

EPI_ISL_1728136

EPI_ISL_1728137

EPI_ISL_1728138

EPI_ISL_1728139

EPI_ISL_1728141

EPI_ISL_1728142

EPI_ISL_1728145

EPI_ISL_1728146

EPI_ISL_1728147

EPI_ISL_1728148

EPI_ISL_1728149

EPI_ISL_1728150

EPI_ISL_1728154

EPI_ISL_2548777

EPI_ISL_2548778

EPI_ISL_1728155

EPI_ISL_1728156

EPI_ISL_1728157

EPI_ISL_1728158

EPI_ISL_1728159

EPI_ISL_1728160

EPI_ISL_1728161

EPI_ISL_1728162

EPI_ISL_1728163

EPI_ISL_1728164

EPI_ISL_2548783

EPI_ISL_2548784

EPI_ISL_1728165

EPI_ISL_2548785

EPI_ISL_1728166

EPI_ISL_1728167

EPI_ISL_1728169

EPI_ISL_1728170

EPI_ISL_1728171

EPI_ISL_1728172

EPI_ISL_1728173

EPI_ISL_1728175

EPI_ISL_1728176

EPI_ISL_1728177

EPI_ISL_1728178

EPI_ISL_1728179

EPI_ISL_1728180

EPI_ISL_1728181

EPI_ISL_1728182

EPI_ISL_1728183

EPI_ISL_1728185

EPI_ISL_1728187

EPI_ISL_1728188

EPI_ISL_1728189

EPI_ISL_1728190

EPI_ISL_1728193

EPI_ISL_1728194

EPI_ISL_1728195

EPI_ISL_1728196

EPI_ISL_1728197

EPI_ISL_1728198

EPI_ISL_1728199

EPI_ISL_1728200

EPI_ISL_1728202

EPI_ISL_1728203

EPI_ISL_1728204

EPI_ISL_1728205

EPI_ISL_1728206

EPI_ISL_1728207

EPI_ISL_1728210

EPI_ISL_1728211

EPI_ISL_1728212

EPI_ISL_1728213

EPI_ISL_1728216

EPI_ISL_1728217

EPI_ISL_1728218

EPI_ISL_1728219

EPI_ISL_1728220

EPI_ISL_1728221

EPI_ISL_1728222

EPI_ISL_1728223

EPI_ISL_1728224

EPI_ISL_1728225

EPI_ISL_1728226

EPI_ISL_1728227

EPI_ISL_1728228

EPI_ISL_1728230

EPI_ISL_1728231

EPI_ISL_1728232

EPI_ISL_1728233

EPI_ISL_1728234

EPI_ISL_1728235

EPI_ISL_1728236

EPI_ISL_1728238

EPI_ISL_1728239

EPI_ISL_1728240

EPI_ISL_1728241

EPI_ISL_1728242

EPI_ISL_1728243

EPI_ISL_1728244

EPI_ISL_1728245

EPI_ISL_1728246

EPI_ISL_1728247

EPI_ISL_1728248

EPI_ISL_1728250

EPI_ISL_1728251

EPI_ISL_1728252

EPI_ISL_1728253

EPI_ISL_1728254

EPI_ISL_1728255

EPI_ISL_1728256

EPI_ISL_1728257

EPI_ISL_1728258

EPI_ISL_1728259

EPI_ISL_1728260

EPI_ISL_1728262

EPI_ISL_1728265

EPI_ISL_1728266

EPI_ISL_1728267

EPI_ISL_1728268

EPI_ISL_1728269

EPI_ISL_1728270

EPI_ISL_1728271

EPI_ISL_1728272

EPI_ISL_1728273

EPI_ISL_1728274

EPI_ISL_1728275

EPI_ISL_1728276

EPI_ISL_1728277

EPI_ISL_1728278

EPI_ISL_1728279

EPI_ISL_1728280

EPI_ISL_1728281

EPI_ISL_1728282

EPI_ISL_1728283

EPI_ISL_1728285

EPI_ISL_1728286

EPI_ISL_1728287

EPI_ISL_1728288

EPI_ISL_1728290

EPI_ISL_1728291

EPI_ISL_1728292

EPI_ISL_1728293

EPI_ISL_1728294

EPI_ISL_1728295

EPI_ISL_1728296

EPI_ISL_1728297

EPI_ISL_1728299

EPI_ISL_1728300

EPI_ISL_1728303

EPI_ISL_1728305

EPI_ISL_1728306

EPI_ISL_1728308

EPI_ISL_1728309

EPI_ISL_1728312

EPI_ISL_1728313

EPI_ISL_1728318

EPI_ISL_1728319

EPI_ISL_1728320

EPI_ISL_1728321

EPI_ISL_1728322

EPI_ISL_1728323

EPI_ISL_1728324

EPI_ISL_1728325

EPI_ISL_1728326

EPI_ISL_1728327

EPI_ISL_1728328

EPI_ISL_2548825

EPI_ISL_1728330

EPI_ISL_1728332

EPI_ISL_1728334

EPI_ISL_1728335

EPI_ISL_1728336

EPI_ISL_1728337

EPI_ISL_1728339

EPI_ISL_1728340

EPI_ISL_1728341

EPI_ISL_1728342

EPI_ISL_1728343

EPI_ISL_1728344

EPI_ISL_1728373

EPI_ISL_1728374

EPI_ISL_1728375

EPI_ISL_1728376

EPI_ISL_1728346

EPI_ISL_1728348

EPI_ISL_1728349

EPI_ISL_1728350

EPI_ISL_1728351

EPI_ISL_1728352

EPI_ISL_1728353

EPI_ISL_1728354

EPI_ISL_1728356

EPI_ISL_1728357

EPI_ISL_1728358

EPI_ISL_1728360

EPI_ISL_1728361

EPI_ISL_1728362

EPI_ISL_1728364

EPI_ISL_1729248

EPI_ISL_1729250

EPI_ISL_1729251

EPI_ISL_1728402

EPI_ISL_1728403

EPI_ISL_1728404

EPI_ISL_1728405

EPI_ISL_1728406

EPI_ISL_1728407

EPI_ISL_1728408

EPI_ISL_1728409

EPI_ISL_1728410

EPI_ISL_1728411

EPI_ISL_1728412

EPI_ISL_1728413

EPI_ISL_1728414

EPI_ISL_1728415

EPI_ISL_1728416

EPI_ISL_1728417

EPI_ISL_1728418

EPI_ISL_1728419

EPI_ISL_1728420

EPI_ISL_1728421

EPI_ISL_1728423

EPI_ISL_1728425

EPI_ISL_1728426

EPI_ISL_1729253

EPI_ISL_1729254

EPI_ISL_1728428

EPI_ISL_1728429

EPI_ISL_1728431

EPI_ISL_1728432

EPI_ISL_1728433

EPI_ISL_1728434

EPI_ISL_1728435

EPI_ISL_1728437

EPI_ISL_1728438

EPI_ISL_1728439

EPI_ISL_1728440

EPI_ISL_1728442

EPI_ISL_1728445

EPI_ISL_1728446

EPI_ISL_1728447

EPI_ISL_1728448

EPI_ISL_1728451

EPI_ISL_1728452

EPI_ISL_1728453

EPI_ISL_1728454

EPI_ISL_1728455

EPI_ISL_1728456

EPI_ISL_1728457

EPI_ISL_1728458

EPI_ISL_1728460

EPI_ISL_1728461

EPI_ISL_1728462

EPI_ISL_1728463

EPI_ISL_1728464

EPI_ISL_1728465

EPI_ISL_1728467

EPI_ISL_1728468

EPI_ISL_1728469

EPI_ISL_1728470

EPI_ISL_1728471

EPI_ISL_1728472

EPI_ISL_1728473

EPI_ISL_1728474

EPI_ISL_1728475

EPI_ISL_1728476

EPI_ISL_1728477

EPI_ISL_1728478

EPI_ISL_1728479

EPI_ISL_1728480

EPI_ISL_1728481

EPI_ISL_1728482

EPI_ISL_1728483

EPI_ISL_1728484

EPI_ISL_1728485

EPI_ISL_1728486

EPI_ISL_1728487

EPI_ISL_1728488

EPI_ISL_1728489

EPI_ISL_1728491

EPI_ISL_1728492

EPI_ISL_1728493

EPI_ISL_1728494

EPI_ISL_1728496

EPI_ISL_1728497

EPI_ISL_1728498

EPI_ISL_1728499

EPI_ISL_1728500

EPI_ISL_1728501

EPI_ISL_1728503

EPI_ISL_1728504

EPI_ISL_1728505

EPI_ISL_1728506

EPI_ISL_1728507

EPI_ISL_1728509

EPI_ISL_1728510

EPI_ISL_1728511

EPI_ISL_1728513

EPI_ISL_1728514

EPI_ISL_1728515

EPI_ISL_1728517

EPI_ISL_1728518

EPI_ISL_1728519

EPI_ISL_1728520

EPI_ISL_1728521

EPI_ISL_1728522

EPI_ISL_1728523

EPI_ISL_1728524

EPI_ISL_1728526

EPI_ISL_1728527

EPI_ISL_1728528

EPI_ISL_1728529

EPI_ISL_1728530

EPI_ISL_1728531

EPI_ISL_1728532

EPI_ISL_1728534

EPI_ISL_1728535

EPI_ISL_1728536

EPI_ISL_1728537

EPI_ISL_1728538

EPI_ISL_1728539

EPI_ISL_1728541

EPI_ISL_1728542

EPI_ISL_1728543

EPI_ISL_1728544

EPI_ISL_1728545

EPI_ISL_1728546

EPI_ISL_1728547

EPI_ISL_1728548

EPI_ISL_1728549

EPI_ISL_1728550

EPI_ISL_1728551

EPI_ISL_1728552

EPI_ISL_1728553

EPI_ISL_1728554

EPI_ISL_1728555

EPI_ISL_1728556

EPI_ISL_1728557

EPI_ISL_1728559

EPI_ISL_1728561

EPI_ISL_1728562

EPI_ISL_1728563

EPI_ISL_1728564

EPI_ISL_1728565

EPI_ISL_1728566

EPI_ISL_1728568

EPI_ISL_1728569

EPI_ISL_1728570

EPI_ISL_1728571

EPI_ISL_1728573

EPI_ISL_1728574

EPI_ISL_1728575

EPI_ISL_1728576

EPI_ISL_1728577

EPI_ISL_1728578

EPI_ISL_1728579

EPI_ISL_1728580

EPI_ISL_1728581

EPI_ISL_1728583

EPI_ISL_1728584

EPI_ISL_1728585

EPI_ISL_1728586

EPI_ISL_1728587

EPI_ISL_1728588

EPI_ISL_1728590

EPI_ISL_4347721

EPI_ISL_1729256

EPI_ISL_1729257

EPI_ISL_1728732

EPI_ISL_1728865

EPI_ISL_1728866

EPI_ISL_1728867

EPI_ISL_1728868

EPI_ISL_1728869

EPI_ISL_1728870

EPI_ISL_1728871

EPI_ISL_1728872

EPI_ISL_1728873

EPI_ISL_1728874

EPI_ISL_1728875

EPI_ISL_1728876

EPI_ISL_1728878

EPI_ISL_1728880

EPI_ISL_1728881

EPI_ISL_1728882

EPI_ISL_1728883

EPI_ISL_1728884

EPI_ISL_1728885

EPI_ISL_1728887

EPI_ISL_1729259

EPI_ISL_1728889

EPI_ISL_1728890

EPI_ISL_1728891

EPI_ISL_1728892

EPI_ISL_1728893

EPI_ISL_1728894

EPI_ISL_1728896

EPI_ISL_1728897

EPI_ISL_1728898

EPI_ISL_1728899

EPI_ISL_1728900

EPI_ISL_1728901

EPI_ISL_1728902

EPI_ISL_1728903

EPI_ISL_1728904

EPI_ISL_1728905

EPI_ISL_1728906

EPI_ISL_1728907

EPI_ISL_1728908

EPI_ISL_1728909

EPI_ISL_1728910

EPI_ISL_1729260

EPI_ISL_1728913

EPI_ISL_1728914

EPI_ISL_1728915

EPI_ISL_1728916

EPI_ISL_1728917

EPI_ISL_1728919

EPI_ISL_1728920

EPI_ISL_1728921

EPI_ISL_1728922

EPI_ISL_1728923

EPI_ISL_1728924

EPI_ISL_1728925

EPI_ISL_1728926

EPI_ISL_1728927

EPI_ISL_1728928

EPI_ISL_1728929

EPI_ISL_1728930

EPI_ISL_1728931

EPI_ISL_1728932

EPI_ISL_1728933

EPI_ISL_1728934

EPI_ISL_1728935

EPI_ISL_1728936

EPI_ISL_1728937

EPI_ISL_1728938

EPI_ISL_1728939

EPI_ISL_1728940

EPI_ISL_1728942

EPI_ISL_1728943

EPI_ISL_1728944

EPI_ISL_1728945

EPI_ISL_1728946

EPI_ISL_1728947

EPI_ISL_1728948

EPI_ISL_1728949

EPI_ISL_1728950

EPI_ISL_1728951

EPI_ISL_1728952

EPI_ISL_1728953

EPI_ISL_1728954

EPI_ISL_1728955

EPI_ISL_1728956

EPI_ISL_1728957

EPI_ISL_1728959

EPI_ISL_1728960

EPI_ISL_1728962

EPI_ISL_1728963

EPI_ISL_1728965

EPI_ISL_1728966

EPI_ISL_1728967

EPI_ISL_1728969

EPI_ISL_1728970

EPI_ISL_1728971

EPI_ISL_1728972

EPI_ISL_1728973

EPI_ISL_1728974

EPI_ISL_1728975

EPI_ISL_1728977

EPI_ISL_1728978

EPI_ISL_1728979

EPI_ISL_1728981

EPI_ISL_1729261

EPI_ISL_1728982

EPI_ISL_1728983

EPI_ISL_1728984

EPI_ISL_1728985

EPI_ISL_1728987

EPI_ISL_1728988

EPI_ISL_1728989

EPI_ISL_1728990

EPI_ISL_1728991

EPI_ISL_1728992

EPI_ISL_1728994

EPI_ISL_1728995

EPI_ISL_1728996

EPI_ISL_1728997

EPI_ISL_1728998

EPI_ISL_1728999

EPI_ISL_1729000

EPI_ISL_1729002

EPI_ISL_1729003

EPI_ISL_1729005

EPI_ISL_1729006

EPI_ISL_1729007

EPI_ISL_1729008

EPI_ISL_1729009

EPI_ISL_1729010

EPI_ISL_1729264

EPI_ISL_1729012

EPI_ISL_1729013

EPI_ISL_1729014

EPI_ISL_1729016

EPI_ISL_1729017

EPI_ISL_1729018

EPI_ISL_1729019

EPI_ISL_1729020

EPI_ISL_1729021

EPI_ISL_1729023

EPI_ISL_1729024

EPI_ISL_1729025

EPI_ISL_1729026

EPI_ISL_1729027

EPI_ISL_1729028

EPI_ISL_1729029

EPI_ISL_1729030

EPI_ISL_1729032

EPI_ISL_1729033

EPI_ISL_1729034

EPI_ISL_1729036

EPI_ISL_1729037

EPI_ISL_1729038

EPI_ISL_1729039

EPI_ISL_1729040

EPI_ISL_1729041

EPI_ISL_1729043

EPI_ISL_1729044

EPI_ISL_1729046

EPI_ISL_1729047

EPI_ISL_1729049

EPI_ISL_1729050

EPI_ISL_1729051

EPI_ISL_1729052

EPI_ISL_1729053

EPI_ISL_1729054

EPI_ISL_1729055

EPI_ISL_1729056

EPI_ISL_1729058

EPI_ISL_1729059

EPI_ISL_2549017

EPI_ISL_1729063

EPI_ISL_1729064

EPI_ISL_1729265

EPI_ISL_1729066

EPI_ISL_1729067

EPI_ISL_1729068

EPI_ISL_1729069

EPI_ISL_1729070

EPI_ISL_1729071

EPI_ISL_1729072

EPI_ISL_1729073

EPI_ISL_1729074

EPI_ISL_1729075

EPI_ISL_1729076

EPI_ISL_1729079

EPI_ISL_1729081

EPI_ISL_1729082

EPI_ISL_1729083

EPI_ISL_1729084

EPI_ISL_1729085

EPI_ISL_1729086

EPI_ISL_1729087

EPI_ISL_1729089

EPI_ISL_1729090

EPI_ISL_1729091

EPI_ISL_1729093

EPI_ISL_1729094

EPI_ISL_1729096

EPI_ISL_1729097

EPI_ISL_1729098

EPI_ISL_1729099

EPI_ISL_1729100

EPI_ISL_1729101

EPI_ISL_1729102

EPI_ISL_1729103

EPI_ISL_1729267

EPI_ISL_1729268

EPI_ISL_1729269

EPI_ISL_1729105

EPI_ISL_1729107

EPI_ISL_1729108

EPI_ISL_1729110

EPI_ISL_1729112

EPI_ISL_1729113

EPI_ISL_1729114

EPI_ISL_1729115

EPI_ISL_1729116

EPI_ISL_1729118

EPI_ISL_1729119

EPI_ISL_1729120

EPI_ISL_1729121

EPI_ISL_1729122

EPI_ISL_1729123

EPI_ISL_1729124

EPI_ISL_1729125

EPI_ISL_1729126

EPI_ISL_1729127

EPI_ISL_1729129

EPI_ISL_1729130

EPI_ISL_1729131

EPI_ISL_1729132

EPI_ISL_1729133

EPI_ISL_1729135

EPI_ISL_1729136

EPI_ISL_1729137

EPI_ISL_1729138

EPI_ISL_1729139

EPI_ISL_1729140

EPI_ISL_1729141

EPI_ISL_1729142

EPI_ISL_1729143

EPI_ISL_1729144

EPI_ISL_1729145

EPI_ISL_1729146

EPI_ISL_1729147

EPI_ISL_1729148

EPI_ISL_1729149

EPI_ISL_1729150

EPI_ISL_1729151

EPI_ISL_1729152

EPI_ISL_1729153

EPI_ISL_1729154

EPI_ISL_1729155

EPI_ISL_1729157

EPI_ISL_1729158

EPI_ISL_1729159

EPI_ISL_1729160

EPI_ISL_1729161

EPI_ISL_1729162

EPI_ISL_1729163

EPI_ISL_1729164

EPI_ISL_1729165

EPI_ISL_1729166

EPI_ISL_1729167

EPI_ISL_1729170

EPI_ISL_1729171

EPI_ISL_1729172

EPI_ISL_1729173

EPI_ISL_1729174

EPI_ISL_1729175

EPI_ISL_1729177

EPI_ISL_1729179

EPI_ISL_1729180

EPI_ISL_1729181

EPI_ISL_1729182

EPI_ISL_1729185

EPI_ISL_1729188

EPI_ISL_1729189

EPI_ISL_1729190

EPI_ISL_1729191

EPI_ISL_1729192

EPI_ISL_1729193

EPI_ISL_1729194

EPI_ISL_1729197

EPI_ISL_1729200

EPI_ISL_1729201

EPI_ISL_1729202

EPI_ISL_1729203

EPI_ISL_1729204

EPI_ISL_1729205

EPI_ISL_1729206

EPI_ISL_1729207

EPI_ISL_1729208

EPI_ISL_1729209

EPI_ISL_1729210

EPI_ISL_2549078

EPI_ISL_2549079

EPI_ISL_1729212

EPI_ISL_1729213

EPI_ISL_1729214

EPI_ISL_1729215

EPI_ISL_1729216

EPI_ISL_1729217

EPI_ISL_1729219

EPI_ISL_1729220

EPI_ISL_1729221

EPI_ISL_1729222

EPI_ISL_1729223

EPI_ISL_1729224

EPI_ISL_1729226

EPI_ISL_1729227

EPI_ISL_1729228

EPI_ISL_1729230

EPI_ISL_1729232

EPI_ISL_1729233

EPI_ISL_1729234

EPI_ISL_1729235

EPI_ISL_1729237

EPI_ISL_1729238

EPI_ISL_1729239

EPI_ISL_1729240

EPI_ISL_1729241

EPI_ISL_1729242

EPI_ISL_1729243

EPI_ISL_1729244

EPI_ISL_1729245

EPI_ISL_1729246

EPI_ISL_1729273

EPI_ISL_1729274

EPI_ISL_1729275

EPI_ISL_1729276

EPI_ISL_1729277

EPI_ISL_1729278

EPI_ISL_1729279

EPI_ISL_1729280

EPI_ISL_1729281

EPI_ISL_1729282

EPI_ISL_1729283

EPI_ISL_1729284

EPI_ISL_1729285

EPI_ISL_1729286

EPI_ISL_1729288

EPI_ISL_1729289

EPI_ISL_1729290

EPI_ISL_1729291

EPI_ISL_1729292

EPI_ISL_1729293

EPI_ISL_1729294

EPI_ISL_1729295

EPI_ISL_1729296

EPI_ISL_1729297

EPI_ISL_2549092

EPI_ISL_1729298

EPI_ISL_1729299

EPI_ISL_1729300

EPI_ISL_1729301

EPI_ISL_1729302

EPI_ISL_1729303

EPI_ISL_1729304

EPI_ISL_1729306

EPI_ISL_1729307

EPI_ISL_1729308

EPI_ISL_1729309

EPI_ISL_1729310

EPI_ISL_1729311

EPI_ISL_1729313

EPI_ISL_1729314

EPI_ISL_1729316

EPI_ISL_1729317

EPI_ISL_1729318

EPI_ISL_1729320

EPI_ISL_1729322

EPI_ISL_1729323

EPI_ISL_1729324

EPI_ISL_1729325

EPI_ISL_1729326

EPI_ISL_1729327

EPI_ISL_1729330

EPI_ISL_1729331

EPI_ISL_1729332

EPI_ISL_1729333

EPI_ISL_1729334

EPI_ISL_1729335

EPI_ISL_1729336

EPI_ISL_1729337

EPI_ISL_1729338

EPI_ISL_1729339

EPI_ISL_1729340

EPI_ISL_1729341

EPI_ISL_1729342

EPI_ISL_1729343

EPI_ISL_1729344

EPI_ISL_1729345

EPI_ISL_1729346

EPI_ISL_1729347

EPI_ISL_1729348

EPI_ISL_1729349

EPI_ISL_1729350

EPI_ISL_1729351

EPI_ISL_1729352

EPI_ISL_1729353

EPI_ISL_1729354

EPI_ISL_1729355

EPI_ISL_1729357

EPI_ISL_1729358

EPI_ISL_1729359

EPI_ISL_1729360

EPI_ISL_1729363

EPI_ISL_1729364

EPI_ISL_1729365

EPI_ISL_1729369

EPI_ISL_1729370

EPI_ISL_1729371

EPI_ISL_1729372

EPI_ISL_1729373

EPI_ISL_1729375

EPI_ISL_1729376

EPI_ISL_1729377

EPI_ISL_1729378

EPI_ISL_1729380

EPI_ISL_1729382

EPI_ISL_1729383

EPI_ISL_1729384

EPI_ISL_1729385

EPI_ISL_1729386

EPI_ISL_1729387

EPI_ISL_1729388

EPI_ISL_1729389

EPI_ISL_1729390

EPI_ISL_1729391

EPI_ISL_1729393

EPI_ISL_1729394

EPI_ISL_1729396

EPI_ISL_1729397

EPI_ISL_1729398

EPI_ISL_1729399

EPI_ISL_1729400

EPI_ISL_1729401

EPI_ISL_1729402

EPI_ISL_1729403

EPI_ISL_1729404

EPI_ISL_1729405

EPI_ISL_1729406

EPI_ISL_1729408

EPI_ISL_1587838

EPI_ISL_1729411

EPI_ISL_1729412

EPI_ISL_1729413

EPI_ISL_1729414

EPI_ISL_1729415

EPI_ISL_1729416

EPI_ISL_1729418

EPI_ISL_1729419

EPI_ISL_1729420

EPI_ISL_1729421

EPI_ISL_1729422

EPI_ISL_1729423

EPI_ISL_1729424

EPI_ISL_1729425

EPI_ISL_1729426

EPI_ISL_1729429

EPI_ISL_1729430

EPI_ISL_1729431

EPI_ISL_1729432

EPI_ISL_1729434

EPI_ISL_1729435

EPI_ISL_1729436

EPI_ISL_1729437

EPI_ISL_1729438

EPI_ISL_1729439

EPI_ISL_1729440

EPI_ISL_1729441

EPI_ISL_1729442

EPI_ISL_1729443

EPI_ISL_1729444

EPI_ISL_1729445

EPI_ISL_1729446

EPI_ISL_1729447

EPI_ISL_1729448

EPI_ISL_1729449

EPI_ISL_1729450

EPI_ISL_1729451

EPI_ISL_1729452

EPI_ISL_1729453

EPI_ISL_1729455

EPI_ISL_1729456

EPI_ISL_1729457

EPI_ISL_1729458

EPI_ISL_1729459

EPI_ISL_1729460

EPI_ISL_1729461

EPI_ISL_1729463

EPI_ISL_1729469

EPI_ISL_1729470

EPI_ISL_1729471

EPI_ISL_1729473

EPI_ISL_1729474

EPI_ISL_1729475

EPI_ISL_1729477

EPI_ISL_1729478

EPI_ISL_1729479

EPI_ISL_1729480

EPI_ISL_1729481

EPI_ISL_1729482

EPI_ISL_1729483

EPI_ISL_1729484

EPI_ISL_1729485

EPI_ISL_1729486

EPI_ISL_1729488

EPI_ISL_1729489

EPI_ISL_1729490

EPI_ISL_1729491

EPI_ISL_1729492

EPI_ISL_1729493

EPI_ISL_1729494

EPI_ISL_1729495

EPI_ISL_1729496

EPI_ISL_1729497

EPI_ISL_1729498

EPI_ISL_1729500

EPI_ISL_1729501

EPI_ISL_1729502

EPI_ISL_1729503

EPI_ISL_1729504

EPI_ISL_1729505

EPI_ISL_1729507

EPI_ISL_1729508

EPI_ISL_1729513

EPI_ISL_1729514

EPI_ISL_1729515

EPI_ISL_1729516

EPI_ISL_1729517

EPI_ISL_1729518

EPI_ISL_1729519

EPI_ISL_1729520

EPI_ISL_1729521

EPI_ISL_1729522

EPI_ISL_1729523

EPI_ISL_1729524

EPI_ISL_1729525

EPI_ISL_1729526

EPI_ISL_1729527

EPI_ISL_1729528

EPI_ISL_1729530

EPI_ISL_1729532

EPI_ISL_1729533

EPI_ISL_1729536

EPI_ISL_1729537

EPI_ISL_1729538

EPI_ISL_1729539

EPI_ISL_1729542

EPI_ISL_1729544

EPI_ISL_1729547

EPI_ISL_1729548

EPI_ISL_1729549

EPI_ISL_1729550

EPI_ISL_1729552

EPI_ISL_1729553

EPI_ISL_1729554

EPI_ISL_1729555

EPI_ISL_1729556

EPI_ISL_1729559

EPI_ISL_1729560

EPI_ISL_1729561

EPI_ISL_1729562

EPI_ISL_1729563

EPI_ISL_1729564

EPI_ISL_1729566

EPI_ISL_1729567

EPI_ISL_1729568

EPI_ISL_1729569

EPI_ISL_1729571

EPI_ISL_1729572

EPI_ISL_1729573

EPI_ISL_1729574

EPI_ISL_1729576

EPI_ISL_1729577

EPI_ISL_1729579

EPI_ISL_1729580

EPI_ISL_1729583

EPI_ISL_1729585

EPI_ISL_1729586

EPI_ISL_1729587

EPI_ISL_1729588

EPI_ISL_1729589

EPI_ISL_1729590

EPI_ISL_1729591

EPI_ISL_1729592

EPI_ISL_1729593

EPI_ISL_1729594

EPI_ISL_1729595

EPI_ISL_1729596

EPI_ISL_1729597

EPI_ISL_1729598

EPI_ISL_1729599

EPI_ISL_1729601

EPI_ISL_1729602

EPI_ISL_1729604

EPI_ISL_1729607

EPI_ISL_1729608

EPI_ISL_1729609

EPI_ISL_1729610

EPI_ISL_1729611

EPI_ISL_1729612

EPI_ISL_1729613

EPI_ISL_1729614

EPI_ISL_1729616

EPI_ISL_1729617

EPI_ISL_1729618

EPI_ISL_1729622

EPI_ISL_1729623

EPI_ISL_1729626

EPI_ISL_1729628

EPI_ISL_1729629

EPI_ISL_1729630

EPI_ISL_1729632

EPI_ISL_1729633

EPI_ISL_1729634

EPI_ISL_1729636

EPI_ISL_1729637

EPI_ISL_1729639

EPI_ISL_1729644

EPI_ISL_1729645

EPI_ISL_1729646

EPI_ISL_1729647

EPI_ISL_1729648

EPI_ISL_1729649

EPI_ISL_1729650

EPI_ISL_1729651

EPI_ISL_1729652

EPI_ISL_1729653

EPI_ISL_1729655

EPI_ISL_1729656

EPI_ISL_1729658

EPI_ISL_1729659

EPI_ISL_1729661

EPI_ISL_1729662

EPI_ISL_1729663

EPI_ISL_1729664

EPI_ISL_1729665

EPI_ISL_1729666

EPI_ISL_1729667

EPI_ISL_1729669

EPI_ISL_1729670

EPI_ISL_1729671

EPI_ISL_1729672

EPI_ISL_1729674

EPI_ISL_1729675

EPI_ISL_1729676

EPI_ISL_1729677

EPI_ISL_1729678

EPI_ISL_1729680

EPI_ISL_1729682

EPI_ISL_1729683

EPI_ISL_1729684

EPI_ISL_1729685

EPI_ISL_1729686

EPI_ISL_1729687

EPI_ISL_1729688

EPI_ISL_1729689

EPI_ISL_1729690

EPI_ISL_1729691

EPI_ISL_1729692

EPI_ISL_1729693

EPI_ISL_1729695

EPI_ISL_1729696

EPI_ISL_1729698

EPI_ISL_1729701

EPI_ISL_1729702

EPI_ISL_1729704

EPI_ISL_1729705

EPI_ISL_1729706

EPI_ISL_1729990

EPI_ISL_1729991

EPI_ISL_1729992

EPI_ISL_1729994

EPI_ISL_1729995

EPI_ISL_1729997

EPI_ISL_1730001

EPI_ISL_1730002

EPI_ISL_1730004

EPI_ISL_1730005

EPI_ISL_1730006

EPI_ISL_1730008

EPI_ISL_1730010

EPI_ISL_1730011

EPI_ISL_1730012

EPI_ISL_1730013

EPI_ISL_1730014

EPI_ISL_1730015

EPI_ISL_1730016

EPI_ISL_1730043

EPI_ISL_1730047

EPI_ISL_1730049

EPI_ISL_1730051

EPI_ISL_1730052

EPI_ISL_1730053

EPI_ISL_1730056

EPI_ISL_1730057

EPI_ISL_1730058

EPI_ISL_1730059

EPI_ISL_1730061

EPI_ISL_1730062

EPI_ISL_1730065

EPI_ISL_1730067

EPI_ISL_1730068

EPI_ISL_1730070

EPI_ISL_1730106

EPI_ISL_1730108

EPI_ISL_1730109

EPI_ISL_1730110

EPI_ISL_1730112

EPI_ISL_1730113

EPI_ISL_1730114

EPI_ISL_1730118

EPI_ISL_1730126

EPI_ISL_1730129

EPI_ISL_1730130

EPI_ISL_1730131

EPI_ISL_1730132

EPI_ISL_1736804

EPI_ISL_1730273

EPI_ISL_1730275

EPI_ISL_1730277

EPI_ISL_1730278

EPI_ISL_1730280

EPI_ISL_1730282

EPI_ISL_1730283

EPI_ISL_1730292

EPI_ISL_1730293

EPI_ISL_1730294

EPI_ISL_1730304

EPI_ISL_1730317

EPI_ISL_1730319

EPI_ISL_1730320

EPI_ISL_1730322

EPI_ISL_1730323

EPI_ISL_1730347

EPI_ISL_1730369

EPI_ISL_1730370

EPI_ISL_1730372

EPI_ISL_1730374

EPI_ISL_1730382

EPI_ISL_1730384

EPI_ISL_1730385

EPI_ISL_1730387

EPI_ISL_1730399

EPI_ISL_1730402

EPI_ISL_1730404

EPI_ISL_1730406

EPI_ISL_1730408

EPI_ISL_1730411

EPI_ISL_1730413

EPI_ISL_1730414

EPI_ISL_1730415

EPI_ISL_1730416

EPI_ISL_1730418

EPI_ISL_1730424

EPI_ISL_1730425

EPI_ISL_1730535

EPI_ISL_1730536

EPI_ISL_1730545

EPI_ISL_1730546

EPI_ISL_1730547

EPI_ISL_1730558

EPI_ISL_1730561

EPI_ISL_1730562

EPI_ISL_1730567

EPI_ISL_1730568

EPI_ISL_1730569

EPI_ISL_1730573

EPI_ISL_1730575

EPI_ISL_1730580

EPI_ISL_1730581

EPI_ISL_1730582

EPI_ISL_1730583

EPI_ISL_1730584

EPI_ISL_1730588

EPI_ISL_1730589

EPI_ISL_1730591

EPI_ISL_1730592

EPI_ISL_1730593

EPI_ISL_1730595

EPI_ISL_1730596

EPI_ISL_1730597

EPI_ISL_1730598

EPI_ISL_1730599

EPI_ISL_1730600

EPI_ISL_1730601

EPI_ISL_1730602

EPI_ISL_1730603

EPI_ISL_1730604

EPI_ISL_1730605

EPI_ISL_1730606

EPI_ISL_1730607

EPI_ISL_1730608

EPI_ISL_1730610

EPI_ISL_1730611

EPI_ISL_1730612

EPI_ISL_1730613

EPI_ISL_1730616

EPI_ISL_1730617

EPI_ISL_1730618

EPI_ISL_1730619

EPI_ISL_1730620

EPI_ISL_1730623

EPI_ISL_1730624

EPI_ISL_1730626

EPI_ISL_1730627

EPI_ISL_1730628

EPI_ISL_1730630

EPI_ISL_1730631

EPI_ISL_1730632

EPI_ISL_1730636

EPI_ISL_1730639

EPI_ISL_1730641

EPI_ISL_1730642

EPI_ISL_1730643

EPI_ISL_1730644

EPI_ISL_1730645

EPI_ISL_1730646

EPI_ISL_1730647

EPI_ISL_1730649

EPI_ISL_1730651

EPI_ISL_1730656

EPI_ISL_1730657

EPI_ISL_1730658

EPI_ISL_1730659

EPI_ISL_1730660

EPI_ISL_1730663

EPI_ISL_1730664

EPI_ISL_1730666

EPI_ISL_1730667

EPI_ISL_1730668

EPI_ISL_1730669

EPI_ISL_1730670

EPI_ISL_1730671

EPI_ISL_1730672

EPI_ISL_1730673

EPI_ISL_1730674

EPI_ISL_1730675

EPI_ISL_1730678

EPI_ISL_1730679

EPI_ISL_1730680

EPI_ISL_1730681

EPI_ISL_1730682

EPI_ISL_1730683

EPI_ISL_1730684

EPI_ISL_1730685

EPI_ISL_1730686

EPI_ISL_1730687

EPI_ISL_1730688

EPI_ISL_1730689

EPI_ISL_1730690

EPI_ISL_1730692

EPI_ISL_1730693

EPI_ISL_1730694

EPI_ISL_1730696

EPI_ISL_1730697

EPI_ISL_1730699

EPI_ISL_1730700

EPI_ISL_1730702

EPI_ISL_1730705

EPI_ISL_1730707

EPI_ISL_1730708

EPI_ISL_1730709

EPI_ISL_1730710

EPI_ISL_1730712

EPI_ISL_1730713

EPI_ISL_1730715

EPI_ISL_1730718

EPI_ISL_1730719

EPI_ISL_1730720

EPI_ISL_1730723

EPI_ISL_1730727

EPI_ISL_1730730

EPI_ISL_1730732

EPI_ISL_1730734

EPI_ISL_1730737

EPI_ISL_1730738

EPI_ISL_1730740

EPI_ISL_1730741

EPI_ISL_1730742

EPI_ISL_1730744

EPI_ISL_1730745

EPI_ISL_1730746

EPI_ISL_1730747

EPI_ISL_1730748

EPI_ISL_1730749

EPI_ISL_1730750

EPI_ISL_1730751

EPI_ISL_1730752

EPI_ISL_1730753

EPI_ISL_1730754

EPI_ISL_1730755

EPI_ISL_1730757

EPI_ISL_1730760

EPI_ISL_1730761

EPI_ISL_1730762

EPI_ISL_1730763

EPI_ISL_1730764

EPI_ISL_1730765

EPI_ISL_1730768

EPI_ISL_1730769

EPI_ISL_1730771

EPI_ISL_1730772

EPI_ISL_1730773

EPI_ISL_1730774

EPI_ISL_1730775

EPI_ISL_1730776

EPI_ISL_1730778

EPI_ISL_1730780

EPI_ISL_1730781

EPI_ISL_1730782

EPI_ISL_1730784

EPI_ISL_1730786

EPI_ISL_1730788

EPI_ISL_1730793

EPI_ISL_1730796

EPI_ISL_1730797

EPI_ISL_1730798

EPI_ISL_1730800

EPI_ISL_1730805

EPI_ISL_1730807

EPI_ISL_1730808

EPI_ISL_1730809

EPI_ISL_1730810

EPI_ISL_1730811

EPI_ISL_1730813

EPI_ISL_1730814

EPI_ISL_1730815

EPI_ISL_1730816

EPI_ISL_1730817

EPI_ISL_1730818

EPI_ISL_1730819

EPI_ISL_1730889

EPI_ISL_1730890

EPI_ISL_1730891

EPI_ISL_1730892

EPI_ISL_1730894

EPI_ISL_1730900

EPI_ISL_1730901

EPI_ISL_1730902

EPI_ISL_1730903

EPI_ISL_1730905

EPI_ISL_1730907

EPI_ISL_1730908

EPI_ISL_1730909

EPI_ISL_1730912

EPI_ISL_1730914

EPI_ISL_1730917

EPI_ISL_1730920

EPI_ISL_1730924

EPI_ISL_1730925

EPI_ISL_1730932

EPI_ISL_1730933

EPI_ISL_1730934

EPI_ISL_1730938

EPI_ISL_1730939

EPI_ISL_1730940

EPI_ISL_1730941

EPI_ISL_1730942

EPI_ISL_1730943

EPI_ISL_1730946

EPI_ISL_1730947

EPI_ISL_1730949

EPI_ISL_1730950

EPI_ISL_1730952

EPI_ISL_1730953

EPI_ISL_1730959

EPI_ISL_1730961

EPI_ISL_1730962

EPI_ISL_1730963

EPI_ISL_1730966

EPI_ISL_1730967

EPI_ISL_1730968

EPI_ISL_1730970

EPI_ISL_1730971

EPI_ISL_1730974

EPI_ISL_1730975

EPI_ISL_1730977

EPI_ISL_1730980

EPI_ISL_1730983

EPI_ISL_1730984

EPI_ISL_1730988

EPI_ISL_1730989

EPI_ISL_1730991

EPI_ISL_1730992

EPI_ISL_1730993

EPI_ISL_1730998

EPI_ISL_1731000

EPI_ISL_1731001

EPI_ISL_1731003

EPI_ISL_1731004

EPI_ISL_1731005

EPI_ISL_1731006

EPI_ISL_1731007

EPI_ISL_1731008

EPI_ISL_1731009

EPI_ISL_1731010

EPI_ISL_1731011

EPI_ISL_1731013

EPI_ISL_1731014

EPI_ISL_1731021

EPI_ISL_1731022

EPI_ISL_1731023

EPI_ISL_1731025

EPI_ISL_1731028

EPI_ISL_1731030

EPI_ISL_1731031

EPI_ISL_1731032

EPI_ISL_1731033

EPI_ISL_1731035

EPI_ISL_1731037

EPI_ISL_1731038

EPI_ISL_1731039

EPI_ISL_1731040

EPI_ISL_1731041

EPI_ISL_1731159

EPI_ISL_1731162

EPI_ISL_1731163

EPI_ISL_1731164

EPI_ISL_1731166

EPI_ISL_1731168

EPI_ISL_1731169

EPI_ISL_1731170

EPI_ISL_1731172

EPI_ISL_1731176

EPI_ISL_1731178

EPI_ISL_1731179

EPI_ISL_1731180

EPI_ISL_1731181

EPI_ISL_1731182

EPI_ISL_1731183

EPI_ISL_1731184

EPI_ISL_1731185

EPI_ISL_1731186

EPI_ISL_1731187

EPI_ISL_1731189

EPI_ISL_1731191

EPI_ISL_1731193

EPI_ISL_1731194

EPI_ISL_1731195

EPI_ISL_1731197

EPI_ISL_1731199

EPI_ISL_1731200

EPI_ISL_1731203

EPI_ISL_1731205

EPI_ISL_1731206

EPI_ISL_1731207

EPI_ISL_1731208

EPI_ISL_1731209

EPI_ISL_1731210

EPI_ISL_1731211

EPI_ISL_1731212

EPI_ISL_1731213

EPI_ISL_1731214

EPI_ISL_1731216

EPI_ISL_1731217

EPI_ISL_1731218

EPI_ISL_1731220

EPI_ISL_1731224

EPI_ISL_1731225

EPI_ISL_1731227

EPI_ISL_1731229

EPI_ISL_1731230

EPI_ISL_1731231

EPI_ISL_1731236

EPI_ISL_1731237

EPI_ISL_1731238

EPI_ISL_1731239

EPI_ISL_1731240

EPI_ISL_1731246

EPI_ISL_1731247

EPI_ISL_1731248

EPI_ISL_1731249

EPI_ISL_1731250

EPI_ISL_1731251

EPI_ISL_1731253

EPI_ISL_1731254

EPI_ISL_1731257

EPI_ISL_1731258

EPI_ISL_1731259

EPI_ISL_1731261

EPI_ISL_1731262

EPI_ISL_1731264

EPI_ISL_1731265

EPI_ISL_1731267

EPI_ISL_1731268

EPI_ISL_1731269

EPI_ISL_1731270

EPI_ISL_1731271

EPI_ISL_1731273

EPI_ISL_1731274

EPI_ISL_1731275

EPI_ISL_1731276

EPI_ISL_1731277

EPI_ISL_1731278

EPI_ISL_1731279

EPI_ISL_1731281

EPI_ISL_1731282

EPI_ISL_1731283

EPI_ISL_1731284

EPI_ISL_1731285

EPI_ISL_1731286

EPI_ISL_1731287

EPI_ISL_1731288

EPI_ISL_1731290

EPI_ISL_1731291

EPI_ISL_1731292

EPI_ISL_1731294

EPI_ISL_1731295

EPI_ISL_1731296

EPI_ISL_1731297

EPI_ISL_1731300

EPI_ISL_1731301

EPI_ISL_1731302

EPI_ISL_1731303

EPI_ISL_1731304

EPI_ISL_1731305

EPI_ISL_1731306

EPI_ISL_1731308

EPI_ISL_1731309

EPI_ISL_1731310

EPI_ISL_1731312

EPI_ISL_1731313

EPI_ISL_1731314

EPI_ISL_1731315

EPI_ISL_1731316

EPI_ISL_1731318

EPI_ISL_1731320

EPI_ISL_1731321

EPI_ISL_1731322

EPI_ISL_1731323

EPI_ISL_1731324

EPI_ISL_1731325

EPI_ISL_1731327

EPI_ISL_1731328

EPI_ISL_1731329

EPI_ISL_1731331

EPI_ISL_1731332

EPI_ISL_1731333

EPI_ISL_1731334

EPI_ISL_1731335

EPI_ISL_1731336

EPI_ISL_1731337

EPI_ISL_1731338

EPI_ISL_1731339

EPI_ISL_1731340

EPI_ISL_1731342

EPI_ISL_1731343

EPI_ISL_1731344

EPI_ISL_1731345

EPI_ISL_1731346

EPI_ISL_1731347

EPI_ISL_1731349

EPI_ISL_1731350

EPI_ISL_1731351

EPI_ISL_1731353

EPI_ISL_1731354

EPI_ISL_1731355

EPI_ISL_1731356

EPI_ISL_1731357

EPI_ISL_1731358

EPI_ISL_1731359

EPI_ISL_1731360

EPI_ISL_1731361

EPI_ISL_1731362

EPI_ISL_1731364

EPI_ISL_1731365

EPI_ISL_1731366

EPI_ISL_1731368

EPI_ISL_1731369

EPI_ISL_1731371

EPI_ISL_1731372

EPI_ISL_1731373

EPI_ISL_1731374

EPI_ISL_1731375

EPI_ISL_1731376

EPI_ISL_1731377

EPI_ISL_1731378

EPI_ISL_1731379

EPI_ISL_1731380

EPI_ISL_1731382

EPI_ISL_1731383

EPI_ISL_1731384

EPI_ISL_1731385

EPI_ISL_1731386

EPI_ISL_1731387

EPI_ISL_1731388

EPI_ISL_1731389

EPI_ISL_1731390

EPI_ISL_1731391

EPI_ISL_1731392

EPI_ISL_1731394

EPI_ISL_1731395

EPI_ISL_1731396

EPI_ISL_1731397

EPI_ISL_1731398

EPI_ISL_1731399

EPI_ISL_1731400

EPI_ISL_1731404

EPI_ISL_1731405

EPI_ISL_1731406

EPI_ISL_1731407

EPI_ISL_1731408

EPI_ISL_1731409

EPI_ISL_1731411

EPI_ISL_1731413

EPI_ISL_1731414

EPI_ISL_1731415

EPI_ISL_1731416

EPI_ISL_1731418

EPI_ISL_1731421

EPI_ISL_1731423

EPI_ISL_1731424

EPI_ISL_1731425

EPI_ISL_1731426

EPI_ISL_1731427

EPI_ISL_1731429

EPI_ISL_1731430

EPI_ISL_1731433

EPI_ISL_1731434

EPI_ISL_1731435

EPI_ISL_1731437

EPI_ISL_1731439

EPI_ISL_1731440

EPI_ISL_1731441

EPI_ISL_1731442

EPI_ISL_1731443

EPI_ISL_1731451

EPI_ISL_1731453

EPI_ISL_1731454

EPI_ISL_1731457

EPI_ISL_1731458

EPI_ISL_1731459

EPI_ISL_1731460

EPI_ISL_1731464

EPI_ISL_1731465

EPI_ISL_1731466

EPI_ISL_1731467

EPI_ISL_1731468

EPI_ISL_1731469

EPI_ISL_1731471

EPI_ISL_1731472

EPI_ISL_1731473

EPI_ISL_1731474

EPI_ISL_1731475

EPI_ISL_1731476

EPI_ISL_1731478

EPI_ISL_1731479

EPI_ISL_1731481

EPI_ISL_1731482

EPI_ISL_1731483

EPI_ISL_1731486

EPI_ISL_1731487

EPI_ISL_1731488

EPI_ISL_1731489

EPI_ISL_1731490

EPI_ISL_1731491

EPI_ISL_1731492

EPI_ISL_1731493

EPI_ISL_1731494

EPI_ISL_1731495

EPI_ISL_1731496

EPI_ISL_1731497

EPI_ISL_1731499

EPI_ISL_1731500

EPI_ISL_1731501

EPI_ISL_1731502

EPI_ISL_1731503

EPI_ISL_1731507

EPI_ISL_1731508

EPI_ISL_1731509

EPI_ISL_1731510

EPI_ISL_1731511

EPI_ISL_1731512

EPI_ISL_1731514

EPI_ISL_1731515

EPI_ISL_1731516

EPI_ISL_1731517

EPI_ISL_1731518

EPI_ISL_1731519

EPI_ISL_1731520

EPI_ISL_1731521

EPI_ISL_1731522

EPI_ISL_1731523

EPI_ISL_1731529

EPI_ISL_1731530

EPI_ISL_1731531

EPI_ISL_1731532

EPI_ISL_1731533

EPI_ISL_1731534

EPI_ISL_1731535

EPI_ISL_1731537

EPI_ISL_1731538

EPI_ISL_1731660

EPI_ISL_1731661

EPI_ISL_1731662

EPI_ISL_1731663

EPI_ISL_1731664

EPI_ISL_1731666

EPI_ISL_1731669

EPI_ISL_1731670

EPI_ISL_1731671

EPI_ISL_1731673

EPI_ISL_1731681

EPI_ISL_1731685

EPI_ISL_1731688

EPI_ISL_1731689

EPI_ISL_1731700

EPI_ISL_1731702

EPI_ISL_1731704

EPI_ISL_1731705

EPI_ISL_1731707

EPI_ISL_1731708

EPI_ISL_1731709

EPI_ISL_1731711

EPI_ISL_1731713

EPI_ISL_1731714

EPI_ISL_1731715

EPI_ISL_1731716

EPI_ISL_1731717

EPI_ISL_1731718

EPI_ISL_1731719

EPI_ISL_1731720

EPI_ISL_1731721

EPI_ISL_1731722

EPI_ISL_1731723

EPI_ISL_1731724

EPI_ISL_1731726

EPI_ISL_1731728

EPI_ISL_1731729

EPI_ISL_1731730

EPI_ISL_1731732

EPI_ISL_1731733

EPI_ISL_1731734

EPI_ISL_1731735

EPI_ISL_1731736

EPI_ISL_1731737

EPI_ISL_1731738

EPI_ISL_1731739

EPI_ISL_1731740

EPI_ISL_1731741

EPI_ISL_1731742

EPI_ISL_1731743

EPI_ISL_1731744

EPI_ISL_1731745

EPI_ISL_1731746

EPI_ISL_1731747

EPI_ISL_1731748

EPI_ISL_1731749

EPI_ISL_1731750

EPI_ISL_1731751

EPI_ISL_1731765

EPI_ISL_1731816

EPI_ISL_1731817

EPI_ISL_1732002

EPI_ISL_1732003

EPI_ISL_1732004

EPI_ISL_1732005

EPI_ISL_1732070

EPI_ISL_1732075

EPI_ISL_1732076

EPI_ISL_1732077

EPI_ISL_1732078

EPI_ISL_1732107

EPI_ISL_1732159

EPI_ISL_1732160

EPI_ISL_1732161

EPI_ISL_1732162

EPI_ISL_1732163

EPI_ISL_1732165

EPI_ISL_1732166

EPI_ISL_1732168

EPI_ISL_1732171

EPI_ISL_1732178

EPI_ISL_1732179

EPI_ISL_1732181

EPI_ISL_1732182

EPI_ISL_1732183

EPI_ISL_1732184

EPI_ISL_1732185

EPI_ISL_1732186

EPI_ISL_1732187

EPI_ISL_1732188

EPI_ISL_1732189

EPI_ISL_1732190

EPI_ISL_1732191

EPI_ISL_1732192

EPI_ISL_1732193

EPI_ISL_1732194

EPI_ISL_1732195

EPI_ISL_1732196

EPI_ISL_1732197

EPI_ISL_1732198

EPI_ISL_1732199

EPI_ISL_1732201

EPI_ISL_1732202

EPI_ISL_1732204

EPI_ISL_1732205

EPI_ISL_1732206

EPI_ISL_1732207

EPI_ISL_1732208

EPI_ISL_1732209

EPI_ISL_1732210

EPI_ISL_1732214

EPI_ISL_1732215

EPI_ISL_1732216

EPI_ISL_1732217

EPI_ISL_1732218

EPI_ISL_1732219

EPI_ISL_1732221

EPI_ISL_1732222

EPI_ISL_1732223

EPI_ISL_1732224

EPI_ISL_1732225

EPI_ISL_1732226

EPI_ISL_1732231

EPI_ISL_1732278

EPI_ISL_1732279

EPI_ISL_1732285

EPI_ISL_1732286

EPI_ISL_1732287

EPI_ISL_1732289

EPI_ISL_1732293

EPI_ISL_1732423

EPI_ISL_1732427

EPI_ISL_1732428

EPI_ISL_1732430

EPI_ISL_1732431

EPI_ISL_1732432

EPI_ISL_1732497

EPI_ISL_1732499

EPI_ISL_1732504

EPI_ISL_1732542

EPI_ISL_1732543

EPI_ISL_1732545

EPI_ISL_1732547

EPI_ISL_1732548

EPI_ISL_1732549

EPI_ISL_1732550

EPI_ISL_1732557

EPI_ISL_1732560

EPI_ISL_1732561

EPI_ISL_1732564

EPI_ISL_1732565

EPI_ISL_1732572

EPI_ISL_1732576

EPI_ISL_1732584

EPI_ISL_1732592

EPI_ISL_1732595

EPI_ISL_1732596

EPI_ISL_1732598

EPI_ISL_1732599

EPI_ISL_1732602

EPI_ISL_1732604

EPI_ISL_1732605

EPI_ISL_1732607

EPI_ISL_1732611

EPI_ISL_1732613

EPI_ISL_1732614

EPI_ISL_1732620

EPI_ISL_1732621

EPI_ISL_1732622

EPI_ISL_1732630

EPI_ISL_1732633

EPI_ISL_1732634

EPI_ISL_1732635

EPI_ISL_1732636

EPI_ISL_1732639

EPI_ISL_1732640

EPI_ISL_1732644

EPI_ISL_1732646

EPI_ISL_1732656

EPI_ISL_1732657

EPI_ISL_1732659

EPI_ISL_1732663

EPI_ISL_1732666

EPI_ISL_1732669

EPI_ISL_1732670

EPI_ISL_1732671

EPI_ISL_1732677

EPI_ISL_1732678

EPI_ISL_1732687

EPI_ISL_1732688

EPI_ISL_1732692

EPI_ISL_1732693

EPI_ISL_1732694

EPI_ISL_1732695

EPI_ISL_1732696

EPI_ISL_1732697

EPI_ISL_1732698

EPI_ISL_1732699

EPI_ISL_1732701

EPI_ISL_1732703

EPI_ISL_1732704

EPI_ISL_1732705

EPI_ISL_1732708

EPI_ISL_1732712

EPI_ISL_1732713

EPI_ISL_1732717

EPI_ISL_1732718

EPI_ISL_1732719

EPI_ISL_1732720

EPI_ISL_1732721

EPI_ISL_1732723

EPI_ISL_1732724

EPI_ISL_1732726

EPI_ISL_1732727

EPI_ISL_1732728

EPI_ISL_1732729

EPI_ISL_1732732

EPI_ISL_1732733

EPI_ISL_1732735

EPI_ISL_1732736

EPI_ISL_1732737

EPI_ISL_1732739

EPI_ISL_1732740

EPI_ISL_1732743

EPI_ISL_1732744

EPI_ISL_1732747

EPI_ISL_1732748

EPI_ISL_1732752

EPI_ISL_1732755

EPI_ISL_1732757

EPI_ISL_1732759

EPI_ISL_1732760

EPI_ISL_1732762

EPI_ISL_1732763

EPI_ISL_1732764

EPI_ISL_1732765

EPI_ISL_1732769

EPI_ISL_1732777

EPI_ISL_1732790

EPI_ISL_1732803

EPI_ISL_1732804

EPI_ISL_1732805

EPI_ISL_1732808

EPI_ISL_1732809

EPI_ISL_1732810

EPI_ISL_1732812

EPI_ISL_1732813

EPI_ISL_1732814

EPI_ISL_1732815

EPI_ISL_1732816

EPI_ISL_1732817

EPI_ISL_1732819

EPI_ISL_1732824

EPI_ISL_1732826

EPI_ISL_1732828

EPI_ISL_1732831

EPI_ISL_1732832

EPI_ISL_1732833

EPI_ISL_1732834

EPI_ISL_1732837

EPI_ISL_1732838

EPI_ISL_1732839

EPI_ISL_1732841

EPI_ISL_1732842

EPI_ISL_1732843

EPI_ISL_1732845

EPI_ISL_1732846

EPI_ISL_1732848

EPI_ISL_1732849

EPI_ISL_1732852

EPI_ISL_1732854

EPI_ISL_1732855

EPI_ISL_1732857

EPI_ISL_1732858

EPI_ISL_1732859

EPI_ISL_1732860

EPI_ISL_1732866

EPI_ISL_1732868

EPI_ISL_1732869

EPI_ISL_1732878

EPI_ISL_1732884

EPI_ISL_1732889

EPI_ISL_1732893

EPI_ISL_1732899

EPI_ISL_1732901

EPI_ISL_1732903

EPI_ISL_1732907

EPI_ISL_1732908

EPI_ISL_1732909

EPI_ISL_1732914

EPI_ISL_1732925

EPI_ISL_1732926

EPI_ISL_1732927

EPI_ISL_1732929

EPI_ISL_1732930

EPI_ISL_1732933

EPI_ISL_1732934

EPI_ISL_1732935

EPI_ISL_1732936

EPI_ISL_1732937

EPI_ISL_1732941

EPI_ISL_1732943

EPI_ISL_1732944

EPI_ISL_1732950

EPI_ISL_1732951

EPI_ISL_1732952

EPI_ISL_1732953

EPI_ISL_1732956

EPI_ISL_1732958

EPI_ISL_1732960

EPI_ISL_1732962

EPI_ISL_1732965

EPI_ISL_1732969

EPI_ISL_1732971

EPI_ISL_1732974

EPI_ISL_1732975

EPI_ISL_1732976

EPI_ISL_1732978

EPI_ISL_1732979

EPI_ISL_1732980

EPI_ISL_1732981

EPI_ISL_1732984

EPI_ISL_1732985

EPI_ISL_1732986

EPI_ISL_1732990

EPI_ISL_1732991

EPI_ISL_1732992

EPI_ISL_1732994

EPI_ISL_1732995

EPI_ISL_1732997

EPI_ISL_1732999

EPI_ISL_1733002

EPI_ISL_1733004

EPI_ISL_1733005

EPI_ISL_1733007

EPI_ISL_1733008

EPI_ISL_1733009

EPI_ISL_1733011

EPI_ISL_1733012

EPI_ISL_1733015

EPI_ISL_1733017

EPI_ISL_1733018

EPI_ISL_1733019

EPI_ISL_1733020

EPI_ISL_1733021

EPI_ISL_1733022

EPI_ISL_1733023

EPI_ISL_1733026

EPI_ISL_1733029

EPI_ISL_1733030

EPI_ISL_1733031

EPI_ISL_1733032

EPI_ISL_1733035

EPI_ISL_1733037

EPI_ISL_1733038

EPI_ISL_1733039

EPI_ISL_1733040

EPI_ISL_1733043

EPI_ISL_1733045

EPI_ISL_1733046

EPI_ISL_1733047

EPI_ISL_1733051

EPI_ISL_1733054

EPI_ISL_1733055

EPI_ISL_1733056

EPI_ISL_1733057

EPI_ISL_1733058

EPI_ISL_1733059

EPI_ISL_1733061

EPI_ISL_1733062

EPI_ISL_1733066

EPI_ISL_1733069

EPI_ISL_1733071

EPI_ISL_1733075

EPI_ISL_1733076

EPI_ISL_1733077

EPI_ISL_1733078

EPI_ISL_1733079

EPI_ISL_1733083

EPI_ISL_1733084

EPI_ISL_1733085

EPI_ISL_1733086

EPI_ISL_1733087

EPI_ISL_1733088

EPI_ISL_1733092

EPI_ISL_1733093

EPI_ISL_1733097

EPI_ISL_1733098

EPI_ISL_1733100

EPI_ISL_1733101

EPI_ISL_1733103

EPI_ISL_1733104

EPI_ISL_1733105

EPI_ISL_1733107

EPI_ISL_1733108

EPI_ISL_1733110

EPI_ISL_1733112

EPI_ISL_1733117

EPI_ISL_1733118

EPI_ISL_1733120

EPI_ISL_1733121

EPI_ISL_1733123

EPI_ISL_1733124

EPI_ISL_1733126

EPI_ISL_1733127

EPI_ISL_1733131

EPI_ISL_1733134

EPI_ISL_1733138

EPI_ISL_1733139

EPI_ISL_1733141

EPI_ISL_1733142

EPI_ISL_1733143

EPI_ISL_1733144

EPI_ISL_1733147

EPI_ISL_1733148

EPI_ISL_1733149

EPI_ISL_1733150

EPI_ISL_1733151

EPI_ISL_1733154

EPI_ISL_1733156

EPI_ISL_1733158

EPI_ISL_1733159

EPI_ISL_1733160

EPI_ISL_1733161

EPI_ISL_1733162

EPI_ISL_1733163

EPI_ISL_1733164

EPI_ISL_1733167

EPI_ISL_1733170

EPI_ISL_1733171

EPI_ISL_1733173

EPI_ISL_1733174

EPI_ISL_1733176

EPI_ISL_1733177

EPI_ISL_1733178

EPI_ISL_1733180

EPI_ISL_1733181

EPI_ISL_1733183

EPI_ISL_1733185

EPI_ISL_1733186

EPI_ISL_1733188

EPI_ISL_1733192

EPI_ISL_1733193

EPI_ISL_1733195

EPI_ISL_1733196

EPI_ISL_1733197

EPI_ISL_1733198

EPI_ISL_1733200

EPI_ISL_1733201

EPI_ISL_1733202

EPI_ISL_1733203

EPI_ISL_1733204

EPI_ISL_1733206

EPI_ISL_1733207

EPI_ISL_1733208

EPI_ISL_1733209

EPI_ISL_1733210

EPI_ISL_1733211

EPI_ISL_1733215

EPI_ISL_1733216

EPI_ISL_1733217

EPI_ISL_1733219

EPI_ISL_1733221

EPI_ISL_1733222

EPI_ISL_1733223

EPI_ISL_1733225

EPI_ISL_1733228

EPI_ISL_1733229

EPI_ISL_1733230

EPI_ISL_1733233

EPI_ISL_1733234

EPI_ISL_1733235

EPI_ISL_1733236

EPI_ISL_1733237

EPI_ISL_1733244

EPI_ISL_1733245

EPI_ISL_1733246

EPI_ISL_1733247

EPI_ISL_1733249

EPI_ISL_1733250

EPI_ISL_1733252

EPI_ISL_1733255

EPI_ISL_1733257

EPI_ISL_1733258

EPI_ISL_1733259

EPI_ISL_1733260

EPI_ISL_1733262

EPI_ISL_1733264

EPI_ISL_1733265

EPI_ISL_1733266

EPI_ISL_1733269

EPI_ISL_1733273

EPI_ISL_1733274

EPI_ISL_1733275

EPI_ISL_1733276

EPI_ISL_1733278

EPI_ISL_1733280

EPI_ISL_1733281

EPI_ISL_1733282

EPI_ISL_1733285

EPI_ISL_1733286

EPI_ISL_1733289

EPI_ISL_1733291

EPI_ISL_1733296

EPI_ISL_1733297

EPI_ISL_1733298

EPI_ISL_1733301

EPI_ISL_1733305

EPI_ISL_1733310

EPI_ISL_1733312

EPI_ISL_1733313

EPI_ISL_1733314

EPI_ISL_1733315

EPI_ISL_1733317

EPI_ISL_1733318

EPI_ISL_1733320

EPI_ISL_1733322

EPI_ISL_1733323

EPI_ISL_1733325

EPI_ISL_1733327

EPI_ISL_1733328

EPI_ISL_1733329

EPI_ISL_1733330

EPI_ISL_1733331

EPI_ISL_1733332

EPI_ISL_1733333

EPI_ISL_1733338

EPI_ISL_1733339

EPI_ISL_1733341

EPI_ISL_1733342

EPI_ISL_1733346

EPI_ISL_1733348

EPI_ISL_1733349

EPI_ISL_1733350

EPI_ISL_1733352

EPI_ISL_1733355

EPI_ISL_1733356

EPI_ISL_1733357

EPI_ISL_1733359

EPI_ISL_1733360

EPI_ISL_1733361

EPI_ISL_1733362

EPI_ISL_1733364

EPI_ISL_1733365

EPI_ISL_1733366

EPI_ISL_1733367

EPI_ISL_1733368

EPI_ISL_1733369

EPI_ISL_1733370

EPI_ISL_1733371

EPI_ISL_1733374

EPI_ISL_1733375

EPI_ISL_1733377

EPI_ISL_1733378

EPI_ISL_1733381

EPI_ISL_1733385

EPI_ISL_1733386

EPI_ISL_1733387

EPI_ISL_1733388

EPI_ISL_1733389

EPI_ISL_1733391

EPI_ISL_1733392

EPI_ISL_1733397

EPI_ISL_1733399

EPI_ISL_1733400

EPI_ISL_1733401

EPI_ISL_1733404

EPI_ISL_1733405

EPI_ISL_1733406

EPI_ISL_1733408

EPI_ISL_1733409

EPI_ISL_1733412

EPI_ISL_1733414

EPI_ISL_1733415

EPI_ISL_1733416

EPI_ISL_1733417

EPI_ISL_1733418

EPI_ISL_1733421

EPI_ISL_1733424

EPI_ISL_1733429

EPI_ISL_1733430

EPI_ISL_1733436

EPI_ISL_1733440

EPI_ISL_1733441

EPI_ISL_1733442

EPI_ISL_1733454

EPI_ISL_1733458

EPI_ISL_1733461

EPI_ISL_1733462

EPI_ISL_1733463

EPI_ISL_1733473

EPI_ISL_1733487

EPI_ISL_1733488

EPI_ISL_1733490

EPI_ISL_1733491

EPI_ISL_1733495

EPI_ISL_1733498

EPI_ISL_1733500

EPI_ISL_1733502

EPI_ISL_1733503

EPI_ISL_1733504

EPI_ISL_1733506

EPI_ISL_1733508

EPI_ISL_1733509

EPI_ISL_1733511

EPI_ISL_1733512

EPI_ISL_1733519

EPI_ISL_1733520

EPI_ISL_1733521

EPI_ISL_1733522

EPI_ISL_1733523

EPI_ISL_1733525

EPI_ISL_1733526

EPI_ISL_1733527

EPI_ISL_1733528

EPI_ISL_1733530

EPI_ISL_1733532

EPI_ISL_1733535

EPI_ISL_1733536

EPI_ISL_1733537

EPI_ISL_1733538

EPI_ISL_1733539

EPI_ISL_1733541

EPI_ISL_1733543

EPI_ISL_1733546

EPI_ISL_1733547

EPI_ISL_1733549

EPI_ISL_1733550

EPI_ISL_1733552

EPI_ISL_1733553

EPI_ISL_1733554

EPI_ISL_1733555

EPI_ISL_1733557

EPI_ISL_1733558

EPI_ISL_1733560

EPI_ISL_1733562

EPI_ISL_1733563

EPI_ISL_1733564

EPI_ISL_1733565

EPI_ISL_1733566

EPI_ISL_1733574

EPI_ISL_1733576

EPI_ISL_1733580

EPI_ISL_1733583

EPI_ISL_1733584

EPI_ISL_1733585

EPI_ISL_1733586

EPI_ISL_1733587

EPI_ISL_1733590

EPI_ISL_1733591

EPI_ISL_1733592

EPI_ISL_1733593

EPI_ISL_1733594

EPI_ISL_1733595

EPI_ISL_1733597

EPI_ISL_1733599

EPI_ISL_1733602

EPI_ISL_1733604

EPI_ISL_1733606

EPI_ISL_1733609

EPI_ISL_1733610

EPI_ISL_1733612

EPI_ISL_1733619

EPI_ISL_1733622

EPI_ISL_1733623

EPI_ISL_1733624

EPI_ISL_1733626

EPI_ISL_1733627

EPI_ISL_1733628

EPI_ISL_1733630

EPI_ISL_1733631

EPI_ISL_1733633

EPI_ISL_1733635

EPI_ISL_1733636

EPI_ISL_1733637

EPI_ISL_1733639

EPI_ISL_1733642

EPI_ISL_1733643

EPI_ISL_1733644

EPI_ISL_1733646

EPI_ISL_1733648

EPI_ISL_1733649

EPI_ISL_1733651

EPI_ISL_1733652

EPI_ISL_1733653

EPI_ISL_1733654

EPI_ISL_1733655

EPI_ISL_1733656

EPI_ISL_1733657

EPI_ISL_1733658

EPI_ISL_1733661

EPI_ISL_1733665

EPI_ISL_1733666

EPI_ISL_1733670

EPI_ISL_1733672

EPI_ISL_1733673

EPI_ISL_1733674

EPI_ISL_1733677

EPI_ISL_1733678

EPI_ISL_1733680

EPI_ISL_1733682

EPI_ISL_1733685

EPI_ISL_1733687

EPI_ISL_1733690

EPI_ISL_1733693

EPI_ISL_1733694

EPI_ISL_1733695

EPI_ISL_1733696

EPI_ISL_1733697

EPI_ISL_1733699

EPI_ISL_1733700

EPI_ISL_1733701

EPI_ISL_1733705

EPI_ISL_1733712

EPI_ISL_1733715

EPI_ISL_1733717

EPI_ISL_1733718

EPI_ISL_1733719

EPI_ISL_1733721

EPI_ISL_1733723

EPI_ISL_1733724

EPI_ISL_1733725

EPI_ISL_1733727

EPI_ISL_1733731

EPI_ISL_1733749

EPI_ISL_1733756

EPI_ISL_1733759

EPI_ISL_1733773

EPI_ISL_1733787

EPI_ISL_1733796

EPI_ISL_1733797

EPI_ISL_1733798

EPI_ISL_1733799

EPI_ISL_1733800

EPI_ISL_1733801

EPI_ISL_1733802

EPI_ISL_1733805

EPI_ISL_1733812

EPI_ISL_1733821

EPI_ISL_1733833

EPI_ISL_1733838

EPI_ISL_1733840

EPI_ISL_1733848

EPI_ISL_1733849

EPI_ISL_1733850

EPI_ISL_1733851

EPI_ISL_1733854

EPI_ISL_1733860

EPI_ISL_1733864

EPI_ISL_1733867

EPI_ISL_1733869

EPI_ISL_1733870

EPI_ISL_1733871

EPI_ISL_1733885

EPI_ISL_1733886

EPI_ISL_1733889

EPI_ISL_1733893

EPI_ISL_1733894

EPI_ISL_1733895

EPI_ISL_1733906

EPI_ISL_1733907

EPI_ISL_1733908

EPI_ISL_1733910

EPI_ISL_1733926

EPI_ISL_1733928

EPI_ISL_1733929

EPI_ISL_1733930

EPI_ISL_1733934

EPI_ISL_1733940

EPI_ISL_1733943

EPI_ISL_1733946

EPI_ISL_1733948

EPI_ISL_1733950

EPI_ISL_1733953

EPI_ISL_1733957

EPI_ISL_1733958

EPI_ISL_1733959

EPI_ISL_1733962

EPI_ISL_1733980

EPI_ISL_1733985

EPI_ISL_1733990

EPI_ISL_1733991

EPI_ISL_1733995

EPI_ISL_1733997

EPI_ISL_1733998

EPI_ISL_1734003

EPI_ISL_1734008

EPI_ISL_1734013

EPI_ISL_1734015

EPI_ISL_1734016

EPI_ISL_1734023

EPI_ISL_1734025

EPI_ISL_1734026

EPI_ISL_1734028

EPI_ISL_1734029

EPI_ISL_1734037

EPI_ISL_1734038

EPI_ISL_1734040

EPI_ISL_1734045

EPI_ISL_1734046

EPI_ISL_1734051

EPI_ISL_1734052

EPI_ISL_1734053

EPI_ISL_1734059

EPI_ISL_1734061

EPI_ISL_1734062

EPI_ISL_1734066

EPI_ISL_1734067

EPI_ISL_1734070

EPI_ISL_1734074

EPI_ISL_1734078

EPI_ISL_1734080

EPI_ISL_1734081

EPI_ISL_1734082

EPI_ISL_1734084

EPI_ISL_1734085

EPI_ISL_1734088

EPI_ISL_1734092

EPI_ISL_1734096

EPI_ISL_1734097

EPI_ISL_1734103

EPI_ISL_1734108

EPI_ISL_1734116

EPI_ISL_1734126

EPI_ISL_1734127

EPI_ISL_1734128

EPI_ISL_1734129

EPI_ISL_1734131

EPI_ISL_1734134

EPI_ISL_1734137

EPI_ISL_1734138

EPI_ISL_1734140

EPI_ISL_1734145

EPI_ISL_1734146

EPI_ISL_1734149

EPI_ISL_1734151

EPI_ISL_1734157

EPI_ISL_1734166

EPI_ISL_1734171

EPI_ISL_1734176

EPI_ISL_1734177

EPI_ISL_1734178

EPI_ISL_1734180

EPI_ISL_1734190

EPI_ISL_1734191

EPI_ISL_1734197

EPI_ISL_1734198

EPI_ISL_1734202

EPI_ISL_1734203

EPI_ISL_1734206

EPI_ISL_1734207

EPI_ISL_1734212

EPI_ISL_1734215

EPI_ISL_1734228

EPI_ISL_1734229

EPI_ISL_1734231

EPI_ISL_1734234

EPI_ISL_1734241

EPI_ISL_1734246

EPI_ISL_1734247

EPI_ISL_1734249

EPI_ISL_1734258

EPI_ISL_1734263

EPI_ISL_1734268

EPI_ISL_1734271

EPI_ISL_1734274

EPI_ISL_1734275

EPI_ISL_1734281

EPI_ISL_1734283

EPI_ISL_1734284

EPI_ISL_1734290

EPI_ISL_1734292

EPI_ISL_1734294

EPI_ISL_1734296

EPI_ISL_1734297

EPI_ISL_1734299

EPI_ISL_1734306

EPI_ISL_1734307

EPI_ISL_1734325

EPI_ISL_1734327

EPI_ISL_1734331

EPI_ISL_1734335

EPI_ISL_1734338

EPI_ISL_1734341

EPI_ISL_1734345

EPI_ISL_1734350

EPI_ISL_1734351

EPI_ISL_1734355

EPI_ISL_1734366

EPI_ISL_1734367

EPI_ISL_1734369

EPI_ISL_1734370

EPI_ISL_1734371

EPI_ISL_1734377

EPI_ISL_1734399

EPI_ISL_1734403

EPI_ISL_1734404

EPI_ISL_1734406

EPI_ISL_1734407

EPI_ISL_1734408

EPI_ISL_1734410

EPI_ISL_1734412

EPI_ISL_1734415

EPI_ISL_1734433

EPI_ISL_1734437

EPI_ISL_1734439

EPI_ISL_1734455

EPI_ISL_1734465

EPI_ISL_1734472

EPI_ISL_1734491

EPI_ISL_1734497

EPI_ISL_1734500

EPI_ISL_1734503

EPI_ISL_1734504

EPI_ISL_1734506

EPI_ISL_1734509

EPI_ISL_1734546

EPI_ISL_1734554

EPI_ISL_1734555

EPI_ISL_1734557

EPI_ISL_1734558

EPI_ISL_1734560

EPI_ISL_1734561

EPI_ISL_1734564

EPI_ISL_1734566

EPI_ISL_1734567

EPI_ISL_1734569

EPI_ISL_1734570

EPI_ISL_1734571

EPI_ISL_1734573

EPI_ISL_1734574

EPI_ISL_1734575

EPI_ISL_1734576

EPI_ISL_1734577

EPI_ISL_1734578

EPI_ISL_1734579

EPI_ISL_1734580

EPI_ISL_1734581

EPI_ISL_1734582

EPI_ISL_1734584

EPI_ISL_1734585

EPI_ISL_1734588

EPI_ISL_1734589

EPI_ISL_1734590

EPI_ISL_1734592

EPI_ISL_1734595

EPI_ISL_1734596

EPI_ISL_1734598

EPI_ISL_1734599

EPI_ISL_1734602

EPI_ISL_1734603

EPI_ISL_1734604

EPI_ISL_1734609

EPI_ISL_1734610

EPI_ISL_1734613

EPI_ISL_1734619

EPI_ISL_1734620

EPI_ISL_1734622

EPI_ISL_1734623

EPI_ISL_1734625

EPI_ISL_1734627

EPI_ISL_1734628

EPI_ISL_1734629

EPI_ISL_1734630

EPI_ISL_1734634

EPI_ISL_1734637

EPI_ISL_1734638

EPI_ISL_1734639

EPI_ISL_1734640

EPI_ISL_1734642

EPI_ISL_1734643

EPI_ISL_1734644

EPI_ISL_1734646

EPI_ISL_1734647

EPI_ISL_1734648

EPI_ISL_1734650

EPI_ISL_1734651

EPI_ISL_1734653

EPI_ISL_1734654

EPI_ISL_1734655

EPI_ISL_1734656

EPI_ISL_1734657

EPI_ISL_1734659

EPI_ISL_1734660

EPI_ISL_1734661

EPI_ISL_1734662

EPI_ISL_1734664

EPI_ISL_1734666

EPI_ISL_1734667

EPI_ISL_1734668

EPI_ISL_1734669

EPI_ISL_1734670

EPI_ISL_1734671

EPI_ISL_1734672

EPI_ISL_1734677

EPI_ISL_1736815

EPI_ISL_1734682

EPI_ISL_1734691

EPI_ISL_1734694

EPI_ISL_1734695

EPI_ISL_1734700

EPI_ISL_1734701

EPI_ISL_1734702

EPI_ISL_1736817

EPI_ISL_1734704

EPI_ISL_1734705

EPI_ISL_1734706

EPI_ISL_1734709

EPI_ISL_1734710

EPI_ISL_1734711

EPI_ISL_1734712

EPI_ISL_1734713

EPI_ISL_1734714

EPI_ISL_1734715

EPI_ISL_1734716

EPI_ISL_1734719

EPI_ISL_1734721

EPI_ISL_1734722

EPI_ISL_1734723

EPI_ISL_1734726

EPI_ISL_1734730

EPI_ISL_1734731

EPI_ISL_1734735

EPI_ISL_1734740

EPI_ISL_1734742

EPI_ISL_1734745

EPI_ISL_1734746

EPI_ISL_1734748

EPI_ISL_1734750

EPI_ISL_1734753

EPI_ISL_1734754

EPI_ISL_1734755

EPI_ISL_1734757

EPI_ISL_1734760

EPI_ISL_1734761

EPI_ISL_1734763

EPI_ISL_1734765

EPI_ISL_1734766

EPI_ISL_1734769

EPI_ISL_1734772

EPI_ISL_1734774

EPI_ISL_1734775

EPI_ISL_1734780

EPI_ISL_1734781

EPI_ISL_1736820

EPI_ISL_1734788

EPI_ISL_1734801

EPI_ISL_1734803

EPI_ISL_1734804

EPI_ISL_1734806

EPI_ISL_1734807

EPI_ISL_1734809

EPI_ISL_1734811

EPI_ISL_1734813

EPI_ISL_1734814

EPI_ISL_1734815

EPI_ISL_1734816

EPI_ISL_1734818

EPI_ISL_1734819

EPI_ISL_1734822

EPI_ISL_1734823

EPI_ISL_1734825

EPI_ISL_1734826

EPI_ISL_1734827

EPI_ISL_1734829

EPI_ISL_1734831

EPI_ISL_1734832

EPI_ISL_1734835

EPI_ISL_1734839

EPI_ISL_1734840

EPI_ISL_1734842

EPI_ISL_1734851

EPI_ISL_1734865

EPI_ISL_1734870

EPI_ISL_1734887

EPI_ISL_1734890

EPI_ISL_1734898

EPI_ISL_1734900

EPI_ISL_1734901

EPI_ISL_1734903

EPI_ISL_1734904

EPI_ISL_1734906

EPI_ISL_1734907

EPI_ISL_1734908

EPI_ISL_1734910

EPI_ISL_1734914

EPI_ISL_1734915

EPI_ISL_1734919

EPI_ISL_1734920

EPI_ISL_1734923

EPI_ISL_1734924

EPI_ISL_1734929

EPI_ISL_1734930

EPI_ISL_1734931

EPI_ISL_1734934

EPI_ISL_1734940

EPI_ISL_1734949

EPI_ISL_1734961

EPI_ISL_1734979

EPI_ISL_1734981

EPI_ISL_1734989

EPI_ISL_1734997

EPI_ISL_1734998

EPI_ISL_1735001

EPI_ISL_1735002

EPI_ISL_1735003

EPI_ISL_1735005

EPI_ISL_1735006

EPI_ISL_1735007

EPI_ISL_1735008

EPI_ISL_1735013

EPI_ISL_1735014

EPI_ISL_1735017

EPI_ISL_1735018

EPI_ISL_1735019

EPI_ISL_1735020

EPI_ISL_1735021

EPI_ISL_1735022

EPI_ISL_1735023

EPI_ISL_1735025

EPI_ISL_1735026

EPI_ISL_1735027

EPI_ISL_1735028

EPI_ISL_1735029

EPI_ISL_1735031

EPI_ISL_1735032

EPI_ISL_1735033

EPI_ISL_1735034

EPI_ISL_1735035

EPI_ISL_1735036

EPI_ISL_1735037

EPI_ISL_1735046

EPI_ISL_1735050

EPI_ISL_1735053

EPI_ISL_1735056

EPI_ISL_1735057

EPI_ISL_1735059

EPI_ISL_1735061

EPI_ISL_1735064

EPI_ISL_1735069

EPI_ISL_1735070

EPI_ISL_1735072

EPI_ISL_1735073

EPI_ISL_1735074

EPI_ISL_1735075

EPI_ISL_1735078

EPI_ISL_1735085

EPI_ISL_1735087

EPI_ISL_1735091

EPI_ISL_1735094

EPI_ISL_1735097

EPI_ISL_1735098

EPI_ISL_1735100

EPI_ISL_1735103

EPI_ISL_1735104

EPI_ISL_1735106

EPI_ISL_1735107

EPI_ISL_1735110

EPI_ISL_1735118

EPI_ISL_1735119

EPI_ISL_1735121

EPI_ISL_1735129

EPI_ISL_1736825

EPI_ISL_1735131

EPI_ISL_1735132

EPI_ISL_1735134

EPI_ISL_1735135

EPI_ISL_1735137

EPI_ISL_1735138

EPI_ISL_1735139

EPI_ISL_1735141

EPI_ISL_1735146

EPI_ISL_1735151

EPI_ISL_1735152

EPI_ISL_1735156

EPI_ISL_1735157

EPI_ISL_1735159

EPI_ISL_1735160

EPI_ISL_1735163

EPI_ISL_1735164

EPI_ISL_1735169

EPI_ISL_1735170

EPI_ISL_1735172

EPI_ISL_1735174

EPI_ISL_1735177

EPI_ISL_1735179

EPI_ISL_1735180

EPI_ISL_1735181

EPI_ISL_1735182

EPI_ISL_1735183

EPI_ISL_1735186

EPI_ISL_1735188

EPI_ISL_1735190

EPI_ISL_1735193

EPI_ISL_1735195

EPI_ISL_1735197

EPI_ISL_1735198

EPI_ISL_1735199

EPI_ISL_1735201

EPI_ISL_1735202

EPI_ISL_1735206

EPI_ISL_1735208

EPI_ISL_1735209

EPI_ISL_1735212

EPI_ISL_1735213

EPI_ISL_1735214

EPI_ISL_1735216

EPI_ISL_1735217

EPI_ISL_1735220

EPI_ISL_1735221

EPI_ISL_1735228

EPI_ISL_1735229

EPI_ISL_1735231

EPI_ISL_1735233

EPI_ISL_1735235

EPI_ISL_1735237

EPI_ISL_1735239

EPI_ISL_1735240

EPI_ISL_1735247

EPI_ISL_1735248

EPI_ISL_1735251

EPI_ISL_1735254

EPI_ISL_1735255

EPI_ISL_1735256

EPI_ISL_1735259

EPI_ISL_1735263

EPI_ISL_1735264

EPI_ISL_1735268

EPI_ISL_1735269

EPI_ISL_1735270

EPI_ISL_1735272

EPI_ISL_1735278

EPI_ISL_1735279

EPI_ISL_1735280

EPI_ISL_1735281

EPI_ISL_1735282

EPI_ISL_1735285

EPI_ISL_1735286

EPI_ISL_1735288

EPI_ISL_1735290

EPI_ISL_1735293

EPI_ISL_1735295

EPI_ISL_1735297

EPI_ISL_1735299

EPI_ISL_1735300

EPI_ISL_1735302

EPI_ISL_1735304

EPI_ISL_1735306

EPI_ISL_1735309

EPI_ISL_1735311

EPI_ISL_1735312

EPI_ISL_1735313

EPI_ISL_1735315

EPI_ISL_1735317

EPI_ISL_1735319

EPI_ISL_1735322

EPI_ISL_1735323

EPI_ISL_1735327

EPI_ISL_1735331

EPI_ISL_1735333

EPI_ISL_1735334

EPI_ISL_1735336

EPI_ISL_1735337

EPI_ISL_1735343

EPI_ISL_1735344

EPI_ISL_1735347

EPI_ISL_1735349

EPI_ISL_1735351

EPI_ISL_1735352

EPI_ISL_1735354

EPI_ISL_1735355

EPI_ISL_1735356

EPI_ISL_1735357

EPI_ISL_1735358

EPI_ISL_1735359

EPI_ISL_1735360

EPI_ISL_1735362

EPI_ISL_1735364

EPI_ISL_1735371

EPI_ISL_1735372

EPI_ISL_1735373

EPI_ISL_1735374

EPI_ISL_1735376

EPI_ISL_1735379

EPI_ISL_1735381

EPI_ISL_1735382

EPI_ISL_1735386

EPI_ISL_1735387

EPI_ISL_1735389

EPI_ISL_1735390

EPI_ISL_1735392

EPI_ISL_1735394

EPI_ISL_1735395

EPI_ISL_1735397

EPI_ISL_1735400

EPI_ISL_1735404

EPI_ISL_1735405

EPI_ISL_1735407

EPI_ISL_1735408

EPI_ISL_1735409

EPI_ISL_1735413

EPI_ISL_1735415

EPI_ISL_1735418

EPI_ISL_1735422

EPI_ISL_1735424

EPI_ISL_1735425

EPI_ISL_1735428

EPI_ISL_1735429

EPI_ISL_1735432

EPI_ISL_1735433

EPI_ISL_1735435

EPI_ISL_1735436

EPI_ISL_1735439

EPI_ISL_1735440

EPI_ISL_1735441

EPI_ISL_1735443

EPI_ISL_1735445

EPI_ISL_1735446

EPI_ISL_1735450

EPI_ISL_1735453

EPI_ISL_1735454

EPI_ISL_1735455

EPI_ISL_1735461

EPI_ISL_1735462

EPI_ISL_1735463

EPI_ISL_1735466

EPI_ISL_1735472

EPI_ISL_1735473

EPI_ISL_1735475

EPI_ISL_1735478

EPI_ISL_1735482

EPI_ISL_1735484

EPI_ISL_1735485

EPI_ISL_1735487

EPI_ISL_1735488

EPI_ISL_1735490

EPI_ISL_1735491

EPI_ISL_1735493

EPI_ISL_1735494

EPI_ISL_1735496

EPI_ISL_1735497

EPI_ISL_1735499

EPI_ISL_1735501

EPI_ISL_1735502

EPI_ISL_1735504

EPI_ISL_1735505

EPI_ISL_1735507

EPI_ISL_1735508

EPI_ISL_1735510

EPI_ISL_1735512

EPI_ISL_1735516

EPI_ISL_1735517

EPI_ISL_1735518

EPI_ISL_1735524

EPI_ISL_1735527

EPI_ISL_1735528

EPI_ISL_1735529

EPI_ISL_1735531

EPI_ISL_1735532

EPI_ISL_1735534

EPI_ISL_1735543

EPI_ISL_1735544

EPI_ISL_1735545

EPI_ISL_1735549

EPI_ISL_1735550

EPI_ISL_1735551

EPI_ISL_1735552

EPI_ISL_1735553

EPI_ISL_1735555

EPI_ISL_1735556

EPI_ISL_1735557

EPI_ISL_1735559

EPI_ISL_1735560

EPI_ISL_1735562

EPI_ISL_1735563

EPI_ISL_1735564

EPI_ISL_1735565

EPI_ISL_1735566

EPI_ISL_1735567

EPI_ISL_1735569

EPI_ISL_1735570

EPI_ISL_1735576

EPI_ISL_1735578

EPI_ISL_1735581

EPI_ISL_1735583

EPI_ISL_1735584

EPI_ISL_1735586

EPI_ISL_1735591

EPI_ISL_1735594

EPI_ISL_1735598

EPI_ISL_1735599

EPI_ISL_1735600

EPI_ISL_1735601

EPI_ISL_1735606

EPI_ISL_1735607

EPI_ISL_1735608

EPI_ISL_1735611

EPI_ISL_1735612

EPI_ISL_1735616

EPI_ISL_1735618

EPI_ISL_1735619

EPI_ISL_1735620

EPI_ISL_1735625

EPI_ISL_1735626

EPI_ISL_1735629

EPI_ISL_1735632

EPI_ISL_1735633

EPI_ISL_1735635

EPI_ISL_1735636

EPI_ISL_1735637

EPI_ISL_1735638

EPI_ISL_1735642

EPI_ISL_1735643

EPI_ISL_1735644

EPI_ISL_1735645

EPI_ISL_1735650

EPI_ISL_1735651

EPI_ISL_1735652

EPI_ISL_1735653

EPI_ISL_1735655

EPI_ISL_1735656

EPI_ISL_1735657

EPI_ISL_1735658

EPI_ISL_1735660

EPI_ISL_1735661

EPI_ISL_1735663

EPI_ISL_1735665

EPI_ISL_1735666

EPI_ISL_1735667

EPI_ISL_1735668

EPI_ISL_1735671

EPI_ISL_1735672

EPI_ISL_1735673

EPI_ISL_1735674

EPI_ISL_1735678

EPI_ISL_1735680

EPI_ISL_1735683

EPI_ISL_1735684

EPI_ISL_1735685

EPI_ISL_1735688

EPI_ISL_1735690

EPI_ISL_1735693

EPI_ISL_1735694

EPI_ISL_1735700

EPI_ISL_1735702

EPI_ISL_1735705

EPI_ISL_1735709

EPI_ISL_1735710

EPI_ISL_1735711

EPI_ISL_1735718

EPI_ISL_1735719

EPI_ISL_1735720

EPI_ISL_1735723

EPI_ISL_1735724

EPI_ISL_1735725

EPI_ISL_1735727

EPI_ISL_1735728

EPI_ISL_1735730

EPI_ISL_1735731

EPI_ISL_1735732

EPI_ISL_1735733

EPI_ISL_1735734

EPI_ISL_1735736

EPI_ISL_1735738

EPI_ISL_1735740

EPI_ISL_1735743

EPI_ISL_1735746

EPI_ISL_1735748

EPI_ISL_1735749

EPI_ISL_1735755

EPI_ISL_1735761

EPI_ISL_1735762

EPI_ISL_1735765

EPI_ISL_1735767

EPI_ISL_1735768

EPI_ISL_1735769

EPI_ISL_1735770

EPI_ISL_1735771

EPI_ISL_1735772

EPI_ISL_1735774

EPI_ISL_1735775

EPI_ISL_1735783

EPI_ISL_1735784

EPI_ISL_1735785

EPI_ISL_1735786

EPI_ISL_1735788

EPI_ISL_1735792

EPI_ISL_1735795

EPI_ISL_1735797

EPI_ISL_1735798

EPI_ISL_1735799

EPI_ISL_1735801

EPI_ISL_1735802

EPI_ISL_1735803

EPI_ISL_1735806

EPI_ISL_1735807

EPI_ISL_1735808

EPI_ISL_1735811

EPI_ISL_1735815

EPI_ISL_1735817

EPI_ISL_1735823

EPI_ISL_1735824

EPI_ISL_1735826

EPI_ISL_1735829

EPI_ISL_1735830

EPI_ISL_1735832

EPI_ISL_1735837

EPI_ISL_1735840

EPI_ISL_1735841

EPI_ISL_1735842

EPI_ISL_1735844

EPI_ISL_1735846

EPI_ISL_1735848

EPI_ISL_1735849

EPI_ISL_1735851

EPI_ISL_1735852

EPI_ISL_1735853

EPI_ISL_1735854

EPI_ISL_1735857

EPI_ISL_1735859

EPI_ISL_1735860

EPI_ISL_1735863

EPI_ISL_1735865

EPI_ISL_1735871

EPI_ISL_1735873

EPI_ISL_1735880

EPI_ISL_1735881

EPI_ISL_1735886

EPI_ISL_1735888

EPI_ISL_1735893

EPI_ISL_1735895

EPI_ISL_1735896

EPI_ISL_1735902

EPI_ISL_1735903

EPI_ISL_1735904

EPI_ISL_1735906

EPI_ISL_1735907

EPI_ISL_1735908

EPI_ISL_1735909

EPI_ISL_1735911

EPI_ISL_1735912

EPI_ISL_1735913

EPI_ISL_1735914

EPI_ISL_1735915

EPI_ISL_1735916

EPI_ISL_1735917

EPI_ISL_1735920

EPI_ISL_1735921

EPI_ISL_1735923

EPI_ISL_1735924

EPI_ISL_1735925

EPI_ISL_1735926

EPI_ISL_1735927

EPI_ISL_1735931

EPI_ISL_1735934

EPI_ISL_1735935

EPI_ISL_1735938

EPI_ISL_1735941

EPI_ISL_1735944

EPI_ISL_1735947

EPI_ISL_1735948

EPI_ISL_1735950

EPI_ISL_1735951

EPI_ISL_1735954

EPI_ISL_1735956

EPI_ISL_1735957

EPI_ISL_1735958

EPI_ISL_1735959

EPI_ISL_1735960

EPI_ISL_1735961

EPI_ISL_1735963

EPI_ISL_1735964

EPI_ISL_1735965

EPI_ISL_1735966

EPI_ISL_1735969

EPI_ISL_1735970

EPI_ISL_1735971

EPI_ISL_1735973

EPI_ISL_1735975

EPI_ISL_1735976

EPI_ISL_1735978

EPI_ISL_1735981

EPI_ISL_1735983

EPI_ISL_1735984

EPI_ISL_1735986

EPI_ISL_1735988

EPI_ISL_1735990

EPI_ISL_1735992

EPI_ISL_1735993

EPI_ISL_1735994

EPI_ISL_1735995

EPI_ISL_1735996

EPI_ISL_1735998

EPI_ISL_1736003

EPI_ISL_1736008

EPI_ISL_1736011

EPI_ISL_1736016

EPI_ISL_1736018

EPI_ISL_1736019

EPI_ISL_1736022

EPI_ISL_1736026

EPI_ISL_1736027

EPI_ISL_1736028

EPI_ISL_1736032

EPI_ISL_1736033

EPI_ISL_1736034

EPI_ISL_1736035

EPI_ISL_1736040

EPI_ISL_1736042

EPI_ISL_1736043

EPI_ISL_1736046

EPI_ISL_1736047

EPI_ISL_1736048

EPI_ISL_1736049

EPI_ISL_1736053

EPI_ISL_1736054

EPI_ISL_1736055

EPI_ISL_1736056

EPI_ISL_1736059

EPI_ISL_1736061

EPI_ISL_1736062

EPI_ISL_1736064

EPI_ISL_1736065

EPI_ISL_1736068

EPI_ISL_1736069

EPI_ISL_1736070

EPI_ISL_1736071

EPI_ISL_1736072

EPI_ISL_1736073

EPI_ISL_1736074

EPI_ISL_1736075

EPI_ISL_1736077

EPI_ISL_1736082

EPI_ISL_1736083

EPI_ISL_1736086

EPI_ISL_1736087

EPI_ISL_1736088

EPI_ISL_1736089

EPI_ISL_1736093

EPI_ISL_1736094

EPI_ISL_1736098

EPI_ISL_1736099

EPI_ISL_1736103

EPI_ISL_1736104

EPI_ISL_1736105

EPI_ISL_1736106

EPI_ISL_1736109

EPI_ISL_1736110

EPI_ISL_1736111

EPI_ISL_1736112

EPI_ISL_1736113

EPI_ISL_1736114

EPI_ISL_1736118

EPI_ISL_1736121

EPI_ISL_1736124

EPI_ISL_1736125

EPI_ISL_1736128

EPI_ISL_1736130

EPI_ISL_1736131

EPI_ISL_1736132

EPI_ISL_1736136

EPI_ISL_1736139

EPI_ISL_1736140

EPI_ISL_1736145

EPI_ISL_1736150

EPI_ISL_1736153

EPI_ISL_1736155

EPI_ISL_1736157

EPI_ISL_1736159

EPI_ISL_1736160

EPI_ISL_1736163

EPI_ISL_1736165

EPI_ISL_1736166

EPI_ISL_1736168

EPI_ISL_1736171

EPI_ISL_1736172

EPI_ISL_1736173

EPI_ISL_1736174

EPI_ISL_1736175

EPI_ISL_1736176

EPI_ISL_1736177

EPI_ISL_1736179

EPI_ISL_1736186

EPI_ISL_1736187

EPI_ISL_1736188

EPI_ISL_1736190

EPI_ISL_1736191

EPI_ISL_1736192

EPI_ISL_1736195

EPI_ISL_1736200

EPI_ISL_1736201

EPI_ISL_1736204

EPI_ISL_1736206

EPI_ISL_1736208

EPI_ISL_1736210

EPI_ISL_1736213

EPI_ISL_1736219

EPI_ISL_1736225

EPI_ISL_1736227

EPI_ISL_1736228

EPI_ISL_1736232

EPI_ISL_1736236

EPI_ISL_1736241

EPI_ISL_1736243

EPI_ISL_1736246

EPI_ISL_1736248

EPI_ISL_1736250

EPI_ISL_1736253

EPI_ISL_1736254

EPI_ISL_1736257

EPI_ISL_1736258

EPI_ISL_1736263

EPI_ISL_1736264

EPI_ISL_1736266

EPI_ISL_1736267

EPI_ISL_1736269

EPI_ISL_1736276

EPI_ISL_1736277

EPI_ISL_1736278

EPI_ISL_1736279

EPI_ISL_1736282

EPI_ISL_1736283

EPI_ISL_1736289

EPI_ISL_1736298

EPI_ISL_1736299

EPI_ISL_1736301

EPI_ISL_1736303

EPI_ISL_1736307

EPI_ISL_1736309

EPI_ISL_1736310

EPI_ISL_1736313

EPI_ISL_1736320

EPI_ISL_1736321

EPI_ISL_1736325

EPI_ISL_1736326

EPI_ISL_1736334

EPI_ISL_1736337

EPI_ISL_1736338

EPI_ISL_1736340

EPI_ISL_1736343

EPI_ISL_1736346

EPI_ISL_1736347

EPI_ISL_1736350

EPI_ISL_1736351

EPI_ISL_1736352

EPI_ISL_1736353

EPI_ISL_1736354

EPI_ISL_1736360

EPI_ISL_1736363

EPI_ISL_1736364

EPI_ISL_1736367

EPI_ISL_1736371

EPI_ISL_1736372

EPI_ISL_1736374

EPI_ISL_1736376

EPI_ISL_1736377

EPI_ISL_1736379

EPI_ISL_1736380

EPI_ISL_1736382

EPI_ISL_1736383

EPI_ISL_1736384

EPI_ISL_1736385

EPI_ISL_1736387

EPI_ISL_1736388

EPI_ISL_1736390

EPI_ISL_1736391

EPI_ISL_1736393

EPI_ISL_1736396

EPI_ISL_1736397

EPI_ISL_1736402

EPI_ISL_1736403

EPI_ISL_1736404

EPI_ISL_1736406

EPI_ISL_1736407

EPI_ISL_1736411

EPI_ISL_1736413

EPI_ISL_1736414

EPI_ISL_1736415

EPI_ISL_1736418

EPI_ISL_1736421

EPI_ISL_1736424

EPI_ISL_1736426

EPI_ISL_1736429

EPI_ISL_1736431

EPI_ISL_1736436

EPI_ISL_1736440

EPI_ISL_1736443

EPI_ISL_1736444

EPI_ISL_1736445

EPI_ISL_1736446

EPI_ISL_1736447

EPI_ISL_1736449

EPI_ISL_1736451

EPI_ISL_1736453

EPI_ISL_1736455

EPI_ISL_1736457

EPI_ISL_1736461

EPI_ISL_1736469

EPI_ISL_1736471

EPI_ISL_1736473

EPI_ISL_1736477

EPI_ISL_1736481

EPI_ISL_1736483

EPI_ISL_1736484

EPI_ISL_1736486

EPI_ISL_1736487

EPI_ISL_1736489

EPI_ISL_1736490

EPI_ISL_1736493

EPI_ISL_1736500

EPI_ISL_1736501

EPI_ISL_1736503

EPI_ISL_1736507

EPI_ISL_1736510

EPI_ISL_1736513

EPI_ISL_1736516

EPI_ISL_1736518

EPI_ISL_1736523

EPI_ISL_1736530

EPI_ISL_1736531

EPI_ISL_1736533

EPI_ISL_1736535

EPI_ISL_1736536

EPI_ISL_1736539

EPI_ISL_1736540

EPI_ISL_1736541

EPI_ISL_1736542

EPI_ISL_1736545

EPI_ISL_1736550

EPI_ISL_1736553

EPI_ISL_1736554

EPI_ISL_1736555

EPI_ISL_1736556

EPI_ISL_1736558

EPI_ISL_1736559

EPI_ISL_1736560

EPI_ISL_1736563

EPI_ISL_1736564

EPI_ISL_1736565

EPI_ISL_1736571

EPI_ISL_1736573

EPI_ISL_1736577

EPI_ISL_1736578

EPI_ISL_1736584

EPI_ISL_1736585

EPI_ISL_1736587

EPI_ISL_1736592

EPI_ISL_1736595

EPI_ISL_1736601

EPI_ISL_1736603

EPI_ISL_1736606

EPI_ISL_1736607

EPI_ISL_1736608

EPI_ISL_1736611

EPI_ISL_1736612

EPI_ISL_1736614

EPI_ISL_1736615

EPI_ISL_1736619

EPI_ISL_1736621

EPI_ISL_1736622

EPI_ISL_1736627

EPI_ISL_1736632

EPI_ISL_1736633

EPI_ISL_1736636

EPI_ISL_1736637

EPI_ISL_1736641

EPI_ISL_1736644

EPI_ISL_1736646

EPI_ISL_1736647

EPI_ISL_1736650

EPI_ISL_1736651

EPI_ISL_1736652

EPI_ISL_1736653

EPI_ISL_1736654

EPI_ISL_1736656

EPI_ISL_1736658

EPI_ISL_1736660

EPI_ISL_1736664

EPI_ISL_1736667

EPI_ISL_1736670

EPI_ISL_1736671

EPI_ISL_1736672

EPI_ISL_1736680

EPI_ISL_1736683

EPI_ISL_1736686

EPI_ISL_1736687

EPI_ISL_1736690

EPI_ISL_1736691

EPI_ISL_1736694

EPI_ISL_1736696

EPI_ISL_1736698

EPI_ISL_1736700

EPI_ISL_1736701

EPI_ISL_1736706

EPI_ISL_1736709

EPI_ISL_1736713

EPI_ISL_1736716

EPI_ISL_1736717

EPI_ISL_1736720

EPI_ISL_1736722

EPI_ISL_1736724

EPI_ISL_1736730

EPI_ISL_1736732

EPI_ISL_1736737

EPI_ISL_1736741

EPI_ISL_1736742

EPI_ISL_1736746

EPI_ISL_1736749

EPI_ISL_1736754

EPI_ISL_1736759

EPI_ISL_1736760

EPI_ISL_1736764

EPI_ISL_1736766

EPI_ISL_1736768

EPI_ISL_1736769

EPI_ISL_1736771

EPI_ISL_1736774

EPI_ISL_1736775

EPI_ISL_1736776

EPI_ISL_1736777

EPI_ISL_1736778

EPI_ISL_1736779

EPI_ISL_1736785

EPI_ISL_1736791

EPI_ISL_1736792

EPI_ISL_1736793

EPI_ISL_1736794

EPI_ISL_1736795

EPI_ISL_1736797

EPI_ISL_1736798

EPI_ISL_1736800

EPI_ISL_1736802

EPI_ISL_1736803

EPI_ISL_1736828

EPI_ISL_1736830

EPI_ISL_1736833

EPI_ISL_1736834

EPI_ISL_1736835

EPI_ISL_1736836

EPI_ISL_1736838

EPI_ISL_1736839

EPI_ISL_1736840

EPI_ISL_1736841

EPI_ISL_1736842

EPI_ISL_1736843

EPI_ISL_1736846

EPI_ISL_1736848

EPI_ISL_1736853

EPI_ISL_1736854

EPI_ISL_1736857

EPI_ISL_1736858

EPI_ISL_1736862

EPI_ISL_1736864

EPI_ISL_1736865

EPI_ISL_1736866

EPI_ISL_1736868

EPI_ISL_1736869

EPI_ISL_1736871

EPI_ISL_1736872

EPI_ISL_1736873

EPI_ISL_1736874

EPI_ISL_1736878

EPI_ISL_1736881

EPI_ISL_1736882

EPI_ISL_1736884

EPI_ISL_1736885

EPI_ISL_1736887

EPI_ISL_1736888

EPI_ISL_1736894

EPI_ISL_1736895

EPI_ISL_1736907

EPI_ISL_1736909

EPI_ISL_1736916

EPI_ISL_1736917

EPI_ISL_1736919

EPI_ISL_1736920

EPI_ISL_1736922

EPI_ISL_1736923

EPI_ISL_1736924

EPI_ISL_1736928

EPI_ISL_1736929

EPI_ISL_1736932

EPI_ISL_1736937

EPI_ISL_1736938

EPI_ISL_1736939

EPI_ISL_1736940

EPI_ISL_1736941

EPI_ISL_1736945

EPI_ISL_1736946

EPI_ISL_1736947

EPI_ISL_1736950

EPI_ISL_1736953

EPI_ISL_1736959

EPI_ISL_1736961

EPI_ISL_1736964

EPI_ISL_1736965

EPI_ISL_1736966

EPI_ISL_1736970

EPI_ISL_1736971

EPI_ISL_1736975

EPI_ISL_1736976

EPI_ISL_1736977

EPI_ISL_1736978

EPI_ISL_1736979

EPI_ISL_1736981

EPI_ISL_1736982

EPI_ISL_1736986

EPI_ISL_1736987

EPI_ISL_1736988

EPI_ISL_1736989

EPI_ISL_1736990

EPI_ISL_1736991

EPI_ISL_1736992

EPI_ISL_1736995

EPI_ISL_1736999

EPI_ISL_1737000

EPI_ISL_1737001

EPI_ISL_1737002

EPI_ISL_1737003

EPI_ISL_1737004

EPI_ISL_1737009

EPI_ISL_1737012

EPI_ISL_1737014

EPI_ISL_1737015

EPI_ISL_1737018

EPI_ISL_1737022

EPI_ISL_1737028

EPI_ISL_1737031

EPI_ISL_1737033

EPI_ISL_1737034

EPI_ISL_1737039

EPI_ISL_1737041

EPI_ISL_1737042

EPI_ISL_1737045

EPI_ISL_1737046

EPI_ISL_1737048

EPI_ISL_1737049

EPI_ISL_1737055

EPI_ISL_1737056

EPI_ISL_1737057

EPI_ISL_1737058

EPI_ISL_1737062

EPI_ISL_1737064

EPI_ISL_1737065

EPI_ISL_1737066

EPI_ISL_1737067

EPI_ISL_1737070

EPI_ISL_1737071

EPI_ISL_1737072

EPI_ISL_1737076

EPI_ISL_1737077

EPI_ISL_1737081

EPI_ISL_1737083

EPI_ISL_1737084

EPI_ISL_1737085

EPI_ISL_1737086

EPI_ISL_1737088

EPI_ISL_1737090

EPI_ISL_1737091

EPI_ISL_1737092

EPI_ISL_1737097

EPI_ISL_1737099

EPI_ISL_1737100

EPI_ISL_1737101

EPI_ISL_1737102

EPI_ISL_1737105

EPI_ISL_1737108

EPI_ISL_1737109

EPI_ISL_1737110

EPI_ISL_1737114

EPI_ISL_1737115

EPI_ISL_1737116

EPI_ISL_1737118

EPI_ISL_1737119

EPI_ISL_1737120

EPI_ISL_1737122

EPI_ISL_1737124

EPI_ISL_1737125

EPI_ISL_1737127

EPI_ISL_1737128

EPI_ISL_1737131

EPI_ISL_1737132

EPI_ISL_1737134

EPI_ISL_1737137

EPI_ISL_1737138

EPI_ISL_1737139

EPI_ISL_1737141

EPI_ISL_1737143

EPI_ISL_1737144

EPI_ISL_1737147

EPI_ISL_1737148

EPI_ISL_1737149

EPI_ISL_1737150

EPI_ISL_1737153

EPI_ISL_1737155

EPI_ISL_1737161

EPI_ISL_1737162

EPI_ISL_1737164

EPI_ISL_1737169

EPI_ISL_1737170

EPI_ISL_1737173

EPI_ISL_1737175

EPI_ISL_1737176

EPI_ISL_1737177

EPI_ISL_1737178

EPI_ISL_1737180

EPI_ISL_1737181

EPI_ISL_1737186

EPI_ISL_1737187

EPI_ISL_1737189

EPI_ISL_1737191

EPI_ISL_1737192

EPI_ISL_1737197

EPI_ISL_1737199

EPI_ISL_1737201

EPI_ISL_1737202

EPI_ISL_1737203

EPI_ISL_1737205

EPI_ISL_1737206

EPI_ISL_1737208

EPI_ISL_1737209

EPI_ISL_1737210

EPI_ISL_1737214

EPI_ISL_1737218

EPI_ISL_1737223

EPI_ISL_1737226

EPI_ISL_1737228

EPI_ISL_1737229

EPI_ISL_1737230

EPI_ISL_1737231

EPI_ISL_1737232

EPI_ISL_1737234

EPI_ISL_1737236

EPI_ISL_1737240

EPI_ISL_1737241

EPI_ISL_1737242

EPI_ISL_1737244

EPI_ISL_1737246

EPI_ISL_1737248

EPI_ISL_1737255

EPI_ISL_1737256

EPI_ISL_1737257

EPI_ISL_1737259

EPI_ISL_1737261

EPI_ISL_1737270

EPI_ISL_1737271

EPI_ISL_1737272

EPI_ISL_1737275

EPI_ISL_1737277

EPI_ISL_1737287

EPI_ISL_1737288

EPI_ISL_1737293

EPI_ISL_1737295

EPI_ISL_1737296

EPI_ISL_1737297

EPI_ISL_1737304

EPI_ISL_1737310

EPI_ISL_1737313

EPI_ISL_1737314

EPI_ISL_1737317

EPI_ISL_1737318

EPI_ISL_1737322

EPI_ISL_1737324

EPI_ISL_1737327

EPI_ISL_1737328

EPI_ISL_1737330

EPI_ISL_1737333

EPI_ISL_1737335

EPI_ISL_1737336

EPI_ISL_1737339

EPI_ISL_1737346

EPI_ISL_1737347

EPI_ISL_1737348

EPI_ISL_1737351

EPI_ISL_1737354

EPI_ISL_1737355

EPI_ISL_1737356

EPI_ISL_1737357

EPI_ISL_1737360

EPI_ISL_1737361

EPI_ISL_1737365

EPI_ISL_1737366

EPI_ISL_1737369

EPI_ISL_1737371

EPI_ISL_1737372

EPI_ISL_1737375

EPI_ISL_1737382

EPI_ISL_1737383

EPI_ISL_1737390

EPI_ISL_1737391

EPI_ISL_1737393

EPI_ISL_1737394

EPI_ISL_1737395

EPI_ISL_1737396

EPI_ISL_1737397

EPI_ISL_1737398

EPI_ISL_1737403

EPI_ISL_1737409

EPI_ISL_1737410

EPI_ISL_1737412

EPI_ISL_1737415

EPI_ISL_1737418

EPI_ISL_1737425

EPI_ISL_1737428

EPI_ISL_1737435

EPI_ISL_1737436

EPI_ISL_1737445

EPI_ISL_1737446

EPI_ISL_1737447

EPI_ISL_1737449

EPI_ISL_1737450

EPI_ISL_1737451

EPI_ISL_1737460

EPI_ISL_1737461

EPI_ISL_1737470

EPI_ISL_1737471

EPI_ISL_1737475

EPI_ISL_1737478

EPI_ISL_1737480

EPI_ISL_1737482

EPI_ISL_1737483

EPI_ISL_1737486

EPI_ISL_1737491

EPI_ISL_1737493

EPI_ISL_1737503

EPI_ISL_1737507

EPI_ISL_1737508

EPI_ISL_1737509

EPI_ISL_1737510

EPI_ISL_1737517

EPI_ISL_1737518

EPI_ISL_1737522

EPI_ISL_1737523

EPI_ISL_1737524

EPI_ISL_1737525

EPI_ISL_1737526

EPI_ISL_1737528

EPI_ISL_1737530

EPI_ISL_1737534

EPI_ISL_1737537

EPI_ISL_1737539

EPI_ISL_1737540

EPI_ISL_1737541

EPI_ISL_1737542

EPI_ISL_1737544

EPI_ISL_1737545

EPI_ISL_1737546

EPI_ISL_1737547

EPI_ISL_1737548

EPI_ISL_1737549

EPI_ISL_1737550

EPI_ISL_1737556

EPI_ISL_1737559

EPI_ISL_1737560

EPI_ISL_1737561

EPI_ISL_1737562

EPI_ISL_1737564

EPI_ISL_1737565

EPI_ISL_1737569

EPI_ISL_1737572

EPI_ISL_1737574

EPI_ISL_1737578

EPI_ISL_1737580

EPI_ISL_1737581

EPI_ISL_1737582

EPI_ISL_1737583

EPI_ISL_1737584

EPI_ISL_1737589

EPI_ISL_1737595

EPI_ISL_1737601

EPI_ISL_1737602

EPI_ISL_1737603

EPI_ISL_1737605

EPI_ISL_1737606

EPI_ISL_1737608

EPI_ISL_1737610

EPI_ISL_1737613

EPI_ISL_1737615

EPI_ISL_1737617

EPI_ISL_1737618

EPI_ISL_1737619

EPI_ISL_1737620

EPI_ISL_1737623

EPI_ISL_1737624

EPI_ISL_1737627

EPI_ISL_1737630

EPI_ISL_1737631

EPI_ISL_1737632

EPI_ISL_1737634

EPI_ISL_1737635

EPI_ISL_1737638

EPI_ISL_1737642

EPI_ISL_1737643

EPI_ISL_1737644

EPI_ISL_1737646

EPI_ISL_1737647

EPI_ISL_1737648

EPI_ISL_1737649

EPI_ISL_1737651

EPI_ISL_1737654

EPI_ISL_1737655

EPI_ISL_1737659

EPI_ISL_1737661

EPI_ISL_1737663

EPI_ISL_1737664

EPI_ISL_1737665

EPI_ISL_1737666

EPI_ISL_1737667

EPI_ISL_1737668

EPI_ISL_1737674

EPI_ISL_1737676

EPI_ISL_1737681

EPI_ISL_1737682

EPI_ISL_1737683

EPI_ISL_1737685

EPI_ISL_1737686

EPI_ISL_1737687

EPI_ISL_1737690

EPI_ISL_1737693

EPI_ISL_1737694

EPI_ISL_1737695

EPI_ISL_1737696

EPI_ISL_1737698

EPI_ISL_1737699

EPI_ISL_1737700

EPI_ISL_1737701

EPI_ISL_1737707

EPI_ISL_1737708

EPI_ISL_1737711

EPI_ISL_1737712

EPI_ISL_1737713

EPI_ISL_1737717

EPI_ISL_1737720

EPI_ISL_1737721

EPI_ISL_1737722

EPI_ISL_1737724

EPI_ISL_1737725

EPI_ISL_1737726

EPI_ISL_1737727

EPI_ISL_1737728

EPI_ISL_1737729

EPI_ISL_1737730

EPI_ISL_1737731

EPI_ISL_1737733

EPI_ISL_1737735

EPI_ISL_1737736

EPI_ISL_1737738

EPI_ISL_1737744

EPI_ISL_1737746

EPI_ISL_1737751

EPI_ISL_1737754

EPI_ISL_1737755

EPI_ISL_1737757

EPI_ISL_1737759

EPI_ISL_1737761

EPI_ISL_1737764

EPI_ISL_1737766

EPI_ISL_1737767

EPI_ISL_1737768

EPI_ISL_1737769

EPI_ISL_1737770

EPI_ISL_1737772

EPI_ISL_1737773

EPI_ISL_1737778

EPI_ISL_1737779

EPI_ISL_1737783

EPI_ISL_1737786

EPI_ISL_1737788

EPI_ISL_1737793

EPI_ISL_1737794

EPI_ISL_1737799

EPI_ISL_1737800

EPI_ISL_1737804

EPI_ISL_1737809

EPI_ISL_1737810

EPI_ISL_1737814

EPI_ISL_1737815

EPI_ISL_1737817

EPI_ISL_1737818

EPI_ISL_1737820

EPI_ISL_1737824

EPI_ISL_1737826

EPI_ISL_1737827

EPI_ISL_1737833

EPI_ISL_1737835

EPI_ISL_1737836

EPI_ISL_1737838

EPI_ISL_1737842

EPI_ISL_1737843

EPI_ISL_1737844

EPI_ISL_1737846

EPI_ISL_1737847

EPI_ISL_1737848

EPI_ISL_1737849

EPI_ISL_1737852

EPI_ISL_1737858

EPI_ISL_1737859

EPI_ISL_1737860

EPI_ISL_1737862

EPI_ISL_1737865

EPI_ISL_1737867

EPI_ISL_1737868

EPI_ISL_1737870

EPI_ISL_1737871

EPI_ISL_1737873

EPI_ISL_1737874

EPI_ISL_1737875

EPI_ISL_1737878

EPI_ISL_1737883

EPI_ISL_1737884

EPI_ISL_1737885

EPI_ISL_1737886

EPI_ISL_1737887

EPI_ISL_1737896

EPI_ISL_1737897

EPI_ISL_1737899

EPI_ISL_1737900

EPI_ISL_1737901

EPI_ISL_1737905

EPI_ISL_1737906

EPI_ISL_1737908

EPI_ISL_1737911

EPI_ISL_1737912

EPI_ISL_1737915

EPI_ISL_1737916

EPI_ISL_1737921

EPI_ISL_1737924

EPI_ISL_1737925

EPI_ISL_1737927

EPI_ISL_1737928

EPI_ISL_1737935

EPI_ISL_1737936

EPI_ISL_1737941

EPI_ISL_1737942

EPI_ISL_1737943

EPI_ISL_1737944

EPI_ISL_1737949

EPI_ISL_1737950

EPI_ISL_1737951

EPI_ISL_1737953

EPI_ISL_1737954

EPI_ISL_1737962

EPI_ISL_1737963

EPI_ISL_1737964

EPI_ISL_1737970

EPI_ISL_1737971

EPI_ISL_1737972

EPI_ISL_1737973

EPI_ISL_1737974

EPI_ISL_1737975

EPI_ISL_1737977

EPI_ISL_1737979

EPI_ISL_1737980

EPI_ISL_1737983

EPI_ISL_1737984

EPI_ISL_1737990

EPI_ISL_1737991

EPI_ISL_1737992

EPI_ISL_1737995

EPI_ISL_1737997

EPI_ISL_1737998

EPI_ISL_1737999

EPI_ISL_1738000

EPI_ISL_1738001

EPI_ISL_1738002

EPI_ISL_1738003

EPI_ISL_1738004

EPI_ISL_1738006

EPI_ISL_1738007

EPI_ISL_1738008

EPI_ISL_1738009

EPI_ISL_1738010

EPI_ISL_1738011

EPI_ISL_1738012

EPI_ISL_1738013

EPI_ISL_1738014

EPI_ISL_1738015

EPI_ISL_1738016

EPI_ISL_1738020

EPI_ISL_1738022

EPI_ISL_1738023

EPI_ISL_1738024

EPI_ISL_1738025

EPI_ISL_1738026

EPI_ISL_1738030

EPI_ISL_1738032

EPI_ISL_1738034

EPI_ISL_1738035

EPI_ISL_1738036

EPI_ISL_1738039

EPI_ISL_1738042

EPI_ISL_1738044

EPI_ISL_1738047

EPI_ISL_1738048

EPI_ISL_1738049

EPI_ISL_1738050

EPI_ISL_1738052

EPI_ISL_1738053

EPI_ISL_1738056

EPI_ISL_1738057

EPI_ISL_1738059

EPI_ISL_1738060

EPI_ISL_1738061

EPI_ISL_1738063

EPI_ISL_1738066

EPI_ISL_1738068

EPI_ISL_1738069

EPI_ISL_1738071

EPI_ISL_1738072

EPI_ISL_1738079

EPI_ISL_1738081

EPI_ISL_1738084

EPI_ISL_1738090

EPI_ISL_1738091

EPI_ISL_1738095

EPI_ISL_1738096

EPI_ISL_1738097

EPI_ISL_1738099

EPI_ISL_1738101

EPI_ISL_1738102

EPI_ISL_1738103

EPI_ISL_1738104

EPI_ISL_1738105

EPI_ISL_1738107

EPI_ISL_1738109

EPI_ISL_1738110

EPI_ISL_1738111

EPI_ISL_1738112

EPI_ISL_1738113

EPI_ISL_1738114

EPI_ISL_1738117

EPI_ISL_1738120

EPI_ISL_1738121

EPI_ISL_1738122

EPI_ISL_1738124

EPI_ISL_1738125

EPI_ISL_1738127

EPI_ISL_1738130

EPI_ISL_1738131

EPI_ISL_1738132

EPI_ISL_1738135

EPI_ISL_1738136

EPI_ISL_1738139

EPI_ISL_1738140

EPI_ISL_1738141

EPI_ISL_1738142

EPI_ISL_1738143

EPI_ISL_1738144

EPI_ISL_1738146

EPI_ISL_1738147

EPI_ISL_1738148

EPI_ISL_1738149

EPI_ISL_1738150

EPI_ISL_1738154

EPI_ISL_1738156

EPI_ISL_1738159

EPI_ISL_1738162

EPI_ISL_1738164

EPI_ISL_1738165

EPI_ISL_1738166

EPI_ISL_1738167

EPI_ISL_1738171

EPI_ISL_1738172

EPI_ISL_1738173

EPI_ISL_1738175

EPI_ISL_1738176

EPI_ISL_1738177

EPI_ISL_1738179

EPI_ISL_1738180

EPI_ISL_1738181

EPI_ISL_1738182

EPI_ISL_1738183

EPI_ISL_1738185

EPI_ISL_1738186

EPI_ISL_1738190

EPI_ISL_1738191

EPI_ISL_1738192

EPI_ISL_1738193

EPI_ISL_1738195

EPI_ISL_1738198

EPI_ISL_1738199

EPI_ISL_1738200

EPI_ISL_1738202

EPI_ISL_1738205

EPI_ISL_1738207

EPI_ISL_1738208

EPI_ISL_1738209

EPI_ISL_1738210

EPI_ISL_1738211

EPI_ISL_1738213

EPI_ISL_1738214

EPI_ISL_1738215

EPI_ISL_1738217

EPI_ISL_1738218

EPI_ISL_1738222

EPI_ISL_1738224

EPI_ISL_1738225

EPI_ISL_1738227

EPI_ISL_1738228

EPI_ISL_1738229

EPI_ISL_1738231

EPI_ISL_1738232

EPI_ISL_1738233

EPI_ISL_1738234

EPI_ISL_1738236

EPI_ISL_1738237

EPI_ISL_1738238

EPI_ISL_1738239

EPI_ISL_1738240

EPI_ISL_1738243

EPI_ISL_1738244

EPI_ISL_1738245

EPI_ISL_1738247

EPI_ISL_1738248

EPI_ISL_1738249

EPI_ISL_1738251

EPI_ISL_1738252

EPI_ISL_1738253

EPI_ISL_1738255

EPI_ISL_1738256

EPI_ISL_1738257

EPI_ISL_1738258

EPI_ISL_1738262

EPI_ISL_1738264

EPI_ISL_1738265

EPI_ISL_1738266

EPI_ISL_1738267

EPI_ISL_1738268

EPI_ISL_1738269

EPI_ISL_1738270

EPI_ISL_1738271

EPI_ISL_1738273

EPI_ISL_1738275

EPI_ISL_1738276

EPI_ISL_1738278

EPI_ISL_1738279

EPI_ISL_1738284

EPI_ISL_1738285

EPI_ISL_1738286

EPI_ISL_1738287

EPI_ISL_1738288

EPI_ISL_1738290

EPI_ISL_1738291

EPI_ISL_1738293

EPI_ISL_1738294

EPI_ISL_1738295

EPI_ISL_1738297

EPI_ISL_1738298

EPI_ISL_1738299

EPI_ISL_1738301

EPI_ISL_1738303

EPI_ISL_1738304

EPI_ISL_1738305

EPI_ISL_1738306

EPI_ISL_1738307

EPI_ISL_1738309

EPI_ISL_1738310

EPI_ISL_1738311

EPI_ISL_1738314

EPI_ISL_1738315

EPI_ISL_1738316

EPI_ISL_1738317

EPI_ISL_1738318

EPI_ISL_1738321

EPI_ISL_1738325

EPI_ISL_1738327

EPI_ISL_1738330

EPI_ISL_1738332

EPI_ISL_1738334

EPI_ISL_1738336

EPI_ISL_1738337

EPI_ISL_1738338

EPI_ISL_1738340

EPI_ISL_1738342

EPI_ISL_1738347

EPI_ISL_1738348

EPI_ISL_1738353

EPI_ISL_1738354

EPI_ISL_1738355

EPI_ISL_1738356

EPI_ISL_1738360

EPI_ISL_1738361

EPI_ISL_1738364

EPI_ISL_1738365

EPI_ISL_1738366

EPI_ISL_1738367

EPI_ISL_1738368

EPI_ISL_1738373

EPI_ISL_1738374

EPI_ISL_1738375

EPI_ISL_1738376

EPI_ISL_1738378

EPI_ISL_1738379

EPI_ISL_1738381

EPI_ISL_1738382

EPI_ISL_1738383

EPI_ISL_1738384

EPI_ISL_1738386

EPI_ISL_1738387

EPI_ISL_1738388

EPI_ISL_1738391

EPI_ISL_1738393

EPI_ISL_1738396

EPI_ISL_1738399

EPI_ISL_1738400

EPI_ISL_1738403

EPI_ISL_1738404

EPI_ISL_1738406

EPI_ISL_1738407

EPI_ISL_1738409

EPI_ISL_1738410

EPI_ISL_1738413

EPI_ISL_1738417

EPI_ISL_1738420

EPI_ISL_1738421

EPI_ISL_1738423

EPI_ISL_1738425

EPI_ISL_1738426

EPI_ISL_1738429

EPI_ISL_1738431

EPI_ISL_1738433

EPI_ISL_1738435

EPI_ISL_1738437

EPI_ISL_1738438

EPI_ISL_1738439

EPI_ISL_1738440

EPI_ISL_1738442

EPI_ISL_1738446

EPI_ISL_1738447

EPI_ISL_1738448

EPI_ISL_1738450

EPI_ISL_1738452

EPI_ISL_1738454

EPI_ISL_1738455

EPI_ISL_1738458

EPI_ISL_1738460

EPI_ISL_1738461

EPI_ISL_1738462

EPI_ISL_1738463

EPI_ISL_1738464

EPI_ISL_1738469

EPI_ISL_1738470

EPI_ISL_1738471

EPI_ISL_1738473

EPI_ISL_1738474

EPI_ISL_1738477

EPI_ISL_1738478

EPI_ISL_1738480

EPI_ISL_1738481

EPI_ISL_1738482

EPI_ISL_1738485

EPI_ISL_1738486

EPI_ISL_1738488

EPI_ISL_1738490

EPI_ISL_1738492

EPI_ISL_1738493

EPI_ISL_1738494

EPI_ISL_1738496

EPI_ISL_1738499

EPI_ISL_1738501

EPI_ISL_1738504

EPI_ISL_1738505

EPI_ISL_1738508

EPI_ISL_1738509

EPI_ISL_1738512

EPI_ISL_1738513

EPI_ISL_1738516

EPI_ISL_1738518

EPI_ISL_1738519

EPI_ISL_1738520

EPI_ISL_1738524

EPI_ISL_1738525

EPI_ISL_1738528

EPI_ISL_1738535

EPI_ISL_1738537

EPI_ISL_1738538

EPI_ISL_1738540

EPI_ISL_1738541

EPI_ISL_1738542

EPI_ISL_1738544

EPI_ISL_1738545

EPI_ISL_1738548

EPI_ISL_1738549

EPI_ISL_1738550

EPI_ISL_1738556

EPI_ISL_1738557

EPI_ISL_1738558

EPI_ISL_1738560

EPI_ISL_1738563

EPI_ISL_1738566

EPI_ISL_1738568

EPI_ISL_1738569

EPI_ISL_1738571

EPI_ISL_1738572

EPI_ISL_1738573

EPI_ISL_1738574

EPI_ISL_1738575

EPI_ISL_1738579

EPI_ISL_1738580

EPI_ISL_1738584

EPI_ISL_1738586

EPI_ISL_1738587

EPI_ISL_1738588

EPI_ISL_1738593

EPI_ISL_1738594

EPI_ISL_1738596

EPI_ISL_1738597

EPI_ISL_1738601

EPI_ISL_1738602

EPI_ISL_1738603

EPI_ISL_1738604

EPI_ISL_1738605

EPI_ISL_1738608

EPI_ISL_1738609

EPI_ISL_1738610

EPI_ISL_1738611

EPI_ISL_1738613

EPI_ISL_1738616

EPI_ISL_1738620

EPI_ISL_1738624

EPI_ISL_1738625

EPI_ISL_1738626

EPI_ISL_1738628

EPI_ISL_1738631

EPI_ISL_1738632

EPI_ISL_1738636

EPI_ISL_1738637

EPI_ISL_1738640

EPI_ISL_1738642

EPI_ISL_1738643

EPI_ISL_1738644

EPI_ISL_1738646

EPI_ISL_1738649

EPI_ISL_1738650

EPI_ISL_1738653

EPI_ISL_1738655

EPI_ISL_1738657

EPI_ISL_1738658

EPI_ISL_1738659

EPI_ISL_1738660

EPI_ISL_1738661

EPI_ISL_1738666

EPI_ISL_1738667

EPI_ISL_1738668

EPI_ISL_1738670

EPI_ISL_1738671

EPI_ISL_1738673

EPI_ISL_1738675

EPI_ISL_1738676

EPI_ISL_1738677

EPI_ISL_1738679

EPI_ISL_1738681

EPI_ISL_1738682

EPI_ISL_1738683

EPI_ISL_1738684

EPI_ISL_1738685

EPI_ISL_1738686

EPI_ISL_1738687

EPI_ISL_1738688

EPI_ISL_1738692

EPI_ISL_1738693

EPI_ISL_1738694

EPI_ISL_1738695

EPI_ISL_1738697

EPI_ISL_1738698

EPI_ISL_1738701

EPI_ISL_1738703

EPI_ISL_1738704

EPI_ISL_1738705

EPI_ISL_1738706

EPI_ISL_1738707

EPI_ISL_1738708

EPI_ISL_1738709

EPI_ISL_1738710

EPI_ISL_1738711

EPI_ISL_1738712

EPI_ISL_1738713

EPI_ISL_1738714

EPI_ISL_1738715

EPI_ISL_1738717

EPI_ISL_1738719

EPI_ISL_1738720

EPI_ISL_1738722

EPI_ISL_1738724

EPI_ISL_1738725

EPI_ISL_1738726

EPI_ISL_1738727

EPI_ISL_1738729

EPI_ISL_1738730

EPI_ISL_1738732

EPI_ISL_1738733

EPI_ISL_1738734

EPI_ISL_1738738

EPI_ISL_1738740

EPI_ISL_1738743

EPI_ISL_1738744

EPI_ISL_1738745

EPI_ISL_1738746

EPI_ISL_1738751

EPI_ISL_1738755

EPI_ISL_1738756

EPI_ISL_1738757

EPI_ISL_1738764

EPI_ISL_1738766

EPI_ISL_1738767

EPI_ISL_1738768

EPI_ISL_1738769

EPI_ISL_1738770

EPI_ISL_1738776

EPI_ISL_1738777

EPI_ISL_1738779

EPI_ISL_1738780

EPI_ISL_1738791

EPI_ISL_1738796

EPI_ISL_1738797

EPI_ISL_1738798

EPI_ISL_1738799

EPI_ISL_1738800

EPI_ISL_1738801

EPI_ISL_1738802

EPI_ISL_1738817

EPI_ISL_1738819

EPI_ISL_1738821

EPI_ISL_1738824

EPI_ISL_1738826

EPI_ISL_1738836

EPI_ISL_1738839

EPI_ISL_1738840

EPI_ISL_1738843

EPI_ISL_1738846

EPI_ISL_1738847

EPI_ISL_1738855

EPI_ISL_1738868

EPI_ISL_1738869

EPI_ISL_1738879

EPI_ISL_6211193

EPI_ISL_1738890

EPI_ISL_1738892

EPI_ISL_1738893

EPI_ISL_1738895

EPI_ISL_1738899

EPI_ISL_1738900

EPI_ISL_1738901

EPI_ISL_1738907

EPI_ISL_1738912

EPI_ISL_1738917

EPI_ISL_1738920

EPI_ISL_1738921

EPI_ISL_1738928

EPI_ISL_1738929

EPI_ISL_1738932

EPI_ISL_1738933

EPI_ISL_1738935

EPI_ISL_1738937

EPI_ISL_1738940

EPI_ISL_1738950

EPI_ISL_1738951

EPI_ISL_1738953

EPI_ISL_1738954

EPI_ISL_1738955

EPI_ISL_1738959

EPI_ISL_1738963

EPI_ISL_1738968

EPI_ISL_1738972

EPI_ISL_1738985

EPI_ISL_1738995

EPI_ISL_1738996

EPI_ISL_1738997

EPI_ISL_1738999

EPI_ISL_1739000

EPI_ISL_1739001

EPI_ISL_1739002

EPI_ISL_1739003

EPI_ISL_1739004

EPI_ISL_1739005

EPI_ISL_1739006

EPI_ISL_1739007

EPI_ISL_1739008

EPI_ISL_1739009

EPI_ISL_1739010

EPI_ISL_1739014

EPI_ISL_1739015

EPI_ISL_1739016

EPI_ISL_1739018

EPI_ISL_1739020

EPI_ISL_1739021

EPI_ISL_1739022

EPI_ISL_1739023

EPI_ISL_1739024

EPI_ISL_1739025

EPI_ISL_1739026

EPI_ISL_1739027

EPI_ISL_1739028

EPI_ISL_1739029

EPI_ISL_1739030

EPI_ISL_1739031

EPI_ISL_1739032

EPI_ISL_1739035

EPI_ISL_1739036

EPI_ISL_1739037

EPI_ISL_1739038

EPI_ISL_1739039

EPI_ISL_1739042

EPI_ISL_1739046

EPI_ISL_1739047

EPI_ISL_1739050

EPI_ISL_1739051

EPI_ISL_1739053

EPI_ISL_1739054

EPI_ISL_1739057

EPI_ISL_1739059

EPI_ISL_1739060

EPI_ISL_1739062

EPI_ISL_1739063

EPI_ISL_1739064

EPI_ISL_1739071

EPI_ISL_1739072

EPI_ISL_1739073

EPI_ISL_1739078

EPI_ISL_1739079

EPI_ISL_1739080

EPI_ISL_1739081

EPI_ISL_1739082

EPI_ISL_1739083

EPI_ISL_1739085

EPI_ISL_1739086

EPI_ISL_1739087

EPI_ISL_1739089

EPI_ISL_1739090

EPI_ISL_1739091

EPI_ISL_1739092

EPI_ISL_1739093

EPI_ISL_1739094

EPI_ISL_1739095

EPI_ISL_1739096

EPI_ISL_1739099

EPI_ISL_1739102

EPI_ISL_1739108

EPI_ISL_1739111

EPI_ISL_1739115

EPI_ISL_1739120

EPI_ISL_1739121

EPI_ISL_1739124

EPI_ISL_1739126

EPI_ISL_1739127

EPI_ISL_1739129

EPI_ISL_1739130

EPI_ISL_1739133

EPI_ISL_1739134

EPI_ISL_1739139

EPI_ISL_1739140

EPI_ISL_1739141

EPI_ISL_1739143

EPI_ISL_1739144

EPI_ISL_1739145

EPI_ISL_1739147

EPI_ISL_1739148

EPI_ISL_1739149

EPI_ISL_1739151

EPI_ISL_1739152

EPI_ISL_1739157

EPI_ISL_1739158

EPI_ISL_1739159

EPI_ISL_1739163

EPI_ISL_1739164

EPI_ISL_1739165

EPI_ISL_1739166

EPI_ISL_1739168

EPI_ISL_1739171

EPI_ISL_1739177

EPI_ISL_1739178

EPI_ISL_1739181

EPI_ISL_1739183

EPI_ISL_1739195

EPI_ISL_1739196

EPI_ISL_1739197

EPI_ISL_1739200

EPI_ISL_1739203

EPI_ISL_1739204

EPI_ISL_1739207

EPI_ISL_1739212

EPI_ISL_1739213

EPI_ISL_1739215

EPI_ISL_1739217

EPI_ISL_1739219

EPI_ISL_1739220

EPI_ISL_1739222

EPI_ISL_1739227

EPI_ISL_1739231

EPI_ISL_1739236

EPI_ISL_1739237

EPI_ISL_1739238

EPI_ISL_1739240

EPI_ISL_1739241

EPI_ISL_1739246

EPI_ISL_1739247

EPI_ISL_1739248

EPI_ISL_1739249

EPI_ISL_1739252

EPI_ISL_1739253

EPI_ISL_1739259

EPI_ISL_1739261

EPI_ISL_1739265

EPI_ISL_1739267

EPI_ISL_1739271

EPI_ISL_1739275

EPI_ISL_1739280

EPI_ISL_1739281

EPI_ISL_1739282

EPI_ISL_1739283

EPI_ISL_1739285

EPI_ISL_1739289

EPI_ISL_1739291

EPI_ISL_1739307

EPI_ISL_1739308

EPI_ISL_1739309

EPI_ISL_1739311

EPI_ISL_1739325

EPI_ISL_1739326

EPI_ISL_1739328

EPI_ISL_1739329

EPI_ISL_1739361

EPI_ISL_1739364

EPI_ISL_1739365

EPI_ISL_1739366

EPI_ISL_1739367

EPI_ISL_1739368

EPI_ISL_1739369

EPI_ISL_1739370

EPI_ISL_1739371

EPI_ISL_1739372

EPI_ISL_1739373

EPI_ISL_1739374

EPI_ISL_1739375

EPI_ISL_1739376

EPI_ISL_1739377

EPI_ISL_1739378

EPI_ISL_1739379

EPI_ISL_1739382

EPI_ISL_1739383

EPI_ISL_1739384

EPI_ISL_1739386

EPI_ISL_1739389

EPI_ISL_1739390

EPI_ISL_1739391

EPI_ISL_1739392

EPI_ISL_1739393

EPI_ISL_1739394

EPI_ISL_1739395

EPI_ISL_1739396

EPI_ISL_1739397

EPI_ISL_1739398

EPI_ISL_1739399

EPI_ISL_1739400

EPI_ISL_1739401

EPI_ISL_1739402

EPI_ISL_1739403

EPI_ISL_1739404

EPI_ISL_1739406

EPI_ISL_1739407

EPI_ISL_1739408

EPI_ISL_1739409

EPI_ISL_1739411

EPI_ISL_1739412

EPI_ISL_1739413

EPI_ISL_1739414

EPI_ISL_1739415

EPI_ISL_1739416

EPI_ISL_1739417

EPI_ISL_1739418

EPI_ISL_1739419

EPI_ISL_1739420

EPI_ISL_1739421

EPI_ISL_1739422

EPI_ISL_1739423

EPI_ISL_1739424

EPI_ISL_1739425

EPI_ISL_1739428

EPI_ISL_1739430

EPI_ISL_1739437

EPI_ISL_1739443

EPI_ISL_1739444

EPI_ISL_1739445

EPI_ISL_1739449

EPI_ISL_1739452

EPI_ISL_1739456

EPI_ISL_1739457

EPI_ISL_1739458

EPI_ISL_1739462

EPI_ISL_1739463

EPI_ISL_1739468

EPI_ISL_1739472

EPI_ISL_1739473

EPI_ISL_1739474

EPI_ISL_1739479

EPI_ISL_1739480

EPI_ISL_1739482

EPI_ISL_1739484

EPI_ISL_1739486

EPI_ISL_1739487

EPI_ISL_1739488

EPI_ISL_1739490

EPI_ISL_1739491

EPI_ISL_1739493

EPI_ISL_1739494

EPI_ISL_1739496

EPI_ISL_1739497

EPI_ISL_1739498

EPI_ISL_1739505

EPI_ISL_1739506

EPI_ISL_1739507

EPI_ISL_1739508

EPI_ISL_1739509

EPI_ISL_1739510

EPI_ISL_1739514

EPI_ISL_1739515

EPI_ISL_1739516

EPI_ISL_1739519

EPI_ISL_1739523

EPI_ISL_1739524

EPI_ISL_1739525

EPI_ISL_1739526

EPI_ISL_1739529

EPI_ISL_1739531

EPI_ISL_1739532

EPI_ISL_1739533

EPI_ISL_1739534

EPI_ISL_1739535

EPI_ISL_1739536

EPI_ISL_1739538

EPI_ISL_1739539

EPI_ISL_1739540

EPI_ISL_1739541

EPI_ISL_1739542

EPI_ISL_1739543

EPI_ISL_1740492

EPI_ISL_1740493

EPI_ISL_1740532

EPI_ISL_1740534

EPI_ISL_1740536

EPI_ISL_1740538

EPI_ISL_1740539

EPI_ISL_1740540

EPI_ISL_1740542

EPI_ISL_1740543

EPI_ISL_1740548

EPI_ISL_1740549

EPI_ISL_1740550

EPI_ISL_1740552

EPI_ISL_1740554

EPI_ISL_1740555

EPI_ISL_1740556

EPI_ISL_1740559

EPI_ISL_1740560

EPI_ISL_1740562

EPI_ISL_1740563

EPI_ISL_1740564

EPI_ISL_1740565

EPI_ISL_1740566

EPI_ISL_1740568

EPI_ISL_1740569

EPI_ISL_1740570

EPI_ISL_1740572

EPI_ISL_1740573

EPI_ISL_1740574

EPI_ISL_1740576

EPI_ISL_1740577

EPI_ISL_1740579

EPI_ISL_1740581

EPI_ISL_1740582

EPI_ISL_1740583

EPI_ISL_1740584

EPI_ISL_1740586

EPI_ISL_1740587

EPI_ISL_1740588

EPI_ISL_1740589

EPI_ISL_1740590

EPI_ISL_1740591

EPI_ISL_1740592

EPI_ISL_1740593

EPI_ISL_1740594

EPI_ISL_1740595

EPI_ISL_1740597

EPI_ISL_1740598

EPI_ISL_1740599

EPI_ISL_1740601

EPI_ISL_1740602

EPI_ISL_1740603

EPI_ISL_1740604

EPI_ISL_1740605

EPI_ISL_1740606

EPI_ISL_1740608

EPI_ISL_1740609

EPI_ISL_1740612

EPI_ISL_1740613

EPI_ISL_1740614

EPI_ISL_1740615

EPI_ISL_1740618

EPI_ISL_1740620

EPI_ISL_1740621

EPI_ISL_1740623

EPI_ISL_1740624

EPI_ISL_1740625

EPI_ISL_1740627

EPI_ISL_1740628

EPI_ISL_1740629

EPI_ISL_1740630

EPI_ISL_1740631

EPI_ISL_1740632

EPI_ISL_1740634

EPI_ISL_1740637

EPI_ISL_1740638

EPI_ISL_1740639

EPI_ISL_1740640

EPI_ISL_1740641

EPI_ISL_1740643

EPI_ISL_1740644

EPI_ISL_1740645

EPI_ISL_1740646

EPI_ISL_1740647

EPI_ISL_1740648

EPI_ISL_1740649

EPI_ISL_1740650

EPI_ISL_1740651

EPI_ISL_1740652

EPI_ISL_1740653

EPI_ISL_1740658

EPI_ISL_1740665

EPI_ISL_1740666

EPI_ISL_1740670

EPI_ISL_1740678

EPI_ISL_1740681

EPI_ISL_1740683

EPI_ISL_1740692

EPI_ISL_1740700

EPI_ISL_1740701

EPI_ISL_1740702

EPI_ISL_1740703

EPI_ISL_1740704

EPI_ISL_1740705

EPI_ISL_1740706

EPI_ISL_1740708

EPI_ISL_1740709

EPI_ISL_1740711

EPI_ISL_1740713

EPI_ISL_1740714

EPI_ISL_1740715

EPI_ISL_1740717

EPI_ISL_1740719

EPI_ISL_1740720

EPI_ISL_1740722

EPI_ISL_1740727

EPI_ISL_1740730

EPI_ISL_1740731

EPI_ISL_1740733

EPI_ISL_1740736

EPI_ISL_1740737

EPI_ISL_1740740

EPI_ISL_1740742

EPI_ISL_1740743

EPI_ISL_1740744

EPI_ISL_1740756

EPI_ISL_1740757

EPI_ISL_1740765

EPI_ISL_1740768

EPI_ISL_1740770

EPI_ISL_1740775

EPI_ISL_1740776

EPI_ISL_1740777

EPI_ISL_1740780

EPI_ISL_1740782

EPI_ISL_1740787

EPI_ISL_1740788

EPI_ISL_1740789

EPI_ISL_1740791

EPI_ISL_1740794

EPI_ISL_1740800

EPI_ISL_1740801

EPI_ISL_1740804

EPI_ISL_1740805

EPI_ISL_1740806

EPI_ISL_1740808

EPI_ISL_1740809

EPI_ISL_1740810

EPI_ISL_1740812

EPI_ISL_1740813

EPI_ISL_1740814

EPI_ISL_1740816

EPI_ISL_1740817

EPI_ISL_1740818

EPI_ISL_1740821

EPI_ISL_1740823

EPI_ISL_1740827

EPI_ISL_1740830

EPI_ISL_1740836

EPI_ISL_1740837

EPI_ISL_1740840

EPI_ISL_1740842

EPI_ISL_1740843

EPI_ISL_1740846

EPI_ISL_1740850

EPI_ISL_1740851

EPI_ISL_1740852

EPI_ISL_1740853

EPI_ISL_1740855

EPI_ISL_1740856

EPI_ISL_1740857

EPI_ISL_1740862

EPI_ISL_1740869

EPI_ISL_1740870

EPI_ISL_1740872

EPI_ISL_1740880

EPI_ISL_1740885

EPI_ISL_1740887

EPI_ISL_1740890

EPI_ISL_1740891

EPI_ISL_1740893

EPI_ISL_1740900

EPI_ISL_1740903

EPI_ISL_1740904

EPI_ISL_1740905

EPI_ISL_1740906

EPI_ISL_1740908

EPI_ISL_1740909

EPI_ISL_1740910

EPI_ISL_1740911

EPI_ISL_1740912

EPI_ISL_1740913

EPI_ISL_1740915

EPI_ISL_1740916

EPI_ISL_1740917

EPI_ISL_1740918

EPI_ISL_1740920

EPI_ISL_1740922

EPI_ISL_1740923

EPI_ISL_1740924

EPI_ISL_1740927

EPI_ISL_1740934

EPI_ISL_1740939

EPI_ISL_1740946

EPI_ISL_1740949

EPI_ISL_1740950

EPI_ISL_1740951

EPI_ISL_1740952

EPI_ISL_1740954

EPI_ISL_1740959

EPI_ISL_1740960

EPI_ISL_1740962

EPI_ISL_1740969

EPI_ISL_1740971

EPI_ISL_1740972

EPI_ISL_1740973

EPI_ISL_1740974

EPI_ISL_1740975

EPI_ISL_1740976

EPI_ISL_1740977

EPI_ISL_1740979

EPI_ISL_1740981

EPI_ISL_1740982

EPI_ISL_1740984

EPI_ISL_1740986

EPI_ISL_1740992

EPI_ISL_1740995

EPI_ISL_1740997

EPI_ISL_1741001

EPI_ISL_1741002

EPI_ISL_1741004

EPI_ISL_1741005

EPI_ISL_1741006

EPI_ISL_1741007

EPI_ISL_1741008

EPI_ISL_1741009

EPI_ISL_1741010

EPI_ISL_1741011

EPI_ISL_1741012

EPI_ISL_1741015

EPI_ISL_1741016

EPI_ISL_1741019

EPI_ISL_1741020

EPI_ISL_1741026

EPI_ISL_1741027

EPI_ISL_1741028

EPI_ISL_1741029

EPI_ISL_1741031

EPI_ISL_1741032

EPI_ISL_1741033

EPI_ISL_1741034

EPI_ISL_1741036

EPI_ISL_1741037

EPI_ISL_1741038

EPI_ISL_1741039

EPI_ISL_1741040

EPI_ISL_1741041

EPI_ISL_1741042

EPI_ISL_1741043

EPI_ISL_1741044

EPI_ISL_1741045

EPI_ISL_1741046

EPI_ISL_1741047

EPI_ISL_1741048

EPI_ISL_1741050

EPI_ISL_1741052

EPI_ISL_1741055

EPI_ISL_1741056

EPI_ISL_1741057

EPI_ISL_1741058

EPI_ISL_1741059

EPI_ISL_1741061

EPI_ISL_1741062

EPI_ISL_1741064

EPI_ISL_1741067

EPI_ISL_1741068

EPI_ISL_1741069

EPI_ISL_1741070

EPI_ISL_1741071

EPI_ISL_1741072

EPI_ISL_1741074

EPI_ISL_1741075

EPI_ISL_1741077

EPI_ISL_1741078

EPI_ISL_1741079

EPI_ISL_1741080

EPI_ISL_1741082

EPI_ISL_1741083

EPI_ISL_1741084

EPI_ISL_1741085

EPI_ISL_1741086

EPI_ISL_1741087

EPI_ISL_1741088

EPI_ISL_1741089

EPI_ISL_1741090

EPI_ISL_1741091

EPI_ISL_1741092

EPI_ISL_1741093

EPI_ISL_1741094

EPI_ISL_1741095

EPI_ISL_1741097

EPI_ISL_1741098

EPI_ISL_1741100

EPI_ISL_1741101

EPI_ISL_1741102

EPI_ISL_1741103

EPI_ISL_1741107

EPI_ISL_1741108

EPI_ISL_1741109

EPI_ISL_1741110

EPI_ISL_1741111

EPI_ISL_1741112

EPI_ISL_1741113

EPI_ISL_1741115

EPI_ISL_1741116

EPI_ISL_1741118

EPI_ISL_1741119

EPI_ISL_1741120

EPI_ISL_1741121

EPI_ISL_1741123

EPI_ISL_1741124

EPI_ISL_1741126

EPI_ISL_1741127

EPI_ISL_1741130

EPI_ISL_1741131

EPI_ISL_1741132

EPI_ISL_1741133

EPI_ISL_1741134

EPI_ISL_1741135

EPI_ISL_1741136

EPI_ISL_1741138

EPI_ISL_1741139

EPI_ISL_1741140

EPI_ISL_1741141

EPI_ISL_1741142

EPI_ISL_1741143

EPI_ISL_1741144

EPI_ISL_1741145

EPI_ISL_1741146

EPI_ISL_1741147

EPI_ISL_1741148

EPI_ISL_1741149

EPI_ISL_1741151

EPI_ISL_1741152

EPI_ISL_1741153

EPI_ISL_1741154

EPI_ISL_1741155

EPI_ISL_1741159

EPI_ISL_1741160

EPI_ISL_1741161

EPI_ISL_1741162

EPI_ISL_1741163

EPI_ISL_1741164

EPI_ISL_1741165

EPI_ISL_1741166

EPI_ISL_1741167

EPI_ISL_1741168

EPI_ISL_1741169

EPI_ISL_1741171

EPI_ISL_1741172

EPI_ISL_1741173

EPI_ISL_1741174

EPI_ISL_1741175

EPI_ISL_1741176

EPI_ISL_1741177

EPI_ISL_1741178

EPI_ISL_1741179

EPI_ISL_1741181

EPI_ISL_1741183

EPI_ISL_1741184

EPI_ISL_1741185

EPI_ISL_1741187

EPI_ISL_1741188

EPI_ISL_1741189

EPI_ISL_1741190

EPI_ISL_1741191

EPI_ISL_1741192

EPI_ISL_1741194

EPI_ISL_1741195

EPI_ISL_1741196

EPI_ISL_1741197

EPI_ISL_1741198

EPI_ISL_1741199

EPI_ISL_1741200

EPI_ISL_1741201

EPI_ISL_1741202

EPI_ISL_1741203

EPI_ISL_1741204

EPI_ISL_1741205

EPI_ISL_1741206

EPI_ISL_1741207

EPI_ISL_1741208

EPI_ISL_1741209

EPI_ISL_1741211

EPI_ISL_1741214

EPI_ISL_1741215

EPI_ISL_1741216

EPI_ISL_1741217

EPI_ISL_1741218

EPI_ISL_1741219

EPI_ISL_1741220

EPI_ISL_1741221

EPI_ISL_1741222

EPI_ISL_1741224

EPI_ISL_1741226

EPI_ISL_1741229

EPI_ISL_1741230

EPI_ISL_1741232

EPI_ISL_1741233

EPI_ISL_1741235

EPI_ISL_1741236

EPI_ISL_1741237

EPI_ISL_1741238

EPI_ISL_1741239

EPI_ISL_1741240

EPI_ISL_1741241

EPI_ISL_1741242

EPI_ISL_1741246

EPI_ISL_1741247

EPI_ISL_1741250

EPI_ISL_1741253

EPI_ISL_1741254

EPI_ISL_1741256

EPI_ISL_1741257

EPI_ISL_1741258

EPI_ISL_1741260

EPI_ISL_1741261

EPI_ISL_1741262

EPI_ISL_1741263

EPI_ISL_1741264

EPI_ISL_1741265

EPI_ISL_1741267

EPI_ISL_1741269

EPI_ISL_1741270

EPI_ISL_1741271

EPI_ISL_1741273

EPI_ISL_1741274

EPI_ISL_1741275

EPI_ISL_1741276

EPI_ISL_1741277

EPI_ISL_1741278

EPI_ISL_1741279

EPI_ISL_1741280

EPI_ISL_1741281

EPI_ISL_1741282

EPI_ISL_1741283

EPI_ISL_1741285

EPI_ISL_1741287

EPI_ISL_1741288

EPI_ISL_1741289

EPI_ISL_1741290

EPI_ISL_1741292

EPI_ISL_1741293

EPI_ISL_1741294

EPI_ISL_1741295

EPI_ISL_1741296

EPI_ISL_1741297

EPI_ISL_1741298

EPI_ISL_1741299

EPI_ISL_1741300

EPI_ISL_1741301

EPI_ISL_1741302

EPI_ISL_1741303

EPI_ISL_1741305

EPI_ISL_1741306

EPI_ISL_1741307

EPI_ISL_1741308

EPI_ISL_1741310

EPI_ISL_1741312

EPI_ISL_1741314

EPI_ISL_1741315

EPI_ISL_1741316

EPI_ISL_1741317

EPI_ISL_1741318

EPI_ISL_1741321

EPI_ISL_1741322

EPI_ISL_1741323

EPI_ISL_1741324

EPI_ISL_1741326

EPI_ISL_1741327

EPI_ISL_1741328

EPI_ISL_1741330

EPI_ISL_1741333

EPI_ISL_1741334

EPI_ISL_1741336

EPI_ISL_1741337

EPI_ISL_1741339

EPI_ISL_1741340

EPI_ISL_1741341

EPI_ISL_1741342

EPI_ISL_1741343

EPI_ISL_1741345

EPI_ISL_1741347

EPI_ISL_1741348

EPI_ISL_1741349

EPI_ISL_1741350

EPI_ISL_1741351

EPI_ISL_1741352

EPI_ISL_1741353

EPI_ISL_1741354

EPI_ISL_1741355

EPI_ISL_1741356

EPI_ISL_1741357

EPI_ISL_1741358

EPI_ISL_1741359

EPI_ISL_1741364

EPI_ISL_1741366

EPI_ISL_1741367

EPI_ISL_1741368

EPI_ISL_1741369

EPI_ISL_1741370

EPI_ISL_1741372

EPI_ISL_1741373

EPI_ISL_1741374

EPI_ISL_1741375

EPI_ISL_1741376

EPI_ISL_1741377

EPI_ISL_1741379

EPI_ISL_1741380

EPI_ISL_1741381

EPI_ISL_1741382

EPI_ISL_1741384

EPI_ISL_1741385

EPI_ISL_1741386

EPI_ISL_1741387

EPI_ISL_1741388

EPI_ISL_1741389

EPI_ISL_1741390

EPI_ISL_1741391

EPI_ISL_1741392

EPI_ISL_1741393

EPI_ISL_1741395

EPI_ISL_1741396

EPI_ISL_1741397

EPI_ISL_1741399

EPI_ISL_1741400

EPI_ISL_1741402

EPI_ISL_1741403

EPI_ISL_1741404

EPI_ISL_1741406

EPI_ISL_1741407

EPI_ISL_1741408

EPI_ISL_1741409

EPI_ISL_1741413

EPI_ISL_1741414

EPI_ISL_1741415

EPI_ISL_1741417

EPI_ISL_1741420

EPI_ISL_1741422

EPI_ISL_1741423

EPI_ISL_1741424

EPI_ISL_1741426

EPI_ISL_1741427

EPI_ISL_1741428

EPI_ISL_1741429

EPI_ISL_1741431

EPI_ISL_1741432

EPI_ISL_1741435

EPI_ISL_1741436

EPI_ISL_1741438

EPI_ISL_1741439

EPI_ISL_1741440

EPI_ISL_1741441

EPI_ISL_1741442

EPI_ISL_1741443

EPI_ISL_1741444

EPI_ISL_1741445

EPI_ISL_1741447

EPI_ISL_1741448

EPI_ISL_1741449

EPI_ISL_1741450

EPI_ISL_1741451

EPI_ISL_1741452

EPI_ISL_1741453

EPI_ISL_1741454

EPI_ISL_1741455

EPI_ISL_1741456

EPI_ISL_1741457

EPI_ISL_1741458

EPI_ISL_1741460

EPI_ISL_1741461

EPI_ISL_1741464

EPI_ISL_1741466

EPI_ISL_1741468

EPI_ISL_1741469

EPI_ISL_1741471

EPI_ISL_1741473

EPI_ISL_1741474

EPI_ISL_1741475

EPI_ISL_1741476

EPI_ISL_1741478

EPI_ISL_1741479

EPI_ISL_1741480

EPI_ISL_1741481

EPI_ISL_1741483

EPI_ISL_1741485

EPI_ISL_1741486

EPI_ISL_1741487

EPI_ISL_1741488

EPI_ISL_1741489

EPI_ISL_1741490

EPI_ISL_1741491

EPI_ISL_1741492

EPI_ISL_1741497

EPI_ISL_1741498

EPI_ISL_1741499

EPI_ISL_1741500

EPI_ISL_1741501

EPI_ISL_1741502

EPI_ISL_1741504

EPI_ISL_1741505

EPI_ISL_1741506

EPI_ISL_1741507

EPI_ISL_1741508

EPI_ISL_1741509

EPI_ISL_1741510

EPI_ISL_1741511

EPI_ISL_1741512

EPI_ISL_1741513

EPI_ISL_1741514

EPI_ISL_1741515

EPI_ISL_1741516

EPI_ISL_1741517

EPI_ISL_1741518

EPI_ISL_1741519

EPI_ISL_1741521

EPI_ISL_1741522

EPI_ISL_1741523

EPI_ISL_1741524

EPI_ISL_1741526

EPI_ISL_1741528

EPI_ISL_1741530

EPI_ISL_1741532

EPI_ISL_1741534

EPI_ISL_1741537

EPI_ISL_1741540

EPI_ISL_1741541

EPI_ISL_1741543

EPI_ISL_1741547

EPI_ISL_1741548

EPI_ISL_1741549

EPI_ISL_1741550

EPI_ISL_1741551

EPI_ISL_1741553

EPI_ISL_1741554

EPI_ISL_1741559

EPI_ISL_1741561

EPI_ISL_1741562

EPI_ISL_1741564

EPI_ISL_1741565

EPI_ISL_1741567

EPI_ISL_1741568

EPI_ISL_1741570

EPI_ISL_1741571

EPI_ISL_1741573

EPI_ISL_1741574

EPI_ISL_1741576

EPI_ISL_1741577

EPI_ISL_1741579

EPI_ISL_1741580

EPI_ISL_1741582

EPI_ISL_1741584

EPI_ISL_1741585

EPI_ISL_1741588

EPI_ISL_1741589

EPI_ISL_1741590

EPI_ISL_1741591

EPI_ISL_1741592

EPI_ISL_1741593

EPI_ISL_1741594

EPI_ISL_1741596

EPI_ISL_1741597

EPI_ISL_1741599

EPI_ISL_1741600

EPI_ISL_1741601

EPI_ISL_1741602

EPI_ISL_1741603

EPI_ISL_1741605

EPI_ISL_1741606

EPI_ISL_1741608

EPI_ISL_1741609

EPI_ISL_1741611

EPI_ISL_1741613

EPI_ISL_1741614

EPI_ISL_1741615

EPI_ISL_1741616

EPI_ISL_1741617

EPI_ISL_1741618

EPI_ISL_1741619

EPI_ISL_1741620

EPI_ISL_1741621

EPI_ISL_1741622

EPI_ISL_1741624

EPI_ISL_1741625

EPI_ISL_1741626

EPI_ISL_1741627

EPI_ISL_1741628

EPI_ISL_1741629

EPI_ISL_1741630

EPI_ISL_1741631

EPI_ISL_1741633

EPI_ISL_1741634

EPI_ISL_1741635

EPI_ISL_1741636

EPI_ISL_1741637

EPI_ISL_1741638

EPI_ISL_1741639

EPI_ISL_1741640

EPI_ISL_1741641

EPI_ISL_1741642

EPI_ISL_1741644

EPI_ISL_1741645

EPI_ISL_1741646

EPI_ISL_1741647

EPI_ISL_1741648

EPI_ISL_1741649

EPI_ISL_1741652

EPI_ISL_1741653

EPI_ISL_1741654

EPI_ISL_1741655

EPI_ISL_1741656

EPI_ISL_1741657

EPI_ISL_1741658

EPI_ISL_1741660

EPI_ISL_1741662

EPI_ISL_1741663

EPI_ISL_1741664

EPI_ISL_1741666

EPI_ISL_1741667

EPI_ISL_1741669

EPI_ISL_1741670

EPI_ISL_1741671

EPI_ISL_1741674

EPI_ISL_1741675

EPI_ISL_1741676

EPI_ISL_1741678

EPI_ISL_1741679

EPI_ISL_1741680

EPI_ISL_1741681

EPI_ISL_1741682

EPI_ISL_1741683

EPI_ISL_1741684

EPI_ISL_1741685

EPI_ISL_1741686

EPI_ISL_1741687

EPI_ISL_1741688

EPI_ISL_1741690

EPI_ISL_1741691

EPI_ISL_1741693

EPI_ISL_1741694

EPI_ISL_1741695

EPI_ISL_1741696

EPI_ISL_1741697

EPI_ISL_1741698

EPI_ISL_1741699

EPI_ISL_1741700

EPI_ISL_1741701

EPI_ISL_1741702

EPI_ISL_1741703

EPI_ISL_1741704

EPI_ISL_1741706

EPI_ISL_1741707

EPI_ISL_1741708

EPI_ISL_1741709

EPI_ISL_1741710

EPI_ISL_1741711

EPI_ISL_1741712

EPI_ISL_1741713

EPI_ISL_1741714

EPI_ISL_1741715

EPI_ISL_1741716

EPI_ISL_1741717

EPI_ISL_1741718

EPI_ISL_1741719

EPI_ISL_1741720

EPI_ISL_1741721

EPI_ISL_1741722

EPI_ISL_1741723

EPI_ISL_1741724

EPI_ISL_1741725

EPI_ISL_1741726

EPI_ISL_1741727

EPI_ISL_1741729

EPI_ISL_1741731

EPI_ISL_1741732

EPI_ISL_1741733

EPI_ISL_1741734

EPI_ISL_1741735

EPI_ISL_1741736

EPI_ISL_1741737

EPI_ISL_1741738

EPI_ISL_1741739

EPI_ISL_1741741

EPI_ISL_1741742

EPI_ISL_1741743

EPI_ISL_1741744

EPI_ISL_1741745

EPI_ISL_1741748

EPI_ISL_1741749

EPI_ISL_1741750

EPI_ISL_1741751

EPI_ISL_1741752

EPI_ISL_1741753

EPI_ISL_1741754

EPI_ISL_1741756

EPI_ISL_1741757

EPI_ISL_1741758

EPI_ISL_1741759

EPI_ISL_1741760

EPI_ISL_1741761

EPI_ISL_1741763

EPI_ISL_1741764

EPI_ISL_1741765

EPI_ISL_1741767

EPI_ISL_1741768

EPI_ISL_1741770

EPI_ISL_1741771

EPI_ISL_1741772

EPI_ISL_1741773

EPI_ISL_1741774

EPI_ISL_1741775

EPI_ISL_1741776

EPI_ISL_1741777

EPI_ISL_1741778

EPI_ISL_1741779

EPI_ISL_1741780

EPI_ISL_1741781

EPI_ISL_1741782

EPI_ISL_1741783

EPI_ISL_1741785

EPI_ISL_1741786

EPI_ISL_1741787

EPI_ISL_1741789

EPI_ISL_1741790

EPI_ISL_1741791

EPI_ISL_1741792

EPI_ISL_1741793

EPI_ISL_1741794

EPI_ISL_1741795

EPI_ISL_1741797

EPI_ISL_1741798

EPI_ISL_1741799

EPI_ISL_1741800

EPI_ISL_1741802

EPI_ISL_1741803

EPI_ISL_1741804

EPI_ISL_1741805

EPI_ISL_1741806

EPI_ISL_1741807

EPI_ISL_1741808

EPI_ISL_1741809

EPI_ISL_1741810

EPI_ISL_1741811

EPI_ISL_1741812

EPI_ISL_1741813

EPI_ISL_1741815

EPI_ISL_1741816

EPI_ISL_1741817

EPI_ISL_1741818

EPI_ISL_1741819

EPI_ISL_1741820

EPI_ISL_1741821

EPI_ISL_1741822

EPI_ISL_1741823

EPI_ISL_1741825

EPI_ISL_1741826

EPI_ISL_1741827

EPI_ISL_1741828

EPI_ISL_1741829

EPI_ISL_1741830

EPI_ISL_1741831

EPI_ISL_1741832

EPI_ISL_1741833

EPI_ISL_1741834

EPI_ISL_1741835

EPI_ISL_1741837

EPI_ISL_1741839

EPI_ISL_1741840

EPI_ISL_1741841

EPI_ISL_1741842

EPI_ISL_1741843

EPI_ISL_1741844

EPI_ISL_1741845

EPI_ISL_1741846

EPI_ISL_1741847

EPI_ISL_1741848

EPI_ISL_1741849

EPI_ISL_1741850

EPI_ISL_1741851

EPI_ISL_1741852

EPI_ISL_1741853

EPI_ISL_1741854

EPI_ISL_1741855

EPI_ISL_1741856

EPI_ISL_1741857

EPI_ISL_1741858

EPI_ISL_1741860

EPI_ISL_1741861

EPI_ISL_1741862

EPI_ISL_1741863

EPI_ISL_1741864

EPI_ISL_1741865

EPI_ISL_1741866

EPI_ISL_1741867

EPI_ISL_1741868

EPI_ISL_1741869

EPI_ISL_1741870

EPI_ISL_1741871

EPI_ISL_1741872

EPI_ISL_1741873

EPI_ISL_1741874

EPI_ISL_1741875

EPI_ISL_1741876

EPI_ISL_1741877

EPI_ISL_1741878

EPI_ISL_1741879

EPI_ISL_1741880

EPI_ISL_1741881

EPI_ISL_1741882

EPI_ISL_1741883

EPI_ISL_1741884

EPI_ISL_1741885

EPI_ISL_1741886

EPI_ISL_1741887

EPI_ISL_1741889

EPI_ISL_1741890

EPI_ISL_1741891

EPI_ISL_1741892

EPI_ISL_1741893

EPI_ISL_1741896

EPI_ISL_1741897

EPI_ISL_1741898

EPI_ISL_1741899

EPI_ISL_1741900

EPI_ISL_1741901

EPI_ISL_1741902

EPI_ISL_1741903

EPI_ISL_1741904

EPI_ISL_1741905

EPI_ISL_1741906

EPI_ISL_1741907

EPI_ISL_1741908

EPI_ISL_1741910

EPI_ISL_1741911

EPI_ISL_1741912

EPI_ISL_1741913

EPI_ISL_1741914

EPI_ISL_1741915

EPI_ISL_1741916

EPI_ISL_1741917

EPI_ISL_1741918

EPI_ISL_1741919

EPI_ISL_1741920

EPI_ISL_1741921

EPI_ISL_1741922

EPI_ISL_1741923

EPI_ISL_1741924

EPI_ISL_1741925

EPI_ISL_1741926

EPI_ISL_1741927

EPI_ISL_1741928

EPI_ISL_1741929

EPI_ISL_1741930

EPI_ISL_1741931

EPI_ISL_1741933

EPI_ISL_1741934

EPI_ISL_1741935

EPI_ISL_1741936

EPI_ISL_1741937

EPI_ISL_1741938

EPI_ISL_1741939

EPI_ISL_1741940

EPI_ISL_1741941

EPI_ISL_1741942

EPI_ISL_1741943

EPI_ISL_1741944

EPI_ISL_1741945

EPI_ISL_1741946

EPI_ISL_1741947

EPI_ISL_1741948

EPI_ISL_1741949

EPI_ISL_1741950

EPI_ISL_1741951

EPI_ISL_1741952

EPI_ISL_1741953

EPI_ISL_1741954

EPI_ISL_1741955

EPI_ISL_1741956

EPI_ISL_1741957

EPI_ISL_1741958

EPI_ISL_1741959

EPI_ISL_1741960

EPI_ISL_1741961

EPI_ISL_1741962

EPI_ISL_1741963

EPI_ISL_1741964

EPI_ISL_1741965

EPI_ISL_1741966

EPI_ISL_1741967

EPI_ISL_1741968

EPI_ISL_1741969

EPI_ISL_1741970

EPI_ISL_1741971

EPI_ISL_1741972

EPI_ISL_1741973

EPI_ISL_1741974

EPI_ISL_1741975

EPI_ISL_1741976

EPI_ISL_1741977

EPI_ISL_1741979

EPI_ISL_1741980

EPI_ISL_1741981

EPI_ISL_1741982

EPI_ISL_1741983

EPI_ISL_1741984

EPI_ISL_1741985

EPI_ISL_1741986

EPI_ISL_1741987

EPI_ISL_1741988

EPI_ISL_1741989

EPI_ISL_1741990

EPI_ISL_1741991

EPI_ISL_1741992

EPI_ISL_1741993

EPI_ISL_1741994

EPI_ISL_1741995

EPI_ISL_1741996

EPI_ISL_1741997

EPI_ISL_1741998

EPI_ISL_1741999

EPI_ISL_1742000

EPI_ISL_1742001

EPI_ISL_1742002

EPI_ISL_1742003

EPI_ISL_1742004

EPI_ISL_1742005

EPI_ISL_1742006

EPI_ISL_1742007

EPI_ISL_1742008

EPI_ISL_1742009

EPI_ISL_1742010

EPI_ISL_1742011

EPI_ISL_1742012

EPI_ISL_1742013

EPI_ISL_1742014

EPI_ISL_1742015

EPI_ISL_1742016

EPI_ISL_1742017

EPI_ISL_1742018

EPI_ISL_1742019

EPI_ISL_1742020

EPI_ISL_1742021

EPI_ISL_1742022

EPI_ISL_1742023

EPI_ISL_1742025

EPI_ISL_1742026

EPI_ISL_1742028

EPI_ISL_1742029

EPI_ISL_1742030

EPI_ISL_1742031

EPI_ISL_1742032

EPI_ISL_1742033

EPI_ISL_1742034

EPI_ISL_1742035

EPI_ISL_1742036

EPI_ISL_1742037

EPI_ISL_1742038

EPI_ISL_1742039

EPI_ISL_1742041

EPI_ISL_1742042

EPI_ISL_1742043

EPI_ISL_1742044

EPI_ISL_1742045

EPI_ISL_1742046

EPI_ISL_1742047

EPI_ISL_1742048

EPI_ISL_1742049

EPI_ISL_1742050

EPI_ISL_1742051

EPI_ISL_1742052

EPI_ISL_1742053

EPI_ISL_1742054

EPI_ISL_1742055

EPI_ISL_1742057

EPI_ISL_1742058

EPI_ISL_1742059

EPI_ISL_1742060

EPI_ISL_1742061

EPI_ISL_1742062

EPI_ISL_1742064

EPI_ISL_1742065

EPI_ISL_1742066

EPI_ISL_1742067

EPI_ISL_1742068

EPI_ISL_1742071

EPI_ISL_1742072

EPI_ISL_1742073

EPI_ISL_1742074

EPI_ISL_1742077

EPI_ISL_1742079

EPI_ISL_1742080

EPI_ISL_1742082

EPI_ISL_1742083

EPI_ISL_1742087

EPI_ISL_1742088

EPI_ISL_1742089

EPI_ISL_1742090

EPI_ISL_1742091

EPI_ISL_1742092

EPI_ISL_1742093

EPI_ISL_1742094

EPI_ISL_1742095

EPI_ISL_1742097

EPI_ISL_1742098

EPI_ISL_1742099

EPI_ISL_1742100

EPI_ISL_1742101

EPI_ISL_1742102

EPI_ISL_1742105

EPI_ISL_1742106

EPI_ISL_1742107

EPI_ISL_1742108

EPI_ISL_1742111

EPI_ISL_1742112

EPI_ISL_1742113

EPI_ISL_1742114

EPI_ISL_1742115

EPI_ISL_1742117

EPI_ISL_1742118

EPI_ISL_1742119

EPI_ISL_1742120

EPI_ISL_1742121

EPI_ISL_1742122

EPI_ISL_1742124

EPI_ISL_1742127

EPI_ISL_1742128

EPI_ISL_1742129

EPI_ISL_1742130

EPI_ISL_1742131

EPI_ISL_1742132

EPI_ISL_1742133

EPI_ISL_1742134

EPI_ISL_1742135

EPI_ISL_1742136

EPI_ISL_1742137

EPI_ISL_1742139

EPI_ISL_1742140

EPI_ISL_1742141

EPI_ISL_1742142

EPI_ISL_1742143

EPI_ISL_1742145

EPI_ISL_1742148

EPI_ISL_1742150

EPI_ISL_1742151

EPI_ISL_1742153

EPI_ISL_1742156

EPI_ISL_1742157

EPI_ISL_1742159

EPI_ISL_1742160

EPI_ISL_1742161

EPI_ISL_1742163

EPI_ISL_1742164

EPI_ISL_1742166

EPI_ISL_1742167

EPI_ISL_1742169

EPI_ISL_1742170

EPI_ISL_1742171

EPI_ISL_1742172

EPI_ISL_1742173

EPI_ISL_1742174

EPI_ISL_1742175

EPI_ISL_1742176

EPI_ISL_1742177

EPI_ISL_1742178

EPI_ISL_1742179

EPI_ISL_1742180

EPI_ISL_1742181

EPI_ISL_1742183

EPI_ISL_1742184

EPI_ISL_1742185

EPI_ISL_1742186

EPI_ISL_1742187

EPI_ISL_1742188

EPI_ISL_1742190

EPI_ISL_1742193

EPI_ISL_1742194

EPI_ISL_1742197

EPI_ISL_1742198

EPI_ISL_1742199

EPI_ISL_1742200

EPI_ISL_1742201

EPI_ISL_1742202

EPI_ISL_1742204

EPI_ISL_1742206

EPI_ISL_1742207

EPI_ISL_1742209

EPI_ISL_1742210

EPI_ISL_1742211

EPI_ISL_1742212

EPI_ISL_1742213

EPI_ISL_1742214

EPI_ISL_1742216

EPI_ISL_1742217

EPI_ISL_1742218

EPI_ISL_1742219

EPI_ISL_1742220

EPI_ISL_1742221

EPI_ISL_1742224

EPI_ISL_1742225

EPI_ISL_1742226

EPI_ISL_1742228

EPI_ISL_1742229

EPI_ISL_1742230

EPI_ISL_1742231

EPI_ISL_1742233

EPI_ISL_1742234

EPI_ISL_1742235

EPI_ISL_1742236

EPI_ISL_1742237

EPI_ISL_1742238

EPI_ISL_1742239

EPI_ISL_1742240

EPI_ISL_1742241

EPI_ISL_1742242

EPI_ISL_1742244

EPI_ISL_1742246

EPI_ISL_1742247

EPI_ISL_1742249

EPI_ISL_1742251

EPI_ISL_1742252

EPI_ISL_1742254

EPI_ISL_1742264

EPI_ISL_1742268

EPI_ISL_1742270

EPI_ISL_1742276

EPI_ISL_1742282

EPI_ISL_1742285

EPI_ISL_1742288

EPI_ISL_1742293

EPI_ISL_1742294

EPI_ISL_1742300

EPI_ISL_1742301

EPI_ISL_1742302

EPI_ISL_1742303

EPI_ISL_1742305

EPI_ISL_1742306

EPI_ISL_1742314

EPI_ISL_1742315

EPI_ISL_1742317

EPI_ISL_1742318

EPI_ISL_1742319

EPI_ISL_1742323

EPI_ISL_1742324

EPI_ISL_1742326

EPI_ISL_1742327

EPI_ISL_1742328

EPI_ISL_1742330

EPI_ISL_1742332

EPI_ISL_1742333

EPI_ISL_1742341

EPI_ISL_1742353

EPI_ISL_1742356

EPI_ISL_1742358

EPI_ISL_1742359

EPI_ISL_1742360

EPI_ISL_1742366

EPI_ISL_1742368

EPI_ISL_1742369

EPI_ISL_1742371

EPI_ISL_1742378

EPI_ISL_1742382

EPI_ISL_1742383

EPI_ISL_1742384

EPI_ISL_1742385

EPI_ISL_1742390

EPI_ISL_1742398

EPI_ISL_1742400

EPI_ISL_1742404

EPI_ISL_1742405

EPI_ISL_1742407

EPI_ISL_1742409

EPI_ISL_1742410

EPI_ISL_1742421

EPI_ISL_1742423

EPI_ISL_1742424

EPI_ISL_1742426

EPI_ISL_1742427

EPI_ISL_1742428

EPI_ISL_1742429

EPI_ISL_1742430

EPI_ISL_1742431

EPI_ISL_1742461

EPI_ISL_1742462

EPI_ISL_1742479

EPI_ISL_1742488

EPI_ISL_1742497

EPI_ISL_1742501

EPI_ISL_1742504

EPI_ISL_1742507

EPI_ISL_1742508

EPI_ISL_1742516

EPI_ISL_1742712

EPI_ISL_1742713

EPI_ISL_1742714

EPI_ISL_1742716

EPI_ISL_1742718

EPI_ISL_1742719

EPI_ISL_1742720

EPI_ISL_1742721

EPI_ISL_1742724

EPI_ISL_1742725

EPI_ISL_1742728

EPI_ISL_1742729

EPI_ISL_1742732

EPI_ISL_1742738

EPI_ISL_1742739

EPI_ISL_1742740

EPI_ISL_1742741

EPI_ISL_1742742

EPI_ISL_1742743

EPI_ISL_1742747

EPI_ISL_1742749

EPI_ISL_1742756

EPI_ISL_1742762

EPI_ISL_1742764

EPI_ISL_1742773

EPI_ISL_1742793

EPI_ISL_1742794

EPI_ISL_1742797

EPI_ISL_1742799

EPI_ISL_1742800

EPI_ISL_1742801

EPI_ISL_1742803

EPI_ISL_1742804

EPI_ISL_1742805

EPI_ISL_1742806

EPI_ISL_1742807

EPI_ISL_1742808

EPI_ISL_1742809

EPI_ISL_1742810

EPI_ISL_1742811

EPI_ISL_1742812

EPI_ISL_1742814

EPI_ISL_1742816

EPI_ISL_1742818

EPI_ISL_1742821

EPI_ISL_1742823

EPI_ISL_1742826

EPI_ISL_1742828

EPI_ISL_1743649

EPI_ISL_1743719

EPI_ISL_1743721

EPI_ISL_1743722

EPI_ISL_1743726

EPI_ISL_1743732

EPI_ISL_1743736

EPI_ISL_1743737

EPI_ISL_1743739

EPI_ISL_1743740

EPI_ISL_1743744

EPI_ISL_1743749

EPI_ISL_1743751

EPI_ISL_1743752

EPI_ISL_1743761

EPI_ISL_1743762

EPI_ISL_1743766

EPI_ISL_1743767

EPI_ISL_1743768

EPI_ISL_1743771

EPI_ISL_1743772

EPI_ISL_1743774

EPI_ISL_1743777

EPI_ISL_1743779

EPI_ISL_1743780

EPI_ISL_1743781

EPI_ISL_1743782

EPI_ISL_1743784

EPI_ISL_1743787

EPI_ISL_1743789

EPI_ISL_1743790

EPI_ISL_1743792

EPI_ISL_1743793

EPI_ISL_1743794

EPI_ISL_1743795

EPI_ISL_1743796

EPI_ISL_1743797

EPI_ISL_1743800

EPI_ISL_1743802

EPI_ISL_1743803

EPI_ISL_1743806

EPI_ISL_1743808

EPI_ISL_1743811

EPI_ISL_1743814

EPI_ISL_1743818

EPI_ISL_1743819

EPI_ISL_1743820

EPI_ISL_1743822

EPI_ISL_1743823

EPI_ISL_1743824

EPI_ISL_1743826

EPI_ISL_1743835

EPI_ISL_1743837

EPI_ISL_1743839

EPI_ISL_1743840

EPI_ISL_1743844

EPI_ISL_1743848

EPI_ISL_1743850

EPI_ISL_1743853

EPI_ISL_1743855

EPI_ISL_1743856

EPI_ISL_1743857

EPI_ISL_1743858

EPI_ISL_1743859

EPI_ISL_1743860

EPI_ISL_1743861

EPI_ISL_1743865

EPI_ISL_1743867

EPI_ISL_1743872

EPI_ISL_1743876

EPI_ISL_1743879

EPI_ISL_1743880

EPI_ISL_1743884

EPI_ISL_1743885

EPI_ISL_1743897

EPI_ISL_1743905

EPI_ISL_1743908

EPI_ISL_1743909

EPI_ISL_1743910

EPI_ISL_1743913

EPI_ISL_1743914

EPI_ISL_1743918

EPI_ISL_1743923

EPI_ISL_1743927

EPI_ISL_1743928

EPI_ISL_1743932

EPI_ISL_1743935

EPI_ISL_1743936

EPI_ISL_1743939

EPI_ISL_1743941

EPI_ISL_1743942

EPI_ISL_1743943

EPI_ISL_1743944

EPI_ISL_1743946

EPI_ISL_1743947

EPI_ISL_1743948

EPI_ISL_1743949

EPI_ISL_1743952

EPI_ISL_1743953

EPI_ISL_1743956

EPI_ISL_1743960

EPI_ISL_1743961

EPI_ISL_1743962

EPI_ISL_1743963

EPI_ISL_1743964

EPI_ISL_1743965

EPI_ISL_1743966

EPI_ISL_1743967

EPI_ISL_1743968

EPI_ISL_1743969

EPI_ISL_1743971

EPI_ISL_1743972

EPI_ISL_1743973

EPI_ISL_1743974

EPI_ISL_1743975

EPI_ISL_1743976

EPI_ISL_1743980

EPI_ISL_1743981

EPI_ISL_1743982

EPI_ISL_1743983

EPI_ISL_1743984

EPI_ISL_1743987

EPI_ISL_1743990

EPI_ISL_1743995

EPI_ISL_1743997

EPI_ISL_1744000

EPI_ISL_1744002

EPI_ISL_1744006

EPI_ISL_1744008

EPI_ISL_1744010

EPI_ISL_1744013

EPI_ISL_1744014

EPI_ISL_1744017

EPI_ISL_1744019

EPI_ISL_1744020

EPI_ISL_1744022

EPI_ISL_1744025

EPI_ISL_1744035

EPI_ISL_1744037

EPI_ISL_1744038

EPI_ISL_1744039

EPI_ISL_1744040

EPI_ISL_1744045

EPI_ISL_1744047

EPI_ISL_1744048

EPI_ISL_1744055

EPI_ISL_1744056

EPI_ISL_1744057

EPI_ISL_1744058

EPI_ISL_1744059

EPI_ISL_1744062

EPI_ISL_1744063

EPI_ISL_1744065

EPI_ISL_1744069

EPI_ISL_1744070

EPI_ISL_1744071

EPI_ISL_1744075

EPI_ISL_1744076

EPI_ISL_1744078

EPI_ISL_1744079

EPI_ISL_1744080

EPI_ISL_1744081

EPI_ISL_1744090

EPI_ISL_1744095

EPI_ISL_1744097

EPI_ISL_1744098

EPI_ISL_1744102

EPI_ISL_1744103

EPI_ISL_1744105

EPI_ISL_1744107

EPI_ISL_1744110

EPI_ISL_1744112

EPI_ISL_1744113

EPI_ISL_1744115

EPI_ISL_1744116

EPI_ISL_1744117

EPI_ISL_1744118

EPI_ISL_1744119

EPI_ISL_1744120

EPI_ISL_1744123

EPI_ISL_1744124

EPI_ISL_1744125

EPI_ISL_1744127

EPI_ISL_1744128

EPI_ISL_1744130

EPI_ISL_1744131

EPI_ISL_1744133

EPI_ISL_1744137

EPI_ISL_1744138

EPI_ISL_1744141

EPI_ISL_1744145

EPI_ISL_1744147

EPI_ISL_1744148

EPI_ISL_1744150

EPI_ISL_1744151

EPI_ISL_1744153

EPI_ISL_1744154

EPI_ISL_1744157

EPI_ISL_1744158

EPI_ISL_1744159

EPI_ISL_1744161

EPI_ISL_1744162

EPI_ISL_1744163

EPI_ISL_1744164

EPI_ISL_1744165

EPI_ISL_1744172

EPI_ISL_1744173

EPI_ISL_1744174

EPI_ISL_1744175

EPI_ISL_1744178

EPI_ISL_1744182

EPI_ISL_1744183

EPI_ISL_1744184

EPI_ISL_1744186

EPI_ISL_1744189

EPI_ISL_1744190

EPI_ISL_1744193

EPI_ISL_1744194

EPI_ISL_1744196

EPI_ISL_1744202

EPI_ISL_1744204

EPI_ISL_1744205

EPI_ISL_1744208

EPI_ISL_1744210

EPI_ISL_1744212

EPI_ISL_1744214

EPI_ISL_1744215

EPI_ISL_1744217

EPI_ISL_1744221

EPI_ISL_1744222

EPI_ISL_1744226

EPI_ISL_1744227

EPI_ISL_1744230

EPI_ISL_1744232

EPI_ISL_1744234

EPI_ISL_1744235

EPI_ISL_1744236

EPI_ISL_1744239

EPI_ISL_1744240

EPI_ISL_1744241

EPI_ISL_1744243

EPI_ISL_1744244

EPI_ISL_1744246

EPI_ISL_1744248

EPI_ISL_1744249

EPI_ISL_1744250

EPI_ISL_1744251

EPI_ISL_1744253

EPI_ISL_1744254

EPI_ISL_1744255

EPI_ISL_1744256

EPI_ISL_1744258

EPI_ISL_1744259

EPI_ISL_1744261

EPI_ISL_1744263

EPI_ISL_1744264

EPI_ISL_1744266

EPI_ISL_1744268

EPI_ISL_1744272

EPI_ISL_1744277

EPI_ISL_1744280

EPI_ISL_1744281

EPI_ISL_1744282

EPI_ISL_1744283

EPI_ISL_1744284

EPI_ISL_1744287

EPI_ISL_1744293

EPI_ISL_1744297

EPI_ISL_1744298

EPI_ISL_1744301

EPI_ISL_1744336

EPI_ISL_1744337

EPI_ISL_1744338

EPI_ISL_1744339

EPI_ISL_1744340

EPI_ISL_1744341

EPI_ISL_1744342

EPI_ISL_1744343

EPI_ISL_1744347

EPI_ISL_1744355

EPI_ISL_1744358

EPI_ISL_1744359

EPI_ISL_1744360

EPI_ISL_1744361

EPI_ISL_1744364

EPI_ISL_1744365

EPI_ISL_1744366

EPI_ISL_1744367

EPI_ISL_1744368

EPI_ISL_1744369

EPI_ISL_1744370

EPI_ISL_1744371

EPI_ISL_1744372

EPI_ISL_1744373

EPI_ISL_1744374

EPI_ISL_1744375

EPI_ISL_1744376

EPI_ISL_1744377

EPI_ISL_1744378

EPI_ISL_1744379

EPI_ISL_1744380

EPI_ISL_1744381

EPI_ISL_1744383

EPI_ISL_1744384

EPI_ISL_1744385

EPI_ISL_1744386

EPI_ISL_1744387

EPI_ISL_1744388

EPI_ISL_1744390

EPI_ISL_1744392

EPI_ISL_1744393

EPI_ISL_1744395

EPI_ISL_1744396

EPI_ISL_1744397

EPI_ISL_1744398

EPI_ISL_1744404

EPI_ISL_1744407

EPI_ISL_1744411

EPI_ISL_1744412

EPI_ISL_1744414

EPI_ISL_1744417

EPI_ISL_1744422

EPI_ISL_1744423

EPI_ISL_1744424

EPI_ISL_1744428

EPI_ISL_1744429

EPI_ISL_1744430

EPI_ISL_1744432

EPI_ISL_1744433

EPI_ISL_1744434

EPI_ISL_1744435

EPI_ISL_1744436

EPI_ISL_1744437

EPI_ISL_1744440

EPI_ISL_1744442

EPI_ISL_1744444

EPI_ISL_1744445

EPI_ISL_1744446

EPI_ISL_1744448

EPI_ISL_1744449

EPI_ISL_1744450

EPI_ISL_1744453

EPI_ISL_1744454

EPI_ISL_1744455

EPI_ISL_1744458

EPI_ISL_1744463

EPI_ISL_1744464

EPI_ISL_1744469

EPI_ISL_1744471

EPI_ISL_1744477

EPI_ISL_1744479

EPI_ISL_1744483

EPI_ISL_1744489

EPI_ISL_1744494

EPI_ISL_1744495

EPI_ISL_1744507

EPI_ISL_1744523

EPI_ISL_1744907

EPI_ISL_1744908

EPI_ISL_1744909

EPI_ISL_1744910

EPI_ISL_1744911

EPI_ISL_1744912

EPI_ISL_1744913

EPI_ISL_1744914

EPI_ISL_1744915

EPI_ISL_1744916

EPI_ISL_1744917

EPI_ISL_1744918

EPI_ISL_1744919

EPI_ISL_1744920

EPI_ISL_1744921

EPI_ISL_1744922

EPI_ISL_1744923

EPI_ISL_1744924

EPI_ISL_1744925

EPI_ISL_1744926

EPI_ISL_1744927

EPI_ISL_1744928

EPI_ISL_1744929

EPI_ISL_1744930

EPI_ISL_1744931

EPI_ISL_1744932

EPI_ISL_1744933

EPI_ISL_1744934

EPI_ISL_1744935

EPI_ISL_1744936

EPI_ISL_1744937

EPI_ISL_1744938

EPI_ISL_1744939

EPI_ISL_1744940

EPI_ISL_1744941

EPI_ISL_1744942

EPI_ISL_1744943

EPI_ISL_1744944

EPI_ISL_1744945

EPI_ISL_1744946

EPI_ISL_1744947

EPI_ISL_1744948

EPI_ISL_1744949

EPI_ISL_1744950

EPI_ISL_1744951

EPI_ISL_1744952

EPI_ISL_1744953

EPI_ISL_1744954

EPI_ISL_1744955

EPI_ISL_1744956

EPI_ISL_1744957

EPI_ISL_1744958

EPI_ISL_1744959

EPI_ISL_1744960

EPI_ISL_1744961

EPI_ISL_1744962

EPI_ISL_1744963

EPI_ISL_1744964

EPI_ISL_1744965

EPI_ISL_1744966

EPI_ISL_1744967

EPI_ISL_1744968

EPI_ISL_1744969

EPI_ISL_1744970

EPI_ISL_1744971

EPI_ISL_1744972

EPI_ISL_1744973

EPI_ISL_1744974

EPI_ISL_1744975

EPI_ISL_1744976

EPI_ISL_1744977

EPI_ISL_1744978

EPI_ISL_1744979

EPI_ISL_1744980

EPI_ISL_1744981

EPI_ISL_1744982

EPI_ISL_1744983

EPI_ISL_1744984

EPI_ISL_1744985

EPI_ISL_1744986

EPI_ISL_1744987

EPI_ISL_1744988

EPI_ISL_1744989

EPI_ISL_1744990

EPI_ISL_1744991

EPI_ISL_1744992

EPI_ISL_1744993

EPI_ISL_1744994

EPI_ISL_1744995

EPI_ISL_1744996

EPI_ISL_1744997

EPI_ISL_1744998

EPI_ISL_1744999

EPI_ISL_1745000

EPI_ISL_1745001

EPI_ISL_1745002

EPI_ISL_1745003

EPI_ISL_1745004

EPI_ISL_1745005

EPI_ISL_1745006

EPI_ISL_1745007

EPI_ISL_1745008

EPI_ISL_1745009

EPI_ISL_1745010

EPI_ISL_1745011

EPI_ISL_1745012

EPI_ISL_1745013

EPI_ISL_1745014

EPI_ISL_1745015

EPI_ISL_1745016

EPI_ISL_1745017

EPI_ISL_1745018

EPI_ISL_1745019

EPI_ISL_1745020

EPI_ISL_1745021

EPI_ISL_1745022

EPI_ISL_1745023

EPI_ISL_1745024

EPI_ISL_1745025

EPI_ISL_1745026

EPI_ISL_1745027

EPI_ISL_1745028

EPI_ISL_1745029

EPI_ISL_1745030

EPI_ISL_1745031

EPI_ISL_1745032

EPI_ISL_1745033

EPI_ISL_1745034

EPI_ISL_1745035

EPI_ISL_1745036

EPI_ISL_1745037

EPI_ISL_1745038

EPI_ISL_1745039

EPI_ISL_1745040

EPI_ISL_1745041

EPI_ISL_1745042

EPI_ISL_1745043

EPI_ISL_1745044

EPI_ISL_1745045

EPI_ISL_1745046

EPI_ISL_1745047

EPI_ISL_1745048

EPI_ISL_1745049

EPI_ISL_1745050

EPI_ISL_1745064

EPI_ISL_1745065

EPI_ISL_1745066

EPI_ISL_1745067

EPI_ISL_1745068

EPI_ISL_1745069

EPI_ISL_1745070

EPI_ISL_1745071

EPI_ISL_1745072

EPI_ISL_1745073

EPI_ISL_1745074

EPI_ISL_1745075

EPI_ISL_1745076

EPI_ISL_1745077

EPI_ISL_1745078

EPI_ISL_1745079

EPI_ISL_1745080

EPI_ISL_1745081

EPI_ISL_1745082

EPI_ISL_1745083

EPI_ISL_1745084

EPI_ISL_1745085

EPI_ISL_1745086

EPI_ISL_1745087

EPI_ISL_1745088

EPI_ISL_1745089

EPI_ISL_1745090

EPI_ISL_1745091

EPI_ISL_1745092

EPI_ISL_1745093

EPI_ISL_1745094

EPI_ISL_1745095

EPI_ISL_1745096

EPI_ISL_1745097

EPI_ISL_1745098

EPI_ISL_1745099

EPI_ISL_1745100

EPI_ISL_1745101

EPI_ISL_1745102

EPI_ISL_1745103

EPI_ISL_1745104

EPI_ISL_1745105

EPI_ISL_1745106

EPI_ISL_1745107

EPI_ISL_1745108

EPI_ISL_1745109

EPI_ISL_1745110

EPI_ISL_1745111

EPI_ISL_1745112

EPI_ISL_1745113

EPI_ISL_1745114

EPI_ISL_1745115

EPI_ISL_1745116

EPI_ISL_1745117

EPI_ISL_1745118

EPI_ISL_1745119

EPI_ISL_1745120

EPI_ISL_1745121

EPI_ISL_1745122

EPI_ISL_1745123

EPI_ISL_1745124

EPI_ISL_1745125

EPI_ISL_1745126

EPI_ISL_1745127

EPI_ISL_1745128

EPI_ISL_1745129

EPI_ISL_1745130

EPI_ISL_1745131

EPI_ISL_1745132

EPI_ISL_1745133

EPI_ISL_1745134

EPI_ISL_1745135

EPI_ISL_1745136

EPI_ISL_1745137

EPI_ISL_1745138

EPI_ISL_1745139

EPI_ISL_1745140

EPI_ISL_1745141

EPI_ISL_1745142

EPI_ISL_1745143

EPI_ISL_1745144

EPI_ISL_1745145

EPI_ISL_1745146

EPI_ISL_1745147

EPI_ISL_1745148

EPI_ISL_1745149

EPI_ISL_1745150

EPI_ISL_1745151

EPI_ISL_1745152

EPI_ISL_1745153

EPI_ISL_1745154

EPI_ISL_1745155

EPI_ISL_1745156

EPI_ISL_1745157

EPI_ISL_1745158

EPI_ISL_1745159

EPI_ISL_1745160

EPI_ISL_1745161

EPI_ISL_1745162

EPI_ISL_1745163

EPI_ISL_1745164

EPI_ISL_1745165

EPI_ISL_1745166

EPI_ISL_1745167

EPI_ISL_1745168

EPI_ISL_1745169

EPI_ISL_1745170

EPI_ISL_1745173

EPI_ISL_1745174

EPI_ISL_1745175

EPI_ISL_1745204

EPI_ISL_1745208

EPI_ISL_1745210

EPI_ISL_1745211

EPI_ISL_1745212

EPI_ISL_1745213

EPI_ISL_1745220

EPI_ISL_1745224

EPI_ISL_1745226

EPI_ISL_1745227

EPI_ISL_1745228

EPI_ISL_1745231

EPI_ISL_1745234

EPI_ISL_1745237

EPI_ISL_1745238

EPI_ISL_1745366

EPI_ISL_1745367

EPI_ISL_1745368

EPI_ISL_1745369

EPI_ISL_1745370

EPI_ISL_1745371

EPI_ISL_1745372

EPI_ISL_1745374

EPI_ISL_1745375

EPI_ISL_1745376

EPI_ISL_1745377

EPI_ISL_1745378

EPI_ISL_1745379

EPI_ISL_1745380

EPI_ISL_1745381

EPI_ISL_1745382

EPI_ISL_1745383

EPI_ISL_1745384

EPI_ISL_1745385

EPI_ISL_1745386

EPI_ISL_1745387

EPI_ISL_1745388

EPI_ISL_1745389

EPI_ISL_1745390

EPI_ISL_1745391

EPI_ISL_1745392

EPI_ISL_1745393

EPI_ISL_1745394

EPI_ISL_1745395

EPI_ISL_1745397

EPI_ISL_1745398

EPI_ISL_1745399

EPI_ISL_1745400

EPI_ISL_1745402

EPI_ISL_1745403

EPI_ISL_1745404

EPI_ISL_1745405

EPI_ISL_1745407

EPI_ISL_1745408

EPI_ISL_1745409

EPI_ISL_1745410

EPI_ISL_1745412

EPI_ISL_1745413

EPI_ISL_1745414

EPI_ISL_1745415

EPI_ISL_1745416

EPI_ISL_1745417

EPI_ISL_1745419

EPI_ISL_1745420

EPI_ISL_1745421

EPI_ISL_1745422

EPI_ISL_1745423

EPI_ISL_1745424

EPI_ISL_1745425

EPI_ISL_1745426

EPI_ISL_1745427

EPI_ISL_1745428

EPI_ISL_1745429

EPI_ISL_1745431

EPI_ISL_1745432

EPI_ISL_1745434

EPI_ISL_1745435

EPI_ISL_1745436

EPI_ISL_1745437

EPI_ISL_1745438

EPI_ISL_1745440

EPI_ISL_1745441

EPI_ISL_1745442

EPI_ISL_1745443

EPI_ISL_1745444

EPI_ISL_1745445

EPI_ISL_1745446

EPI_ISL_1745447

EPI_ISL_1745448

EPI_ISL_1745449

EPI_ISL_1745450

EPI_ISL_1745451

EPI_ISL_1745452

EPI_ISL_1745453

EPI_ISL_1745454

EPI_ISL_1745455

EPI_ISL_1745456

EPI_ISL_1745458

EPI_ISL_1745460

EPI_ISL_1745461

EPI_ISL_1745462

EPI_ISL_1745463

EPI_ISL_1745465

EPI_ISL_1745466

EPI_ISL_1745468

EPI_ISL_1745470

EPI_ISL_1745471

EPI_ISL_1745472

EPI_ISL_1745473

EPI_ISL_1745474

EPI_ISL_1745477

EPI_ISL_1745478

EPI_ISL_1745479

EPI_ISL_1745480

EPI_ISL_1745481

EPI_ISL_1745482

EPI_ISL_1745483

EPI_ISL_1745484

EPI_ISL_1745485

EPI_ISL_1745486

EPI_ISL_1745487

EPI_ISL_1745488

EPI_ISL_1745489

EPI_ISL_1745490

EPI_ISL_1745491

EPI_ISL_1745492

EPI_ISL_1745493

EPI_ISL_1745496

EPI_ISL_1745497

EPI_ISL_1745498

EPI_ISL_1745499

EPI_ISL_1745500

EPI_ISL_1745502

EPI_ISL_1745504

EPI_ISL_1745516

EPI_ISL_1745518

EPI_ISL_1745519

EPI_ISL_1745520

EPI_ISL_1745521

EPI_ISL_1745522

EPI_ISL_1745523

EPI_ISL_1745524

EPI_ISL_1745525

EPI_ISL_1745526

EPI_ISL_1745527

EPI_ISL_1745528

EPI_ISL_1745532

EPI_ISL_1745533

EPI_ISL_1745534

EPI_ISL_1745535

EPI_ISL_1745536

EPI_ISL_1745538

EPI_ISL_1745540

EPI_ISL_1745541

EPI_ISL_1745542

EPI_ISL_1745544

EPI_ISL_1745545

EPI_ISL_1745546

EPI_ISL_1745547

EPI_ISL_1745548

EPI_ISL_1745550

EPI_ISL_1745553

EPI_ISL_1745554

EPI_ISL_1745555

EPI_ISL_1745556

EPI_ISL_1745557

EPI_ISL_1745559

EPI_ISL_1745562

EPI_ISL_1745563

EPI_ISL_1745564

EPI_ISL_1745565

EPI_ISL_1745566

EPI_ISL_1745567

EPI_ISL_1745568

EPI_ISL_1745569

EPI_ISL_1745570

EPI_ISL_1745571

EPI_ISL_1745572

EPI_ISL_1745573

EPI_ISL_1745574

EPI_ISL_1745575

EPI_ISL_1745576

EPI_ISL_1745577

EPI_ISL_1745578

EPI_ISL_1745579

EPI_ISL_1745580

EPI_ISL_1745581

EPI_ISL_1745582

EPI_ISL_1745583

EPI_ISL_1745584

EPI_ISL_1745585

EPI_ISL_1745586

EPI_ISL_1745587

EPI_ISL_1745588

EPI_ISL_1745589

EPI_ISL_1745590

EPI_ISL_1745591

EPI_ISL_1745592

EPI_ISL_1745593

EPI_ISL_1745594

EPI_ISL_1745595

EPI_ISL_1745596

EPI_ISL_1745597

EPI_ISL_1745599

EPI_ISL_1745600

EPI_ISL_1745601

EPI_ISL_1745602

EPI_ISL_1745603

EPI_ISL_1745604

EPI_ISL_1745605

EPI_ISL_1745606

EPI_ISL_1745607

EPI_ISL_1745608

EPI_ISL_1745609

EPI_ISL_1745610

EPI_ISL_1745611

EPI_ISL_1745612

EPI_ISL_1745613

EPI_ISL_1745614

EPI_ISL_1745615

EPI_ISL_1745616

EPI_ISL_1745617

EPI_ISL_1745618

EPI_ISL_1745619

EPI_ISL_1745621

EPI_ISL_1745622

EPI_ISL_1745623

EPI_ISL_1745624

EPI_ISL_1745625

EPI_ISL_1745626

EPI_ISL_1745627

EPI_ISL_1745628

EPI_ISL_1745629

EPI_ISL_1745632

EPI_ISL_1745633

EPI_ISL_1745634

EPI_ISL_1745637

EPI_ISL_1745638

EPI_ISL_1745639

EPI_ISL_1745640

EPI_ISL_1745641

EPI_ISL_1745642

EPI_ISL_1745643

EPI_ISL_1745644

EPI_ISL_1745645

EPI_ISL_1745646

EPI_ISL_1745647

EPI_ISL_1745648

EPI_ISL_1745649

EPI_ISL_1745650

EPI_ISL_1745651

EPI_ISL_1745653

EPI_ISL_1745654

EPI_ISL_1745655

EPI_ISL_1745656

EPI_ISL_1745657

EPI_ISL_1745658

EPI_ISL_1745659

EPI_ISL_1745660

EPI_ISL_1745661

EPI_ISL_1745662

EPI_ISL_1745663

EPI_ISL_1745664

EPI_ISL_1745665

EPI_ISL_1745666

EPI_ISL_1745667

EPI_ISL_1745668

EPI_ISL_1745669

EPI_ISL_1745670

EPI_ISL_1745671

EPI_ISL_1745672

EPI_ISL_1745673

EPI_ISL_1745674

EPI_ISL_1745675

EPI_ISL_1745678

EPI_ISL_1745679

EPI_ISL_1745680

EPI_ISL_1745681

EPI_ISL_1745683

EPI_ISL_1745687

EPI_ISL_1745688

EPI_ISL_1745695

EPI_ISL_1745696

EPI_ISL_1745699

EPI_ISL_1745700

EPI_ISL_1745701

EPI_ISL_1745702

EPI_ISL_1745703

EPI_ISL_1745704

EPI_ISL_1745705

EPI_ISL_1745706

EPI_ISL_1745707

EPI_ISL_1745709

EPI_ISL_1745710

EPI_ISL_1745711

EPI_ISL_1745712

EPI_ISL_1745713

EPI_ISL_1745714

EPI_ISL_1745751

EPI_ISL_1745754

EPI_ISL_1745755

EPI_ISL_1745756

EPI_ISL_1745777

EPI_ISL_1745778

EPI_ISL_1745789

EPI_ISL_1745790

EPI_ISL_1745791

EPI_ISL_1745794

EPI_ISL_1745807

EPI_ISL_1745808

EPI_ISL_1745809

EPI_ISL_1745813

EPI_ISL_1745814

EPI_ISL_1745821

EPI_ISL_1745822

EPI_ISL_1745823

EPI_ISL_1745864

EPI_ISL_1745868

EPI_ISL_1745869

EPI_ISL_1745870

EPI_ISL_1745872

EPI_ISL_1745877

EPI_ISL_1745878

EPI_ISL_1745879

EPI_ISL_1745883

EPI_ISL_1745884

EPI_ISL_1745885

EPI_ISL_1745886

EPI_ISL_1745887

EPI_ISL_1745888

EPI_ISL_1745889

EPI_ISL_1745890

EPI_ISL_1745891

EPI_ISL_1745899

EPI_ISL_1745900

EPI_ISL_1745901

EPI_ISL_1745907

EPI_ISL_1745908

EPI_ISL_1745914

EPI_ISL_1745915

EPI_ISL_1745916

EPI_ISL_1745922

EPI_ISL_1745925

EPI_ISL_1745966

EPI_ISL_1745967

EPI_ISL_1745968

EPI_ISL_1745970

EPI_ISL_1745971

EPI_ISL_1745988

EPI_ISL_1746017

EPI_ISL_1746019

EPI_ISL_1746041

EPI_ISL_1746076

EPI_ISL_1746077

EPI_ISL_1746078

EPI_ISL_1746080

EPI_ISL_1746081

EPI_ISL_1746082

EPI_ISL_1746083

EPI_ISL_1746084

EPI_ISL_1746085

EPI_ISL_1746086

EPI_ISL_1746091

EPI_ISL_1746092

EPI_ISL_1746093

EPI_ISL_1746111

EPI_ISL_1746120

EPI_ISL_1746121

EPI_ISL_1746122

EPI_ISL_1746469

EPI_ISL_1746538

EPI_ISL_1746539

EPI_ISL_1746544

EPI_ISL_1746545

EPI_ISL_1746546

EPI_ISL_1746547

EPI_ISL_1746548

EPI_ISL_1746549

EPI_ISL_1746550

EPI_ISL_1746551

EPI_ISL_1746552

EPI_ISL_1746553

EPI_ISL_1746558

EPI_ISL_1746559

EPI_ISL_1746560

EPI_ISL_1746561

EPI_ISL_1746562

EPI_ISL_1746563

EPI_ISL_1746564

EPI_ISL_1746565

EPI_ISL_1746566

EPI_ISL_1746567

EPI_ISL_1746568

EPI_ISL_1746569

EPI_ISL_1746570

EPI_ISL_1746571

EPI_ISL_1746572

EPI_ISL_1746573

EPI_ISL_1746574

EPI_ISL_1746575

EPI_ISL_1746576

EPI_ISL_1746577

EPI_ISL_1746578

EPI_ISL_1746579

EPI_ISL_1746580

EPI_ISL_1746581

EPI_ISL_1746582

EPI_ISL_1746583

EPI_ISL_1746584

EPI_ISL_1746585

EPI_ISL_1746586

EPI_ISL_1746587

EPI_ISL_1746588

EPI_ISL_1746589

EPI_ISL_1746590

EPI_ISL_1746591

EPI_ISL_1746592

EPI_ISL_1746593

EPI_ISL_1746594

EPI_ISL_1746595

EPI_ISL_1746596

EPI_ISL_1746597

EPI_ISL_1746598

EPI_ISL_1746599

EPI_ISL_1746600

EPI_ISL_1746601

EPI_ISL_1746602

EPI_ISL_1746603

EPI_ISL_1746604

EPI_ISL_1746605

EPI_ISL_1746606

EPI_ISL_1746607

EPI_ISL_1746608

EPI_ISL_1746609

EPI_ISL_1746610

EPI_ISL_1746611

EPI_ISL_1746612

EPI_ISL_1746613

EPI_ISL_1746614

EPI_ISL_1746615

EPI_ISL_1746616

EPI_ISL_1746617

EPI_ISL_1746618

EPI_ISL_1746619

EPI_ISL_1746620

EPI_ISL_1746621

EPI_ISL_1746622

EPI_ISL_1746623

EPI_ISL_1746624

EPI_ISL_1746625

EPI_ISL_1746626

EPI_ISL_1746627

EPI_ISL_1746628

EPI_ISL_1746629

EPI_ISL_1746630

EPI_ISL_1746631

EPI_ISL_1746632

EPI_ISL_1746633

EPI_ISL_1746634

EPI_ISL_1746635

EPI_ISL_1746636

EPI_ISL_1746637

EPI_ISL_1746638

EPI_ISL_1746639

EPI_ISL_1746640

EPI_ISL_1746641

EPI_ISL_1746642

EPI_ISL_1746643

EPI_ISL_1746644

EPI_ISL_1746645

EPI_ISL_1746646

EPI_ISL_1746647

EPI_ISL_1746648

EPI_ISL_1746649

EPI_ISL_1746650

EPI_ISL_1746651

EPI_ISL_1746652

EPI_ISL_1746653

EPI_ISL_1746654

EPI_ISL_1746655

EPI_ISL_1746656

EPI_ISL_1746657

EPI_ISL_1746658

EPI_ISL_1746659

EPI_ISL_1746660

EPI_ISL_1746661

EPI_ISL_1746662

EPI_ISL_1746663

EPI_ISL_1746664

EPI_ISL_1746665

EPI_ISL_1746666

EPI_ISL_1746667

EPI_ISL_1746668

EPI_ISL_1746669

EPI_ISL_1746670

EPI_ISL_1746671

EPI_ISL_1746672

EPI_ISL_1746673

EPI_ISL_1746674

EPI_ISL_1746675

EPI_ISL_1746676

EPI_ISL_1746677

EPI_ISL_1746678

EPI_ISL_1746679

EPI_ISL_1746680

EPI_ISL_1746681

EPI_ISL_1746682

EPI_ISL_1746683

EPI_ISL_1746684

EPI_ISL_1746685

EPI_ISL_1746686

EPI_ISL_1746687

EPI_ISL_1746688

EPI_ISL_1746689

EPI_ISL_1746690

EPI_ISL_1746691

EPI_ISL_1746692

EPI_ISL_1746693

EPI_ISL_1746694

EPI_ISL_1746695

EPI_ISL_1746696

EPI_ISL_1746697

EPI_ISL_1746698

EPI_ISL_1746699

EPI_ISL_1746700

EPI_ISL_1746701

EPI_ISL_1746705

EPI_ISL_1746706

EPI_ISL_1746707

EPI_ISL_1746708

EPI_ISL_1746709

EPI_ISL_1746710

EPI_ISL_1746715

EPI_ISL_1746716

EPI_ISL_1746717

EPI_ISL_1746718

EPI_ISL_1746719

EPI_ISL_1746720

EPI_ISL_1746721

EPI_ISL_1746722

EPI_ISL_1746723

EPI_ISL_1746724

EPI_ISL_1746725

EPI_ISL_1746726

EPI_ISL_1746727

EPI_ISL_1746728

EPI_ISL_1746729

EPI_ISL_1746731

EPI_ISL_1746732

EPI_ISL_1746733

EPI_ISL_1746734

EPI_ISL_1746735

EPI_ISL_1746736

EPI_ISL_1746737

EPI_ISL_1746738

EPI_ISL_1746739

EPI_ISL_1746740

EPI_ISL_1746741

EPI_ISL_1746742

EPI_ISL_1746743

EPI_ISL_1746744

EPI_ISL_1746745

EPI_ISL_1746746

EPI_ISL_1746747

EPI_ISL_1746748

EPI_ISL_1746749

EPI_ISL_1746750

EPI_ISL_1746751

EPI_ISL_1746752

EPI_ISL_1746753

EPI_ISL_1746754

EPI_ISL_1746755

EPI_ISL_1746756

EPI_ISL_1746757

EPI_ISL_1746758

EPI_ISL_1746759

EPI_ISL_1746760

EPI_ISL_1746761

EPI_ISL_1746762

EPI_ISL_1746763

EPI_ISL_1746764

EPI_ISL_1746765

EPI_ISL_1746766

EPI_ISL_1746767

EPI_ISL_1746768

EPI_ISL_1746769

EPI_ISL_1746770

EPI_ISL_1746771

EPI_ISL_1746772

EPI_ISL_1746773

EPI_ISL_1746774

EPI_ISL_1746775

EPI_ISL_1746776

EPI_ISL_1746777

EPI_ISL_1746778

EPI_ISL_1746779

EPI_ISL_1746780

EPI_ISL_1746781

EPI_ISL_1746782

EPI_ISL_1746783

EPI_ISL_1746784

EPI_ISL_1746785

EPI_ISL_1746786

EPI_ISL_1746787

EPI_ISL_1746788

EPI_ISL_1746789

EPI_ISL_1746790

EPI_ISL_1746791

EPI_ISL_1746792

EPI_ISL_1746793

EPI_ISL_1746794

EPI_ISL_1746795

EPI_ISL_1746796

EPI_ISL_1746797

EPI_ISL_1746798

EPI_ISL_1746799

EPI_ISL_1746800

EPI_ISL_1746801

EPI_ISL_1746802

EPI_ISL_1746803

EPI_ISL_1746804

EPI_ISL_1746805

EPI_ISL_1746806

EPI_ISL_1746807

EPI_ISL_1746808

EPI_ISL_1746809

EPI_ISL_1746810

EPI_ISL_1746811

EPI_ISL_1746812

EPI_ISL_1746813

EPI_ISL_1746814

EPI_ISL_1746815

EPI_ISL_1746816

EPI_ISL_1746817

EPI_ISL_1746818

EPI_ISL_1746819

EPI_ISL_1746820

EPI_ISL_1746821

EPI_ISL_1746822

EPI_ISL_1746823

EPI_ISL_1746824

EPI_ISL_1746825

EPI_ISL_1746826

EPI_ISL_1747392

EPI_ISL_1747401

EPI_ISL_1758226

EPI_ISL_1747405

EPI_ISL_1747409

EPI_ISL_1747410

EPI_ISL_1747412

EPI_ISL_1747413

EPI_ISL_1747416

EPI_ISL_1747417

EPI_ISL_1747422

EPI_ISL_1747426

EPI_ISL_1747428

EPI_ISL_1747433

EPI_ISL_1747434

EPI_ISL_1747438

EPI_ISL_1747439

EPI_ISL_1747446

EPI_ISL_1747449

EPI_ISL_1747453

EPI_ISL_1747457

EPI_ISL_1747459

EPI_ISL_1748443

EPI_ISL_1748448

EPI_ISL_1748463

EPI_ISL_1749082

EPI_ISL_1749101

EPI_ISL_1749103

EPI_ISL_1749109

EPI_ISL_1749110

EPI_ISL_1758229

EPI_ISL_1758230

EPI_ISL_1758231

EPI_ISL_1749430

EPI_ISL_1749431

EPI_ISL_1749435

EPI_ISL_1749452

EPI_ISL_1749453

EPI_ISL_1749454

EPI_ISL_1749455

EPI_ISL_1749456

EPI_ISL_1749457

EPI_ISL_1749458

EPI_ISL_1749459

EPI_ISL_1749460

EPI_ISL_1749461

EPI_ISL_1749462

EPI_ISL_1749463

EPI_ISL_1749464

EPI_ISL_1749465

EPI_ISL_1749466

EPI_ISL_1749467

EPI_ISL_1749468

EPI_ISL_1749469

EPI_ISL_1749470

EPI_ISL_1749471

EPI_ISL_1749472

EPI_ISL_1749473

EPI_ISL_1749475

EPI_ISL_1749476

EPI_ISL_1749477

EPI_ISL_1749478

EPI_ISL_1749479

EPI_ISL_1749480

EPI_ISL_1749481

EPI_ISL_1749482

EPI_ISL_1749483

EPI_ISL_1749485

EPI_ISL_1749486

EPI_ISL_1749487

EPI_ISL_1749488

EPI_ISL_1749489

EPI_ISL_1749490

EPI_ISL_1749491

EPI_ISL_1749492

EPI_ISL_1749493

EPI_ISL_1749494

EPI_ISL_1749495

EPI_ISL_1749496

EPI_ISL_1749497

EPI_ISL_1749498

EPI_ISL_1749499

EPI_ISL_1749500

EPI_ISL_1749501

EPI_ISL_1749502

EPI_ISL_1749503

EPI_ISL_1749504

EPI_ISL_1749505

EPI_ISL_1749506

EPI_ISL_1749507

EPI_ISL_1749508

EPI_ISL_1749509

EPI_ISL_1749510

EPI_ISL_1749511

EPI_ISL_1749512

EPI_ISL_1749513

EPI_ISL_1749514

EPI_ISL_1749515

EPI_ISL_1749516

EPI_ISL_1749517

EPI_ISL_1749518

EPI_ISL_1749519

EPI_ISL_1749520

EPI_ISL_1749521

EPI_ISL_1749522

EPI_ISL_1749523

EPI_ISL_1749524

EPI_ISL_1749525

EPI_ISL_1749526

EPI_ISL_1749527

EPI_ISL_1749528

EPI_ISL_1749529

EPI_ISL_1749530

EPI_ISL_1749531

EPI_ISL_1749532

EPI_ISL_1749533

EPI_ISL_1749534

EPI_ISL_1749535

EPI_ISL_1749536

EPI_ISL_1749537

EPI_ISL_1749538

EPI_ISL_1749539

EPI_ISL_1749540

EPI_ISL_1749541

EPI_ISL_1749542

EPI_ISL_1749543

EPI_ISL_1749544

EPI_ISL_1749545

EPI_ISL_1749546

EPI_ISL_1749547

EPI_ISL_1749548

EPI_ISL_1749549

EPI_ISL_1749550

EPI_ISL_1749551

EPI_ISL_1749552

EPI_ISL_1749553

EPI_ISL_1749554

EPI_ISL_1749555

EPI_ISL_1749556

EPI_ISL_1749557

EPI_ISL_1749558

EPI_ISL_1749559

EPI_ISL_1749560

EPI_ISL_1749561

EPI_ISL_1749562

EPI_ISL_1749563

EPI_ISL_1749565

EPI_ISL_1749566

EPI_ISL_1749567

EPI_ISL_1749568

EPI_ISL_1749569

EPI_ISL_1749570

EPI_ISL_1749571

EPI_ISL_1749572

EPI_ISL_1749575

EPI_ISL_1749576

EPI_ISL_1749577

EPI_ISL_1749578

EPI_ISL_1749579

EPI_ISL_1749580

EPI_ISL_1749581

EPI_ISL_1749582

EPI_ISL_1749583

EPI_ISL_1749584

EPI_ISL_1749585

EPI_ISL_1749589

EPI_ISL_1749590

EPI_ISL_1749591

EPI_ISL_1749592

EPI_ISL_1749593

EPI_ISL_1749594

EPI_ISL_1749595

EPI_ISL_1749596

EPI_ISL_1749597

EPI_ISL_1749598

EPI_ISL_1749599

EPI_ISL_1749600

EPI_ISL_1749601

EPI_ISL_1749602

EPI_ISL_1749605

EPI_ISL_1749606

EPI_ISL_1749607

EPI_ISL_1749608

EPI_ISL_1749609

EPI_ISL_1749612

EPI_ISL_1749613

EPI_ISL_1749614

EPI_ISL_1749615

EPI_ISL_1749616

EPI_ISL_1749809

EPI_ISL_1749810

EPI_ISL_1749811

EPI_ISL_1749812

EPI_ISL_1749813

EPI_ISL_1749814

EPI_ISL_1749815

EPI_ISL_1749816

EPI_ISL_1749819

EPI_ISL_1749820

EPI_ISL_1749821

EPI_ISL_1749822

EPI_ISL_1749823

EPI_ISL_1749824

EPI_ISL_1749825

EPI_ISL_1749826

EPI_ISL_1749827

EPI_ISL_1749828

EPI_ISL_1749829

EPI_ISL_1749830

EPI_ISL_1749831

EPI_ISL_1749832

EPI_ISL_1749833

EPI_ISL_1749834

EPI_ISL_1749835

EPI_ISL_1749836

EPI_ISL_1749837

EPI_ISL_1749838

EPI_ISL_1749839

EPI_ISL_1749840

EPI_ISL_1749841

EPI_ISL_1749842

EPI_ISL_1749843

EPI_ISL_1749844

EPI_ISL_1749845

EPI_ISL_1749846

EPI_ISL_1749847

EPI_ISL_1749848

EPI_ISL_1749849

EPI_ISL_1749850

EPI_ISL_1749851

EPI_ISL_1749852

EPI_ISL_1749853

EPI_ISL_1749854

EPI_ISL_1749855

EPI_ISL_1749856

EPI_ISL_1749857

EPI_ISL_1749858

EPI_ISL_1749859

EPI_ISL_1749860

EPI_ISL_1749861

EPI_ISL_1749862

EPI_ISL_1749863

EPI_ISL_1749864

EPI_ISL_1749865

EPI_ISL_1749866

EPI_ISL_1749867

EPI_ISL_1749868

EPI_ISL_1749869

EPI_ISL_1749870

EPI_ISL_1749871

EPI_ISL_1749872

EPI_ISL_1749873

EPI_ISL_1749874

EPI_ISL_1749875

EPI_ISL_1749876

EPI_ISL_1749877

EPI_ISL_1749878

EPI_ISL_1749879

EPI_ISL_1749880

EPI_ISL_1749881

EPI_ISL_1749882

EPI_ISL_1749883

EPI_ISL_1749884

EPI_ISL_1749885

EPI_ISL_1749886

EPI_ISL_1749887

EPI_ISL_1749888

EPI_ISL_1749889

EPI_ISL_1749890

EPI_ISL_1749891

EPI_ISL_1749892

EPI_ISL_1749893

EPI_ISL_1749894

EPI_ISL_1749895

EPI_ISL_1749896

EPI_ISL_1749897

EPI_ISL_1749898

EPI_ISL_1749899

EPI_ISL_1749900

EPI_ISL_1749901

EPI_ISL_1749902

EPI_ISL_1749903

EPI_ISL_1749904

EPI_ISL_1749905

EPI_ISL_1749906

EPI_ISL_1749907

EPI_ISL_1749908

EPI_ISL_1749909

EPI_ISL_1749910

EPI_ISL_1749911

EPI_ISL_1749912

EPI_ISL_1749913

EPI_ISL_1749914

EPI_ISL_1749915

EPI_ISL_1749916

EPI_ISL_1749917

EPI_ISL_1749918

EPI_ISL_1749919

EPI_ISL_1749920

EPI_ISL_1749921

EPI_ISL_1749922

EPI_ISL_1749923

EPI_ISL_1749924

EPI_ISL_1749925

EPI_ISL_1749926

EPI_ISL_1749927

EPI_ISL_1749928

EPI_ISL_1749929

EPI_ISL_1749930

EPI_ISL_1749931

EPI_ISL_1749932

EPI_ISL_1749933

EPI_ISL_1749934

EPI_ISL_1749935

EPI_ISL_1749936

EPI_ISL_1749937

EPI_ISL_1749938

EPI_ISL_1749939

EPI_ISL_1749940

EPI_ISL_1749941

EPI_ISL_1749942

EPI_ISL_1749943

EPI_ISL_1749944

EPI_ISL_1749945

EPI_ISL_1749946

EPI_ISL_1749947

EPI_ISL_1749948

EPI_ISL_1749949

EPI_ISL_1749950

EPI_ISL_1749951

EPI_ISL_1749952

EPI_ISL_1749953

EPI_ISL_1749954

EPI_ISL_1749955

EPI_ISL_1749956

EPI_ISL_1749957

EPI_ISL_1749958

EPI_ISL_1749959

EPI_ISL_1749960

EPI_ISL_1749961

EPI_ISL_1749962

EPI_ISL_1749963

EPI_ISL_1749964

EPI_ISL_1749965

EPI_ISL_1749966

EPI_ISL_1749967

EPI_ISL_1749968

EPI_ISL_1749969

EPI_ISL_1749970

EPI_ISL_1749971

EPI_ISL_1749972

EPI_ISL_1749973

EPI_ISL_1749974

EPI_ISL_1749975

EPI_ISL_1749976

EPI_ISL_1749977

EPI_ISL_1749978

EPI_ISL_1749979

EPI_ISL_1749980

EPI_ISL_1749981

EPI_ISL_1749982

EPI_ISL_1749983

EPI_ISL_1749984

EPI_ISL_1749985

EPI_ISL_1749986

EPI_ISL_1749987

EPI_ISL_1749988

EPI_ISL_1749989

EPI_ISL_1749990

EPI_ISL_1749991

EPI_ISL_1749992

EPI_ISL_1749993

EPI_ISL_1749994

EPI_ISL_1749995

EPI_ISL_1749996

EPI_ISL_1749997

EPI_ISL_1749998

EPI_ISL_1749999

EPI_ISL_1750000

EPI_ISL_1750001

EPI_ISL_1750002

EPI_ISL_1750003

EPI_ISL_1750004

EPI_ISL_1750005

EPI_ISL_1750006

EPI_ISL_1750007

EPI_ISL_1750008

EPI_ISL_1750009

EPI_ISL_1750010

EPI_ISL_1750011

EPI_ISL_1750012

EPI_ISL_1750013

EPI_ISL_1750014

EPI_ISL_1750015

EPI_ISL_1750016

EPI_ISL_1750017

EPI_ISL_1750018

EPI_ISL_1750019

EPI_ISL_1750020

EPI_ISL_1750021

EPI_ISL_1750022

EPI_ISL_1750023

EPI_ISL_1750024

EPI_ISL_1750025

EPI_ISL_1750026

EPI_ISL_1750027

EPI_ISL_1750028

EPI_ISL_1750029

EPI_ISL_1750030

EPI_ISL_1750031

EPI_ISL_1750032

EPI_ISL_1750033

EPI_ISL_1750034

EPI_ISL_1750035

EPI_ISL_1750036

EPI_ISL_1750037

EPI_ISL_1750039

EPI_ISL_1750040

EPI_ISL_1750041

EPI_ISL_1750042

EPI_ISL_1750043

EPI_ISL_1750044

EPI_ISL_1750045

EPI_ISL_1750046

EPI_ISL_1750047

EPI_ISL_1750048

EPI_ISL_1750049

EPI_ISL_1750050

EPI_ISL_1750051

EPI_ISL_1750052

EPI_ISL_1750053

EPI_ISL_1750054

EPI_ISL_1750055

EPI_ISL_1750056

EPI_ISL_1750057

EPI_ISL_1750058

EPI_ISL_1750059

EPI_ISL_1750060

EPI_ISL_1750061

EPI_ISL_1750062

EPI_ISL_1750063

EPI_ISL_1750064

EPI_ISL_1750065

EPI_ISL_1750066

EPI_ISL_1750067

EPI_ISL_1750068

EPI_ISL_1750069

EPI_ISL_1750070

EPI_ISL_1750071

EPI_ISL_1750072

EPI_ISL_1750073

EPI_ISL_1750074

EPI_ISL_1750075

EPI_ISL_1750076

EPI_ISL_1750077

EPI_ISL_1750078

EPI_ISL_1750079

EPI_ISL_1750080

EPI_ISL_1750081

EPI_ISL_1750082

EPI_ISL_1750083

EPI_ISL_1750084

EPI_ISL_1750085

EPI_ISL_1750086

EPI_ISL_1750087

EPI_ISL_1750088

EPI_ISL_1750089

EPI_ISL_1750090

EPI_ISL_1750091

EPI_ISL_1750092

EPI_ISL_1750093

EPI_ISL_1750094

EPI_ISL_1750095

EPI_ISL_1750096

EPI_ISL_1750097

EPI_ISL_1750098

EPI_ISL_1750099

EPI_ISL_1750100

EPI_ISL_1750101

EPI_ISL_1750102

EPI_ISL_1750103

EPI_ISL_1750104

EPI_ISL_1750105

EPI_ISL_1750106

EPI_ISL_1750107

EPI_ISL_1750108

EPI_ISL_1750109

EPI_ISL_1750110

EPI_ISL_1750111

EPI_ISL_1750112

EPI_ISL_1750113

EPI_ISL_1750114

EPI_ISL_1750115

EPI_ISL_1750116

EPI_ISL_1750117

EPI_ISL_1750118

EPI_ISL_1750119

EPI_ISL_1750120

EPI_ISL_1750121

EPI_ISL_1750122

EPI_ISL_1750123

EPI_ISL_1750124

EPI_ISL_1750125

EPI_ISL_1750126

EPI_ISL_1750127

EPI_ISL_1750128

EPI_ISL_1750129

EPI_ISL_1750130

EPI_ISL_1750131

EPI_ISL_1750132

EPI_ISL_1750133

EPI_ISL_1750134

EPI_ISL_1750135

EPI_ISL_1750136

EPI_ISL_1750137

EPI_ISL_1750138

EPI_ISL_1750139

EPI_ISL_1750140

EPI_ISL_1750141

EPI_ISL_1750142

EPI_ISL_1750143

EPI_ISL_1750144

EPI_ISL_1750145

EPI_ISL_1750146

EPI_ISL_1750147

EPI_ISL_1750148

EPI_ISL_1750149

EPI_ISL_1750150

EPI_ISL_1750151

EPI_ISL_1750152

EPI_ISL_1750153

EPI_ISL_1750154

EPI_ISL_1750155

EPI_ISL_1750156

EPI_ISL_1750157

EPI_ISL_1750158

EPI_ISL_1750159

EPI_ISL_1750160

EPI_ISL_1750161

EPI_ISL_1750162

EPI_ISL_1750163

EPI_ISL_1750164

EPI_ISL_1750165

EPI_ISL_1750166

EPI_ISL_1750167

EPI_ISL_1750168

EPI_ISL_1750169

EPI_ISL_1750170

EPI_ISL_1750171

EPI_ISL_1750172

EPI_ISL_1750173

EPI_ISL_1750174

EPI_ISL_1750175

EPI_ISL_1750176

EPI_ISL_1750177

EPI_ISL_1750178

EPI_ISL_1750179

EPI_ISL_1750180

EPI_ISL_1750181

EPI_ISL_1750182

EPI_ISL_1750183

EPI_ISL_1750184

EPI_ISL_1750185

EPI_ISL_1750186

EPI_ISL_1750187

EPI_ISL_1750188

EPI_ISL_1750189

EPI_ISL_1750190

EPI_ISL_1750191

EPI_ISL_1750192

EPI_ISL_1750193

EPI_ISL_1750194

EPI_ISL_1750195

EPI_ISL_1750196

EPI_ISL_1750197

EPI_ISL_1750198

EPI_ISL_1750199

EPI_ISL_1750200

EPI_ISL_1750201

EPI_ISL_1750202

EPI_ISL_1750203

EPI_ISL_1750204

EPI_ISL_1750205

EPI_ISL_1750206

EPI_ISL_1750207

EPI_ISL_1750208

EPI_ISL_1750209

EPI_ISL_1750210

EPI_ISL_1750211

EPI_ISL_1750212

EPI_ISL_1750213

EPI_ISL_1750214

EPI_ISL_1750215

EPI_ISL_1750216

EPI_ISL_1750217

EPI_ISL_1750218

EPI_ISL_1750219

EPI_ISL_1750220

EPI_ISL_1750221

EPI_ISL_1750222

EPI_ISL_1750223

EPI_ISL_1750224

EPI_ISL_1750225

EPI_ISL_1750226

EPI_ISL_1750227

EPI_ISL_1750228

EPI_ISL_1750229

EPI_ISL_1750230

EPI_ISL_1750231

EPI_ISL_1750232

EPI_ISL_1750233

EPI_ISL_1750234

EPI_ISL_1750235

EPI_ISL_1750236

EPI_ISL_1750237

EPI_ISL_1750238

EPI_ISL_1750239

EPI_ISL_1750240

EPI_ISL_1750241

EPI_ISL_1750242

EPI_ISL_1750243

EPI_ISL_1750244

EPI_ISL_1750245

EPI_ISL_1750246

EPI_ISL_1750247

EPI_ISL_1750248

EPI_ISL_1750249

EPI_ISL_1750250

EPI_ISL_1750251

EPI_ISL_1750252

EPI_ISL_1750253

EPI_ISL_1750254

EPI_ISL_1750255

EPI_ISL_1750256

EPI_ISL_1750257

EPI_ISL_1750258

EPI_ISL_1750259

EPI_ISL_1750260

EPI_ISL_1750261

EPI_ISL_1750262

EPI_ISL_1750263

EPI_ISL_1750264

EPI_ISL_1750265

EPI_ISL_1750266

EPI_ISL_1750267

EPI_ISL_1750268

EPI_ISL_1750269

EPI_ISL_1750270

EPI_ISL_1750271

EPI_ISL_1750272

EPI_ISL_1750273

EPI_ISL_1750274

EPI_ISL_1750275

EPI_ISL_1750276

EPI_ISL_1750277

EPI_ISL_1750278

EPI_ISL_1750279

EPI_ISL_1750280

EPI_ISL_1750281

EPI_ISL_1750282

EPI_ISL_1750283

EPI_ISL_1750284

EPI_ISL_1750285

EPI_ISL_1750286

EPI_ISL_1750287

EPI_ISL_1750288

EPI_ISL_1750289

EPI_ISL_1750290

EPI_ISL_1750291

EPI_ISL_1750292

EPI_ISL_1750293

EPI_ISL_1750294

EPI_ISL_1750295

EPI_ISL_1750296

EPI_ISL_1750297

EPI_ISL_1750298

EPI_ISL_1750299

EPI_ISL_1750300

EPI_ISL_1750301

EPI_ISL_1750302

EPI_ISL_1750303

EPI_ISL_1750304

EPI_ISL_1750305

EPI_ISL_1750306

EPI_ISL_1750307

EPI_ISL_1750308

EPI_ISL_1750309

EPI_ISL_1750310

EPI_ISL_1750311

EPI_ISL_1750312

EPI_ISL_1750313

EPI_ISL_1750314

EPI_ISL_1750315

EPI_ISL_1750316

EPI_ISL_1750317

EPI_ISL_1750318

EPI_ISL_1750319

EPI_ISL_1750320

EPI_ISL_1750321

EPI_ISL_1750322

EPI_ISL_1750323

EPI_ISL_1750324

EPI_ISL_1750325

EPI_ISL_1750326

EPI_ISL_1750327

EPI_ISL_1750328

EPI_ISL_1750329

EPI_ISL_1750330

EPI_ISL_1750331

EPI_ISL_1750332

EPI_ISL_1750333

EPI_ISL_1750334

EPI_ISL_1750335

EPI_ISL_1750336

EPI_ISL_1750337

EPI_ISL_1750338

EPI_ISL_1750339

EPI_ISL_1750340

EPI_ISL_1750341

EPI_ISL_1750342

EPI_ISL_1750343

EPI_ISL_1750344

EPI_ISL_1750345

EPI_ISL_1750357

EPI_ISL_1750358

EPI_ISL_1750359

EPI_ISL_1750360

EPI_ISL_1750361

EPI_ISL_1750362

EPI_ISL_1750363

EPI_ISL_1750364

EPI_ISL_1750365

EPI_ISL_1750366

EPI_ISL_1750367

EPI_ISL_1750368

EPI_ISL_1750369

EPI_ISL_1750370

EPI_ISL_1750371

EPI_ISL_1750372

EPI_ISL_1750373

EPI_ISL_1750374

EPI_ISL_1750375

EPI_ISL_1750376

EPI_ISL_1750377

EPI_ISL_1750378

EPI_ISL_1750379

EPI_ISL_1750380

EPI_ISL_1750381

EPI_ISL_1750382

EPI_ISL_1750383

EPI_ISL_1750384

EPI_ISL_1750385

EPI_ISL_1750386

EPI_ISL_1750387

EPI_ISL_1750388

EPI_ISL_1750389

EPI_ISL_1750390

EPI_ISL_1750391

EPI_ISL_1750392

EPI_ISL_1750393

EPI_ISL_1750394

EPI_ISL_1750395

EPI_ISL_1750396

EPI_ISL_1750397

EPI_ISL_1750398

EPI_ISL_1750399

EPI_ISL_1750400

EPI_ISL_1750401

EPI_ISL_1750402

EPI_ISL_1750403

EPI_ISL_1750404

EPI_ISL_1750405

EPI_ISL_1750406

EPI_ISL_1750407

EPI_ISL_1750408

EPI_ISL_1750409

EPI_ISL_1750410

EPI_ISL_1750411

EPI_ISL_1750412

EPI_ISL_1750413

EPI_ISL_1750414

EPI_ISL_1750415

EPI_ISL_1750416

EPI_ISL_1750417

EPI_ISL_1750418

EPI_ISL_1750419

EPI_ISL_1750420

EPI_ISL_1750421

EPI_ISL_1750422

EPI_ISL_1750423

EPI_ISL_1750424

EPI_ISL_1750425

EPI_ISL_1750426

EPI_ISL_1750427

EPI_ISL_1750428

EPI_ISL_1750429

EPI_ISL_1750430

EPI_ISL_1750431

EPI_ISL_1750432

EPI_ISL_1750433

EPI_ISL_1750434

EPI_ISL_1750435

EPI_ISL_1750436

EPI_ISL_1750437

EPI_ISL_1750438

EPI_ISL_1750439

EPI_ISL_1750440

EPI_ISL_1750441

EPI_ISL_1750442

EPI_ISL_1750443

EPI_ISL_1750444

EPI_ISL_1750445

EPI_ISL_1750446

EPI_ISL_1750447

EPI_ISL_1750448

EPI_ISL_1750449

EPI_ISL_1750450

EPI_ISL_1750451

EPI_ISL_1750452

EPI_ISL_1750453

EPI_ISL_1750454

EPI_ISL_1750455

EPI_ISL_1750456

EPI_ISL_1750457

EPI_ISL_1750458

EPI_ISL_1750459

EPI_ISL_1750460

EPI_ISL_1750461

EPI_ISL_1750462

EPI_ISL_1750463

EPI_ISL_1750464

EPI_ISL_1750465

EPI_ISL_1750466

EPI_ISL_1750467

EPI_ISL_1750468

EPI_ISL_1750469

EPI_ISL_1750470

EPI_ISL_1750471

EPI_ISL_1750472

EPI_ISL_1750473

EPI_ISL_1750474

EPI_ISL_1750475

EPI_ISL_1750476

EPI_ISL_1750477

EPI_ISL_1750478

EPI_ISL_1750479

EPI_ISL_1750480

EPI_ISL_1750481

EPI_ISL_1750482

EPI_ISL_1750483

EPI_ISL_1750484

EPI_ISL_1750485

EPI_ISL_1750486

EPI_ISL_1750487

EPI_ISL_1750488

EPI_ISL_1750489

EPI_ISL_1750490

EPI_ISL_1750491

EPI_ISL_1750492

EPI_ISL_1750493

EPI_ISL_1750494

EPI_ISL_1750495

EPI_ISL_1750496

EPI_ISL_1750497

EPI_ISL_1750498

EPI_ISL_1750499

EPI_ISL_1750507

EPI_ISL_1750508

EPI_ISL_1750509

EPI_ISL_1750510

EPI_ISL_1750512

EPI_ISL_1750513

EPI_ISL_1750514

EPI_ISL_1750516

EPI_ISL_1750517

EPI_ISL_1750518

EPI_ISL_1758232

EPI_ISL_1758233

EPI_ISL_1758234

EPI_ISL_1750963

EPI_ISL_1750968

EPI_ISL_1752571

EPI_ISL_1752572

EPI_ISL_1752576

EPI_ISL_1752577

EPI_ISL_1752578

EPI_ISL_1752580

EPI_ISL_1752581

EPI_ISL_1752582

EPI_ISL_1752583

EPI_ISL_1752584

EPI_ISL_1752586

EPI_ISL_1752644

EPI_ISL_1752670

EPI_ISL_1752672

EPI_ISL_1752680

EPI_ISL_1752681

EPI_ISL_1752683

EPI_ISL_1752684

EPI_ISL_1752685

EPI_ISL_1752686

EPI_ISL_1752688

EPI_ISL_1752762

EPI_ISL_1752763

EPI_ISL_1752764

EPI_ISL_1752765

EPI_ISL_1752767

EPI_ISL_1752768

EPI_ISL_1752769

EPI_ISL_1752770

EPI_ISL_1752771

EPI_ISL_1752772

EPI_ISL_1752773

EPI_ISL_1752775

EPI_ISL_1752776

EPI_ISL_1752777

EPI_ISL_1752778

EPI_ISL_1752780

EPI_ISL_1752781

EPI_ISL_1752782

EPI_ISL_1752783

EPI_ISL_1752784

EPI_ISL_1752785

EPI_ISL_1752786

EPI_ISL_1752787

EPI_ISL_1752788

EPI_ISL_1752789

EPI_ISL_1752792

EPI_ISL_1752795

EPI_ISL_1752796

EPI_ISL_1752797

EPI_ISL_1752798

EPI_ISL_1752800

EPI_ISL_1752801

EPI_ISL_1752802

EPI_ISL_1752806

EPI_ISL_1752807

EPI_ISL_1752809

EPI_ISL_1752810

EPI_ISL_1752811

EPI_ISL_1752812

EPI_ISL_1752813

EPI_ISL_1752814

EPI_ISL_1752815

EPI_ISL_1752816

EPI_ISL_1752817

EPI_ISL_1752818

EPI_ISL_1752819

EPI_ISL_1752821

EPI_ISL_1752825

EPI_ISL_1752826

EPI_ISL_1752827

EPI_ISL_1752829

EPI_ISL_1752830

EPI_ISL_1752831

EPI_ISL_1752832

EPI_ISL_1752833

EPI_ISL_1752834

EPI_ISL_1752836

EPI_ISL_1752837

EPI_ISL_1752838

EPI_ISL_1752840

EPI_ISL_1752841

EPI_ISL_1752842

EPI_ISL_1752843

EPI_ISL_1752844

EPI_ISL_1752846

EPI_ISL_1752847

EPI_ISL_1752848

EPI_ISL_1752850

EPI_ISL_1752852

EPI_ISL_1752853

EPI_ISL_1752854

EPI_ISL_1752855

EPI_ISL_1752856

EPI_ISL_1752857

EPI_ISL_1752859

EPI_ISL_1752860

EPI_ISL_1752861

EPI_ISL_1752862

EPI_ISL_1752863

EPI_ISL_1752864

EPI_ISL_1752865

EPI_ISL_1752866

EPI_ISL_1752867

EPI_ISL_1752868

EPI_ISL_1752869

EPI_ISL_1752870

EPI_ISL_1752871

EPI_ISL_1752872

EPI_ISL_1752873

EPI_ISL_1752874

EPI_ISL_1752877

EPI_ISL_1752878

EPI_ISL_1752880

EPI_ISL_1752881

EPI_ISL_1752882

EPI_ISL_1752883

EPI_ISL_1752885

EPI_ISL_1752886

EPI_ISL_1752887

EPI_ISL_1752888

EPI_ISL_1752889

EPI_ISL_1752890

EPI_ISL_1752891

EPI_ISL_1752892

EPI_ISL_1752893

EPI_ISL_1752894

EPI_ISL_1752895

EPI_ISL_1752896

EPI_ISL_1752897

EPI_ISL_1752899

EPI_ISL_1752900

EPI_ISL_1752901

EPI_ISL_1752904

EPI_ISL_1752905

EPI_ISL_1752906

EPI_ISL_1752907

EPI_ISL_1752908

EPI_ISL_1752910

EPI_ISL_1752911

EPI_ISL_1752912

EPI_ISL_1752913

EPI_ISL_1752914

EPI_ISL_1752916

EPI_ISL_1752923

EPI_ISL_1752924

EPI_ISL_1752925

EPI_ISL_1752926

EPI_ISL_1752927

EPI_ISL_1752928

EPI_ISL_1752929

EPI_ISL_1752930

EPI_ISL_1752931

EPI_ISL_1752932

EPI_ISL_1752933

EPI_ISL_1752934

EPI_ISL_1752935

EPI_ISL_1752936

EPI_ISL_1752937

EPI_ISL_1752939

EPI_ISL_1752941

EPI_ISL_1752944

EPI_ISL_1752945

EPI_ISL_1752946

EPI_ISL_1752947

EPI_ISL_1752952

EPI_ISL_1752953

EPI_ISL_1753019

EPI_ISL_1753026

EPI_ISL_1753028

EPI_ISL_1753030

EPI_ISL_1753031

EPI_ISL_1753034

EPI_ISL_1753035

EPI_ISL_1753037

EPI_ISL_1753038

EPI_ISL_1753039

EPI_ISL_1753043

EPI_ISL_1753044

EPI_ISL_1753045

EPI_ISL_1753047

EPI_ISL_1753051

EPI_ISL_1753052

EPI_ISL_1753054

EPI_ISL_1753055

EPI_ISL_1753057

EPI_ISL_1753058

EPI_ISL_1753059

EPI_ISL_1753060

EPI_ISL_1753061

EPI_ISL_1753064

EPI_ISL_1753068

EPI_ISL_1753070

EPI_ISL_1753073

EPI_ISL_1753074

EPI_ISL_1753075

EPI_ISL_1753076

EPI_ISL_1753077

EPI_ISL_1753078

EPI_ISL_1753081

EPI_ISL_1753082

EPI_ISL_1753083

EPI_ISL_1753084

EPI_ISL_1753086

EPI_ISL_1753087

EPI_ISL_1753088

EPI_ISL_1753089

EPI_ISL_1753091

EPI_ISL_1753092

EPI_ISL_1753096

EPI_ISL_1753100

EPI_ISL_1753101

EPI_ISL_1753102

EPI_ISL_1753106

EPI_ISL_1753109

EPI_ISL_1753110

EPI_ISL_1753111

EPI_ISL_1753113

EPI_ISL_1753114

EPI_ISL_1753115

EPI_ISL_1753116

EPI_ISL_1753117

EPI_ISL_1753123

EPI_ISL_1753127

EPI_ISL_1753130

EPI_ISL_1753131

EPI_ISL_1753132

EPI_ISL_1753135

EPI_ISL_1753137

EPI_ISL_1753139

EPI_ISL_1753141

EPI_ISL_1753142

EPI_ISL_1753143

EPI_ISL_1753146

EPI_ISL_1753147

EPI_ISL_1753148

EPI_ISL_1753150

EPI_ISL_1753151

EPI_ISL_1753153

EPI_ISL_1753156

EPI_ISL_1753158

EPI_ISL_1753159

EPI_ISL_1753160

EPI_ISL_1753161

EPI_ISL_1753163

EPI_ISL_1753164

EPI_ISL_1753166

EPI_ISL_1753168

EPI_ISL_1753169

EPI_ISL_1753171

EPI_ISL_1753172

EPI_ISL_1753173

EPI_ISL_1753175

EPI_ISL_1753178

EPI_ISL_1753179

EPI_ISL_1753180

EPI_ISL_1753182

EPI_ISL_1753184

EPI_ISL_1753186

EPI_ISL_1753192

EPI_ISL_1753196

EPI_ISL_1753199

EPI_ISL_1753205

EPI_ISL_1753206

EPI_ISL_1753209

EPI_ISL_1753213

EPI_ISL_1753214

EPI_ISL_1753217

EPI_ISL_1753221

EPI_ISL_1753222

EPI_ISL_1753223

EPI_ISL_1753224

EPI_ISL_1753227

EPI_ISL_1753229

EPI_ISL_1753231

EPI_ISL_1753232

EPI_ISL_1753233

EPI_ISL_1753235

EPI_ISL_1753237

EPI_ISL_1753238

EPI_ISL_1753239

EPI_ISL_1753240

EPI_ISL_1753244

EPI_ISL_1753246

EPI_ISL_1753247

EPI_ISL_1753249

EPI_ISL_1753251

EPI_ISL_1753253

EPI_ISL_1753254

EPI_ISL_1753258

EPI_ISL_1753259

EPI_ISL_1753261

EPI_ISL_1753262

EPI_ISL_1753263

EPI_ISL_1753267

EPI_ISL_1753268

EPI_ISL_1753269

EPI_ISL_1753273

EPI_ISL_1753275

EPI_ISL_1753276

EPI_ISL_1753277

EPI_ISL_1753278

EPI_ISL_1753283

EPI_ISL_1753284

EPI_ISL_1753285

EPI_ISL_1753286

EPI_ISL_1753289

EPI_ISL_1753292

EPI_ISL_1753293

EPI_ISL_1753294

EPI_ISL_1753296

EPI_ISL_1753297

EPI_ISL_1753298

EPI_ISL_1753299

EPI_ISL_1753305

EPI_ISL_1753306

EPI_ISL_1753308

EPI_ISL_1753310

EPI_ISL_1753311

EPI_ISL_1753312

EPI_ISL_1753315

EPI_ISL_1753316

EPI_ISL_1753317

EPI_ISL_1753324

EPI_ISL_1753325

EPI_ISL_1753334

EPI_ISL_1753335

EPI_ISL_1753336

EPI_ISL_1753337

EPI_ISL_1753338

EPI_ISL_1753339

EPI_ISL_1753340

EPI_ISL_1753341

EPI_ISL_1753342

EPI_ISL_1753343

EPI_ISL_1753344

EPI_ISL_1753345

EPI_ISL_1753346

EPI_ISL_1753348

EPI_ISL_1753351

EPI_ISL_1753353

EPI_ISL_1753355

EPI_ISL_1753358

EPI_ISL_1753360

EPI_ISL_1753361

EPI_ISL_1753362

EPI_ISL_1753364

EPI_ISL_1753366

EPI_ISL_1753369

EPI_ISL_1753374

EPI_ISL_1753376

EPI_ISL_1753377

EPI_ISL_1753380

EPI_ISL_1753384

EPI_ISL_1753385

EPI_ISL_1753386

EPI_ISL_1753387

EPI_ISL_1753388

EPI_ISL_1753390

EPI_ISL_1753391

EPI_ISL_1753393

EPI_ISL_1753394

EPI_ISL_1753395

EPI_ISL_1753397

EPI_ISL_1753399

EPI_ISL_1753400

EPI_ISL_1753403

EPI_ISL_1753405

EPI_ISL_1753407

EPI_ISL_1753409

EPI_ISL_1753415

EPI_ISL_1753417

EPI_ISL_1753419

EPI_ISL_1753421

EPI_ISL_1753423

EPI_ISL_1753451

EPI_ISL_1753457

EPI_ISL_1753462

EPI_ISL_1753463

EPI_ISL_1753465

EPI_ISL_1753469

EPI_ISL_1753471

EPI_ISL_1753473

EPI_ISL_1753477

EPI_ISL_1753478

EPI_ISL_1753483

EPI_ISL_1753484

EPI_ISL_1753486

EPI_ISL_1753488

EPI_ISL_1753495

EPI_ISL_1753496

EPI_ISL_1753497

EPI_ISL_1753498

EPI_ISL_1753499

EPI_ISL_1753500

EPI_ISL_1753501

EPI_ISL_1753504

EPI_ISL_1753512

EPI_ISL_1753513

EPI_ISL_1753514

EPI_ISL_1753522

EPI_ISL_1753526

EPI_ISL_1753535

EPI_ISL_1753542

EPI_ISL_1753543

EPI_ISL_1753547

EPI_ISL_1753550

EPI_ISL_1753551

EPI_ISL_1753557

EPI_ISL_1753565

EPI_ISL_1753566

EPI_ISL_1753588

EPI_ISL_1753589

EPI_ISL_1753603

EPI_ISL_1753604

EPI_ISL_1753606

EPI_ISL_1753608

EPI_ISL_1753609

EPI_ISL_1753610

EPI_ISL_1753611

EPI_ISL_1753662

EPI_ISL_1753663

EPI_ISL_1753664

EPI_ISL_1753665

EPI_ISL_1753666

EPI_ISL_1753667

EPI_ISL_1753671

EPI_ISL_1753677

EPI_ISL_1753679

EPI_ISL_1753682

EPI_ISL_1753683

EPI_ISL_1753684

EPI_ISL_1753686

EPI_ISL_1753687

EPI_ISL_1753689

EPI_ISL_1753690

EPI_ISL_1753691

EPI_ISL_1753692

EPI_ISL_1753693

EPI_ISL_1753694

EPI_ISL_1753695

EPI_ISL_1753696

EPI_ISL_1753697

EPI_ISL_1753698

EPI_ISL_1753701

EPI_ISL_1753702

EPI_ISL_1753703

EPI_ISL_1753704

EPI_ISL_1753705

EPI_ISL_1753706

EPI_ISL_1753711

EPI_ISL_1753713

EPI_ISL_1753714

EPI_ISL_1753715

EPI_ISL_1753716

EPI_ISL_1753717

EPI_ISL_1753718

EPI_ISL_1753719

EPI_ISL_1753720

EPI_ISL_1753721

EPI_ISL_1753723

EPI_ISL_1753724

EPI_ISL_1753725

EPI_ISL_1753726

EPI_ISL_1753727

EPI_ISL_1753728

EPI_ISL_1753729

EPI_ISL_1753730

EPI_ISL_1753731

EPI_ISL_1753732

EPI_ISL_1753734

EPI_ISL_1753736

EPI_ISL_1753737

EPI_ISL_1753738

EPI_ISL_1753739

EPI_ISL_1753740

EPI_ISL_1753741

EPI_ISL_1753742

EPI_ISL_1753743

EPI_ISL_1753744

EPI_ISL_1753745

EPI_ISL_1753746

EPI_ISL_1753747

EPI_ISL_1753748

EPI_ISL_1753749

EPI_ISL_1753750

EPI_ISL_1753751

EPI_ISL_1753752

EPI_ISL_1753759

EPI_ISL_1753760

EPI_ISL_1753761

EPI_ISL_1753762

EPI_ISL_1753763

EPI_ISL_1753764

EPI_ISL_1753765

EPI_ISL_1753766

EPI_ISL_1753767

EPI_ISL_1753768

EPI_ISL_1753769

EPI_ISL_1753770

EPI_ISL_1753771

EPI_ISL_1753772

EPI_ISL_1753773

EPI_ISL_1753774

EPI_ISL_1753775

EPI_ISL_1753776

EPI_ISL_1753778

EPI_ISL_1753779

EPI_ISL_1753780

EPI_ISL_1753783

EPI_ISL_1753785

EPI_ISL_1753786

EPI_ISL_1753787

EPI_ISL_1753788

EPI_ISL_1753789

EPI_ISL_1753790

EPI_ISL_1753791

EPI_ISL_1753792

EPI_ISL_1753793

EPI_ISL_1753795

EPI_ISL_1753796

EPI_ISL_1753798

EPI_ISL_1753799

EPI_ISL_1753800

EPI_ISL_1753802

EPI_ISL_1753803

EPI_ISL_1753804

EPI_ISL_1753805

EPI_ISL_1753808

EPI_ISL_1753809

EPI_ISL_1753810

EPI_ISL_1753812

EPI_ISL_1753814

EPI_ISL_1753815

EPI_ISL_1753816

EPI_ISL_1753817

EPI_ISL_1753818

EPI_ISL_1753819

EPI_ISL_1753820

EPI_ISL_1753821

EPI_ISL_1753823

EPI_ISL_1753824

EPI_ISL_1753825

EPI_ISL_1753826

EPI_ISL_1753828

EPI_ISL_1753829

EPI_ISL_1753830

EPI_ISL_1753831

EPI_ISL_1753836

EPI_ISL_1753838

EPI_ISL_1754123

EPI_ISL_1754125

EPI_ISL_1754130

EPI_ISL_1754132

EPI_ISL_1754138

EPI_ISL_1754165

EPI_ISL_1754166

EPI_ISL_1754175

EPI_ISL_1754176

EPI_ISL_1754178

EPI_ISL_1754180

EPI_ISL_1754181

EPI_ISL_1754862

EPI_ISL_1754865

EPI_ISL_1754878

EPI_ISL_1754889

EPI_ISL_1754894

EPI_ISL_1754917

EPI_ISL_1755005

EPI_ISL_1755006

EPI_ISL_1755007

EPI_ISL_1755266

EPI_ISL_1755268

EPI_ISL_1755329

EPI_ISL_1755333

EPI_ISL_1755336

EPI_ISL_1755337

EPI_ISL_1755342

EPI_ISL_1755346

EPI_ISL_1755351

EPI_ISL_1755355

EPI_ISL_1755356

EPI_ISL_1755357

EPI_ISL_1755361

EPI_ISL_1755362

EPI_ISL_1755363

EPI_ISL_1755364

EPI_ISL_1755365

EPI_ISL_1755670

EPI_ISL_1755672

EPI_ISL_1755673

EPI_ISL_1755694

EPI_ISL_1755697

EPI_ISL_1755699

EPI_ISL_1755700

EPI_ISL_1755706

EPI_ISL_1755709

EPI_ISL_1755710

EPI_ISL_1755711

EPI_ISL_1755712

EPI_ISL_1755714

EPI_ISL_1755739

EPI_ISL_1755743

EPI_ISL_1755744

EPI_ISL_1755745

EPI_ISL_1755746

EPI_ISL_1755747

EPI_ISL_1758235

EPI_ISL_1755795

EPI_ISL_1755845

EPI_ISL_1755847

EPI_ISL_1755848

EPI_ISL_1755885

EPI_ISL_1755887

EPI_ISL_1755888

EPI_ISL_1755889

EPI_ISL_1755890

EPI_ISL_1755891

EPI_ISL_1755892

EPI_ISL_1755893

EPI_ISL_1755894

EPI_ISL_1755895

EPI_ISL_1755897

EPI_ISL_1755898

EPI_ISL_1755899

EPI_ISL_1755992

EPI_ISL_1755993

EPI_ISL_1755994

EPI_ISL_1755995

EPI_ISL_1755996

EPI_ISL_1755997

EPI_ISL_1755998

EPI_ISL_1755999

EPI_ISL_1756001

EPI_ISL_1756006

EPI_ISL_1756008

EPI_ISL_1756009

EPI_ISL_1756010

EPI_ISL_1756011

EPI_ISL_1756012

EPI_ISL_1756013

EPI_ISL_1756014

EPI_ISL_1756016

EPI_ISL_1756017

EPI_ISL_1756020

EPI_ISL_1756021

EPI_ISL_1756025

EPI_ISL_1756127

EPI_ISL_1756128

EPI_ISL_1756130

EPI_ISL_1756142

EPI_ISL_1756143

EPI_ISL_1756145

EPI_ISL_1756146

EPI_ISL_1756149

EPI_ISL_1756150

EPI_ISL_1756153

EPI_ISL_1756154

EPI_ISL_1756155

EPI_ISL_1756156

EPI_ISL_1756161

EPI_ISL_1756163

EPI_ISL_1756166

EPI_ISL_1756168

EPI_ISL_1756184

EPI_ISL_1756188

EPI_ISL_1756189

EPI_ISL_1756194

EPI_ISL_1756197

EPI_ISL_1756198

EPI_ISL_1756199

EPI_ISL_1756200

EPI_ISL_1756205

EPI_ISL_1756206

EPI_ISL_1756207

EPI_ISL_1756209

EPI_ISL_1756210

EPI_ISL_1756216

EPI_ISL_1756219

EPI_ISL_1756220

EPI_ISL_1756221

EPI_ISL_1756223

EPI_ISL_1756225

EPI_ISL_1756227

EPI_ISL_1756231

EPI_ISL_1756232

EPI_ISL_1756235

EPI_ISL_1756237

EPI_ISL_1756246

EPI_ISL_1756248

EPI_ISL_1756254

EPI_ISL_1756258

EPI_ISL_1756259

EPI_ISL_1756263

EPI_ISL_1756273

EPI_ISL_1756274

EPI_ISL_1756276

EPI_ISL_1756277

EPI_ISL_1756284

EPI_ISL_1758236

EPI_ISL_1756294

EPI_ISL_1756302

EPI_ISL_1756303

EPI_ISL_1756304

EPI_ISL_1756310

EPI_ISL_1756315

EPI_ISL_1756317

EPI_ISL_1756323

EPI_ISL_1756332

EPI_ISL_1756337

EPI_ISL_1756340

EPI_ISL_1756341

EPI_ISL_1756344

EPI_ISL_1756345

EPI_ISL_1756347

EPI_ISL_1756349

EPI_ISL_1756350

EPI_ISL_1756351

EPI_ISL_1756352

EPI_ISL_1756355

EPI_ISL_1756357

EPI_ISL_1756358

EPI_ISL_1756361

EPI_ISL_1756362

EPI_ISL_1756363

EPI_ISL_1756364

EPI_ISL_1756365

EPI_ISL_1756367

EPI_ISL_1756373

EPI_ISL_1756375

EPI_ISL_1756376

EPI_ISL_1756379

EPI_ISL_1756380

EPI_ISL_1756381

EPI_ISL_1756382

EPI_ISL_1756384

EPI_ISL_1756385

EPI_ISL_1756387

EPI_ISL_1756396

EPI_ISL_1756399

EPI_ISL_1756401

EPI_ISL_1756404

EPI_ISL_1756407

EPI_ISL_1756487

EPI_ISL_1756488

EPI_ISL_1756489

EPI_ISL_1756490

EPI_ISL_1756492

EPI_ISL_1756493

EPI_ISL_1756499

EPI_ISL_1756500

EPI_ISL_1756501

EPI_ISL_1756502

EPI_ISL_1756503

EPI_ISL_1756504

EPI_ISL_1756505

EPI_ISL_1756506

EPI_ISL_1756513

EPI_ISL_1756515

EPI_ISL_1756516

EPI_ISL_1756519

EPI_ISL_1756523

EPI_ISL_1756524

EPI_ISL_1756527

EPI_ISL_1756529

EPI_ISL_1756532

EPI_ISL_1756535

EPI_ISL_1756539

EPI_ISL_1756542

EPI_ISL_1756543

EPI_ISL_1756545

EPI_ISL_1756546

EPI_ISL_1756547

EPI_ISL_1756548

EPI_ISL_1756549

EPI_ISL_1756550

EPI_ISL_1756551

EPI_ISL_1756552

EPI_ISL_1756553

EPI_ISL_1756554

EPI_ISL_1756555

EPI_ISL_1756556

EPI_ISL_1756557

EPI_ISL_1756558

EPI_ISL_1756559

EPI_ISL_1756560

EPI_ISL_1756561

EPI_ISL_1756562

EPI_ISL_1756564

EPI_ISL_1756565

EPI_ISL_1756566

EPI_ISL_1756567

EPI_ISL_1756568

EPI_ISL_1756569

EPI_ISL_1756570

EPI_ISL_1756571

EPI_ISL_1756572

EPI_ISL_1756573

EPI_ISL_1756574

EPI_ISL_1756575

EPI_ISL_1756576

EPI_ISL_1756577

EPI_ISL_1756578

EPI_ISL_1756579

EPI_ISL_1756580

EPI_ISL_1756581

EPI_ISL_1756583

EPI_ISL_1756584

EPI_ISL_1756585

EPI_ISL_1756586

EPI_ISL_1756588

EPI_ISL_1756589

EPI_ISL_1756590

EPI_ISL_1756591

EPI_ISL_1756592

EPI_ISL_1756593

EPI_ISL_1756596

EPI_ISL_1756597

EPI_ISL_1756598

EPI_ISL_1756599

EPI_ISL_1756600

EPI_ISL_1756601

EPI_ISL_1756602

EPI_ISL_1756603

EPI_ISL_1756604

EPI_ISL_1756605

EPI_ISL_1756606

EPI_ISL_1756608

EPI_ISL_1756609

EPI_ISL_1756610

EPI_ISL_1756611

EPI_ISL_1756613

EPI_ISL_1756614

EPI_ISL_1756617

EPI_ISL_1756619

EPI_ISL_1756621

EPI_ISL_1756622

EPI_ISL_1756623

EPI_ISL_1756624

EPI_ISL_1756625

EPI_ISL_1756626

EPI_ISL_1756628

EPI_ISL_1756630

EPI_ISL_1756632

EPI_ISL_1756633

EPI_ISL_1756634

EPI_ISL_1756635

EPI_ISL_1756636

EPI_ISL_1756638

EPI_ISL_1756639

EPI_ISL_1756640

EPI_ISL_1756642

EPI_ISL_1756643

EPI_ISL_1756644

EPI_ISL_1756645

EPI_ISL_1756646

EPI_ISL_1756647

EPI_ISL_1756648

EPI_ISL_1756649

EPI_ISL_1756650

EPI_ISL_1756651

EPI_ISL_1756653

EPI_ISL_1756655

EPI_ISL_1756656

EPI_ISL_1756657

EPI_ISL_1756659

EPI_ISL_1756660

EPI_ISL_1756662

EPI_ISL_1756663

EPI_ISL_1756664

EPI_ISL_1756665

EPI_ISL_1756666

EPI_ISL_1756667

EPI_ISL_1756668

EPI_ISL_1756669

EPI_ISL_1756671

EPI_ISL_1756672

EPI_ISL_1756673

EPI_ISL_1756674

EPI_ISL_1756675

EPI_ISL_1756676

EPI_ISL_1756677

EPI_ISL_1756678

EPI_ISL_1756679

EPI_ISL_1756681

EPI_ISL_1756683

EPI_ISL_1756684

EPI_ISL_1756685

EPI_ISL_1756686

EPI_ISL_1756687

EPI_ISL_1756688

EPI_ISL_1756689

EPI_ISL_1756690

EPI_ISL_1756691

EPI_ISL_1756694

EPI_ISL_1756695

EPI_ISL_1756696

EPI_ISL_1756697

EPI_ISL_1756698

EPI_ISL_1756699

EPI_ISL_1756700

EPI_ISL_1756704

EPI_ISL_1756705

EPI_ISL_1756707

EPI_ISL_1756708

EPI_ISL_1756711

EPI_ISL_1756713

EPI_ISL_1756715

EPI_ISL_1756716

EPI_ISL_1756717

EPI_ISL_1756718

EPI_ISL_1756719

EPI_ISL_1756720

EPI_ISL_1756721

EPI_ISL_1756722

EPI_ISL_1756723

EPI_ISL_1756724

EPI_ISL_1756725

EPI_ISL_1756726

EPI_ISL_1756727

EPI_ISL_1756728

EPI_ISL_1756730

EPI_ISL_1756731

EPI_ISL_1756733

EPI_ISL_1756734

EPI_ISL_1756735

EPI_ISL_1756736

EPI_ISL_1756737

EPI_ISL_1756738

EPI_ISL_1756739

EPI_ISL_1756740

EPI_ISL_1756741

EPI_ISL_1756743

EPI_ISL_1756745

EPI_ISL_1756746

EPI_ISL_1756747

EPI_ISL_1756748

EPI_ISL_1756750

EPI_ISL_1756751

EPI_ISL_1756752

EPI_ISL_1756753

EPI_ISL_1756755

EPI_ISL_1756756

EPI_ISL_1756757

EPI_ISL_1756758

EPI_ISL_1756760

EPI_ISL_1756761

EPI_ISL_1756762

EPI_ISL_1756763

EPI_ISL_1756766

EPI_ISL_1756767

EPI_ISL_1756768

EPI_ISL_1756770

EPI_ISL_1756771

EPI_ISL_1756772

EPI_ISL_1756773

EPI_ISL_1756774

EPI_ISL_1756775

EPI_ISL_1756776

EPI_ISL_1756780

EPI_ISL_1756781

EPI_ISL_1756782

EPI_ISL_1756784

EPI_ISL_1756785

EPI_ISL_1756786

EPI_ISL_1756787

EPI_ISL_1756788

EPI_ISL_1756789

EPI_ISL_1756790

EPI_ISL_1756791

EPI_ISL_1756792

EPI_ISL_1756793

EPI_ISL_1756794

EPI_ISL_1756795

EPI_ISL_1756796

EPI_ISL_1756797

EPI_ISL_1756798

EPI_ISL_1756799

EPI_ISL_1756802

EPI_ISL_1756803

EPI_ISL_1756805

EPI_ISL_1756808

EPI_ISL_1756810

EPI_ISL_1756811

EPI_ISL_1756812

EPI_ISL_1756814

EPI_ISL_1756815

EPI_ISL_1756817

EPI_ISL_1756818

EPI_ISL_1756819

EPI_ISL_1756820

EPI_ISL_1756822

EPI_ISL_1756823

EPI_ISL_1756824

EPI_ISL_1756827

EPI_ISL_1756828

EPI_ISL_1756830

EPI_ISL_1756831

EPI_ISL_1756832

EPI_ISL_1756833

EPI_ISL_1756836

EPI_ISL_1756838

EPI_ISL_1756839

EPI_ISL_1756840

EPI_ISL_1756841

EPI_ISL_1756843

EPI_ISL_1756844

EPI_ISL_1756845

EPI_ISL_1756846

EPI_ISL_1756847

EPI_ISL_1756848

EPI_ISL_1756849

EPI_ISL_1756850

EPI_ISL_1756851

EPI_ISL_1756852

EPI_ISL_1756853

EPI_ISL_1756854

EPI_ISL_1756855

EPI_ISL_1756856

EPI_ISL_1756857

EPI_ISL_1756858

EPI_ISL_1756859

EPI_ISL_1756860

EPI_ISL_1756861

EPI_ISL_1756862

EPI_ISL_1756863

EPI_ISL_1756864

EPI_ISL_1756865

EPI_ISL_1756866

EPI_ISL_1756867

EPI_ISL_1756868

EPI_ISL_1756869

EPI_ISL_1756870

EPI_ISL_1756872

EPI_ISL_1756873

EPI_ISL_1756874

EPI_ISL_1756876

EPI_ISL_1756878

EPI_ISL_1756879

EPI_ISL_1756880

EPI_ISL_1756883

EPI_ISL_1756885

EPI_ISL_1756888

EPI_ISL_1756889

EPI_ISL_1756890

EPI_ISL_1756892

EPI_ISL_1756894

EPI_ISL_1756897

EPI_ISL_1756898

EPI_ISL_1756899

EPI_ISL_1756900

EPI_ISL_1756901

EPI_ISL_1756902

EPI_ISL_1756905

EPI_ISL_1756907

EPI_ISL_1756908

EPI_ISL_1756909

EPI_ISL_1756912

EPI_ISL_1756913

EPI_ISL_1756916

EPI_ISL_1756917

EPI_ISL_1756918

EPI_ISL_1756919

EPI_ISL_1756920

EPI_ISL_1756921

EPI_ISL_1756923

EPI_ISL_1756924

EPI_ISL_1756925

EPI_ISL_1756928

EPI_ISL_1756931

EPI_ISL_1756932

EPI_ISL_1756934

EPI_ISL_1756935

EPI_ISL_1756936

EPI_ISL_1756938

EPI_ISL_1756939

EPI_ISL_1756940

EPI_ISL_1756942

EPI_ISL_1756943

EPI_ISL_1756944

EPI_ISL_1756946

EPI_ISL_1756947

EPI_ISL_1756948

EPI_ISL_1756950

EPI_ISL_1756951

EPI_ISL_1756952

EPI_ISL_1756953

EPI_ISL_1756954

EPI_ISL_1756955

EPI_ISL_1756956

EPI_ISL_1756957

EPI_ISL_1756958

EPI_ISL_1756959

EPI_ISL_1756960

EPI_ISL_1756961

EPI_ISL_1756962

EPI_ISL_1756963

EPI_ISL_1756964

EPI_ISL_1756965

EPI_ISL_1756966

EPI_ISL_1756967

EPI_ISL_1756968

EPI_ISL_1756969

EPI_ISL_1756970

EPI_ISL_1756971

EPI_ISL_1756972

EPI_ISL_1756973

EPI_ISL_1756974

EPI_ISL_1756975

EPI_ISL_1756976

EPI_ISL_1756977

EPI_ISL_1756978

EPI_ISL_1756979

EPI_ISL_1756980

EPI_ISL_1756981

EPI_ISL_1756982

EPI_ISL_1756984

EPI_ISL_1756985

EPI_ISL_1756999

EPI_ISL_1757000

EPI_ISL_1757002

EPI_ISL_1757017

EPI_ISL_1757018

EPI_ISL_1757019

EPI_ISL_1757023

EPI_ISL_1757025

EPI_ISL_1757031

EPI_ISL_1757033

EPI_ISL_1757035

EPI_ISL_1757036

EPI_ISL_1757047

EPI_ISL_1757048

EPI_ISL_1757049

EPI_ISL_1757050

EPI_ISL_1757053

EPI_ISL_1757056

EPI_ISL_1757059

EPI_ISL_1757060

EPI_ISL_1757061

EPI_ISL_1757071

EPI_ISL_1757072

EPI_ISL_1757073

EPI_ISL_1757075

EPI_ISL_1757078

EPI_ISL_1757079

EPI_ISL_1757084

EPI_ISL_1757085

EPI_ISL_1757088

EPI_ISL_1757089

EPI_ISL_1757093

EPI_ISL_1757094

EPI_ISL_1757098

EPI_ISL_1757100

EPI_ISL_1757102

EPI_ISL_1757103

EPI_ISL_1757105

EPI_ISL_1757114

EPI_ISL_1757115

EPI_ISL_1757117

EPI_ISL_1757118

EPI_ISL_1757119

EPI_ISL_1757120

EPI_ISL_1757123

EPI_ISL_1757126

EPI_ISL_1757128

EPI_ISL_1757131

EPI_ISL_1757136

EPI_ISL_1757137

EPI_ISL_1757140

EPI_ISL_1757141

EPI_ISL_1757142

EPI_ISL_1757149

EPI_ISL_1757162

EPI_ISL_1757165

EPI_ISL_1757166

EPI_ISL_1757169

EPI_ISL_1757172

EPI_ISL_1757174

EPI_ISL_1757176

EPI_ISL_1757187

EPI_ISL_1757189

EPI_ISL_1757191

EPI_ISL_1757192

EPI_ISL_1757198

EPI_ISL_1757200

EPI_ISL_1757201

EPI_ISL_1757203

EPI_ISL_1757204

EPI_ISL_1757206

EPI_ISL_1757210

EPI_ISL_1757211

EPI_ISL_1757214

EPI_ISL_1757216

EPI_ISL_1757219

EPI_ISL_1757223

EPI_ISL_1757224

EPI_ISL_1757226

EPI_ISL_1757230

EPI_ISL_1757232

EPI_ISL_1757233

EPI_ISL_1757234

EPI_ISL_1757239

EPI_ISL_1757242

EPI_ISL_1757244

EPI_ISL_1757256

EPI_ISL_1757271

EPI_ISL_1757287

EPI_ISL_1757289

EPI_ISL_1757294

EPI_ISL_1757298

EPI_ISL_1757300

EPI_ISL_1757307

EPI_ISL_1757308

EPI_ISL_1757310

EPI_ISL_1757319

EPI_ISL_1757321

EPI_ISL_1757322

EPI_ISL_1757323

EPI_ISL_1757326

EPI_ISL_1757327

EPI_ISL_1757329

EPI_ISL_1757330

EPI_ISL_1757331

EPI_ISL_1757335

EPI_ISL_1757337

EPI_ISL_1757341

EPI_ISL_1757342

EPI_ISL_1757344

EPI_ISL_1757346

EPI_ISL_1757349

EPI_ISL_1757350

EPI_ISL_1757351

EPI_ISL_1757352

EPI_ISL_1757353

EPI_ISL_1757354

EPI_ISL_1757355

EPI_ISL_1757356

EPI_ISL_1757358

EPI_ISL_1757360

EPI_ISL_1757361

EPI_ISL_1757365

EPI_ISL_1757368

EPI_ISL_1757372

EPI_ISL_1757373

EPI_ISL_1757374

EPI_ISL_1757375

EPI_ISL_1757380

EPI_ISL_1757381

EPI_ISL_1757382

EPI_ISL_1757383

EPI_ISL_1757385

EPI_ISL_1757389

EPI_ISL_1757390

EPI_ISL_1757391

EPI_ISL_1757395

EPI_ISL_1757415

EPI_ISL_1757417

EPI_ISL_1757423

EPI_ISL_1757432

EPI_ISL_1757440

EPI_ISL_1757441

EPI_ISL_1757443

EPI_ISL_1757444

EPI_ISL_1757445

EPI_ISL_1757447

EPI_ISL_1757452

EPI_ISL_1757453

EPI_ISL_1757456

EPI_ISL_1757463

EPI_ISL_1757464

EPI_ISL_1757465

EPI_ISL_1757466

EPI_ISL_1757467

EPI_ISL_1757468

EPI_ISL_1757469

EPI_ISL_1757471

EPI_ISL_1757472

EPI_ISL_1757473

EPI_ISL_1757474

EPI_ISL_1757477

EPI_ISL_1757478

EPI_ISL_1757479

EPI_ISL_1757480

EPI_ISL_1757481

EPI_ISL_1757484

EPI_ISL_1757485

EPI_ISL_1757486

EPI_ISL_1757487

EPI_ISL_1757488

EPI_ISL_1757489

EPI_ISL_1757490

EPI_ISL_1757491

EPI_ISL_1757492

EPI_ISL_1757493

EPI_ISL_1757494

EPI_ISL_1757495

EPI_ISL_1757496

EPI_ISL_1757499

EPI_ISL_1757500

EPI_ISL_1757501

EPI_ISL_1757502

EPI_ISL_1757503

EPI_ISL_1757504

EPI_ISL_1757505

EPI_ISL_1757506

EPI_ISL_1757507

EPI_ISL_1757508

EPI_ISL_1757509

EPI_ISL_1757510

EPI_ISL_1757511

EPI_ISL_1757512

EPI_ISL_1757513

EPI_ISL_1757514

EPI_ISL_1757515

EPI_ISL_1757516

EPI_ISL_1757517

EPI_ISL_1757518

EPI_ISL_1757519

EPI_ISL_1757520

EPI_ISL_1757522

EPI_ISL_1757523

EPI_ISL_1757524

EPI_ISL_1757525

EPI_ISL_1757526

EPI_ISL_1757527

EPI_ISL_1757529

EPI_ISL_1757530

EPI_ISL_1757531

EPI_ISL_1757533

EPI_ISL_1757534

EPI_ISL_1757535

EPI_ISL_1757536

EPI_ISL_1757540

EPI_ISL_1757541

EPI_ISL_1757542

EPI_ISL_1757544

EPI_ISL_1757551

EPI_ISL_1757553

EPI_ISL_1757554

EPI_ISL_1757556

EPI_ISL_1757557

EPI_ISL_1757561

EPI_ISL_1757562

EPI_ISL_1757564

EPI_ISL_1757565

EPI_ISL_1757568

EPI_ISL_1757569

EPI_ISL_1757570

EPI_ISL_1757577

EPI_ISL_1757578

EPI_ISL_1757580

EPI_ISL_1757585

EPI_ISL_1757588

EPI_ISL_1757589

EPI_ISL_1757591

EPI_ISL_1757593

EPI_ISL_1757594

EPI_ISL_1757596

EPI_ISL_1757598

EPI_ISL_1757599

EPI_ISL_1757602

EPI_ISL_1757603

EPI_ISL_1757604

EPI_ISL_1757608

EPI_ISL_1757609

EPI_ISL_1757614

EPI_ISL_1757619

EPI_ISL_1757623

EPI_ISL_1757628

EPI_ISL_1757645

EPI_ISL_1757646

EPI_ISL_1757647

EPI_ISL_1757648

EPI_ISL_1757650

EPI_ISL_1757651

EPI_ISL_1757652

EPI_ISL_1757653

EPI_ISL_1757654

EPI_ISL_1757658

EPI_ISL_1757659

EPI_ISL_1757660

EPI_ISL_1757661

EPI_ISL_1757662

EPI_ISL_1757663

EPI_ISL_1757664

EPI_ISL_1757666

EPI_ISL_1757667

EPI_ISL_1757668

EPI_ISL_1757670

EPI_ISL_1757671

EPI_ISL_1757674

EPI_ISL_1757677

EPI_ISL_1757681

EPI_ISL_1757687

EPI_ISL_1757688

EPI_ISL_1757690

EPI_ISL_1757691

EPI_ISL_1757692

EPI_ISL_1757693

EPI_ISL_1757697

EPI_ISL_1757698

EPI_ISL_1757699

EPI_ISL_1757700

EPI_ISL_1757701

EPI_ISL_1757702

EPI_ISL_1757703

EPI_ISL_1757705

EPI_ISL_1757714

EPI_ISL_1757722

EPI_ISL_1757723

EPI_ISL_1757724

EPI_ISL_1757725

EPI_ISL_1757726

EPI_ISL_1757727

EPI_ISL_1757731

EPI_ISL_1757732

EPI_ISL_1757733

EPI_ISL_1757734

EPI_ISL_1757735

EPI_ISL_1757737

EPI_ISL_1757738

EPI_ISL_1757740

EPI_ISL_1757742

EPI_ISL_1757743

EPI_ISL_1757744

EPI_ISL_1757745

EPI_ISL_1757747

EPI_ISL_1757748

EPI_ISL_1757749

EPI_ISL_1757751

EPI_ISL_1757752

EPI_ISL_1757754

EPI_ISL_1757755

EPI_ISL_1757756

EPI_ISL_1757757

EPI_ISL_1757759

EPI_ISL_1757761

EPI_ISL_1757762

EPI_ISL_1757765

EPI_ISL_1757768

EPI_ISL_1757776

EPI_ISL_1757780

EPI_ISL_1757781

EPI_ISL_1757784

EPI_ISL_1757786

EPI_ISL_1757787

EPI_ISL_1757788

EPI_ISL_1757790

EPI_ISL_1757791

EPI_ISL_1757792

EPI_ISL_1757793

EPI_ISL_1757795

EPI_ISL_1757796

EPI_ISL_1757797

EPI_ISL_1757798

EPI_ISL_1757799

EPI_ISL_1757800

EPI_ISL_1757801

EPI_ISL_1757802

EPI_ISL_1757803

EPI_ISL_1757804

EPI_ISL_1757809

EPI_ISL_1757812

EPI_ISL_1757815

EPI_ISL_1757816

EPI_ISL_1757817

EPI_ISL_1757818

EPI_ISL_1757820

EPI_ISL_1757827

EPI_ISL_1757828

EPI_ISL_1757829

EPI_ISL_1757830

EPI_ISL_1757833

EPI_ISL_1757836

EPI_ISL_1757837

EPI_ISL_1757840

EPI_ISL_1757842

EPI_ISL_1757843

EPI_ISL_1757844

EPI_ISL_1757875

EPI_ISL_1757876

EPI_ISL_1757878

EPI_ISL_1757880

EPI_ISL_1757888

EPI_ISL_1757889

EPI_ISL_1757897

EPI_ISL_1757900

EPI_ISL_1757902

EPI_ISL_1757903

EPI_ISL_1757906

EPI_ISL_1757909

EPI_ISL_1757917

EPI_ISL_1757924

EPI_ISL_1757925

EPI_ISL_1757926

EPI_ISL_1757927

EPI_ISL_1757928

EPI_ISL_1757930

EPI_ISL_1757933

EPI_ISL_1757936

EPI_ISL_1757937

EPI_ISL_1757940

EPI_ISL_1757941

EPI_ISL_1757942

EPI_ISL_1757943

EPI_ISL_1757944

EPI_ISL_1757945

EPI_ISL_1757946

EPI_ISL_1757951

EPI_ISL_1757953

EPI_ISL_1757955

EPI_ISL_1757956

EPI_ISL_1757958

EPI_ISL_1757960

EPI_ISL_1757963

EPI_ISL_1757965

EPI_ISL_1757966

EPI_ISL_1757967

EPI_ISL_1757968

EPI_ISL_1757969

EPI_ISL_1757970

EPI_ISL_1757971

EPI_ISL_1757972

EPI_ISL_1757976

EPI_ISL_1757979

EPI_ISL_1757980

EPI_ISL_1757982

EPI_ISL_1757983

EPI_ISL_1757985

EPI_ISL_1757986

EPI_ISL_1757988

EPI_ISL_1757989

EPI_ISL_1757990

EPI_ISL_1757992

EPI_ISL_1757993

EPI_ISL_1757994

EPI_ISL_1757997

EPI_ISL_1757998

EPI_ISL_1757999

EPI_ISL_1758000

EPI_ISL_1758001

EPI_ISL_1758002

EPI_ISL_1758003

EPI_ISL_1758005

EPI_ISL_1758006

EPI_ISL_1758007

EPI_ISL_1758008

EPI_ISL_1758010

EPI_ISL_1758012

EPI_ISL_1758013

EPI_ISL_1758014

EPI_ISL_1758019

EPI_ISL_1758021

EPI_ISL_1758022

EPI_ISL_1758023

EPI_ISL_1758024

EPI_ISL_1758025

EPI_ISL_1758027

EPI_ISL_1758028

EPI_ISL_1758029

EPI_ISL_1758030

EPI_ISL_1758032

EPI_ISL_1758033

EPI_ISL_1758034

EPI_ISL_1758036

EPI_ISL_1758037

EPI_ISL_1758039

EPI_ISL_1758040

EPI_ISL_1758041

EPI_ISL_1758043

EPI_ISL_1758044

EPI_ISL_1758045

EPI_ISL_1758046

EPI_ISL_1758047

EPI_ISL_1758048

EPI_ISL_1758050

EPI_ISL_1758051

EPI_ISL_1758052

EPI_ISL_1758053

EPI_ISL_1758054

EPI_ISL_1758055

EPI_ISL_1758056

EPI_ISL_1758058

EPI_ISL_1758059

EPI_ISL_1758060

EPI_ISL_1758061

EPI_ISL_1758062

EPI_ISL_1758237

EPI_ISL_1758238

EPI_ISL_1758064

EPI_ISL_1758065

EPI_ISL_1758066

EPI_ISL_1758067

EPI_ISL_1758071

EPI_ISL_1758088

EPI_ISL_1758103

EPI_ISL_1758109

EPI_ISL_1758110

EPI_ISL_1758111

EPI_ISL_1758112

EPI_ISL_1758117

EPI_ISL_1758120

EPI_ISL_1758121

EPI_ISL_1758122

EPI_ISL_1758124

EPI_ISL_1758127

EPI_ISL_1758130

EPI_ISL_1758131

EPI_ISL_1758132

EPI_ISL_1758134

EPI_ISL_1758137

EPI_ISL_1758145

EPI_ISL_1758146

EPI_ISL_1758152

EPI_ISL_1758153

EPI_ISL_1758154

EPI_ISL_1758155

EPI_ISL_1758161

EPI_ISL_1758162

EPI_ISL_1758163

EPI_ISL_1758164

EPI_ISL_1758171

EPI_ISL_1758172

EPI_ISL_1758173

EPI_ISL_1758175

EPI_ISL_1758182

EPI_ISL_1758183

EPI_ISL_1758184

EPI_ISL_1758185

EPI_ISL_1758187

EPI_ISL_1758188

EPI_ISL_1758189

EPI_ISL_1758191

EPI_ISL_1758192

EPI_ISL_1758193

EPI_ISL_1758194

EPI_ISL_1758195

EPI_ISL_1758197

EPI_ISL_1758198

EPI_ISL_1758199

EPI_ISL_1758200

EPI_ISL_1758201

EPI_ISL_1758202

EPI_ISL_1758203

EPI_ISL_1758204

EPI_ISL_1758206

EPI_ISL_1758207

EPI_ISL_1758208

EPI_ISL_1758209

EPI_ISL_1758210

EPI_ISL_1758212

EPI_ISL_1758213

EPI_ISL_1758214

EPI_ISL_1758215

EPI_ISL_1758216

EPI_ISL_1758217

EPI_ISL_1758218

EPI_ISL_1758219

EPI_ISL_1758220

EPI_ISL_1758221

EPI_ISL_1758224

EPI_ISL_1758225

EPI_ISL_1758239

EPI_ISL_1758240

EPI_ISL_1758241

EPI_ISL_1758244

EPI_ISL_1758245

EPI_ISL_1758246

EPI_ISL_1758247

EPI_ISL_1758248

EPI_ISL_1758249

EPI_ISL_1758250

EPI_ISL_1758251

EPI_ISL_1758252

EPI_ISL_1758253

EPI_ISL_1758254

EPI_ISL_1758255

EPI_ISL_1758256

EPI_ISL_1758257

EPI_ISL_1758258

EPI_ISL_1758259

EPI_ISL_1758260

EPI_ISL_1758261

EPI_ISL_1758262

EPI_ISL_1758263

EPI_ISL_1758264

EPI_ISL_1758265

EPI_ISL_1758266

EPI_ISL_1758267

EPI_ISL_1758269

EPI_ISL_1758270

EPI_ISL_1758271

EPI_ISL_1758272

EPI_ISL_1758273

EPI_ISL_1758274

EPI_ISL_1758275

EPI_ISL_1758276

EPI_ISL_1758278

EPI_ISL_1758279

EPI_ISL_1758280

EPI_ISL_1758281

EPI_ISL_1758285

EPI_ISL_1758286

EPI_ISL_1758287

EPI_ISL_1758288

EPI_ISL_1758289

EPI_ISL_1758290

EPI_ISL_1758291

EPI_ISL_1758292

EPI_ISL_1758293

EPI_ISL_1758295

EPI_ISL_1758296

EPI_ISL_1758297

EPI_ISL_1758298

EPI_ISL_1758299

EPI_ISL_1758302

EPI_ISL_1758305

EPI_ISL_1758308

EPI_ISL_1758309

EPI_ISL_1758310

EPI_ISL_1758311

EPI_ISL_1758313

EPI_ISL_1758314

EPI_ISL_1758317

EPI_ISL_1758318

EPI_ISL_1758319

EPI_ISL_1758320

EPI_ISL_1758321

EPI_ISL_1758322

EPI_ISL_1758324

EPI_ISL_1758326

EPI_ISL_1758327

EPI_ISL_1758328

EPI_ISL_1758330

EPI_ISL_1758332

EPI_ISL_1758334

EPI_ISL_1758335

EPI_ISL_1758336

EPI_ISL_1758337

EPI_ISL_1758338

EPI_ISL_1758339

EPI_ISL_1758341

EPI_ISL_1758342

EPI_ISL_1758344

EPI_ISL_1758345

EPI_ISL_1758346

EPI_ISL_1758347

EPI_ISL_1758348

EPI_ISL_1758349

EPI_ISL_1758350

EPI_ISL_1758351

EPI_ISL_1758352

EPI_ISL_1758353

EPI_ISL_1758354

EPI_ISL_1758355

EPI_ISL_1758356

EPI_ISL_1758357

EPI_ISL_1758358

EPI_ISL_1758359

EPI_ISL_1758360

EPI_ISL_1758361

EPI_ISL_1758363

EPI_ISL_1758364

EPI_ISL_1758365

EPI_ISL_1758366

EPI_ISL_1758367

EPI_ISL_1758368

EPI_ISL_1758369

EPI_ISL_1758370

EPI_ISL_1758371

EPI_ISL_1758374

EPI_ISL_1758375

EPI_ISL_1758377

EPI_ISL_1758378

EPI_ISL_1758379

EPI_ISL_1758382

EPI_ISL_1758383

EPI_ISL_1758384

EPI_ISL_1758385

EPI_ISL_1758386

EPI_ISL_1758387

EPI_ISL_1758389

EPI_ISL_1758390

EPI_ISL_1758392

EPI_ISL_1758393

EPI_ISL_1758394

EPI_ISL_1758395

EPI_ISL_1758396

EPI_ISL_1758397

EPI_ISL_1758398

EPI_ISL_1758399

EPI_ISL_1758400

EPI_ISL_1758402

EPI_ISL_1758403

EPI_ISL_1758404

EPI_ISL_1758405

EPI_ISL_1758406

EPI_ISL_1758407

EPI_ISL_1758408

EPI_ISL_1758409

EPI_ISL_1758410

EPI_ISL_1758411

EPI_ISL_1758412

EPI_ISL_1758415

EPI_ISL_1758416

EPI_ISL_1758417

EPI_ISL_1758418

EPI_ISL_1758419

EPI_ISL_1758420

EPI_ISL_1758421

EPI_ISL_1758423

EPI_ISL_1758424

EPI_ISL_1758425

EPI_ISL_1758426

EPI_ISL_1758428

EPI_ISL_1758429

EPI_ISL_1758431

EPI_ISL_1758432

EPI_ISL_1758433

EPI_ISL_1758434

EPI_ISL_1758435

EPI_ISL_1758436

EPI_ISL_1758437

EPI_ISL_1758438

EPI_ISL_1758439

EPI_ISL_1758442

EPI_ISL_1758443

EPI_ISL_1758444

EPI_ISL_1758445

EPI_ISL_1758446

EPI_ISL_1758447

EPI_ISL_1758448

EPI_ISL_1758450

EPI_ISL_1758452

EPI_ISL_1758453

EPI_ISL_1758454

EPI_ISL_1758457

EPI_ISL_1758458

EPI_ISL_1758459

EPI_ISL_1758460

EPI_ISL_1758461

EPI_ISL_1758462

EPI_ISL_1758463

EPI_ISL_1758464

EPI_ISL_1758465

EPI_ISL_1758466

EPI_ISL_1758467

EPI_ISL_1758468

EPI_ISL_1758469

EPI_ISL_1758470

EPI_ISL_1758471

EPI_ISL_1758472

EPI_ISL_1758473

EPI_ISL_1758474

EPI_ISL_1758476

EPI_ISL_1758477

EPI_ISL_1758478

EPI_ISL_1758479

EPI_ISL_1758480

EPI_ISL_1758481

EPI_ISL_1758482

EPI_ISL_1758483

EPI_ISL_1758485

EPI_ISL_1758486

EPI_ISL_1758487

EPI_ISL_1758488

EPI_ISL_1758489

EPI_ISL_1758490

EPI_ISL_1758492

EPI_ISL_1758493

EPI_ISL_1758494

EPI_ISL_1758495

EPI_ISL_1758496

EPI_ISL_1758497

EPI_ISL_1758499

EPI_ISL_1758500

EPI_ISL_1758501

EPI_ISL_1758502

EPI_ISL_1758504

EPI_ISL_1758505

EPI_ISL_1758506

EPI_ISL_1758507

EPI_ISL_1758508

EPI_ISL_1758511

EPI_ISL_1758512

EPI_ISL_1758513

EPI_ISL_1758514

EPI_ISL_1758515

EPI_ISL_1758516

EPI_ISL_1758517

EPI_ISL_1758518

EPI_ISL_1758519

EPI_ISL_1758520

EPI_ISL_1758521

EPI_ISL_1758522

EPI_ISL_1758523

EPI_ISL_1758524

EPI_ISL_1758525

EPI_ISL_1758526

EPI_ISL_1758527

EPI_ISL_1758529

EPI_ISL_1758530

EPI_ISL_1758531

EPI_ISL_1758533

EPI_ISL_1758534

EPI_ISL_1758535

EPI_ISL_1758536

EPI_ISL_1758537

EPI_ISL_1758538

EPI_ISL_1758539

EPI_ISL_1758540
[truncated: 14,379,235 more chars]
